# Supplementary material for: Integrated Gel Electrophoresis and Mass Spectrometry Approach for Detecting and Quantifying Extraneous Milk in Protected Designation of Origin Buffalo Mozzarella Cheese
Source: Foods. 2025 Mar 28;14(7):1193. doi: 10.3390/foods14071193 (PMC11988604; doi:10.3390/foods14071193)
Supplement: Supplementary file 1 [file foods-14-01193-s001.zip › foods-3534996-supplementary.pdf]

Integrated Gel Electrophoresis and Mass Spectrometry Approach for Detecting and Quantifying Extraneous Milk in Protected Designation of Origin Buffalo Mozzarella Cheese

Sabrina De Pascale[1], Giuseppina Garro[2], Silvia Ines Pellicano[3], Andrea Scaloni[1], Stefania Carpino[4], Simonetta Caira[1,*], and Francesco Addeo[2]

1 Proteomics, Metabolomics & Mass Spectrometry Laboratory, Institute for the Animal Production System in the Mediterranean Environment, National Research Council, 80055 Portici, Italy; sabrinadepascale@cnr.it (S.D.P.); andrea.scaloni@cnr.it (A.S.); simonetta.caira@cnr.it (S.C.).

2 Department of Agriculture, University of Naples "Federico II", 80055 Portici, Italy; giuseppina.garro@unina.it (G.G.); doglie42@gmail.com (F.A.).

3 Central Inspectorate for Fraud Repression and Quality Protection of the Agrifood Products and Food, Ministry of Agricultural, Food and Forestry Policies, 06128 Perugia, Italy; s.pellicano@masaf.gov.it.

4 Central Inspectorate for Fraud Repression and Quality Protection of the Agrifood Products and Food, Ministry of Agricultural, Food and Forestry Policies, 00187 Roma, Italy; s.carpino@masaf.gov.it

* Correspondence: simonetta.caira@cnr.it; Tel.: +39 3926081165

SUPPLEMENTARY MATERIAL

Supplementary Material Table S1. MALDI-TOF-MS data for the tryptic digest of the pH 4.6 insoluble fraction of anonymized PDO MdBC cheeses labelled IM1 to IM25. Samples positive for bovine contamination are indicated with the respective quantified adulteration percentages. The symbol "●" indicates the MdBC samples positive for contamination with exogenous milk from additional animal species or non-Mediterranean buffaloes, as determined by nanoLC-ESI-MS/MS analysis. Each sample was analysed in technical triplicate. MALDI-TOF-MS signal intensities of the peptide α_{s1}-CN(8-22) are presented as mean ± standard deviation. Statistical analysis was conducted using one-way ANOVA, followed by Tukey's HSD test ($p < 0.05$).

Protein	Peptide	Species	aa substitution/ deletion	Phos N.	MH$^+$	IM1	IM2	IM3	IM4	IM5	IM6	IM7	IM8	IM9	IM10	IM11	IM12	IM13	IM14	IM15	IM16	IM17	IM18	IM19	IM20	IM21	IM22	IM23	IM24	IM25
α_{s1}-CN	8-22	Buf M			1687,9									98,6±0,5		91,6±0,2		99,2±0,3		99,9±0,3							64,9±0,3			99,9±0,02
		Bov	E→G$_{14}$		1759,9			●		●				●; 0,5±0,02	●	●; 1,6±0,02	●	●; 0,8±0,02	●	●; 0,1±0,0	●	●		●	●	●	●; 35,1±0,1	●	●	●; 0,1±0,0
		Ovine C	P→S$_{12}$; E→P$_{13}$		1718,9									0,8±0,01		6,8±0,04														
	23-34	Bov/Buf M			1384,7									85,3±0,8		73,2±0,4														89,0±0,03
		Buf F	V→M$_{31}$		1416,7							●																		
	23-33	Ovine	G→R$_{33}$		1307,7									14,7±0,2		26,8														11,0±0,02
	37-58	Buf M		2P	2571,0																									
		Bov	T→K$_{42}$	2P	2598,1	●							●				●		●		●	●								
	35-58	Buf F	I→V$_{36}$; del (35-42) P	2P	1913,7																									
	43-58	Bov	T→K$_{42}$	2P	1927,7																						●			
				1P	1847,7																						●			
				0P	1767,8												●								●					
		Ovine	T→I$_{49}$; →A$_{57}$	2P	1896,7																									
				1P	1816,7																									
				0P	1736,7																									
	104-124	Buf M/F			2452,3																									
	104-119	Bov	L→S$_{115}$; Q→R$_{119}$	1P	1952,0													●	●								●	●		
	106-119			1P	1660,8										●	●		●									●		●	
	133-151	Buf M			2315,2									99,1±1,8		96,5±0,3											77,0±0,03			
		Bovine	E→Q$_{148}$		2316,2			●		●				●	●	●	●	●	●		●			●	●	●	●; 23±0,01	●	●	
		Ovine	G→A$_{133}$; E→Q$_{137}$; E→Q$_{148}$		2328,2									0,9±0,02		3,5±0,02														
ß-CN	1-28	Buf Med		4P	3458,5																						39,2±0,02			
				3P	3378,5																									
				2P	3298,5																									
				1P	3218,6																									
				0P	3137,6																									
	1-25	Bov	H→R$_{25}$	4P	3122,3																						60,8±0,01			
				3P	3042,3																									
				2P	2962,3																									
				1P	2882,4																									
				0P	2801,4																									
	1-28	Bov	H→R$_{25}$	4P	3477,5																									
		Buf F	G→S$_{10}$	4P	3488,5																									
				5P	3568,4																									
	1-29	Buf M		4P	3586,5																									
		Bov	H→R$_{25}$	4P	3605,6																									
	33-48	Buf M		1P	2091,8														95,2±0,1		99,6±0,02			99,7±0,02			54,8±0,01			
				0P	2011,9																									
		Bov/Ovi/Buf F	M→T$_{41}$	1P	2061,8														4,8±0,02		0,4±0,02			0,3±0,0			45,2±0,2			
				0P	1981,9																									
	49-68	Buf M			2237,2	98,4±0,2						98,5±0,2				94,8±0,5				32,3±0,05		98,0±0,1		97,8±0,02			75,2±0,1			99,1±0,2
		Bov/Buf F	K→N$_{68}$		2223,2	1,6±0,01						1,5±0,02				5,2±0,01				67,7±0,1		2,0±0,01		2,2±0,01			24,8±0,1			0,9±0,03
		Ovine	T→A$_{55}$; K→N$_{68}$		2197,2																									
	114-132	Buf M/Bov			2168,0																									
		Ovine	N→K$_{131}$		2183,1																									
	133-169	Buf M			4168,2																									
		Bov	P→H$_{148}$;		4208,2																									
		Ovine	L→V$_{40}$; S→P$_{167}$		4165,3																									

Supplementary Material Table S2. MALDI-TOF-MS data for the tryptic digest of the pH 4.6 insoluble fraction of anonymized PDO MdBC cheeses labelled TU1 to TU25. Samples positive for bovine contamination are indicated with the respective quantified adulteration percentages. The symbol "●" indicates the MdBC samples positive for contamination with exogenous milk from additional animal species or non-Mediterranean buffaloes, as determined by nanoLC-ESI-MS/MS analysis. Each sample was analysed in technical triplicate. MALDI-TOF-MS signal intensities of the peptide α_{s1}-CN(8-22) are presented as mean ± standard deviation. Statistical analysis was conducted using one-way ANOVA, followed by Tukey's HSD test ($p < 0.05$).

Protein	Peptide	Species	aa substitution/	Phos	MH+	TU1	TU2	TU3	TU4	TU5	TU6	TU7	TU8	TU9	TU10	TU11	TU12	TU13	TU14	TU15	TU16	TU17	TU18	TU19	TU20	TU21	TU22	TU23	TU24	TU25
α_{s1}-CN	8-22	Buf M			1687,9																	99,8±0,02								
		Bov	E→G_{14}		1759,9						●								●	●		●								
		Ovine C	P→S_{12}; E→P_{13}		1718,9																	0,2±0,01								
	23-34	Bov/Buf M			1384,7																	44,2±0,1								
		Buf F	V→M_{31}		1416,7																									
	23-33	Ovine	G→R_{33}		1307,7																	55,8±0,2								
	37-58	Buf M		2P	2571,0																	71,9±0,1								
		Bov	T→K_{42}	2P	2598,1																									
	35-58	Buf F	I→V_{36}; del (35-42)P	2P	1913,7																									
	43-58	Bov	T→K_{42}	2P	1927,7																	28,1±0,05								
				1P	1847,7																									
				0P	1767,8																									
		Ovine	T→I_{49}; →A_{57}	2P	1896,7																									
				1P	1816,7																									
				0P	1736,7																									
	104-124	Buf M/F			2452,3																									
	104-119	Bov	L→S_{115}; Q→R_{119}	1P	1952,0																		●							●
	106-119			1P	1660,8																									
	133-151	Buf M			2315,2																	95±0,3								
		Bovine	E→Q_{148}		2316,2																									●
		Ovine	G→A_{133}; E→Q_{137}; E→Q_{148}		2328,2																	●; 5,3±0,3								
ß-CN	1-28	Buf Med		4P	3458,5																						60,8±0,02			
				3P	3378,5																									
				2P	3298,5																									
				1P	3218,6																									
				0P	3137,6																									
	1-25	Bov	H→R_{25}	4P	3122,3																						39,2±0,01			
				3P	3042,3																									
				2P	2962,3																									
				1P	2882,4																									
				0P	2801,4																									
	1-28	Bov	H→R_{25}	4P	3477,5																									
		Buf F	G→$\underline{S}_{10}$	4P	3488,5																									
				5P	3568,4																									
	1-29	Buf M		4P	3586,5																									
		Bov	H→R_{25}	4P	3605,6																									
	33-48	Buf M		1P	2091,8																									
				0P	2011,9																									
		Bov/Ovi/Buf F	M→T_{41}	1P	2061,8						●								●	●			●		●		●	●		
				0P	1981,9																				●					
	49-68	Buf M			2237,2																									
		Bov/Buf F	K→N_{68}		2223,2																									
		Ovine	T→A_{55}; K→N_{68}		2197,2																									
	114-132	Buf M/Bov			2168,0																									
		Ovine	N→K_{131}		2183,1																				●					
	133-169	Buf M			4168,2																									
		Bov	P→H_{148};		4208,2																									
		Ovine	L→V_{40}; S→P_{167}		4165,3																		●							
κ-CN	35-68	Ovine	K→R_{46}; A→V_{65}		4065,1																	●								
	69-86	Buf M/F			1989,1																	●								
		Bov	K→R_{46}		1979,1																	●								
		Ovi	K→R_{47}; I→T_{73}		1947,1																	●								

Supplementary Material Table S3. Complete list of peptides identified by nano-LC-ESI-MS/MS in PDO MdBC cheeses labelled IM1 to IM25. Details provided include parent protein, peptide sequence, modification(s), intensity value, localization within protein sequence, and experimental mass value.

Sample IM1

Description	Modifications	# PSMs	Start	End	Theor. MH+ [Da]
**Alpha-S1-casein (Fragment) OS=Bubalus bubalis**					
[R].YLGYLEQLLR.[L]		10	91	100	1267,70
[R].FFVAPFPEVFGK.[E]		9	23	34	1384,73
[K].HQGLPQGVLNENLLR.[F]		5	8	22	1687,92
[K].HQGLPQGVLNENLLR.[F]	1xDeamidated [Q/N]	5	8	22	1688,91
[K].EPMIGVNQELAYFYPQLFR.[Q]		5	133	151	2315,15
[K].EPMIGVNQELAYFYPQLFR.[Q]	1xOxidation [M3]	5	133	151	2331,15
[K].YNVPQLEIVPNLAEEQLHSMK.[E]		5	104	124	2452,25
[K].YNVPQLEIVPNLAEEQLHSMK.[E]	1xOxidation [M20]	5	104	124	2468,25
[K].VNELSTDIGSESTEDQAMEDIK.[Q]	1xPhospho [S10]	4	37	58	2491,04
[K].VNELSTDIGSESTEDQAMEDIK.[Q]	1xPhospho [S12]; 1xOxidation [M18]	4	37	58	2507,04
[K].VNELSTDIGSESTEDQAMEDIK.[Q]	2xPhospho [S/T]	4	37	58	2571,01
[K].KYNVPQLEIVPNLAEEQLHSMK.[E]		5	103	124	2580,35
[K].KYNVPQLEIVPNLAEEQLHSMK.[E]	1xOxidation [M21]	5	103	124	2596,34
[K].VNELSTDIGSESTEDQAMEDIK.[Q]	3xPhospho [T13; T/S]; 1xOxidation [M18]	4	37	58	2666,97
[-].DAYPSGAWYYVPLGTQYPDAPLFSDIPNPIGSENSGK.[T]		1	157	193	3986,88
**Alpha S1 casein OS=Bos taurus**					
[K].VNELSKDIGSESTEDQAMEDIK.[Q]	2xPhospho [S10; S/T]	6	37	58	2598,06
[K].VNELSKDIGSESTEDQAMEDIK.[Q]	2xPhospho [S10; T13]; 1xOxidation [M18]	6	37	58	2614,05
**Beta-casein OS=Bubalus bubalis**					
[K].FQSEEQQQMEDELQDK.[I]	1xOxidation [M9]	1	33	48	2027,85
[K].FQSEEQQQMEDELQDK.[I]	1xPhospho [S3]	1	33	48	2091,82
[K].FQSEEQQQMEDELQDK.[I]	1xDeamidated [Q]; 1xPhospho [S3]	1	33	48	2092,81
[K].FQSEEQQQMEDELQDK.[I]	2xDeamidated [Q]; 1xPhospho [S3]	1	33	48	2093,79
[K].FQSEEQQQMEDELQDK.[I]	3xDeamidated [Q2; Q]; 1xPhospho [S3]	1	33	48	2094,77
[K].FQSEEQQQMEDELQDK.[I]	4xDeamidated [Q2; Q]; 1xPhospho [S3]	1	33	48	2095,76
[K].FQSEEQQQMEDELQDK.[I]	1xPhospho [S3]; 1xOxidation [M9]	1	33	48	2107,82
[K].FQSEEQQQMEDELQDK.[I]	1xDeamidated [Q]; 1xPhospho [S3]; 1xOxidation [M9]	1	33	48	2108,80
[KR].DMPIQAFLLYQEPVLGPVR.[G]		17	184	202	2186,17
[KR].DMPIQAFLLYQEPVLGPVR.[G]	1xDeamidated [Q5]; 1xOxidation [M2]	17	184	202	2203,15
[K-].IHPFAQTQSLVYPFPGPIPK.[S]		3	49	68	2237,21
[K].IEKFQSEEQQQMEDELQDK.[I]	1xPhospho [S6]	1	30	48	2462,04
[K].SLPQNIPPLTQTPVVVPPFLQPEIMGVSK.[V]		8	69	97	3126,73
[K].SLPQNIPPLTQTPVVVPPFLQPEIMGVSK.[V]	1xOxidation [M25]	8	69	97	3142,72
[-].RELEELNVPGEIVESLSSSEESITHINK.[K]	1xDeamidated [N27]; 3xPhospho [S17; S18; S19]	2	1	28	3379,46
[-].RELEELNVPGEIVESLSSSEESITHINK.[K]	4xPhospho [S/T]	2	1	28	3458,45
[-].RELEELNVPGEIVESLSSSEESITHINK.[K]	1xDeamidated [N7]; 4xPhospho [S15; S17; S18; S19]	2	1	28	3459,43
**Kappa-casein OS=Bubalus bubalis**					
[R].SPAQILQWQVLPNTVPAK.[S]		5	69	86	1990,11
[R].SPAQILQWQVLPNTVPAK.[S]	1xDeamidated [N/Q]	5	69	86	1991,10
[R].YPSYGLNYYQQKPVALINNQFLPYPYYAKPAAVR.[S]		20	35	68	4010,06
**Beta-lactoglobulin OS=Bubalus bubalis**					
[R].TPEVDDEALEK.[F]		7	125	135	1245,58
[R].VYVEQLKPTPEGDLEILLQK.[W]	1xDeamidated [Q5]	2	41	60	2313,26
**Alpha-lactalbumin protein variant D OS=Bos taurus**					
[K].VGINYWLAHK.[A]		7	99	108	1200,65

Sample IM2

Description	Modifications	# PSMs	Start	End	Theor. MH+ [Da]
**Alpha-S1-casein (Fragment) OS=Bubalus bubalis**					
[R].YLGYLEQLLR.[L]		10	91	100	1267,70
[R].FFVAPFPEVFGK.[E]		9	23	34	1384,73
[K].HQGLPQGVLNENLLR.[F]		5	8	22	1687,92
[K].HQGLPQGVLNENLLR.[F]	1xDeamidated [N/Q]	5	8	22	1688,91
[K].EPMIGVNQELAYFYPQLFR.[Q]		5	133	151	2315,15
[K].EPMIGVNQELAYFYPQLFR.[Q]	1xDeamidated [N/Q]	5	133	151	2316,14
[K].EPMIGVNQELAYFYPQLFR.[Q]	1xOxidation [M3]	5	133	151	2331,15
[K].YNVPQLEIVPNLAEEQLHSMK.[E]		5	104	124	2452,25
[K].YNVPQLEIVPNLAEEQLHSMK.[E]	1xDeamidated [N2]	5	104	124	2453,24
[K].YNVPQLEIVPNLAEEQLHSMK.[E]	1xOxidation [M20]	5	104	124	2468,25
[K].VNELSTDIGSESTEDQAMEDIK.[Q]	1xPhospho [S10]	4	37	58	2491,04
[K].VNELSTDIGSESTEDQAMEDIK.[Q]	2xPhospho [S/T]	4	37	58	2571,01
[K].KYNVPQLEIVPNLAEEQLHSMK.[E]	1xDeamidated [N/Q]	5	103	124	2581,33
[K].VNELSTDIGSESTEDQAMEDIK.[Q]	2xPhospho [S/T]; 1xOxidation [M18]	4	37	58	2587,00
[K].KYNVPQLEIVPNLAEEQLHSMK.[E]	1xOxidation [M21]	5	103	124	2596,34
[K].KYNVPQLEIVPNLAEEQLHSMK.[E]	1xDeamidated [Q/N]; 1xOxidation [M21]	5	103	124	2597,33
**Beta-casein OS=Bubalus bubalis**					
[K].FQSEEQQQMEDELQDK.[I]	1xOxidation [M9]	1	33	48	2027,85
[K].FQSEEQQQMEDELQDK.[I]	1xPhospho [S3]	1	33	48	2091,82
[K].FQSEEQQQMEDELQDK.[I]	1xDeamidated [Q]; 1xPhospho [S3]	1	33	48	2092,81
[K].FQSEEQQQMEDELQDK.[I]	2xDeamidated [Q]; 1xPhospho [S3]	1	33	48	2093,79
[K].FQSEEQQQMEDELQDK.[I]	3xDeamidated [Q2; Q]; 1xPhospho [S3]	1	33	48	2094,77
[K].FQSEEQQQMEDELQDK.[I]	1xPhospho [S3]; 1xOxidation [M9]	1	33	48	2107,82
[K].FQSEEQQQMEDELQDK.[I]	1xDeamidated [Q]; 1xPhospho [S3]; 1xOxidation [M9]	1	33	48	2108,80
[KR].DMPIQAFLLYQEPVLGPVR.[G]		17	184	202	2186,17
[KR].DMPIQAFLLYQEPVLGPVR.[G]	2xDeamidated [Q5; Q11]	17	184	202	2188,14
[KR].DMPIQAFLLYQEPVLGPVR.[G]	1xOxidation [M2]	17	184	202	2202,16
[K-].IHPFAQTQSLVYPFPGPIPK.[S]		3	49	68	2237,21
[K].NHPFAQTQSLVYPFPGPIPK.[S]		1	49	68	2238,17
[K].IEKFQSEEQQQMEDELQDK.[I]	1xPhospho [S6]; 1xOxidation [M12]	1	30	48	2478,04
[K].SLPQNIPPLTQTPVVVPPFLQPEIMGVSK.[V]		8	69	97	3126,73
[K].SLPQNIPPLTQTPVVVPPFLQPEIMGVSK.[V]	1xOxidation [M25]	8	69	97	3142,72
[R].ELEELNVPGEIVESLSSSEESITHINK.[K]	3xPhospho [S17; S18; S21]	1	2	28	3222,38
[R].ELEELNVPGEIVESLSSSEESITHINK.[K]	4xPhospho [S14; S16; S17; S21]	1	2	28	3302,34
[-].RELEELNVPGEIVESLSSSEESITHINK.[K]	3xPhospho [S17; S18; S19]	2	1	28	3378,48
[-].RELEELNVPGEIVESLSSSEESITHINK.[K]	4xPhospho [S15; S17; S18; S19]	2	1	28	3458,45
**Alpha s2-casein OS=Bubalus bubalis**					
[K].ALNEINQFYQK.[F]		8	81	91	1367,70
[K].KTVDMESTEVITK.[K]	1xPhospho [S7]; 1xOxidation [M5]	5	137	149	1576,72
**Kappa-casein OS=Bubalus bubalis**					
[R].SPAQILQWQVLPNTVPAK.[S]		5	69	86	1990,11
**Alpha-lactalbumin protein variant D OS=Bos taurus**					
[K].VGINYWLAHK.[A]		7	99	108	1200,65

Sample IM3

Description	Modifications	# PSMs					Theor. MH+ [Da]
**Alpha-S1-casein (Fragment) OS=Bubalus bubalis**							
[R].YLGYLEQLLR.[L]		10	106	91	100	115	1267,70
[R].FFVAPFPEVFGK.[E]		9	38	23	34	49	1384,73
[K].HQGLPQGVLNENLLR.[F]		5	23	8	22	37	1687,92
[K].HQGLPQGVLNENLLR.[F]	1xDeamidated [Q/N]	5	23	8	22	37	1688,91
[K].EPMIGVNQELAYFYPQLFR.[Q]		5	148	133	151	166	2315,15
[K].EPMIGVNQELAYFYPQLFR.[Q]	1xOxidation [M3]	5	148	133	151	166	2331,15
[K].EPMIGVNQELAYFYPQLFR.[Q]	1xDeamidated [Q16]; 1xOxidation [M3]	5	148	133	151	166	2332,13
[K].YNVPQLEIVPNLAEEQLHSMK.[E]	1xOxidation [M20]	5	119	104	124	139	2468,25
[K].VNELSTDIGSESTEDQAMEDIK.[Q]	1xPhospho [S10]	4	52	37	58	73	2491,04
[K].VNELSTDIGSESTEDQAMEDIK.[Q]	2xPhospho [S/T]	4	52	37	58	73	2571,01
[K].KYNVPQLEIVPNLAEEQLHSMK.[E]		5	118	103	124	139	2580,35
[K].KYNVPQLEIVPNLAEEQLHSMK.[E]	1xDeamidated [N/Q]	5	118	103	124	139	2581,33
[K].VNELSTDIGSESTEDQAMEDIK.[Q]	2xPhospho [S10; T13]; 1xOxidation [M18]	4	52	37	58	73	2587,00
[K].KYNVPQLEIVPNLAEEQLHSMK.[E]	1xOxidation [M21]	5	118	103	124	139	2596,34
[K].VNELSTDIGSESTEDQAMEDIK.[Q]	3xPhospho [T/S]	4	52	37	58	73	2650,98
**Alpha S1 casein OS=Bos taurus**							
[K].HQGLPQEVLNENLLR.[F]		5	23	8	22	37	1759,94
**Beta-casein OS=Bubalus bubalis**							
[K].FQSEEQQQMEDELQDK.[I]		1	33	33	48	48	2011,85
[K].FQSEEQQQMEDELQDK.[I]	1xDeamidated [Q2]	1	33	33	48	48	2012,84
[K].FQSEEQQQMEDELQDK.[I]	1xOxidation [M9]	1	33	33	48	48	2027,85
[K].FQSEEQQQMEDELQDK.[I]	1xPhospho [S3]	1	33	33	48	48	2091,82
[K].FQSEEQQQMEDELQDK.[I]	1xDeamidated [Q]; 1xPhospho [S3]	1	33	33	48	48	2092,81
[K].FQSEEQQQMEDELQDK.[I]	2xDeamidated [Q]; 1xPhospho [S3]	1	33	33	48	48	2093,79
[K].FQSEEQQQMEDELQDK.[I]	3xDeamidated [Q2; Q]; 1xPhospho [S3]	1	33	33	48	48	2094,77
[K].FQSEEQQQMEDELQDK.[I]	1xPhospho [S3]; 1xOxidation [M9]	1	33	33	48	48	2107,82
[K].FQSEEQQQMEDELQDK.[I]	1xDeamidated [Q2]; 1xPhospho [S3]; 1xOxidation [M9]	1	33	33	48	48	2108,80
[K].FQSEEQQQMEDELQDK.[I]	2xDeamidated [Q]; 1xPhospho [S3]; 1xOxidation [M9]	1	33	33	48	48	2109,78
[KR].DMPIQAFLLYQEPVLGPVR.[G]		17	184	184	202	202	2186,17
[KR].DMPIQAFLLYQEPVLGPVR.[G]	2xDeamidated [Q5; Q11]	17	184	184	202	202	2188,14
[KR].DMPIQAFLLYQEPVLGPVR.[G]	1xDeamidated [Q]; 1xOxidation [M2]	17	184	184	202	202	2203,15
[K-].IHPFAQTQSLVYPFPGPIPK.[S]		3	49	49	68	68	2237,21
[K].NHPFAQTQSLVYPFPGPIPK.[S]		1	7	49	68	26	2238,17
[K-].IHPFAQTQSLVYPFPGPIPK.[S]	1xDeamidated [Q]	3	49	49	68	68	2238,20
[K].NHPFAQTQSLVYPFPGPIPK.[S]	1xDeamidated [Q8]	1	7	49	68	26	2239,15
[K].IEKFQSEEQQQMEDELQDK.[I]	1xPhospho [S6]; 1xOxidation [M12]	1	30	30	48	48	2478,04
[K].SLPQNIPPLTQTPVVVPPFLQPEIMGVSK.[V]		8	69	69	97	97	3126,73
[K].SLPQNIPPLTQTPVVVPPFLQPEIMGVSK.[V]	1xDeamidated [Q/N]	8	69	69	97	97	3127,71
[K].SLPQNIPPLTQTPVVVPPFLQPEIMGVSK.[V]	1xOxidation [M25]	8	69	69	97	97	3142,72
[R].ELEELNVPGEIVESLSSSEESITHINK.[K]	4xPhospho [S]	1	2	2	28	28	3302,34
[-].RELEELNVPGEIVESLSSSEESITHINK.[K]	4xPhospho [S15; S17; S18; S19]	2	1	1	28	28	3458,45
**AS2-casein (Fragment) OS=Bubalus bubalis**							
[K].ALNEINQFYQK.[F]		8	96	81	91	106	1367,70
[K].ALNEINQFYQK.[F]	1xDeamidated [N/Q]	8	96	81	91	106	1368,68
[K].FPQYLQYLYQGPIVLNPWDQVK.[R]		7	107	92	113	128	2709,41
[K].TVDMESTEVITK.[K]		5	141	138	148	152	1352,66
**Kappa-casein OS=Bubalus bubalis**							
[R].SPAQILQWQVLPNTVPAK.[S]		5	69	69	86	86	1990,11
[R].SPAQILQWQVLPNTVPAK.[S]	1xDeamidated [N13]	5	69	69	86	86	1991,10
[R].SPAQILQWQVLPNTVPAK.[S]	2xDeamidated [Q9; N13]	5	69	69	86	86	1992,08
[R].YPSYGLNYYQQKPVALINNQFLPYPYYAKPAAVR.[S]		20	35	35	68	68	4010,06
**Alpha-lactalbumin protein variant D OS=Bos taurus**							
[K].VGINYWLAHK.[A]		7	118	99	108	127	1200,65
**Beta-lactoglobulin variant D (Fragment) OS=Bos taurus**							
[R].VVYVEQLKPTPEGDLEILLQK.[W]		2	27	41	60	46	2312,27

IM4

Description	Modifications	# PSMs	Start	End	Theor. MH+ [Da]
**Alpha-S1-casein (Fragment) OS=Bubalus bubalis**					
[R].YLGYLEQLLR.[L]		10	91	100	1267,70
[R].FFVAPFPEVFGK.[E]		9	23	34	1384,73
[K].HQGLPQGVLNENLLR.[F]		5	8	22	1687,92
[K].HQGLPQGVLNENLLR.[F]	1xDeamidated [Q6]	5	8	22	1688,91
[K].HQGLPQGVLNENLLR.[F]	2xDeamidated [Q/N]	5	8	22	1689,89
[K].VNELSTDIGSESTEDQAMEDIK.[Q]	1xPhospho [S10]	4	37	58	2491,04
[K].VNELSTDIGSESTEDQAMEDIK.[Q]	2xPhospho [S/T]	4	37	58	2571,01
**Beta-casein OS=Bubalus bubalis**					
[K].FQSEEQQQMEDELQDK.[I]	1xOxidation [M9]	1	33	48	2027,85
[K].FQSEEQQQMEDELQDK.[I]	1xPhospho [S3]	1	33	48	2091,82
[K].FQSEEQQQMEDELQDK.[I]	1xDeamidated [Q]; 1xPhospho [S3]	1	33	48	2092,81
[K].FQSEEQQQMEDELQDK.[I]	2xDeamidated [Q]; 1xPhospho [S3]	1	33	48	2093,79
[K].FQSEEQQQMEDELQDK.[I]	3xDeamidated [Q]; 1xPhospho [S3]	1	33	48	2094,77
[K].FQSEEQQQMEDELQDK.[I]	1xPhospho [S3]; 1xOxidation [M9]	1	33	48	2107,82
[K].FQSEEQQQMEDELQDK.[I]	1xDeamidated [Q]; 1xPhospho [S3]; 1xOxidation [M9]	1	33	48	2108,80
[K-].IHPFAQTQSLVYPFPGPIPK.[S]		3	49	68	2237,21
[K].IEKFQSEEQQQMEDELQDK.[I]	1xPhospho [S6]	1	30	48	2462,04
[K].IEKFQSEEQQQMEDELQDK.[I]	1xDeamidated [Q]; 1xPhospho [S6]	1	30	48	2463,03
[K].SLPQNIPPLTQTPVVVPPFLQPEIMGVSK.[V]		8	69	97	3126,73
[-].RELEELNVPGEIVESLSSSEESITHINKK.[I]	2xPhospho [S17; S18]	1	1	29	3426,61
[-].RELEELNVPGEIVESLSSSEESITHINK.[K]	4xPhospho [S15; S17; S18; S19]	2	1	28	3458,45
[-].RELEELNVPGEIVESLSSSEESITHINK.[K]	1xDeamidated [N27]; 4xPhospho [S15; S17; S18; S19]	2	1	28	3459,43

IM5

Description	Modifications	# PSMs	Start	End	Theor. MH+ [Da]
**Alpha-S1-casein (Fragment) OS=Bubalus bubalis**					
[R].YLGYLEQLLR.[L]		10	91	100	1267,70
[R].FFVAPFPEVFGK.[E]		9	23	34	1384,73
[K].HQGLPQGVLNENLLR.[F]		5	8	22	1687,92
[K].HQGLPQGVLNENLLR.[F]	1xDeamidated [N/Q]	5	8	22	1688,91
[K].HQGLPQGVLNENLLR.[F]	3xDeamidated [Q2; Q6; N]	5	8	22	1690,88
[K].EPMIGVNQELAYFYPQLFR.[Q]		5	133	151	2315,15
[K].EPMIGVNQELAYFYPQLFR.[Q]	1xDeamidated [Q/N]	5	133	151	2316,14
[K].EPMIGVNQELAYFYPQLFR.[Q]	2xDeamidated [N/Q]	5	133	151	2317,12
[K].EPMIGVNQELAYFYPQLFR.[Q]	1xOxidation [M3]	5	133	151	2331,15
[K].YNVPQLEIVPNLAEEQLHSMK.[E]		5	104	124	2452,25
[K].YNVPQLEIVPNLAEEQLHSMK.[E]	1xOxidation [M20]	5	104	124	2468,25
[K].VNELSTDIGSESTEDQAMEDIK.[Q]	1xPhospho [S10]	4	37	58	2491,04
[K].VNELSTDIGSESTEDQAMEDIK.[Q]	2xPhospho [S/T]	4	37	58	2571,01
[K].KYNVPQLEIVPNLAEEQLHSMK.[E]		5	103	124	2580,35
[K].KYNVPQLEIVPNLAEEQLHSMK.[E]	1xDeamidated [N12]	5	103	124	2581,33
[K].KYNVPQLEIVPNLAEEQLHSMK.[E]	1xOxidation [M21]	5	103	124	2596,34
**Alpha S1 casein OS=Bos taurus**					
[K].EPMIGVNQELAYFYPELFR.[Q]	1xDeamidated [Q/N]	6	133	151	2317,12
[R].FFVAPFPEVFGK.[E]		9	23	34	1384,73
[K].HQGLPQEVLNENLLR.[F]		5	8	22	1759,94
[R].YLGYLEQLLR.[L]		10	91	100	1267,70
**Beta-casein OS=Bubalus bubalis**					
[K].FQSEEQQQMEDELQDK.[I]		1	33	48	2011,85
[K].FQSEEQQQMEDELQDK.[I]	1xOxidation [M9]	1	33	48	2027,85
[K].FQSEEQQQMEDELQDK.[I]	1xPhospho [S3]	1	33	48	2091,82
[K].FQSEEQQQMEDELQDK.[I]	1xDeamidated [Q]; 1xPhospho [S3]	1	33	48	2092,81
[K].FQSEEQQQMEDELQDK.[I]	2xDeamidated [Q]; 1xPhospho [S3]	1	33	48	2093,79
[K].FQSEEQQQMEDELQDK.[I]	3xDeamidated [Q]; 1xPhospho [S3]	1	33	48	2094,77
[K].FQSEEQQQMEDELQDK.[I]	1xPhospho [S3]; 1xOxidation [M9]	1	33	48	2107,82
[K].FQSEEQQQMEDELQDK.[I]	1xDeamidated [Q]; 1xPhospho [S3]; 1xOxidation [M9]	1	33	48	2108,80
[KR].DMPIQAFLLYQEPVLGPVR.[G]		17	184	202	2186,17
[KR].DMPIQAFLLYQEPVLGPVR.[G]	1xDeamidated [Q11]	17	184	202	2187,15
[KR].DMPIQAFLLYQEPVLGPVR.[G]	2xDeamidated [Q5; Q11]; 1xOxidation [M2]	17	184	202	2204,13
[K-].IHPFAQTQSLVYPFPGPIPK.[S]		3	49	68	2237,21
[K-].IHPFAQTQSLVYPFPGPIPK.[S]	1xDeamidated [Q]	3	49	68	2238,20
[K].IEKFQSEEQQQMEDELQDK.[I]	1xPhospho [S6]	1	30	48	2462,04
[K].SLPQNIPPLTQTPVVVPPFLQPEIMGVSK.[V]		8	69	97	3126,73
[-].RELEELNVPGEIVESLSSSEESITHINK.[K]	4xPhospho [S15; S17; S18; S19]	2	1	28	3458,45
**Alpha s2-casein OS=Bubalus bubalis**					
[K].ALNEINQFYQK.[F]		8	81	91	1367,70
[K].ALNEINQFYQK.[F]	1xDeamidated [N6]	8	81	91	1368,68
[K].TVDMESTEVITK.[K]	1xPhospho [T7]	5	138	149	1432,63
[K].KTVDMESTEVITK.[K]	1xPhospho [S/T]	5	137	149	1560,72
[K].FPQYLQYLYQGPIVLNPWDQVK.[R]		7	92	113	2709,41
[K].FPQYLQYLYQGPIVLNPWDQVK.[R]	1xDeamidated [N16]	7	92	113	2710,39
**Kappa-casein OS=Bubalus bubalis**					
[R].HPHPHLSFMAIPPK.[K]		23	98	111	1608,85
[R].SPAQILQWQVLPNTVPAK.[S]		5	69	86	1990,11
**Alpha-lactalbumin protein variant D OS=Bos taurus**					
[K].VGINYWLAHK.[A]		7	99	108	1200,65
**Beta-lactoglobulin OS=Bubalus bubalis**					
[R].TPEVDDEALEK.[F]		7	125	135	1245,58

Sample IM6

Description	Modifications	# PSMs	Start	End	Theor. MH+ [Da]
**Alpha-S1-casein (Fragment) OS=Bubalus bubalis**					
[R].YLGYLEQLLR.[L]		10	91	100	1267,70
[R].YLGYLEQLLR.[L]	1xDeamidated [Q7]	10	91	100	1268,69
[R].FFVAPFPEVFGK.[E]		9	23	34	1384,73
[K].HQGLPQGVLNENLLR.[F]		5	8	22	1687,92
[K].HQGLPQGVLNENLLR.[F]	1xDeamidated [Q/N]	5	8	22	1688,91
[K].HQGLPQGVLNENLLR.[F]	4xDeamidated [Q2; Q6; N10; N12]	5	8	22	1691,86
[K].EPMIGVNQELAYFYPQLFR.[Q]		5	133	151	2315,15
[K].EPMIGVNQELAYFYPQLFR.[Q]	1xDeamidated [Q16]	5	133	151	2316,14
[K].EPMIGVNQELAYFYPQLFR.[Q]	1xOxidation [M3]	5	133	151	2331,15
[K].YNVPQLEIVPNLAEEQLHSMK.[E]		5	104	124	2452,25
[K].YNVPQLEIVPNLAEEQLHSMK.[E]	1xDeamidated [Q/N]	5	104	124	2453,24
[K].YNVPQLEIVPNLAEEQLHSMK.[E]	1xOxidation [M20]	5	104	124	2468,25
[K].VNELSTDIGSESTEDQAMEDIK.[Q]	1xPhospho [S10]	4	37	58	2491,04
[K].VNELSTDIGSESTEDQAMEDIK.[Q]	1xDeamidated [Q16]; 1xPhospho [S10]	4	37	58	2492,03
[K].VNELSTDIGSESTEDQAMEDIK.[Q]	2xPhospho [T/S]	4	37	58	2571,01
[K].KYNVPQLEIVPNLAEEQLHSMK.[E]		5	103	124	2580,35
[K].KYNVPQLEIVPNLAEEQLHSMK.[E]	1xDeamidated [Q/N]	5	103	124	2581,33
[K].VNELSTDIGSESTEDQAMEDIK.[Q]	2xPhospho [S10; T13]; 1xOxidation [M18]	4	37	58	2587,00
[K].KYNVPQLEIVPNLAEEQLHSMK.[E]	1xOxidation [M21]	5	103	124	2596,34
[K].VNELSTDIGSESTEDQAMEDIK.[Q]	3xPhospho [S10; S/T]	4	37	58	2650,98
**Beta-casein OS=Bubalus bubalis**					
[K].FQSEEQQQMEDELQDK.[I]		1	33	48	2011,85
[K].FQSEEQQQMEDELQDK.[I]	1xDeamidated [Q2]	1	33	48	2012,84
[K].FQSEEQQQMEDELQDK.[I]	1xPhospho [S3]	1	33	48	2091,82
[K].FQSEEQQQMEDELQDK.[I]	1xDeamidated [Q]; 1xPhospho [S3]	1	33	48	2092,81
[K].FQSEEQQQMEDELQDK.[I]	2xDeamidated [Q]; 1xPhospho [S3]	1	33	48	2093,79
[K].FQSEEQQQMEDELQDK.[I]	3xDeamidated [Q2; Q]; 1xPhospho [S3]	1	33	48	2094,77
[K].FQSEEQQQMEDELQDK.[I]	4xDeamidated [Q2; Q14; Q]; 1xPhospho [S3]	1	33	48	2095,76
[K].FQSEEQQQMEDELQDK.[I]	1xPhospho [S3]; 1xOxidation [M9]	1	33	48	2107,82
[K].FQSEEQQQMEDELQDK.[I]	1xDeamidated [Q]; 1xPhospho [S3]; 1xOxidation [M9]	1	33	48	2108,80
[K].FQSEEQQQMEDELQDK.[I]	3xDeamidated [Q2; Q]; 1xPhospho [S3]; 1xOxidation [M9]	1	33	48	2110,77
[KR].DMPIQAFLLYQEPVLGPVR.[G]		17	184	202	2186,17
[KR].DMPIQAFLLYQEPVLGPVR.[G]	1xDeamidated [Q]	17	184	202	2187,15
[KR].DMPIQAFLLYQEPVLGPVR.[G]	1xOxidation [M2]	17	184	202	2202,16
[K-].IHPFAQTQSLVYPFPGPIPK.[S]		3	49	68	2237,21
[K-].IHPFAQTQSLVYPFPGPIPK.[S]	1xDeamidated [Q]	3	49	68	2238,20
[K].IEKFQSEEQQQMEDELQDK.[I]	1xPhospho [S6]	1	30	48	2462,04
[K].IEKFQSEEQQQMEDELQDK.[I]	1xDeamidated [Q]; 1xPhospho [S6]	1	30	48	2463,03
[K].IEKFQSEEQQQMEDELQDK.[I]	2xDeamidated [Q5; Q9]; 1xPhospho [S6]	1	30	48	2464,01
[K].IEKFQSEEQQQMEDELQDK.[I]	1xPhospho [S6]; 1xOxidation [M12]	1	30	48	2478,04
[K].SLPQNIPPLTQTPVVVPPFLQPEIMGVSK.[V]		8	69	97	3126,73
[-].RELEELNVPGEIVESLSSSEESITHINKK.[I]	2xPhospho [S18; S]	1	1	29	3426,61
[-].RELEELNVPGEIVESLSSSEESITHINK.[K]	4xPhospho [S15; S/T]	2	1	28	3458,45
[-].RELEELNVPGEIVESLSSSEESITHINK.[K]	1xDeamidated [N]; 4xPhospho [S15; S/T]	2	1	28	3459,43
**Alpha s2-casein OS=Bubalus bubalis**					
[R].EQLSTSEENSK.[K]	1xPhospho [S6]	8	126	136	1331,54
[R].EQLSTSEENSK.[K]	1xDeamidated [Q2]; 1xPhospho [S6]	8	126	136	1332,52
[K].TVDMESTEVITK.[K]	1xPhospho [S/T]	5	138	149	1432,63
[K].TVDMESTEVITK.[K]	1xPhospho [S6]; 1xOxidation [M4]	5	138	149	1448,62
[K].TVDMESTEVITKK.[T]	1xPhospho [S/T]	5	138	150	1560,72
[K].KTVDMESTEVITK.[K]	1xPhospho [S7]; 1xOxidation [M5]	5	137	149	1576,72
[K].FPQYLQYLYQGPIVLNPWDQVK.[R]		7	92	113	2709,41
[K].FPQYLQYLYQGPIVLNPWDQVK.[R]	1xDeamidated [N/Q]	7	92	113	2710,39
**Kappa-casein OS=Bubalus bubalis**					
[R].YPSYGLNYYQQKPVALINNQFLPYPYYAKPAAVR.[S]		20	35	68	4010,06
[R].SPAQILQWQVLPNTVPAK.[S]		5	69	86	1990,11
**Alpha-lactalbumin protein variant D OS=Bos taurus**					
[K].VGINYWLAHK.[A]		1	99	108	1200,65
**Beta-lactoglobulin variant D (Fragment) OS=Bos taurus**					
[R].VYVEQLKPTPEGDLEILLQK.[W]		1	41	60	2312,27
[R].VYVEQLKPTPEGDLEILLQK.[W]	1xDeamidated [Q5]	1	41	60	2313,26

Sample IM7

Description	Modifications	# PSMs	Start	End	Theor. MH+ [Da]
**Alpha-S1-casein (Fragment) OS=Bubalus bubalis**					
[R].YLGYLEQLLR.[L]		10	91	100	1267,70
[R].FFVAPFPEVFGK.[E]		9	23	34	1384,73
[R].FFVAPFPEMFGK.[D]		1	23	34	1416,70
[K].HQGLPQGVLNENLLR.[F]		5	8	22	1687,92
[K].HQGLPQGVLNENLLR.[F]	1xDeamidated [Q/N]	5	8	22	1688,91
[K].EPMIGVNQELAYFYPQLFR.[Q]		5	133	151	2315,15
[K].EPMIGVNQELAYFYPQLFR.[Q]	1xDeamidated [Q/N]	5	133	151	2316,14
[K].EPMIGVNQELAYFYPQLFR.[Q]	1xOxidation [M3]	5	133	151	2331,15
[K].YNVPQLEIVPNLAEEQLHSMK.[E]		5	104	124	2452,25
[K].YNVPQLEIVPNLAEEQLHSMK.[E]	1xOxidation [M20]	5	104	124	2468,25
[K].VNELSTDIGSESTEDQAMEDIK.[Q]	1xPhospho [S10]	4	37	58	2491,04
[K].VNELSTDIGSESTEDQAMEDIK.[Q]	2xPhospho [S/T]	4	37	58	2571,01
[K].KYNVPQLEIVPNLAEEQLHSMK.[E]		5	103	124	2580,35
[K].VNELSTDIGSESTEDQAMEDIK.[Q]	2xPhospho [S/T]; 1xOxidation [M18]	4	37	58	2587,00
[K].KYNVPQLEIVPNLAEEQLHSMK.[E]	1xOxidation [M21]	5	103	124	2596,34
[K].KYNVPQLEIVPNLAEEQLHSMK.[E]	1xDeamidated [N3]; 1xOxidation [M21]	5	103	124	2597,33
**Beta-casein OS=Bubalus bubalis**					
[K].FQSEEQQQMEDELQDK.[I]		1	33	48	2011,85
[K].FQSEEQQQMEDELQDK.[I]	1xOxidation [M9]	1	33	48	2027,85
[K].FQSEEQQQMEDELQDK.[I]	1xPhospho [S3]	1	33	48	2091,82
[K].FQSEEQQQMEDELQDK.[I]	1xDeamidated [Q]; 1xPhospho [S3]	1	33	48	2092,81
[K].FQSEEQQQMEDELQDK.[I]	2xDeamidated [Q]; 1xPhospho [S3]	1	33	48	2093,79
[K].FQSEEQQQMEDELQDK.[I]	1xPhospho [S3]; 1xOxidation [M9]	1	33	48	2107,82
[K].FQSEEQQQMEDELQDK.[I]	1xDeamidated [Q]; 1xPhospho [S3]; 1xOxidation [M9]	1	33	48	2108,80
[KR].DMPIQAFLLYQEPVLGPVR.[G]		17	184	202	2186,17
[KR].DMPIQAFLLYQEPVLGPVR.[G]	1xDeamidated [Q]	17	184	202	2187,15
[KR].DMPIQAFLLYQEPVLGPVR.[G]	1xDeamidated [Q]; 1xOxidation [M2]	17	184	202	2203,15
[K-].IHPFAQTQSLVYPFPGPIPK.[S]		3	49	68	2237,21
[K-].IHPFAQTQSLVYPFPGPIPK.[S]	1xDeamidated [Q]	3	49	68	2238,20
[K-].IHPFAQTQSLVYPFPGPIPK.[S]	2xDeamidated [Q6; Q8]	3	49	68	2239,18
[K].IEKFQSEEQQQMEDELQDK.[I]	1xPhospho [S6]	1	30	48	2462,04
[K].IEKFQSEEQQQMEDELQDK.[I]	1xDeamidated [Q9]; 1xPhospho [S6]	1	30	48	2463,03
[K].SLPQNIPPLTQTPVVVPPFLQPEIMGVSK.[V]		8	69	97	3126,73
[K].SLPQNIPPLTQTPVVVPPFLQPEIMGVSK.[V]	1xDeamidated [Q/N]	8	69	97	3127,71
[K].SLPQNIPPLTQTPVVVPPFLQPEIMGVSK.[V]	1xOxidation [M25]	8	69	97	3142,72
[-].RELEELNVPGEIVESLSSSEESITHINK.[K]	2xPhospho [S18; S19]	2	1	28	3298,51
[R].ELEELNVPGEIVESLSSSEESITHINK.[K]	4xPhospho [S/T]	1	2	28	3302,34
[-].RELEELNVPGEIVESLSSSEESITHINK.[K]	4xPhospho [S/T]	2	1	28	3458,45
[-].RELEELNVPGEIVESLSSSEESITHINK.[K]	1xDeamidated [N7]; 4xPhospho [S15; S17; S18; S19]	2	1	28	3459,43
[-].RELEELNVPGEIVESLSSSEESITHINKK.[I]	1xDeamidated [N27]; 3xPhospho [S17; S18; S19]	1	1	29	3507,56
**Alpha-S2-casein OS=Bubalus bubalis**					
[K].ALNEINQFYQK.[F]		8	81	91	1367,70
[K].ALNEINQFYQK.[F]	1xDeamidated [N3]	8	81	91	1368,68
[K].FPQYLQYLYQGPIVLNPWDQVK.[R]		7	92	113	2709,41
[K].ISQHYQK.[F]		9	167	173	903,47
[K].TVDMESTEVFTK.[K]	1xPhospho [S6]	3	138	149	1466,61
[K].TVDMESTEVITK.[K]	1xPhospho [S/T]	5	138	149	1432,63
**Kappa-casein OS=Bubalus bubalis**					
[R].SPAQILQWQVLPNTVPAK.[S]		5	69	86	1990,11
[R].YPSYGLNYYQQKPVALINNQFLPYPYYAKPAAVR.[S]		20	35	68	4010,06
**Alpha-lactalbumin protein variant D OS=Bos taurus**					
[K].VGINYWLAHK.[A]		7	99	108	1200,65

Sample IM8

Description	Modifications	# PSMs	start	end	Theor. MH+ [Da]
**Alpha-S1-casein (Fragment) OS=Bubalus bubalis**					
[R].YLGYLEQLLR.[L]		10	91	100	1267,70
[R].FFVAPFPEVFGK.[E]		9	23	34	1384,73
[K].HQGLPQGVLNENLLR.[F]		5	8	22	1687,92
[K].HQGLPQGVLNENLLR.[F]	1xDeamidated [Q6]	5	8	22	1688,91
[K].HQGLPQGVLNENLLR.[F]	4xDeamidated [Q2; Q6; N10; N12]	5	8	22	1691,86
[K].EPMIGVNQELAYFYPQLFR.[Q]		5	133	151	2315,15
[K].EPMIGVNQELAYFYPQLFR.[Q]	1xDeamidated [Q/N]	5	133	151	2316,14
[K].EPMIGVNQELAYFYPQLFR.[Q]	1xOxidation [M3]	5	133	151	2331,15
[K].YNVPQLEIVPNLAEEQLHSMK.[E]		5	104	124	2452,25
[K].YNVPQLEIVPNLAEEQLHSMK.[E]	1xDeamidated [N/Q]	5	104	124	2453,24
[K].YNVPQLEIVPNLAEEQLHSMK.[E]	1xOxidation [M20]	5	104	124	2468,25
[K].VNELSTDIGSESTEDQAMEDIK.[Q]	1xPhospho [S10]	4	37	58	2491,04
[K].VNELSTDIGSESTEDQAMEDIK.[Q]	2xPhospho [S/T]	4	37	58	2571,01
[K].KYNVPQLEIVPNLAEEQLHSMK.[E]		5	103	124	2580,35
[K].KYNVPQLEIVPNLAEEQLHSMK.[E]	1xDeamidated [N12]	5	103	124	2581,33
[K].VNELSTDIGSESTEDQAMEDIK.[Q]	2xPhospho [S/T]; 1xOxidation [M18]	4	37	58	2587,00
[K].KYNVPQLEIVPNLAEEQLHSMK.[E]	1xOxidation [M21]	5	103	124	2596,34
[K].KYNVPQLEIVPNLAEEQLHSMK.[E]	1xDeamidated [N/Q]; 1xOxidation [M21]	5	103	124	2597,33
[K].VNELSTDIGSESTEDQAMEDIK.[Q]	3xPhospho [S5; S10; S12]; 1xOxidation [M18]	4	37	58	2666,97
**AlphaS1-casein OS=Bos taurus**					
[K].VNELSKDIGSESTEDQAMEDIK.[Q]	2xPhospho [S10; T/S]	6	37	58	2598,06
[R].YLGYLEQLLR.[L]		10	91	100	1267,70
**Beta-casein OS=Bubalus bubalis**					
[K].FQSEEQQQMEDELQDK.[I]		1	33	48	2011,85
[K].FQSEEQQQMEDELQDK.[I]	1xOxidation [M9]	1	33	48	2027,85
[K].FQSEEQQQMEDELQDK.[I]	1xDeamidated [Q2]; 1xOxidation [M9]	1	33	48	2028,83
[K].FQSEEQQQMEDELQDK.[I]	1xPhospho [S3]	1	33	48	2091,82
[K].FQSEEQQQMEDELQDK.[I]	1xDeamidated [Q]; 1xPhospho [S3]	1	33	48	2092,81
[K].FQSEEQQQMEDELQDK.[I]	2xDeamidated [Q]; 1xPhospho [S3]	1	33	48	2093,79
[K].FQSEEQQQMEDELQDK.[I]	3xDeamidated [Q]; 1xPhospho [S3]	1	33	48	2094,77
[K].FQSEEQQQMEDELQDK.[I]	1xPhospho [S3]; 1xOxidation [M9]	1	33	48	2107,82
[K].FQSEEQQQMEDELQDK.[I]	1xDeamidated [Q]; 1xPhospho [S3]; 1xOxidation [M9]	1	33	48	2108,80
[KR].DMPIQAFLLYQEPVLGPVR.[G]		17	184	202	2186,17
[KR].DMPIQAFLLYQEPVLGPVR.[G]	1xDeamidated [Q11]	17	184	202	2187,15
[KR].DMPIQAFLLYQEPVLGPVR.[G]	1xDeamidated [Q]; 1xOxidation [M2]	17	184	202	2203,15
[K-].IHPFAQTQSLVYPFPGPIPK.[S]		3	49	68	2237,21
[K-].IHPFAQTQSLVYPFPGPIPK.[S]	1xDeamidated [Q]	3	49	68	2238,20
[K].IEKFQSEEQQQMEDELQDK.[I]	1xPhospho [S6]	1	30	48	2462,04
[K].IEKFQSEEQQQMEDELQDK.[I]	1xPhospho [S6]; 1xOxidation [M12]	1	30	48	2478,04
[K].SLPQNIPPLTQTPVVVPPFLQPEIMGVSK.[V]		8	69	97	3126,73
[K].SLPQNIPPLTQTPVVVPPFLQPEIMGVSK.[V]	1xDeamidated [Q/N]	8	69	97	3127,71
[K].SLPQNIPPLTQTPVVVPPFLQPEIMGVSK.[V]	1xOxidation [M25]	8	69	97	3142,72
[K].SLPQNIPPLTQTPVVVPPFLQPEIMGVSK.[V]	1xDeamidated [Q/N]; 1xOxidation [M25]	8	69	97	3143,71
[R].ELEELNVPGEIVESLSSSEESITHINK.[K]	4xPhospho [S14; S16; S17; S21]	1	2	28	3302,34
[-].RELEELNVPGEIVESLSSSEESITHINK.[K]	4xPhospho [S15; S/T]	2	1	28	3458,45
[-].RELEELNVPGEIVESLSSSEESITHINK.[K]	1xDeamidated [N]; 4xPhospho [S15; S17; S18; S19]	2	1	28	3459,43
**AlphaS2-casein (Fragment) OS=Bubalus bubalis**					
[K].ALNEINQFYQK.[F]	1xDeamidated [N/Q]	8	81	91	1368,68
[K].FPQYLQYLYQGPIVLNPWDQVK.[R]		7	92	113	2709,41
[K].FPQYLQYLYQGPIVLNPWDQVK.[R]	1xDeamidated [N/Q]	7	92	113	2710,39
[K].KTVDMESTEVITK.[K]	1xPhospho [S7]	5	137	149	1560,72
**Kappa-casein OS=Bubalus bubalis**					
[R].SPAQILQWQVLPNTVPAK.[S]		5	69	86	1990,11
[R].SPAQILQWQVLPNTVPAK.[S]	1xDeamidated [Q/N]	5	69	86	1991,10
[R].YPSYGLNYYQQKPVALINNQFLPYPYYAKPAAVR.[S]		20	35	68	4010,06
**Beta-lactoglobulin OS=Bubalus bubalis**					
[R].TPEVDDEALEK.[F]		7	125	135	1245,58

Sample IM9

Description	Modifications	# PSMs	Start	End	Theor. MH+ [Da]
**Alpha-S1-casein (Fragment) OS=Bubalus bubalis**					
[R].YLGYLEQLLR.[L]		10	91	100	1267,70
[R].FFVAPFPEVFGK.[E]		9	23	34	1384,73
[K].HQGLPQGVLNENLLR.[F]		5	8	22	1687,92
[K].HQGLPQGVLNENLLR.[F]	1xDeamidated [Q/N]	5	8	22	1688,91
[K].HQGLPQGVLNENLLR.[F]	2xDeamidated [Q6; N10]	5	8	22	1689,89
[K].EPMIGVNQELAYFYPQLFR.[Q]		5	133	151	2315,15
[K].YNVPQLEIVPNLAEEQLHSMK.[E]		5	104	124	2452,25
[K].YNVPQLEIVPNLAEEQLHSMK.[E]	1xDeamidated [Q/N]	5	104	124	2453,24
[K].YNVPQLEIVPNLAEEQLHSMK.[E]	1xOxidation [M20]	5	104	124	2468,25
[K].YNVPQLEIVPNLAEEQLHSMK.[E]	1xDeamidated [Q16]; 1xOxidation [M20]	5	104	124	2469,23
[K].VNELSTDIGSESTEDQAMEDIK.[Q]	1xPhospho [S10]	4	37	58	2491,04
[K].VNELSTDIGSESTEDQAMEDIK.[Q]	2xPhospho [T/S]	4	37	58	2571,01
[K].KYNVPQLEIVPNLAEEQLHSMK.[E]		5	103	124	2580,35
[K].VNELSTDIGSESTEDQAMEDIK.[Q]	2xPhospho [S10; T13]; 1xOxidation [M18]	4	37	58	2587,00
[K].KYNVPQLEIVPNLAEEQLHSMK.[E]	1xOxidation [M21]	5	103	124	2596,34
[K].KYNVPQLEIVPNLAEEQLHSMK.[E]	1xDeamidated [Q/N]; 1xOxidation [M21]	5	103	124	2597,33
**Alpha S1 casein OS=Bos taurus**					
[K].EPMIGVNQELAYFYPELFR.[Q]		6	133	151	2316,14
[R].FFVAPFPEVFGK.[E]		9	23	34	1384,73
[K].HQGLPQEVLNENLLR.[F]		5	8	22	1759,94
[K].HQGLPQEVLNENLLR.[F]	1xDeamidated [Q6]	5	8	22	1760,93
[R].YLGYLEQLLR.[L]		10	91	100	1267,70
**Beta-casein OS=Bubalus bubalis**					
[K].FQSEEQQQMEDELQDK.[I]		1	33	48	2011,85
[K].FQSEEQQQMEDELQDK.[I]	1xOxidation [M9]	1	33	48	2027,85
[K].FQSEEQQQMEDELQDK.[I]	1xPhospho [S3]	1	33	48	2091,82
[K].FQSEEQRQTEDELQDK.[I]	2xDeamidated [Q8; Q]; 1xPhospho [S3]	1	33	48	2091,84
[K].FQSEEQQQMEDELQDK.[I]	1xDeamidated [Q]; 1xPhospho [S3]	1	33	48	2092,81
[K].FQSEEQQQMEDELQDK.[I]	2xDeamidated [Q]; 1xPhospho [S3]	1	33	48	2093,79
[K].FQSEEQQQMEDELQDK.[I]	3xDeamidated [Q2; Q14; Q]; 1xPhospho [S3]	1	33	48	2094,77
[K].FQSEEQQQMEDELQDK.[I]	4xDeamidated [Q2; Q6; Q]; 1xPhospho [S3]	1	33	48	2095,76
[K].FQSEEQQQMEDELQDK.[I]	1xPhospho [S3]; 1xOxidation [M9]	1	33	48	2107,82
[K].FQSEEQQQMEDELQDK.[I]	1xDeamidated [Q2]; 1xPhospho [S3]; 1xOxidation [M9]	1	33	48	2108,80
[K].FQSEEQQQMEDELQDK.[I]	2xDeamidated [Q]; 1xPhospho [S3]; 1xOxidation [M9]	1	33	48	2109,78
[KR].DMPIQAFLLYQEPVLGPVR.[G]		17	184	202	2186,17
[KR].DMPIQAFLLYQEPVLGPVR.[G]	1xOxidation [M2]	17	184	202	2202,16
[K-].IHPFAQTQSLVYPFPGPIPK.[S]		3	49	68	2237,21
[K-].IHPFAQTQSLVYPFPGPIPK.[S]	1xDeamidated [Q]	3	49	68	2238,20
[K-].IHPFAQTQSLVYPFPGPIPK.[S]	2xDeamidated [Q6; Q8]	3	49	68	2239,18
[K].IEKFQSEEQQQMEDELQDK.[I]	1xPhospho [S6]	1	30	48	2462,04
[K].SLPQNIPPLTQTPVVVPPFLQPEIMGVSK.[V]		8	69	97	3126,73
[K].SLPQNIPPLTQTPVVVPPFLQPEIMGVSK.[V]	1xDeamidated [Q11]	8	69	97	3127,71
[-].RELEELNVPGEIVESLSSSEESITHINK.[K]	4xPhospho [S15; S17; S18; S19]	2	1	28	3458,45
[-].RELEELNVPGEIVESLSSSEESITHINK.[K]	1xDeamidated [N7]; 4xPhospho [S15; S17; S18; S19]	2	1	28	3459,43
**Alpha-S2-casein OS=Bubalus bubalis**					
[K].TVDMESTEVITK.[K]		5	138	149	1352,66
[K].ALNEINQFYQK.[F]		8	81	91	1367,70
[K].KTVDMESTEVITK.[K]	1xPhospho [S7]	5	137	149	1560,72
[K].KTVDMESTEVFTK.[K]	1xPhospho [S7]	3	137	149	1594,71
[K].HTMEHVSSSEESIISQETYK.[Q]	1xPhospho [S]	7	2	21	2402,02
[K].FPQYLQYLYQGPIVLNPWDQVK.[R]		7	92	113	2709,41
[K].FPQYLQYLYQGPIVLNPWDQVK.[R]	1xDeamidated [Q20]	7	92	113	2710,39
**Kappa-casein OS=Bubalus bubalis**					
[R].SPAQILQWQVLPNTVPAK.[S]		5	69	86	1990,11
[R].SPAQILQWQVLPNTVPAK.[S]	1xDeamidated [N13]	5	69	86	1991,10
[R].YPSYGLNYYQQKPVALINNQFLPYPYYAKPAAVR.[S]		20	35	68	4010,06
[R].YPSYGLNYYQQKPVALINNQFLPYPYYAKPAAVR.[S]	1xDeamidated [N7]	20	35	68	4011,05
**Alpha-lactalbumin protein variant D OS=Bos taurus**					
[K].VGINYWLAHK.[A]		7	99	108	1200,65
**Beta-lactoglobulin OS=Bubalus bubalis**					
[R].TPEVDDEALEK.[F]		7	125	135	1245,58
[K].VLVLDTDYK.[K]		13	92	100	1065,58
[R].VYVEELKPTPEGDLEILLQK.[W]		8	41	60	2313,26

Sample IM10

Description	Modifications	# PSMs	Start	End	Theor. MH+ [Da]
**Alpha-S1-casein (Fragment) OS=Bubalus bubalis**					
[R].YLGYLEQLLR.[L]		10	91	100	1267,70
[R].FFVAPFPEVFGK.[E]		9	23	34	1384,73
[K].HQGLPQGVLNENLLR.[F]		5	8	22	1687,92
[K].HQGLPQGVLNENLLR.[F]	1xDeamidated [Q6]	5	8	22	1688,91
[K].HQGLPQGVLNENLLR.[F]	3xDeamidated [Q6; N/Q]	5	8	22	1690,88
[K].EPMIGVNQELAYFYPQLFR.[Q]		5	133	151	2315,15
[K].EPMIGVNQELAYFYPQLFR.[Q]	1xDeamidated [Q/N]	5	133	151	2316,14
[K].YNVPQLEIVPNLAEEQLHSMK.[E]		5	104	124	2452,25
[K].YNVPQLEIVPNLAEEQLHSMK.[E]	1xOxidation [M20]	5	104	124	2468,25
[K].VNELSTDIGSESTEDQAMEDIK.[Q]	1xPhospho [S10]	4	37	58	2491,04
[K].VNELSTDIGSESTEDQAMEDIK.[Q]	2xPhospho [S/T]	4	37	58	2571,01
[K].KYNVPQLEIVPNLAEEQLHSMK.[E]		5	103	124	2580,35
[K].VNELSTDIGSESTEDQAMEDIK.[Q]	2xPhospho [S10; T13]; 1xOxidation [M18]	4	37	58	2587,00
[K].KYNVPQLEIVPNLAEEQLHSMK.[E]	1xOxidation [M21]	5	103	124	2596,34
[K].EKVNELSTDIGSESTEDQAMEDIK.[Q]	1xPhospho [S12]	4	35	58	2748,18
**Alpha S1 casein OS=Bos taurus**					
[K].EPMIGVNQELAYFYPELFR.[Q]		6	133	151	2316,14
[R].FFVAPFPEVFGK.[E]		9	23	34	1384,73
[K].HQGLPQEVLNENLLR.[F]		5	8	22	1759,94
[K].VPQLEIVPNSAEER.[L]	1xPhospho [S10]	4	106	119	1660,79
[R].YLGYLEQLLR.[L]		10	91	100	1267,70
**Beta-casein OS=Bubalus bubalis**					
[K].FQSEEQQQMEDELQDK.[I]		1	33	48	2011,85
[K].FQSEEQQQMEDELQDK.[I]	1xOxidation [M9]	1	33	48	2027,85
[K].FQSEEQQQMEDELQDK.[I]	1xPhospho [S3]	1	33	48	2091,82
[K].FQSEEQQQMEDELQDK.[I]	1xDeamidated [Q]; 1xPhospho [S3]	1	33	48	2092,81
[K].FQSEEQQQMEDELQDK.[I]	2xDeamidated [Q2; Q]; 1xPhospho [S3]	1	33	48	2093,79
[K].FQSEEQQQMEDELQDK.[I]	3xDeamidated [Q2; Q]; 1xPhospho [S3]	1	33	48	2094,77
[K].FQSEEQQQMEDELQDK.[I]	4xDeamidated [Q2; Q]; 1xPhospho [S3]	1	33	48	2095,76
[K].FQSEEQQQMEDELQDK.[I]	1xPhospho [S3]; 1xOxidation [M9]	1	33	48	2107,82
[K].FQSEEQQQMEDELQDK.[I]	1xDeamidated [Q]; 1xPhospho [S3]; 1xOxidation [M9]	1	33	48	2108,80
[KR].DMPIQAFLLYQEPVLGPVR.[G]		17	184	202	2186,17
[KR].DMPIQAFLLYQEPVLGPVR.[G]	1xDeamidated [Q11]	17	184	202	2187,15
[K-].IHPFAQTQSLVYPFPGPIPK.[S]		3	49	68	2237,21
[K-].IHPFAQTQSLVYPFPGPIPK.[S]	1xDeamidated [Q]	3	49	68	2238,20
[K-].IHPFAQTQSLVYPFPGPIPK.[S]	2xDeamidated [Q6; Q8]	3	49	68	2239,18
[K].SLPQNIPPLTQTPVVVPPFLQPEIMGVSK.[V]		8	69	97	3126,73
[K].SLPQNIPPLTQTPVVVPPFLQPEIMGVSK.[V]	1xOxidation [M25]	8	69	97	3142,72
[-].RELEELNVPGEIVESLSSSEESITHINK.[K]	1xPhospho [S18]	2	1	28	3218,55
[-].RELEELNVPGEIVESLSSSEESITHINK.[K]	4xPhospho [S15; S17; S18; S19]	2	1	28	3458,45
**Alpha s2-casein OS=Bubalus bubalis**					
[K].TVDMESTEVITK.[K]		5	138	149	1352,66
[K].ALNEINQFYQK.[F]		8	81	91	1367,70
[K].TVDMESTEVFTK.[K]	1xOxidation [M4]	3	138	149	1402,64
[K].TVDMESTEVITK.[K]	1xPhospho [S/T]	5	138	149	1432,63
[K].TVDMESTEVFTK.[K]	1xPhospho [S6]	3	138	149	1466,61
[K].KTVDMESTEVFTK.[K]	1xPhospho [S7]	3	137	149	1594,71
[K].HTMEHVSSSEESIISQETYK.[Q]		7	2	21	2322,06
[K].FPQYLQYLYQGPIVLNPWDQVK.[R]		7	92	113	2709,41
**Kappa-casein OS=Bubalus bubalis**					
[R].SPAQILQWQVLPNTVPAK.[S]		5	69	86	1990,11
[-].DAYPSGAWYYVPLGTQYPDAPLFSDIPNPIGSENSGK.[T]		1	1	37	3986,88
**Beta-lactoglobulin OS=Bubalus bubalis**					
[R].TPEVDDEALEK.[F]		7	125	135	1245,58

Sample IM10

Description	Modifications	# PSMs	Start	End	Theor. MH+ [Da]
**Alpha-S1-casein (Fragment) OS=Bubalus bubalis**					
[R].YLGYLEQLLR.[L]		10	91	100	1267,70
[K].HQGLPQGVLNENLLR.[F]		5	8	22	1687,92
[K].HQGLPQGVLNENLLR.[F]	1xDeamidated [N/Q]	5	8	22	1688,91
[K].HQGLPQGVLNENLLR.[F]	2xDeamidated [Q/N]	5	8	22	1689,89
[K].EPMIGVNQELAYFYPQLFR.[Q]		5	133	151	2315,15
[K].EPMIGVNQELAYFYPQLFR.[Q]	1xDeamidated [Q16]	5	133	151	2316,14
[K].VNELSTDIGSESTEDQAMEDIK.[Q]		4	37	58	2411,08
[K].YNVPQLEIVPNLAEEQLHSMK.[E]		5	104	124	2452,25
[K].YNVPQLEIVPNLAEEQLHSMK.[E]	1xDeamidated [Q/N]	5	104	124	2453,24
[K].YNVPQLEIVPNLAEEQLHSMK.[E]	1xOxidation [M20]	5	104	124	2468,25
[K].VNELSTDIGSESTEDQAMEDIK.[Q]	1xPhospho [S10]	4	37	58	2491,04
[K].VNELSTDIGSESTEDQAMEDIK.[Q]	2xPhospho [S/T]	4	37	58	2571,01
[K].KYNVPQLEIVPNLAEEQLHSMK.[E]		5	103	124	2580,35
[K].VNELSTDIGSESTEDQAMEDIK.[Q]	2xPhospho [S10; T13]; 1xOxidation [M18]	4	37	58	2587,00
[K].KYNVPQLEIVPNLAEEQLHSMK.[E]	1xOxidation [M21]	5	103	124	2596,34
**Alpha S1 casein OS=Bos taurus**					
[K].EPMIGVNQELAYFYPELFR.[Q]		6	133	151	2316,14
[K].HQGLPQEVLNENLLR.[F]	1xDeamidated [Q]	5	8	22	1760,93
[K].VPQLEIVPNSAEER.[L]	1xPhospho [S10]	4	106	119	1660,79
[R].YLGYLEQLLR.[L]		10	91	100	1267,70
**Beta-casein OS=Bubalus bubalis**					
[K].FQSEEQQQMEDELQDK.[I]		1	33	48	2011,85
[K].FQSEEQQQMEDELQDK.[I]	1xOxidation [M9]	1	33	48	2027,85
[K].FQSEEQQQMEDELQDK.[I]	1xPhospho [S3]	1	33	48	2091,82
[K].FQSEEQQQMEDELQDK.[I]	1xDeamidated [Q]; 1xPhospho [S3]	1	33	48	2092,81
[K].FQSEEQQQMEDELQDK.[I]	2xDeamidated [Q2; Q]; 1xPhospho [S3]	1	33	48	2093,79
[K].FQSEEQQQMEDELQDK.[I]	2xDeamidated [Q]; 1xPhospho [S3]	1	33	48	2093,79
[K].FQSEEQQQMEDELQDK.[I]	3xDeamidated [Q]; 1xPhospho [S3]	1	33	48	2094,77
[K].FQSEEQQQMEDELQDK.[I]	5xDeamidated [Q2; Q6; Q7; Q8; Q14]; 1xPhospho [S3]	1	33	48	2096,74
[K].FQSEEQQQMEDELQDK.[I]	1xPhospho [S3]; 1xOxidation [M9]	1	33	48	2107,82
[K].FQSEEQQQMEDELQDK.[I]	1xDeamidated [Q]; 1xPhospho [S3]; 1xOxidation [M9]	1	33	48	2108,80
[KR].DMPIQAFLLYQEPVLGPVR.[G]		17	184	202	2186,17
[KR].DMPIQAFLLYQEPVLGPVR.[G]	1xDeamidated [Q5]	17	184	202	2187,15
[KR].DMPIQAFLLYQEPVLGPVR.[G]	1xOxidation [M2]	17	184	202	2202,16
[KR].DMPIQAFLLYQEPVLGPVR.[G]	1xDeamidated [Q]; 1xOxidation [M2]	17	184	202	2203,15
[K-].IHPFAQTQSLVYPFPGPIPK.[S]		3	49	68	2237,21
[K-].IHPFAQTQSLVYPFPGPIPK.[S]	1xDeamidated [Q]	3	49	68	2238,20
[K].IEKFQSEEQQQMEDELQDK.[I]	1xPhospho [S6]	1	30	48	2462,04
[K].IEKFQSEEQQQMEDELQDK.[I]	1xDeamidated [Q9]; 1xPhospho [S6]; 1xOxidation [M12]	1	30	48	2479,02
[K].SLPQNIPPLTQTPVVVPPFLQPEIMGVSK.[V]		8	69	97	3126,73
[-].RELEELNVPGEIVESLSSSEESITHINK.[K]	3xPhospho [S17; S18; S19]	2	1	28	3378,48
[-].RELEELNVPGEIVESLSSSEESITHINK.[K]	4xPhospho [S15; S17; S18; S19]	2	1	28	3458,45
[-].RELEELNVPGEIVESLSSSEESITHINK.[K]	1xDeamidated [N27]; 4xPhospho [S15; S17; S18; S19]	2	1	28	3459,43
**Alpha s2-casein OS=Bubalus bubalis**					
[K].ALNEINQFYQK.[F]		8	81	91	1367,70
[K].KTVDMESTEVITK.[K]	1xPhospho [S/T]	5	137	149	1560,72
[K].TVDMESTEVITK.[K]	1xPhospho [S/T]	5	138	149	1432,63
[K].FPQYLQYLYQGPIVLNPWDQVK.[R]		7	92	113	2709,41
[K].FPQYLQYLYQGPIVLNPWDQVK.[R]	1xDeamidated [Q/N]	7	92	113	2710,39
[K].TVDMESTEVITK.[K]		5	138	149	1352,66
[K].TVDMESTEVITK.[K]	1xPhospho [S6]	5	138	149	1432,63
**Kappa-casein OS=Bubalus bubalis**					
[R].HPHPHLSFMAIPPK.[K]		23	98	111	1608,85
[R].SPAQILQWQVLPNTVPAK.[S]		5	69	86	1990,11
[R].YPSYGLNYYQQKPVALINNQFLPYPYYAKPAAVR.[S]		20	35	68	4010,06
[R].YPSYGLNYYQQKPVALINNQFLPYPYYAKPAAVR.[S]	1xDeamidated [N/Q]	20	35	68	4011,05
**Alpha-lactalbumin protein variant D OS=Bos taurus**					
[K].VGINYWLAHK.[A]		7	99	108	1200,65
Beta-lactoglobulin OS=Bubalus bubalis					
[R].TPEVDDEALEK.[F]		7	125	135	1245,58
[R].TPEVDDEALEKFDK.[A]		7	125	138	1635,77
[R].VYVEELKPTPEGDLEILLQK.[W]		8	41	60	2313,26
**Beta-lactoglobulin variant D (Fragment) OS=Bos taurus**					
[R].VYVEQLKPTPEGDLEILLQK.[W]		2	41	60	2312,27
[R].VYVEQLKPTPEGDLEILLQK.[W]	1xDeamidated [Q5]	2	41	60	2313,26

Sample IM12

Description	Modifications	# PSMs	Start	End	Theor. MH+ [Da]
**Alpha-S1-casein (Fragment) OS=Bubalus bubalis**					
[R].YLGYLEQLLR.[L]		10	91	100	1267,70
[R].FFVAPFPEVFGK.[E]		9	23	34	1384,73
[K].HQGLPQGVLNENLLR.[F]		5	8	22	1687,92
[K].HQGLPQGVLNENLLR.[F]	1xDeamidated [Q/N]	5	8	22	1688,91
[K].EPMIGVNQELAYFYPQLFR.[Q]		5	133	151	2315,15
[K].EPMIGVNQELAYFYPQLFR.[Q]	1xDeamidated [Q/N]	5	133	151	2316,14
[K].YNVPQLEIVPNLAEEQLHSMK.[E]		5	104	124	2452,25
[K].YNVPQLEIVPNLAEEQLHSMK.[E]	1xDeamidated [Q/N]	5	104	124	2453,24
[K].YNVPQLEIVPNLAEEQLHSMK.[E]	1xOxidation [M20]	5	104	124	2468,25
[K].VNELSTDIGSESTEDQAMEDIK.[Q]	1xPhospho [S10]	4	37	58	2491,04
[K].VNELSTDIGSESTEDQAMEDIK.[Q]	2xPhospho [S10; S/T]	4	37	58	2571,01
[K].KYNVPQLEIVPNLAEEQLHSMK.[E]		5	103	124	2580,35
[K].KYNVPQLEIVPNLAEEQLHSMK.[E]	1xDeamidated [Q/N]	5	103	124	2581,33
[K].VNELSTDIGSESTEDQAMEDIK.[Q]	2xPhospho [S/T]; 1xOxidation [M18]	4	37	58	2587,00
**Alpha S1 casein OS=Bos taurus**					
[K].HQGLPQEVLNENLLR.[F]		5	8	22	1759,94
[R].YLGYLEQLLR.[L]		10	91	100	1267,70
[K].EPMIGVNQELAYFYPELFR.[Q]		6	133	151	2316,14
[K].DIGSESTEDQAMEDIK.[Q]		6	43	58	1767,76
[K].VNELSKDIGSESTEDQAMEDIK.[Q]	2xPhospho [S10; T13]; 1xOxidation [M18]	6	37	58	2614,05
[K].VNELSKDIGSESTEDQAMEDIK.[Q]	1xDeamidated [N2]; 2xPhospho [S10; T13]; 1xOxidation [M18]	6	37	58	2615,04
**Beta-casein OS=Bubalus bubalis**					
[K].FQSEEQQQMEDELQDK.[I]		1	33	48	2011,85
[K].FQSEEQQQMEDELQDK.[I]	1xDeamidated [Q]	1	33	48	2012,84
[K].FQSEEQQQMEDELQDK.[I]	1xOxidation [M9]	1	33	48	2027,85
[K].FQSEEQQQMEDELQDK.[I]	1xPhospho [S3]	1	33	48	2091,82
[K].FQSEEQQQMEDELQDK.[I]	1xDeamidated [Q]; 1xPhospho [S3]	1	33	48	2092,81
[K].FQSEEQQQMEDELQDK.[I]	2xDeamidated [Q2; Q]; 1xPhospho [S3]	1	33	48	2093,79
[K].FQSEEQQQMEDELQDK.[I]	3xDeamidated [Q]; 1xPhospho [S3]	1	33	48	2094,77
[K].FQSEEQQQMEDELQDK.[I]	5xDeamidated [Q2; Q6; Q7; Q8; Q14]; 1xPhospho [S3]	1	33	48	2096,74
[K].FQSEEQQQMEDELQDK.[I]	1xPhospho [S3]; 1xOxidation [M9]	1	33	48	2107,82
[K].FQSEEQQQMEDELQDK.[I]	1xDeamidated [Q]; 1xPhospho [S3]; 1xOxidation [M9]	1	33	48	2108,80
[KR].DMPIQAFLLYQEPVLGPVR.[G]		17	184	202	2186,17
[KR].DMPIQAFLLYQEPVLGPVR.[G]	1xDeamidated [Q]	17	184	202	2187,15
[K-].IHPFAQTQSLVYPFPGPIPK.[S]		3	49	68	2237,21
[K-].IHPFAQTQSLVYPFPGPIPK.[S]	1xDeamidated [Q]	3	49	68	2238,20
[K].IEKFQSEEQQQMEDELQDK.[I]	1xPhospho [S6]	1	30	48	2462,04
[K].IEKFQSEEQQQMEDELQDK.[I]	1xDeamidated [Q9]; 1xPhospho [S6]	1	30	48	2463,03
[K].SLPQNIPPLTQTPVVVPPFLQPEIMGVSK.[V]		8	69	97	3126,73
[-].RELEELNVPGEIVESLSSSEESITHINK.[K]	2xPhospho [S]	2	1	28	3298,51
[R].ELEELNVPGEIVESLSSSEESITHINK.[K]	4xPhospho [S14; S16; S17; S21]	1	2	28	3302,34
[-].RELEELNVPGEIVESLSSSEESITHINK.[K]	3xPhospho [S/T]	2	1	28	3378,48
[-].RELEELNVPGEIVESLSSSEESITHINK.[K]	4xPhospho [S15; S17; S18; S19]	2	1	28	3458,45
**Alpha s2-casein OS=Bubalus bubalis**					
[K].KTVDMESTEVITK.[K]	1xPhospho [T/S]	5	137	149	1560,72
[K].TVDMESTEVITK.[K]	1xPhospho [S6]	5	138	149	1432,63
**Kappa-casein OS=Bubalus bubalis**					
[R].YPSYGLNYYQQKPVALINNQFLPYPYYAKPAAVR.[S]	1xDeamidated [Q/N]	20	35	68	4011,05
[R].SPAQILQWQVLPNTVPAK.[S]		5	69	86	1990,11
**Beta-lactoglobulin OS=Bubalus bubalis**					
[R].TPEVDDEALEK.[F]		7	125	135	1245,58
**Beta-lactoglobulin variant D (Fragment) OS=Bos taurus**					
[R].VYVEQLKPTPEGDLEILLQK.[W]		2	41	60	2312,27
[R].VYVEQLKPTPEGDLEILLQK.[W]	1xDeamidated [Q5]	2	41	60	2313,26

Sample IM13

Description	Modifications	# PSMs	Start	End	Theor. MH+ [Da]
**Alpha-S1-casein (Fragment) OS=Bubalus bubalis**					
[R].YLGYLEQLLR.[L]		10	91	100	1267,70
[R].FFVAPFPEVFGK.[E]		9	23	34	1384,73
[K].HQGLPQGVLNENLLR.[F]		5	8	22	1687,92
[K].HQGLPQGVLNENLLR.[F]	1xDeamidated [N/Q]	5	8	22	1688,91
[K].EPMIGVNQELAYFYPQLFR.[Q]		5	133	151	2315,15
[K].EPMIGVNQELAYFYPQLFR.[Q]	1xDeamidated [Q16]	5	133	151	2316,14
[K].YNVPQLEIVPNLAEEQLHSMK.[E]		5	104	124	2452,25
[K].YNVPQLEIVPNLAEEQLHSMK.[E]	1xDeamidated [N/Q]	5	104	124	2453,24
[K].YNVPQLEIVPNLAEEQLHSMK.[E]	1xOxidation [M20]	5	104	124	2468,25
[K].YNVPQLEIVPNLAEEQLHSMK.[E]	1xDeamidated [Q5]; 1xOxidation [M20]	5	104	124	2469,23
[K].VNELSTDIGSESTEDQAMEDIK.[Q]	1xPhospho [S/T]	4	37	58	2491,04
[K].VNELSTDIGSESTEDQAMEDIK.[Q]	1xPhospho [S10]; 1xOxidation [M18]	4	37	58	2507,04
[-].RPKQPIKHQGLPQGVLNENLLR.[F]		3	1	22	2535,46
[K].VNELSTDIGSESTEDQAMEDIK.[Q]	2xPhospho [S/T]	4	37	58	2571,01
[K].KYNVPQLEIVPNLAEEQLHSMK.[E]		5	103	124	2580,35
[K].KYNVPQLEIVPNLAEEQLHSMK.[E]	1xDeamidated [N12]	5	103	124	2581,33
[K].VNELSTDIGSESTEDQAMEDIK.[Q]	2xPhospho [S/T]; 1xOxidation [M18]	4	37	58	2587,00
[K].KYNVPQLEIVPNLAEEQLHSMK.[E]	1xOxidation [M21]	5	103	124	2596,34
[K].VNELSTDIGSESTEDQAMEDIK.[Q]	3xPhospho [S/T]	4	37	58	2650,98
[K].EKVNELSTDIGSESTEDQAMEDIK.[Q]	1xPhospho [S14]	4	35	58	2748,18
**Alpha S1 casein OS=Bos taurus**					
[K].HQGLPQEVLNENLLR.[F]		5	8	22	1759,94
[K].YKVPQLEIVPNSAEER.[L]	1xPhospho [S12]	4	104	119	1951,95
[K].HQGLPQEVLNENLLR.[F]	1xDeamidated [Q]	5	8	22	1760,93
[R].YLGYLEQLLR.[L]		10	91	100	1267,70
[K].EPMIGVNQELAYFYPELFR.[Q]		6	133	151	2316,14
[R].FFVAPFPEVFGK.[E]		9	23	34	1384,73
[K].VPQLEIVPNSAEER.[L]	1xPhospho [S10]	4	106	119	1660,79
**Beta-casein OS=Bubalus bubalis**					
[K].FQSEEQQQMEDELQDK.[I]		1	33	48	2011,85
[K].FQSEEQQQMEDELQDK.[I]	1xOxidation [M9]	1	33	48	2027,85
[K].FQSEEQQQMEDELQDK.[I]	1xPhospho [S3]	1	33	48	2091,82
[K].FQSEEQQQMEDELQDK.[I]	1xDeamidated [Q]; 1xPhospho [S3]	1	33	48	2092,81
[K].FQSEEQQQMEDELQDK.[I]	2xDeamidated [Q]; 1xPhospho [S3]	1	33	48	2093,79
[K].FQSEEQQQMEDELQDK.[I]	3xDeamidated [Q2; Q]; 1xPhospho [S3]	1	33	48	2094,77
[K].FQSEEQQQMEDELQDK.[I]	1xPhospho [S3]; 1xOxidation [M9]	1	33	48	2107,82
[K].FQSEEQQQMEDELQDK.[I]	1xDeamidated [Q]; 1xPhospho [S3]; 1xOxidation [M9]	1	33	48	2108,80
[KR].DMPIQAFLLYQEPVLGPVR.[G]		17	184	202	2186,17
[KR].DMPIQAFLLYQEPVLGPVR.[G]	1xDeamidated [Q11]	17	184	202	2187,15
[KR].DMPIQAFLLYQEPVLGPVR.[G]	1xOxidation [M2]	17	184	202	2202,16
[K-].IHPFAQTQSLVYPFPGPIPK.[S]		3	49	68	2237,21
[K-].IHPFAQTQSLVYPFPGPIPK.[S]	1xDeamidated [Q]	3	49	68	2238,20
[K].IEKFQSEEQQQMEDELQDK.[I]	1xPhospho [S6]	1	30	48	2462,04
[K].SLPQNIPPLTQTPVVVPPFLQPEIMGVSK.[V]		8	69	97	3126,73
[K].SLPQNIPPLTQTPVVVPPFLQPEIMGVSK.[V]	1xDeamidated [Q21]	8	69	97	3127,71
[-].RELEELNVPGEIVESLSSSEESITHINK.[K]	1xPhospho [S19]	2	1	28	3218,55
[-].RELEELNVPGEIVESLSSSEESITHINK.[K]	4xPhospho [S15; S/T]	2	1	28	3458,45
**Alpha s2-casein OS=Bubalus bubalis**					
[K].KTVDMESTEVITK.[K]	1xPhospho [T/S]	5	137	149	1560,72
[K].ALNEINQFYQK.[F]	1xDeamidated [N/Q]	8	81	91	1368,68
[K].FPQYLQYLYQGPIVLNPWDQVK.[R]		7	92	113	2709,41
[K].FPQYLQYLYQGPIVLNPWDQVK.[R]	1xDeamidated [Q10]	7	92	113	2710,39
[K].ALNEINQFYQK.[F]		8	81	91	1367,70
**Kappa-casein OS=Bubalus bubalis**					
[R].YPSYGLNYYQQKPVALINNQFLPYPYYAKPAAVR.[S]		20	35	68	4010,06
[R].YPSYGLNYYQQKPVALINNQFLPYPYYAKPAAVR.[S]	1xDeamidated [N18]	20	35	68	4011,05
[R].SPAQILQWQVLPNTVPAK.[S]		5	69	86	1990,11
[R].HPHPHLSFMAIPPK.[K]		23	98	111	1608,85
**Beta-lactoglobulin OS=Bubalus bubalis**					
[R].TPEVDDEALEK.[F]		7	125	135	1245,58
[K].VLVLDTDYK.[K]		13	92	100	1065,58
**Beta-lactoglobulin variant D (Fragment) OS=Bos taurus**					
[R].VYVEQLKPTPEGDLEILLQK.[W]		2	41	60	2312,27

Sample M14

Description	Modifications	# PSMs	Start	End	Theor. MH+ [Da]
**Alpha-S1-casein (Fragment) OS=Bubalus bubalis**					
[R].YLGYLEQLLR.[L]		10	91	100	1267,70
[R].FFVAPFPEVFGK.[E]		9	23	34	1384,73
[K].HQGLPQGVLNENLLR.[F]		5	8	22	1687,92
[K].HQGLPQGVLNENLLR.[F]	1xDeamidated [Q/N]	5	8	22	1688,91
[K].YKVPQLEIVPNSAEER.[L]	1xPhospho [S12]	4	104	119	1951,95
[K].EPMIGVNQELAYFYPQLFR.[Q]		5	133	151	2315,15
[K].EPMIGVNQELAYFYPQLFR.[Q]	1xDeamidated [Q/N]	5	133	151	2316,14
[K].EPMIGVNQELAYFYPQLFR.[Q]	2xDeamidated [Q16; N/Q]	5	133	151	2317,12
[K].YNVPQLEIVPNLAEEQLHSMK.[E]		5	104	124	2452,25
[K].YNVPQLEIVPNLAEEQLHSMK.[E]	1xDeamidated [N/Q]	5	104	124	2453,24
[K].YNVPQLEIVPNLAEEQLHSMK.[E]	2xDeamidated [N/Q]	5	104	124	2454,22
[K].YNVPQLEIVPNLAEEQLHSMK.[E]	1xOxidation [M20]	5	104	124	2468,25
[K].VNELSTDIGSESTEDQAMEDIK.[Q]	1xPhospho [S10]	4	37	58	2491,04
[K].VNELSTDIGSESTEDQAMEDIK.[Q]	1xPhospho [S12]; 1xOxidation [M18]	4	37	58	2507,04
[K].VNELSTDIGSESTEDQAMEDIK.[Q]	2xPhospho [S/T]	4	37	58	2571,01
[K].KYNVPQLEIVPNLAEEQLHSMK.[E]		5	103	124	2580,35
[K].KYNVPQLEIVPNLAEEQLHSMK.[E]	1xDeamidated [Q/N]	5	103	124	2581,33
[K].VNELSTDIGSESTEDQAMEDIK.[Q]	2xPhospho [S/T]; 1xOxidation [M18]	4	37	58	2587,00
[K].KYNVPQLEIVPNLAEEQLHSMK.[E]	1xOxidation [M21]	5	103	124	2596,34
[K].VNELSKDIGSESTEDQAMEDIK.[Q]	2xPhospho [S10; T13]	6	37	58	2598,06
**Alpha S1 casein OS=Bos taurus**					
[K].HQGLPQEVLNENLLR.[F]		5	8	22	1759,94
[R].YLGYLEQLLR.[L]		10	91	100	1267,70
[K].EPMIGVNQELAYFYPELFR.[Q]	1xDeamidated [N/Q]	6	133	151	2317,12
[R].FFVAPFPEVFGK.[E]		9	23	34	1384,73
**Beta-casein OS=Bubalus bubalis**					
[K].FQSEEQQQMEDELQDK.[I]		1	33	48	2011,85
[K].FQSEEQQQMEDELQDK.[I]	1xOxidation [M9]	1	33	48	2027,85
[K].FQSEEQQQMEDELQDK.[I]	1xDeamidated [Q2]; 1xOxidation [M9]	1	33	48	2028,83
[K].FQSEEQQQMEDELQDK.[I]	1xPhospho [S3]	1	33	48	2091,82
[K].FQSEEQQQMEDELQDK.[I]	1xDeamidated [Q]; 1xPhospho [S3]	1	33	48	2092,81
[K].FQSEEQQQMEDELQDK.[I]	2xDeamidated [Q]; 1xPhospho [S3]	1	33	48	2093,79
[K].FQSEEQQQMEDELQDK.[I]	3xDeamidated [Q]; 1xPhospho [S3]	1	33	48	2094,77
[K].FQSEEQQQMEDELQDK.[I]	4xDeamidated [Q2; Q6; Q14; Q]; 1xPhospho [S3]	1	33	48	2095,76
[K].FQSEEQQQMEDELQDK.[I]	1xPhospho [S3]; 1xOxidation [M9]	1	33	48	2107,82
[K].FQSEEQQQMEDELQDK.[I]	1xDeamidated [Q]; 1xPhospho [S3]; 1xOxidation [M9]	1	33	48	2108,80
[KR].DMPIQAFLLYQEPVLGPVR.[G]		17	184	202	2186,17
[KR].DMPIQAFLLYQEPVLGPVR.[G]	1xDeamidated [Q11]	17	184	202	2187,15
[KR].DMPIQAFLLYQEPVLGPVR.[G]	1xDeamidated [Q]; 1xOxidation [M2]	17	184	202	2203,15
[K-].IHPFAQTQSLVYPFPGPIPK.[S]		3	49	68	2237,21
[K-].IHPFAQTQSLVYPFPGPIPK.[S]	1xDeamidated [Q]	3	49	68	2238,20
[K].IEKFQSEEQQQMEDELQDK.[I]	1xPhospho [S6]	1	30	48	2462,04
[K].IEKFQSEEQQQMEDELQDK.[I]	1xDeamidated [Q5]; 1xPhospho [S6]	1	30	48	2463,03
[K].IEKFQSEEQQQMEDELQDK.[I]	2xDeamidated [Q9; Q10]; 1xPhospho [S6]; 1xOxidation [M12]	1	30	48	2480,01
[K].SLPQNIPPLTQTPVVVPPFLQPEIMGVSK.[V]		8	69	97	3126,73
[K].SLPQNIPPLTQTPVVVPPFLQPEIMGVSK.[V]	1xDeamidated [N/Q]	8	69	97	3127,71
[K].SLPQNIPPLTQTPVVVPPFLQPEIMGVSK.[V]	1xDeamidated [Q/N]; 1xOxidation [M25]	8	69	97	3143,71
[-].RELEELNVPGEIVESLSSSEESITHINK.[K]	2xPhospho [S18; S22]	2	1	28	3298,51
[-].RELEELNVPGEIVESLSSSEESITHINK.[K]	3xPhospho [S17; S18; S19]	2	1	28	3378,48
[-].RELEELNVPGEIVESLSSSEESITHINK.[K]	4xPhospho [S15; S17; S18; S19]	2	1	28	3458,45
[-].RELEELNVPGEIVESLSSSEESITHINK.[K]	4xPhospho [S15; S17; S18; S19]	2	1	28	3458,45
[-].RELEELNVPGEIVESLSSSEESITHINKK.[I]	3xPhospho [S17; S18; S19]	1	1	29	3506,57
**Alpha s2-casein OS=Bubalus bubalis**					
[K].KTVDMESTEVITK.[K]	1xPhospho [T/S]	5	137	149	1560,72
**Kappa-casein OS=Bubalus bubalis**					
[R].YPSYGLNYYQQKPVALINNQFLPYPYYAKPAAVR.[S]		20	35	68	4010,06
[R].YPSYGLNYYQQKPVALINNQFLPYPYYAKPAAVR.[S]	1xDeamidated [Q/N]	20	35	68	4011,05
[R].SPAQILQWQVLPNTVPAK.[S]		5	69	86	1990,11
**Beta-lactoglobulin OS=Bubalus bubalis**					
[R].TPEVDDEALEK.[F]		7	125	135	1245,58
**Beta-lactoglobulin variant D (Fragment) OS=Bos taurus**					
[R].VYVEQLKPTPEGDLEILLQK.[W]	1xDeamidated [Q5]	2	41	60	2313,26

Sample IM15

Description	Modifications	# PSMs	Start	End	Theor. MH+ [Da]
**Alpha-S1-casein (Fragment) OS=Bubalus bubalis**					
[R].YLGYLEQLLR.[L]		10	91	100	1267,70
[K].HQGLPQGVLNENLLR.[F]		5	8	22	1687,92
[K].HQGLPQGVLNENLLR.[F]	1xDeamidated [Q/N]	5	8	22	1688,91
[K].YNVPQLEIVPNLAEEQLHSMK.[E]		5	104	124	2452,25
[K].YNVPQLEIVPNLAEEQLHSMK.[E]	1xOxidation [M20]	5	104	124	2468,25
[K].VNELSTDIGSESTEDQAMEDIK.[Q]	1xPhospho [S/T]	4	37	58	2491,04
[K].VNELSTDIGSESTEDQAMEDIK.[Q]	1xDeamidated [N2]; 1xPhospho [S/T]	4	37	58	2492,03
[K].VNELSTDIGSESTEDQAMEDIK.[Q]	1xPhospho [T/S]; 1xOxidation [M18]	4	37	58	2507,04
[K].VNELSTDIGSESTEDQAMEDIK.[Q]	2xPhospho [S/T]	4	37	58	2571,01
[K].VNELSTDIGSESTEDQAMEDIK.[Q]	2xPhospho [S/T]; 1xOxidation [M18]	4	37	58	2587,00
[K].VNELSTDIGSESTEDQAMEDIK.[Q]	3xPhospho [S10; T/S]	4	37	58	2650,98
[K].VNELSTDIGSESTEDQAMEDIK.[Q]	2xDeamidated [N2; Q16]; 3xPhospho [S5; S10; S12]; 1xOxidation [M18]	4	37	58	2668,94
[K].EKVNELSTDIGSESTEDQAMEDIK.[Q]	1xPhospho [T/S]	4	35	58	2748,18
**Alpha S1 casein OS=Bos taurus**					
[K].HQGLPQEVLNENLLR.[F]		5	8	22	1759,94
[R].YLGYLEQLLR.[L]		10	91	100	1267,70
**Beta-casein OS=Bubalus bubalis**					
[K].FQSEEQQQMEDELQDK.[I]		1	33	48	2011,85
[K].FQSEEQQQMEDELQDK.[I]	4xDeamidated [Q2; Q7; Q8; Q]	1	33	48	2015,79
[K].FQSEEQQQMEDELQDK.[I]	1xOxidation [M9]	1	33	48	2027,85
[K].FQSEEQQQMEDELQDK.[I]	1xPhospho [S3]	1	33	48	2091,82
[K].FQSEEQQQMEDELQDK.[I]	1xDeamidated [Q]; 1xPhospho [S3]	1	33	48	2092,81
[K].FQSEEQQQMEDELQDK.[I]	2xDeamidated [Q]; 1xPhospho [S3]	1	33	48	2093,79
[K].FQSEEQQQMEDELQDK.[I]	3xDeamidated [Q2; Q6; Q7]; 1xPhospho [S3]	1	33	48	2094,77
[K].FQSEEQQQMEDELQDK.[I]	4xDeamidated [Q2; Q6; Q7; Q14]; 1xPhospho [S3]	1	33	48	2095,76
[K].FQSEEQQQMEDELQDK.[I]	1xPhospho [S3]; 1xOxidation [M9]	1	33	48	2107,82
[K].FQSEEQQQMEDELQDK.[I]	1xDeamidated [Q]; 1xPhospho [S3]; 1xOxidation [M9]	1	33	48	2108,80
[KR].DMPIQAFLLYQEPVLGPVR.[G]		17	184	202	2186,17
[K-].IHPFAQTQSLVYPFPGPIPK.[S]		3	49	68	2237,21
[K].IEKFQSEEQQQMEDELQDK.[I]	1xPhospho [S6]	1	30	48	2462,04
[K].SLPQNIPPLTQTPVVVPPFLQPEIMGVSK.[V]		8	69	97	3126,73
[K].SLPQNIPPLTQTPVVVPPFLQPEIMGVSK.[V]	1xDeamidated [Q/N]	8	69	97	3127,71
[-].RELEELNVPGEIVESLSSSEESITHINK.[K]	1xPhospho [S18]	2	1	28	3218,55
[-].RELEELNVPGEIVESLSSSEESITHINK.[K]	2xPhospho [S]	2	1	28	3298,51
[R].ELEELNVPGEIVESLSSSEESITHINK.[K]	4xPhospho [S14; S/T]	1	2	28	3302,34
[-].RELEELNVPGEIVESLSSSEESITHINK.[K]	3xPhospho [S17; S18; S19]	2	1	28	3378,48
[R].ELEELNVPGEIVESLSSSEESITHINKK.[I]	4xPhospho [S14; S16; S17; T23]	1	2	29	3430,44
[-].RELEELNVPGEIVESLSSSEESITHINK.[K]	4xPhospho [S15; S/T]	2	1	28	3458,45
**Alpha s2-casein OS=Bubalus bubalis**					
[K].TVDMESTEVITK.[K]	1xPhospho [T/S]	5	138	149	1432,63
[K].ALNEINQFYQK.[F]		8	81	91	1367,70
[K].HTMEHVSSSEESIISQETYK.[Q]		7	2	21	2322,06
[K].KTVDMESTEVFTK.[K]	1xPhospho [S7]	3	137	149	1594,71
[K].TVDMESTEVFTK.[K]	1xOxidation [M4]	3	138	149	1402,64
[K].TVDMESTEVFTK.[K]	1xPhospho [S6]	3	138	149	1466,61
**Kappa-casein (Fragment) OS=Bos taurus**					
[R].YPSYGLNYYQQKPVALINNQFLPYPYYAKPAAVR.[S]		20	35	68	4010,06
**Alpha-lactalbumin protein variant D OS=Bos taurus**					
[K].VGINYWLAHK.[A]		7	99	108	1200,65
**Beta-lactoglobulin OS=Bubalus bubalis**					
[R].TPEVDDEALEK.[F]		7	125	135	1245,58
[R].VYVEELKPTPEGDLEILLQK.[W]		8	41	60	2313,26

Sample IM16

Description	Modifications	# PSMs	Start	End	Theor. MH+ [Da]
**Alpha-S1-casein (Fragment) OS=Bubalus bubalis**					
[R].YLGYLEQLLR.[L]		10	91	100	1267,70
[R].FFVAPFPEVFGK.[E]		9	23	34	1384,73
[K].HQGLPQGVLNENLLR.[F]		5	8	22	1687,92
[K].HQGLPQGVLNENLLR.[F]	1xDeamidated [N/Q]	5	8	22	1688,91
[K].HQGLPQGVLNENLLR.[F]	2xDeamidated [Q6; N12]	5	8	22	1689,89
[K].HQGLPQGVLNENLLR.[F]	3xDeamidated [Q2; Q6; N]	5	8	22	1690,88
[K].EPMIGVNQELAYFYPQLFR.[Q]		5	133	151	2315,15
[K].EPMIGVNQELAYFYPQLFR.[Q]	1xDeamidated [N/Q]	5	133	151	2316,14
[K].EPMIGVNQELAYFYPQLFR.[Q]	1xOxidation [M3]	5	133	151	2331,15
[K].YNVPQLEIVPNLAEEQLHSMK.[E]		5	104	124	2452,25
[K].YNVPQLEIVPNLAEEQLHSMK.[E]	1xDeamidated [N/Q]	5	104	124	2453,24
[K].YNVPQLEIVPNLAEEQLHSMK.[E]	1xOxidation [M20]	5	104	124	2468,25
[K].VNELSTDIGSESTEDQAMEDIK.[Q]	1xPhospho [S10]	4	37	58	2491,04
[K].VNELSTDIGSESTEDQAMEDIK.[Q]	2xPhospho [S/T]	4	37	58	2571,01
[K].KYNVPQLEIVPNLAEEQLHSMK.[E]		5	103	124	2580,35
[K].VNELSTDIGSESTEDQAMEDIK.[Q]	2xPhospho [S/T]; 1xOxidation [M18]	4	37	58	2587,00
[K].KYNVPQLEIVPNLAEEQLHSMK.[E]	1xOxidation [M21]	5	103	124	2596,34
[K].VNELSTDIGSESTEDQAMEDIK.[Q]	3xPhospho [S/T]	4	37	58	2650,98
**Alpha S1 casein OS=Bos taurus**					
[K].EPMIGVNQELAYFYPELFR.[Q]		6	133	151	2316,14
[R].FFVAPFPEVFGK.[E]		9	23	34	1384,73
[K].HQGLPQEVLNENLLR.[F]		5	8	22	**1759,94**
[R].YLGYLEQLLR.[L]		10	91	100	1267,70
[K].VNELSKDIGSESTEDQAMEDIK.[Q]	2xPhospho [S10; T13]; 1xOxidation [M18]	6	37	58	2614,05
[K].VNELSKDIGSESTEDQAMEDIK.[Q]	1xDeamidated [N2]; 2xPhospho [S10; T13]; 1xOxidation [M18]	6	37	58	2615,04
**Beta-casein OS=Bubalus bubalis**					
[K].FQSEEQQQMEDELQDK.[I]		1	33	48	2011,85
[K].FQSEEQQQMEDELQDK.[I]	1xOxidation [M9]	1	33	48	2027,85
[K].FQSEEQQQMEDELQDK.[I]	1xPhospho [S3]	1	33	48	2091,82
[K].FQSEEQQQMEDELQDK.[I]	1xDeamidated [Q14]; 1xPhospho [S3]	1	33	48	2092,81
[K].FQSEEQQQMEDELQDK.[I]	2xDeamidated [Q2; Q14]; 1xPhospho [S3]	1	33	48	2093,79
[K].FQSEEQQQMEDELQDK.[I]	3xDeamidated [Q2; Q]; 1xPhospho [S3]	1	33	48	2094,77
[K].FQSEEQQQMEDELQDK.[I]	1xPhospho [S3]; 1xOxidation [M9]	1	33	48	2107,82
[K].FQSEEQQQMEDELQDK.[I]	1xDeamidated [Q]; 1xPhospho [S3]; 1xOxidation [M9]	1	33	48	2108,80
[K].FQSEEQQQMEDELQDK.[I]	2xDeamidated [Q2; Q]; 1xPhospho [S3]; 1xOxidation [M9]	1	33	48	2109,78
[K].FQSEEQQQMEDELQDK.[I]	4xDeamidated [Q2; Q6; Q7; Q]; 1xPhospho [S3]; 1xOxidation [M9]	1	33	48	2111,75
[KR].DMPIQAFLLYQEPVLGPVR.[G]		17	184	202	2186,17
[KR].DMPIQAFLLYQEPVLGPVR.[G]	1xOxidation [M2]	17	184	202	2202,16
[K-].IHPFAQTQSLVYPFPGPIPK.[S]		3	49	68	2237,21
[K-].IHPFAQTQSLVYPFPGPIPK.[S]	1xDeamidated [Q]	3	49	68	2238,20
[K].IEKFQSEEQQQMEDELQDK.[I]	1xPhospho [S6]	1	30	48	2462,04
[K].SLPQNIPPLTQTPVVVPPFLQPEIMGVSK.[V]		8	69	97	3126,73
[-].RELEELNVPGEIVESLSSSEESITHINK.[K]	2xPhospho [S]	2	1	28	3298,51
[-].RELEELNVPGEIVESLSSSEESITHINK.[K]	2xPhospho [S18; S19]	2	1	28	3298,51
[R].ELEELNVPGEIVESLSSSEESITHINK.[K]	4xPhospho [S]	1	2	28	3302,34
[-].RELEELNVPGEIVESLSSSEESITHINK.[K]	4xPhospho [S15; S17; S18; S19]	2	1	28	3458,45
[-].RELEELNVPGEIVESLSSSEESITHINK.[K]	1xDeamidated [N27]; 4xPhospho [S15; S17; S18; S19]	2	1	28	3459,43
[-].RELEELNVPGEIVESLSSSEESITHINKK.[I]	3xPhospho [S17; S18; S19]	1	1	29	3506,57
**Alpha-S2-casein (Fragment) OS=Bubalus bubalis**					
[K].ALNEINQFYQK.[F]		8	81	91	1367,70
[K].ALNEINQFYQK.[F]	1xDeamidated [N6]	8	81	91	1368,68
[K].FPQYLQYLYQGPIVLNPWDQVK.[R]		7	92	113	2709,41
[K].FPQYLQYLYQGPIVLNPWDQVK.[R]	1xDeamidated [Q10]	7	92	113	2710,39
[K].TVDMESTEVITK.[K]		5	138	149	1352,66
[K].KTVDMESTEVITK.[K]	1xPhospho [S7]	5	137	149	1560,72
[K].TVDMESTEVITK.[K]	1xPhospho [S/T]	5	138	149	1432,63
**Kappa-casein OS=Bubalus bubalis**					
[R].SPAQILQWQVLPNTVPAK.[S]		5	69	86	1990,11
[R].SPAQILQWQVLPNTVPAK.[S]	2xDeamidated [N13; Q]	5	69	86	1992,08
[R].YPSYGLNYYQQKPVALINNQFLPYPYYAKPAAVR.[S]		20	35	68	4010,06
**Beta-lactoglobulin OS=Bubalus bubalis**					
[R].TPEVDDEALEK.[F]		7	125	135	1245,58
[R].VYVEELKPTPEGDLEILLQK.[W]		8	41	60	2313,26

Sample IM17

Description	Modifications	# PSMs	Start	End	Theor. MH+ [Da]
**Alpha-S1-casein (Fragment) OS=Bubalus bubalis**					
[R].YLGYLEQLLR.[L]		10	91	100	1267,70
[R].YLGYLEQLLR.[L]	1xDeamidated [Q7]	10	91	100	1268,69
[R].FFVAPFPEVFGK.[E]		9	23	34	1384,73
[K].HQGLPQGVLNENLLR.[F]		5	8	22	1687,92
[K].HQGLPQGVLNENLLR.[F]	1xDeamidated [Q/N]	5	8	22	1688,91
[K].EPMIGVNQELAYFYPQLFR.[Q]		5	133	151	2315,15
[K].EPMIGVNQELAYFYPQLFR.[Q]	1xDeamidated [Q/N]	5	133	151	2316,14
[K].YNVPQLEIVPNLAEEQLHSMK.[E]		5	104	124	2452,25
[K].YNVPQLEIVPNLAEEQLHSMK.[E]	1xDeamidated [Q/N]	5	104	124	2453,24
[K].YNVPQLEIVPNLAEEQLHSMK.[E]	2xDeamidated [N2; N11]	5	104	124	2454,22
[K].YNVPQLEIVPNLAEEQLHSMK.[E]	1xOxidation [M20]	5	104	124	2468,25
[K].VNELSTDIGSESTEDQAMEDIK.[Q]	1xPhospho [S/T]	4	37	58	2491,04
[K].VNELSTDIGSESTEDQAMEDIK.[Q]	2xPhospho [S10; S/T]	4	37	58	2571,01
[K].KYNVPQLEIVPNLAEEQLHSMK.[E]		5	103	124	2580,35
[K].KYNVPQLEIVPNLAEEQLHSMK.[E]	1xDeamidated [N/Q]	5	103	124	2581,33
[K].VNELSTDIGSESTEDQAMEDIK.[Q]	2xPhospho [S10; T13]; 1xOxidation [M18]	4	37	58	2587,00
[K].KYNVPQLEIVPNLAEEQLHSMK.[E]	1xOxidation [M21]	5	103	124	2596,34
[K].KYNVPQLEIVPNLAEEQLHSMK.[E]	1xDeamidated [N/Q]; 1xOxidation [M21]	5	103	124	2597,33
[K].VNELSTDIGSESTEDQAMEDIK.[Q]	3xPhospho [S/T]	4	37	58	2650,98
[K].VNELSTDIGSESTEDQAMEDIK.[Q]	3xPhospho [S5; S10; S12]; 1xOxidation [M18]	4	37	58	2666,97
**Alpha S1 casein OS=Bos taurus**					
[R].YLGYLEQLLR.[L]		10	91	100	1267,70
[K].VNELSKDIGSESTEDQAMEDIK.[Q]	1xPhospho [T/S]	6	37	58	2518,09
**Beta-casein OS=Bubalus bubalis**					
[K].FQSEEQQQMEDELQDK.[I]		1	33	48	2011,85
[K].FQSEEQQQMEDELQDK.[I]	1xOxidation [M9]	1	33	48	2027,85
[K].FQSEEQQQMEDELQDK.[I]	1xPhospho [S3]	1	33	48	2091,82
[K].FQSEEQQQMEDELQDK.[I]	1xDeamidated [Q]; 1xPhospho [S3]	1	33	48	2092,81
[K].FQSEEQQQMEDELQDK.[I]	2xDeamidated [Q]; 1xPhospho [S3]	1	33	48	2093,79
[K].FQSEEQQQMEDELQDK.[I]	3xDeamidated [Q2; Q]; 1xPhospho [S3]	1	33	48	2094,77
[K].FQSEEQQQMEDELQDK.[I]	4xDeamidated [Q2; Q]; 1xPhospho [S3]	1	33	48	2095,76
[K].FQSEEQQQMEDELQDK.[I]	1xPhospho [S3]; 1xOxidation [M9]	1	33	48	2107,82
[K].FQSEEQQQMEDELQDK.[I]	1xDeamidated [Q]; 1xPhospho [S3]; 1xOxidation [M9]	1	33	48	2108,80
[KR].DMPIQAFLLYQEPVLGPVR.[G]		17	184	202	2186,17
[KR].DMPIQAFLLYQEPVLGPVR.[G]	1xDeamidated [Q]	17	184	202	2187,15
[KR].DMPIQAFLLYQEPVLGPVR.[G]	1xOxidation [M2]	17	184	202	2202,16
[K-].IHPFAQTQSLVYPFPGPIPK.[S]		3	49	68	2237,21
[K].NHPFAQTQSLVYPFPGPIPK.[S]		1	7	26	2238,17
[K-].IHPFAQTQSLVYPFPGPIPK.[S]	1xDeamidated [Q8]	3	49	68	2238,20
[K].IEKFQSEEQQQMEDELQDK.[I]	1xPhospho [S6]	1	30	48	2462,04
[K].SLPQNIPPLTQTPVVVPPFLQPEIMGVSK.[V]		8	69	97	3126,73
[K].SLPQNIPPLTQTPVVVPPFLQPEIMGVSK.[V]	1xDeamidated [N5]	8	69	97	3127,71
[-].RELEELNVPGEIVESLSSSEESITHINK.[K]	1xPhospho [S]	2	1	28	3218,55
[R].ELEELNVPGEIVESLSSSEESITHINKK.[I]	4xPhospho [S14; S16; S17; S18]	1	2	29	3430,44
[-].RELEELNVPGEIVESLSSSEESITHINK.[K]	4xPhospho [S15; S17; S18; S19]	2	1	28	3458,45
**Alpha-S2-casein OS=Bubalus bubalis**					
[K].ALNEINQFYQK.[F]		8	81	91	1367,70
[K].ALNEINQFYQK.[F]	1xDeamidated [N/Q]	8	81	91	1368,68
[K].FPQYLQYLYQGPIVLNPWDQVK.[R]		7	92	113	2709,41
[K].HTMEHVSSSEESIISQETYK.[Q]		7	2	21	2322,06
[K].KTVDMESTEVFTK.[K]	1xPhospho [S7]	3	137	149	1594,71
[K].ISQHYQK.[F]		9	167	173	903,47
[K].KTVDMESTEVITK.[K]	1xPhospho [S7]	5	137	149	1560,72
[K].TVDMESTEVITK.[K]	1xPhospho [T/S]	5	138	149	1432,63
**Kappa-casein OS=Bubalus bubalis**					
[R].HPHPHLSFMAIPPK.[K]		23	98	111	1608,85
[R].SPAQILQWQVLPNTVPAK.[S]		5	69	86	1990,11
[R].SPAQILQWQVLPNTVPAK.[S]	2xDeamidated [Q9; N13]	5	69	86	1992,08
[K-].YIPIQYVLSR.[Y]		12	25	34	1251,71
[R].YPSYGLNYYQQKPVALINNQFLPYPYYAKPAAVR.[S]		20	35	68	4010,06
**Alpha-lactalbumin protein variant D OS=Bos taurus**					
[K].VGINYWLAHK.[A]		7	99	108	1200,65
**Beta-lactoglobulin OS=Bubalus bubalis**					
[R].TPEVDDEALEK.[F]		7	125	135	1245,58

Sample IM18

Description	Modifications	# PSMs	Start	End	Theor. MH+ [Da]
**Alpha-S1-casein (Fragment) OS=Bubalus bubalis**					
[R].YLGYLEQLLR.[L]		10	91	100	1267,70
[R].FFVAPFPEVFGK.[E]		9	23	34	1384,73
[K].HQGLPQGVLNENLLR.[F]		5	8	22	1687,92
[K].HQGLPQGVLNENLLR.[F]	1xDeamidated [N/Q]	5	8	22	1688,91
[K].EPMIGVNQELAYFYPQLFR.[Q]		5	133	151	2315,15
[K].EPMIGVNQELAYFYPQLFR.[Q]	1xDeamidated [Q/N]	5	133	151	2316,14
[K].EPMIGVNQELAYFYPQLFR.[Q]	1xOxidation [M3]	5	133	151	2331,15
[K].YNVPQLEIVPNLAEEQLHSMK.[E]		5	104	124	2452,25
[K].YNVPQLEIVPNLAEEQLHSMK.[E]	1xDeamidated [Q/N]	5	104	124	2453,24
[K].YNVPQLEIVPNLAEEQLHSMK.[E]	1xOxidation [M20]	5	104	124	2468,25
[K].YNVPQLEIVPNLAEEQLHSMK.[E]	1xDeamidated [Q/N]; 1xOxidation [M20]	5	104	124	2469,23
[K].VNELSTDIGSESTEDQAMEDIK.[Q]	1xPhospho [S10]	4	37	58	2491,04
[K].VNELSTDIGSESTEDQAMEDIK.[Q]	2xDeamidated [N2; Q16]; 1xPhospho [S10]	4	37	58	2493,01
[K].VNELSTDIGSESTEDQAMEDIK.[Q]	2xPhospho [S/T]	4	37	58	2571,01
[K].KYNVPQLEIVPNLAEEQLHSMK.[E]		5	103	124	2580,35
[K].KYNVPQLEIVPNLAEEQLHSMK.[E]	1xDeamidated [Q6]	5	103	124	2581,33
[K].VNELSTDIGSESTEDQAMEDIK.[Q]	2xPhospho [S10; T13]; 1xOxidation [M18]	4	37	58	2587,00
[K].KYNVPQLEIVPNLAEEQLHSMK.[E]	1xOxidation [M21]	5	103	124	2596,34
[K].VNELSTDIGSESTEDQAMEDIK.[Q]	3xPhospho [T/S]	4	37	58	2650,98
**Beta-casein OS=Bubalus bubalis**					
[K].FQSEEQQQMEDELQDK.[I]		1	33	48	2011,85
[K].FQSEEQQQMEDELQDK.[I]	1xOxidation [M9]	1	33	48	2027,85
[K].FQSEEQQQMEDELQDK.[I]	1xPhospho [S3]	1	33	48	2091,82
[K].FQSEEQQQMEDELQDK.[I]	1xDeamidated [Q]; 1xPhospho [S3]	1	33	48	2092,81
[K].FQSEEQQQMEDELQDK.[I]	2xDeamidated [Q]; 1xPhospho [S3]	1	33	48	2093,79
[K].FQSEEQQQMEDELQDK.[I]	3xDeamidated [Q2; Q]; 1xPhospho [S3]	1	33	48	2094,77
[K].FQSEEQQQMEDELQDK.[I]	4xDeamidated [Q2; Q]; 1xPhospho [S3]	1	33	48	2095,76
[K].FQSEEQQQMEDELQDK.[I]	1xPhospho [S3]; 1xOxidation [M9]	1	33	48	2107,82
[K].FQSEEQQQMEDELQDK.[I]	1xDeamidated [Q]; 1xPhospho [S3]; 1xOxidation [M9]	1	33	48	2108,80
[K].FQSEEQQQMEDELQDK.[I]	3xDeamidated [Q6; Q7; Q8]; 1xPhospho [S3]; 1xOxidation [M9]	1	33	48	2110,77
[KR].DMPIQAFLLYQEPVLGPVR.[G]		17	184	202	2186,17
[KR].DMPIQAFLLYQEPVLGPVR.[G]	1xDeamidated [Q]	17	184	202	2187,15
[KR].DMPIQAFLLYQEPVLGPVR.[G]	1xOxidation [M2]	17	184	202	2202,16
[K-].IHPFAQTQSLVYPFPGPIPK.[S]		3	49	68	2237,21
[K-].IHPFAQTQSLVYPFPGPIPK.[S]	1xDeamidated [Q8]	3	49	68	2238,20
[K].IEKFQSEEQQQMEDELQDK.[I]	1xPhospho [S6]	1	30	48	2462,04
[K].IEKFQSEEQQQMEDELQDK.[I]	1xDeamidated [Q]; 1xPhospho [S6]	1	30	48	2463,03
[K].IEKFQSEEQQQMEDELQDK.[I]	1xPhospho [S6]; 1xOxidation [M12]	1	30	48	2478,04
[K].SLPQNIPPLTQTPVVVPPFLQPEIMGVSK.[V]		8	69	97	3126,73
[K].SLPQNIPPLTQTPVVVPPFLQPEIMGVSK.[V]	1xDeamidated [Q/N]	8	69	97	3127,71
[K].SLPQNIPPLTQTPVVVPPFLQPEIMGVSK.[V]	1xDeamidated [Q/N]; 1xOxidation [M25]	8	69	97	3143,71
[-].RELEELNVPGEIVESLSSSEESITHINK.[K]	2xPhospho [S]	2	1	28	3298,51
[-].RELEELNVPGEIVESLSSSEESITHINK.[K]	2xPhospho [S]	2	1	28	3298,51
[-].RELEELNVPGEIVESLSSSEESITHINKK.[I]	2xPhospho [S18; S19]	1	1	29	3426,61
[-].RELEELNVPGEIVESLSSSEESITHINK.[K]	4xPhospho [S15; S17; S18; S19]	2	1	28	3458,45
[-].RELEELNVPGEIVESLSSSEESITHINKK.[I]	3xPhospho [S17; S18; S19]	1	1	29	3506,57
[-].RELEELNVPGEIVESLSSSEESITHINKK.[I]	1xDeamidated [N27]; 3xPhospho [S17; S18; S19]	1	1	29	3507,56
[-].RELEELNVPGEIVESLSSSEESITHINKK.[I]	4xPhospho [S15; S17; S18; S22]	1	1	29	3586,54
**Alpha s2-casein OS=Bubalus bubalis**					
[K].KTVDMESTEVITK.[K]	1xPhospho [S7]	5	137	149	1560,72
[K].TVDMESTEVITK.[K]	1xPhospho [S/T]	5	138	149	1432,63
[K].ALNEINQFYQK.[F]		8	81	91	1367,70
[K].FPQYLQYLYQGPIVLNPWDQVK.[R]		7	92	113	2709,41
[K].FPQYLQYLYQGPIVLNPWDQVK.[R]	1xDeamidated [N/Q]	7	92	113	2710,39
[K].KTVDMESTEVITK.[K]	1xPhospho [S7]; 1xOxidation [M5]	5	137	149	1576,72
[K].TVDMESTEVITK.[K]		5	138	149	1352,66
**Kappa-casein OS=Bubalus bubalis**					
[R].SPAQILQWQVLPNTVPAK.[S]		5	69	86	1990,11
[R].YPSYGLNYYQQKPVALINNQFLPYPYYAKPAAVR.[S]		20	35	68	4010,06
[R].YPSYGLNYYQQKPVALINNQFLPYPYYAKPAAVR.[S]	1xDeamidated [N/Q]	20	35	68	4011,05
**Alpha-lactalbumin protein variant D OS=Bos taurus**					
[K].VGINYWLAHK.[A]		7	99	108	1200,65
**Beta-lactoglobulin OS=Bubalus bubalis**					
[R].TPEVDDEALEK.[F]		7	125	135	1245,58
[R].VYVEELKPTPEGDLEILLQK.[W]		8	41	60	2313,26
**Beta-lactoglobulin variant D (Fragment) OS=Bos taurus**					
[R].VYVEQLKPTPEGDLEILLQK.[W]		2	41	60	2312,27
[R].VYVEQLKPTPEGDLEILLQK.[W]	1xDeamidated [Q5]	2	41	60	2313,26

Sample IM19

Description	Modifications	# PSMs	Start	End	Theor. MH+ [Da]
**Alpha-S1-casein (Fragment) OS=Bubalus bubalis**					
[R].YLGYLEQLLR.[L]		10	91	100	1267,70
[R].FFVAPFPEVFGK.[E]		9	23	34	1384,73
[K].HQGLPQGVLNENLLR.[F]		5	8	22	1687,92
[K].HQGLPQGVLNENLLR.[F]	1xDeamidated [N/Q]	5	8	22	1688,91
[K].HQGLPQGVLNENLLR.[F]	3xDeamidated [Q6; Q/N]	5	8	22	1690,88
[K].EPMIGVNQELAYFYPQLFR.[Q]		5	133	151	2315,15
[K].YNVPQLEIVPNLAEEQLHSMK.[E]		5	104	124	2452,25
[K].YNVPQLEIVPNLAEEQLHSMK.[E]	1xDeamidated [Q/N]	5	104	124	2453,24
[K].YNVPQLEIVPNLAEEQLHSMK.[E]	1xOxidation [M20]	5	104	124	2468,25
[K].VNELSTDIGSESTEDQAMEDIK.[Q]	1xPhospho [S12]	4	37	58	2491,04
[K].VNELSTDIGSESTEDQAMEDIK.[Q]	2xPhospho [S/T]	4	37	58	2571,01
[K].KYNVPQLEIVPNLAEEQLHSMK.[E]		5	103	124	2580,35
[K].KYNVPQLEIVPNLAEEQLHSMK.[E]	1xDeamidated [Q6]	5	103	124	2581,33
[K].KYNVPQLEIVPNLAEEQLHSMK.[E]	2xDeamidated [N3; Q6]	5	103	124	2582,32
[K].VNELSTDIGSESTEDQAMEDIK.[Q]	2xPhospho [S/T]; 1xOxidation [M18]	4	37	58	2587,00
[K].KYNVPQLEIVPNLAEEQLHSMK.[E]	1xOxidation [M21]	5	103	124	2596,34
[K].KYNVPQLEIVPNLAEEQLHSMK.[E]	1xDeamidated [N/Q]; 1xOxidation [M21]	5	103	124	2597,33
[K].VNELSTDIGSESTEDQAMEDIK.[Q]	3xPhospho [S/T]	4	37	58	2650,98
[K].VNELSTDIGSESTEDQAMEDIK.[Q]	3xPhospho [S5; S10; T13]; 1xOxidation [M18]	4	37	58	2666,97
**Beta-casein OS=Bubalus bubalis**					
[K].FQSEEQQQMEDELQDK.[I]		1	33	48	2011,85
[K].FQSEEQQQMEDELQDK.[I]	1xOxidation [M9]	1	33	48	2027,85
[K].FQSEEQQQMEDELQDK.[I]	1xPhospho [S3]	1	33	48	2091,82
[K].FQSEEQQQMEDELQDK.[I]	1xDeamidated [Q14]; 1xPhospho [S3]	1	33	48	2092,81
[K].FQSEEQQQMEDELQDK.[I]	2xDeamidated [Q]; 1xPhospho [S3]	1	33	48	2093,79
[K].FQSEEQQQMEDELQDK.[I]	3xDeamidated [Q]; 1xPhospho [S3]	1	33	48	2094,77
[K].FQSEEQQQMEDELQDK.[I]	1xPhospho [S3]; 1xOxidation [M9]	1	33	48	2107,82
[K].FQSEEQQQMEDELQDK.[I]	1xDeamidated [Q2]; 1xPhospho [S3]; 1xOxidation [M9]	1	33	48	2108,80
[K].FQSEEQQQMEDELQDK.[I]	2xDeamidated [Q]; 1xPhospho [S3]; 1xOxidation [M9]	1	33	48	2109,78
[KR].DMPIQAFLLYQEPVLGPVR.[G]		17	184	202	2186,17
[KR].DMPIQAFLLYQEPVLGPVR.[G]	1xDeamidated [Q]	17	184	202	2187,15
[KR].DMPIQAFLLYQEPVLGPVR.[G]	2xDeamidated [Q5; Q11]	17	184	202	2188,14
[KR].DMPIQAFLLYQEPVLGPVR.[G]	1xDeamidated [Q]; 1xOxidation [M2]	17	184	202	2203,15
[K-].IHPFAQTQSLVYPFPGPIPK.[S]		3	49	68	2237,21
[K-].IHPFAQTQSLVYPFPGPIPK.[S]	1xDeamidated [Q]	3	49	68	2238,20
[K].IEKFQSEEQQQMEDELQDK.[I]	1xPhospho [S6]	1	30	48	2462,04
[K].IEKFQSEEQQQMEDELQDK.[I]	1xDeamidated [Q]; 1xPhospho [S6]	1	30	48	2463,03
[K].SLPQNIPPLTQTPVVVPPFLQPEIMGVSK.[V]		8	69	97	3126,73
[K].SLPQNIPPLTQTPVVVPPFLQPEIMGVSK.[V]	1xDeamidated [Q/N]	8	69	97	3127,71
[K].SLPQNIPPLTQTPVVVPPFLQPEIMGVSK.[V]	1xOxidation [M25]	8	69	97	3142,72
[K].SLPQNIPPLTQTPVVVPPFLQPEIMGVSK.[V]	1xDeamidated [Q/N]; 1xOxidation [M25]	8	69	97	3143,71
[-].RELEELNVPGEIVESLSSSEESITHINK.[K]	1xPhospho [S]	2	1	28	3218,55
[-].RELEELNVPGEIVESLSSSEESITHINK.[K]	2xPhospho [S18; S]	2	1	28	3298,51
[R].ELEELNVPGEIVESLSSSEESITHINK.[K]	4xPhospho [S/T]	1	2	28	3302,34
[-].RELEELNVPGEIVESLSSSEESITHINK.[K]	4xPhospho [S15; S17; S18; S19]	2	1	28	3458,45
[-].RELEELNVPGEIVESLSSSEESITHINKK.[I]	3xPhospho [S17; S18; S19]	1	1	29	3506,57
[-].RELEELNVPGEIVESLSSSEESITHINKK.[I]	4xPhospho [S15; S17; S18; S19]	1	1	29	3586,54
**Alpha s2-casein OS=Bubalus bubalis**					
[K].ALNEINQFYQK.[F]		8	81	91	1367,70
[K].ALNEINQFYQK.[F]	1xDeamidated [Q/N]	8	81	91	1368,68
[K].TVDMESTEVITK.[K]	1xPhospho [S/T]	5	138	149	1432,63
[K].KTVDMESTEVITK.[K]	1xPhospho [T/S]	5	137	149	1560,72
[K].FPQYLQYLYQGPIVLNPWDQVK.[R]		7	92	113	2709,41
**Kappa-casein OS=Bubalus bubalis**					
[R].SPAQILQWQVLPNTVPAK.[S]		5	69	86	1990,11
[R].SPAQILQWQVLPNTVPAK.[S]	1xDeamidated [N13]	5	69	86	1991,10
[R].SPAQILQWQVLPNTVPAK.[S]	2xDeamidated [Q9; N13]	5	69	86	1992,08
Alpha-lactalbumin protein variant D OS=Bos taurus					
[K].VGINYWLAHK.[A]		7	99	108	1200,65
**Beta-lactoglobulin OS=Bubalus bubalis**					
[R].TPEVDDEALEK.[F]		7	125	135	1245,58
**Beta-lactoglobulin variant D (Fragment) OS=Bos taurus**					
[R].VYVEQLKPTPEGDLEILLQK.[W]	1xDeamidated [Q5]	2	41	60	2313,26

Sample IM20

Description	Modifications	# PSMs	Start	End	Theor. MH+ [Da]
**Alpha-S1-casein OS=Bubalus bubalis**					
[R].YLGYLEQLLR.[L]		10	91	100	1267,70
[R].FFVAPFPEVFGK.[E]		9	23	34	1384,73
[K].HQGLPQGVLNENLLR.[F]		5	8	22	1687,92
[K].HQGLPQGVLNENLLR.[F]	1xDeamidated [N/Q]	5	8	22	1688,91
[K].EPMIGVNQELAYFYPQLFR.[Q]		5	133	151	2315,15
[K].EPMIGVNQELAYFYPQLFR.[Q]	1xDeamidated [Q16]	5	133	151	2316,14
[K].EPMIGVNQELAYFYPQLFR.[Q]	1xOxidation [M3]	5	133	151	2331,15
[K].YNVPQLEIVPNLAEEQLHSMK.[E]		5	104	124	2452,25
[K].YNVPQLEIVPNLAEEQLHSMK.[E]	1xDeamidated [N/Q]	5	104	124	2453,24
[K].YNVPQLEIVPNLAEEQLHSMK.[E]	1xOxidation [M20]	5	104	124	2468,25
[K].YNVPQLEIVPNLAEEQLHSMK.[E]	1xDeamidated [Q/N]; 1xOxidation [M20]	5	104	124	2469,23
[K].VNELSTDIGSESTEDQAMEDIK.[Q]	1xPhospho [S10]	4	37	58	2491,04
[K].VNELSTDIGSESTEDQAMEDIK.[Q]	1xPhospho [S10]; 1xOxidation [M18]	4	37	58	2507,04
[-].RPKQPIKHQGLPQGVLNENLLR.[F]		3	1	22	2535,46
[-].RPKQPIKHQGLPQGVLNENLLR.[F]	1xDeamidated [Q4]	3	1	22	2536,45
[K].VNELSTDIGSESTEDQAMEDIK.[Q]	2xPhospho [S/T]	4	37	58	2571,01
[K].KYNVPQLEIVPNLAEEQLHSMK.[E]		5	103	124	2580,35
[K].VNELSTDIGSESTEDQAMEDIK.[Q]	2xPhospho [S/T]; 1xOxidation [M18]	4	37	58	2587,00
[K].KYNVPQLEIVPNLAEEQLHSMK.[E]	1xOxidation [M21]	5	103	124	2596,34
[K].VNELSTDIGSESTEDQAMEDIK.[Q]	3xPhospho [S/T]	4	37	58	2650,98
[K].EKVNELSTDIGSESTEDQAMEDIK.[Q]	1xPhospho [S12]	4	35	58	2748,18
[K].EKVNELSTDIGSESTEDQAMEDIK.[Q]	1xPhospho [S12]; 1xOxidation [M20]	4	35	58	2764,18
**Alpha S1 casein OS=Bos taurus**					
[R].YLGYLEQLLR.[L]		10	91	100	1267,70
[R].FFVAPFPEVFGK.[E]		9	23	34	1384,73
[K].HQGLPQEVLNENLLR.[F]		5	8	22	**1759,94**
[K].DIGSESTEDQAMEDIK.[Q]		6	43	58	1767,76
[K].EPMIGVNQELAYFYPELFR.[Q]		6	133	151	2316,14
**Beta-casein OS=Bubalus bubalis**					
[K].FQSEEQQQMEDELQDK.[I]		1	33	48	2011,85
[K].FQSEEQQQMEDELQDK.[I]	1xDeamidated [Q]	1	33	48	2012,84
[K].FQSEEQQQMEDELQDK.[I]	2xDeamidated [Q2; Q14]	1	33	48	2013,82
[K].FQSEEQQQMEDELQDK.[I]	1xOxidation [M9]	1	33	48	2027,85
[K].FQSEEQQQMEDELQDK.[I]	1xPhospho [S3]	1	33	48	2091,82
[K].FQSEEQQQMEDELQDK.[I]	1xDeamidated [Q]; 1xPhospho [S3]	1	33	48	2092,81
[K].FQSEEQQQMEDELQDK.[I]	2xDeamidated [Q]; 1xPhospho [S3]	1	33	48	2093,79
[K].FQSEEQQQMEDELQDK.[I]	3xDeamidated [Q2; Q6; Q7]; 1xPhospho [S3]	1	33	48	2094,77
[K].FQSEEQQQMEDELQDK.[I]	4xDeamidated [Q2; Q]; 1xPhospho [S3]	1	33	48	2095,76
[K].FQSEEQQQMEDELQDK.[I]	1xPhospho [S3]; 1xOxidation [M9]	1	33	48	2107,82
[K].FQSEEQQQMEDELQDK.[I]	1xDeamidated [Q]; 1xPhospho [S3]; 1xOxidation [M9]	1	33	48	2108,80
[KR].DMPIQAFLLYQEPVLGPVR.[G]		17	184	202	2186,17
[KR].DMPIQAFLLYQEPVLGPVR.[G]	1xDeamidated [Q11]	17	184	202	2187,15
[KR].DMPIQAFLLYQEPVLGPVR.[G]	1xDeamidated [Q5]; 1xOxidation [M2]	17	184	202	2203,15
[K-].IHPFAQTQSLVYPFPGPIPK.[S]		3	49	68	2237,21
[K].IEKFQSEEQRQTEDELQDK.[I]	1xDeamidated [Q11]; 1xPhospho [S6]	1	30	48	2461,08
[K].IEKFQSEEQQQMEDELQDK.[I]	1xPhospho [S6]	1	30	48	2462,04
[K].IEKFQSEEQQQMEDELQDK.[I]	1xDeamidated [Q17]; 1xPhospho [S6]	1	30	48	2463,03
[K].SLPQNIPPLTQTPVVVPPFLQPEIMGVSK.[V]		8	69	97	3126,73
[K].SLPQNIPPLTQTPVVVPPFLQPEIMGVSK.[V]	1xDeamidated [Q21]	8	69	97	3127,71
[K].SLPQNIPPLTQTPVVVPPFLQPEIMGVSK.[V]	1xOxidation [M25]	8	69	97	3142,72
[-].RELEELNVPGEIVESLSSSEESITHINK.[K]	1xPhospho [S17]	2	1	28	3218,55
[-].RELEELNVPGEIVESLSSSEESITHINK.[K]	3xPhospho [S/T]	2	1	28	3378,48
[-].RELEELNVPGEIVESLSSSEESITHINK.[K]	4xPhospho [S15; S17; S18; S19]	2	1	28	3458,45
[-].RELEELNVPGEIVESLSSSEESITHINKK.[I]	3xPhospho [S/T]	1	1	29	3506,57
**Alpha s2-casein OS=Bubalus bubalis**					
[K].TVDMESTEVITK.[K]		5	138	149	1352,66
[K].ALNEINQFYQK.[F]		8	81	91	1367,70
[K].ALNEINQFYQK.[F]	1xDeamidated [Q/N]	8	81	91	1368,68
[K].TVDMESTEVITK.[K]	1xPhospho [S/T]	5	138	149	1432,63
[K].KTVDMESTEVITK.[K]	1xPhospho [T/S]	5	137	149	1560,72
[K].FPQYLQYLYQGPIVLNPWDQVK.[R]		7	92	113	2709,41
**Kappa-casein OS=Bubalus bubalis**					
[R].SPAQILQWQVLPNTVPAK.[S]		5	69	86	1990,11
[R].SPAQILQWQVLPNTVPAK.[S]	1xDeamidated [N13]	5	69	86	1991,10
[R].SPAQILQWQVLPNTVPAK.[S]	2xDeamidated [N13; Q]	5	69	86	1992,08
[R].YPSYGLNYYQQKPVALINNQFLPYPYYAKPAAVR.[S]		20	35	68	4010,06
[R].YPSYGLNYYQQKPVALINNQFLPYPYYAKPAAVR.[S]	1xDeamidated [N/Q]	20	35	68	4011,05
**Alpha-lactalbumin protein variant D OS=Bos taurus**					
[K].VGINYWLAHK.[A]		7	99	108	1200,65
**Beta-lactoglobulin OS=Bubalus bubalis**					
[R].TPEVDDEALEK.[F]		7	125	135	1245,58
[R].VYVEELKPTPEGDLEILLQK.[W]		8	41	60	2313,26
**Beta-lactoglobulin variant D (Fragment) OS=Bos taurus**					
[R].VYVEQLKPTPEGDLEILLQK.[W]		2	41	60	2312,27
[R].VYVEQLKPTPEGDLEILLQK.[W]	1xDeamidated [Q5]	2	41	60	2313,26

Sample IM21

Description	Modifications	# PSMs	Start	End	Theor. MH+ [Da]
**Alpha-S1-casein (Fragment) OS=Bubalus bubalis**					
[R].YLGYLEQLLR.[L]		10	91	100	1267,70
[R].FFVAPFPEVFGK.[E]		9	23	34	1384,73
[K].HQGLPQGVLNENLLR.[F]		5	8	22	1687,92
[K].HQGLPQGVLNENLLR.[F]	1xDeamidated [N/Q]	5	8	22	1688,91
[K].HQGLPQGVLNENLLR.[F]	2xDeamidated [Q2; N/Q]	5	8	22	1689,89
[K].HQGLPQGVLNENLLR.[F]	3xDeamidated [Q2; Q6; N]	5	8	22	1690,88
[K].EPMIGVNQELAYFYPQLFR.[Q]		5	133	151	2315,15
[K].EPMIGVNQELAYFYPQLFR.[Q]	1xDeamidated [Q/N]	5	133	151	2316,14
[K].EPMIGVNQELAYFYPQLFR.[Q]	1xDeamidated [Q16]; 1xOxidation [M3]	5	133	151	2332,13
[K].YNVPQLEIVPNLAEEQLHSMK.[E]		5	104	124	2452,25
[K].YNVPQLEIVPNLAEEQLHSMK.[E]	1xOxidation [M20]	5	104	124	2468,25
[K].VNELSTDIGSESTEDQAMEDIK.[Q]	1xPhospho [S/T]	4	37	58	2491,04
[K].VNELSTDIGSESTEDQAMEDIK.[Q]	2xPhospho [S10; S/T]	4	37	58	2571,01
[K].KYNVPQLEIVPNLAEEQLHSMK.[E]		5	103	124	2580,35
[K].KYNVPQLEIVPNLAEEQLHSMK.[E]	1xDeamidated [N/Q]	5	103	124	2581,33
[K].VNELSTDIGSESTEDQAMEDIK.[Q]	2xPhospho [S/T]; 1xOxidation [M18]	4	37	58	2587,00
[K].KYNVPQLEIVPNLAEEQLHSMK.[E]	1xOxidation [M21]	5	103	124	2596,34
[K].VNELSTDIGSESTEDQAMEDIK.[Q]	3xPhospho [S/T]	4	37	58	2650,98
**Alpha S1 casein OS=Bos taurus**					
[K].EPMIGVNQELAYFYPELFR.[Q]	1xOxidation [M3]	6	133	151	2332,13
[R].FFVAPFPEVFGK.[E]		9	23	34	1384,73
[K].HQGLPQEVLNENLLR.[F]		5	8	22	1759,94
[R].YLGYLEQLLR.[L]		10	91	100	1267,70
**Beta-casein OS=Bubalus bubalis**					
[K].FQSEEQQQMEDELQDK.[I]		1	33	48	2011,85
[K].FQSEEQQQMEDELQDK.[I]	1xOxidation [M9]	1	33	48	2027,85
[K].FQSEEQQQMEDELQDK.[I]	1xPhospho [S3]	1	33	48	2091,82
[K].FQSEEQQQMEDELQDK.[I]	1xDeamidated [Q]; 1xPhospho [S3]	1	33	48	2092,81
[K].FQSEEQQQMEDELQDK.[I]	2xDeamidated [Q]; 1xPhospho [S3]	1	33	48	2093,79
[K].FQSEEQQQMEDELQDK.[I]	3xDeamidated [Q2; Q]; 1xPhospho [S3]	1	33	48	2094,77
[K].FQSEEQQQMEDELQDK.[I]	1xPhospho [S3]; 1xOxidation [M9]	1	33	48	2107,82
[K].FQSEEQQQMEDELQDK.[I]	1xDeamidated [Q]; 1xPhospho [S3]; 1xOxidation [M9]	1	33	48	2108,80
[K].FQSEEQQQMEDELQDK.[I]	2xDeamidated [Q]; 1xPhospho [S3]; 1xOxidation [M9]	1	33	48	2109,78
[KR].DMPIQAFLLYQEPVLGPVR.[G]		17	184	202	2186,17
[KR].DMPIQAFLLYQEPVLGPVR.[G]	1xDeamidated [Q11]	17	184	202	2187,15
[KR].DMPIQAFLLYQEPVLGPVR.[G]	1xOxidation [M2]	17	184	202	2202,16
[KR].DMPIQAFLLYQEPVLGPVR.[G]	1xDeamidated [Q]; 1xOxidation [M2]	17	184	202	2203,15
[K-].IHPFAQTQSLVYPFPGPIPK.[S]		3	49	68	2237,21
[K-].IHPFAQTQSLVYPFPGPIPK.[S]	1xDeamidated [Q]	3	49	68	2238,20
[K].IEKFQSEEQQQMEDELQDK.[I]	1xPhospho [S6]	1	30	48	2462,04
[K].IEKFQSEEQQQMEDELQDK.[I]	1xDeamidated [Q5]; 1xPhospho [S6]; 1xOxidation [M12]	1	30	48	2479,02
[K].SLPQNIPPLTQTPVVVPPFLQPEIMGVSK.[V]		8	69	97	3126,73
[K].SLPQNIPPLTQTPVVVPPFLQPEIMGVSK.[V]	1xDeamidated [Q/N]	8	69	97	3127,71
[K].SLPQNIPPLTQTPVVVPPFLQPEIMGVSK.[V]	1xOxidation [M25]	8	69	97	3142,72
[K].SLPQNIPPLTQTPVVVPPFLQPEIMGVSK.[V]	1xDeamidated [Q/N]; 1xOxidation [M25]	8	69	97	3143,71
[-].RELEELNVPGEIVESLSSSEESITHINK.[K]	2xPhospho [S18; S19]	2	1	28	3298,51
[R].ELEELNVPGEIVESLSSSEESITHINK.[K]	4xPhospho [S/T]	1	2	28	3302,34
[-].RELEELNVPGEIVESLSSSEESITHINK.[K]	3xPhospho [S17; S18; S19]	2	1	28	3378,48
[-].RELEELNVPGEIVESLSSSEESITHINK.[K]	4xPhospho [S15; S17; S18; S19]	2	1	28	3458,45
[-].RELEELNVPGEIVESLSSSEESITHINKK.[I]	3xPhospho [S17; S18; S19]	1	1	29	3506,57
[-].RELEELNVPGEIVESLSSSEESITHINKK.[I]	4xPhospho [S/T]	1	1	29	3586,54
**Alpha s2-casein OS=Bubalus bubalis**					
[K].ALNEINQFYQK.[F]		8	81	91	1367,70
[K].FPQYLQYLYQGPIVLNPWDQVK.[R]		7	92	113	2709,41
[K].FPQYLQYLYQGPIVLNPWDQVK.[R]	1xDeamidated [Q10]	7	92	113	2710,39
[K].TVDMESTEVITK.[K]		5	138	149	1352,66
[K].TVDMESTEVITK.[K]	1xPhospho [S6]	5	138	149	1432,63
[K].KTVDMESTEVITK.[K]	1xPhospho [S7]	5	137	149	1560,72
**Kappa-casein OS=Bubalus bubalis**					
[R].HPHPHLSFMAIPPK.[K]		23	98	111	1608,85
[R].SPAQILQWQVLPNTVPAK.[S]	1xDeamidated [N/Q]	5	69	86	1991,10
[R].YPSYGLNYYQQKPVALINNQFLPYPYYAKPAAVR.[S]		20	35	68	4010,06
**Alpha-lactalbumin protein variant D OS=Bos taurus**					
[K].VGINYWLAHK.[A]		7	99	108	1200,65
**Beta-lactoglobulin OS=Bubalus bubalis**					
[R].TPEVDDEALEK.[F]		7	125	135	1245,58
**Beta-lactoglobulin variant D (Fragment) OS=Bos taurus**					
[R].VYVEQLKPTPEGDLEILLQK.[W]		2	41	60	2312,27

Sample IM22

Description	Modifications	# PSMs	Start	End	Theor. MH+ [Da]
**Alpha-S1-casein (Fragment) OS=Bubalus bubalis**					
[R].YLGYLEQLLR.[L]		10	91	100	1267,70
[R].FFVAPFPEVFGK.[E]		9	23	34	1384,73
[K].HQGLPQGVLNENLLR.[F]		5	8	22	1687,92
[K].HQGLPQGVLNENLLR.[F]	1xDeamidated [Q/N]	5	8	22	1688,91
[K].EPMIGVNQELAYFYPQLFR.[Q]		5	133	151	2315,15
[K].EPMIGVNQELAYFYPQLFR.[Q]	1xDeamidated [Q/N]	5	133	151	2316,14
[K].EPMIGVNQELAYFYPQLFR.[Q]	1xOxidation [M3]	5	133	151	2331,15
[K].YNVPQLEIVPNLAEEQLHSMK.[E]		5	104	124	2452,25
[K].YNVPQLEIVPNLAEEQLHSMK.[E]	1xDeamidated [N2]	5	104	124	2453,24
[K].YNVPQLEIVPNLAEEQLHSMK.[E]	2xDeamidated [N2; N11]	5	104	124	2454,22
[K].YNVPQLEIVPNLAEEQLHSMK.[E]	1xOxidation [M20]	5	104	124	2468,25
[K].VNELSTDIGSESTEDQAMEDIK.[Q]	1xPhospho [S/T]	4	37	58	2491,04
[K].VNELSTDIGSESTEDQAMEDIK.[Q]	2xPhospho [S/T]	4	37	58	2571,01
[K].KYNVPQLEIVPNLAEEQLHSMK.[E]		5	103	124	2580,35
[K].VNELSTDIGSESTEDQAMEDIK.[Q]	2xPhospho [S/T]; 1xOxidation [M18]	4	37	58	2587,00
**Alpha S1 casein OS=Bos taurus**					
[R].YLGYLEQLLR.[L]		10	91	100	1267,70
[R].FFVAPFPEVFGK.[E]		9	23	34	1384,73
[K].VPQLEIVPNSAEER.[L]	1xPhospho [S10]	4	106	119	1660,79
[K].VPQLEIVPNSAEER.[L]	1xDeamidated [N/Q]; 1xPhospho [S10]	4	106	119	1661,78
[K].VPQLEIVPNSAEER.[L]	1xDeamidated [Q3]; 1xPhospho [S10]	4	106	119	1661,78
[K].HQGLPQEVLNENLLR.[F]		5	8	22	1759,94
[K].HQGLPQEVLNENLLR.[F]	1xDeamidated [N/Q]	5	8	22	1760,93
[K].HQGLPQEVLNENLLR.[F]	2xDeamidated [Q2; Q6]	5	8	22	1761,91
[K].DIGSESTEDQAMEDIK.[Q]	1xPhospho [S4]; 1xOxidation [M12]	6	43	58	1863,72
[K].YKVPQLEIVPNSAEER.[L]		4	104	119	1871,99
[K].DIGSESTEDQAMEDIK.[Q]	2xPhospho [S4; S6]	6	43	58	1927,69
[K].YKVPQLEIVPNSAEER.[L]	1xPhospho [S12]	4	104	119	1951,95
[K].YKVPQLEIVPNSAEER.[L]	1xDeamidated [Q5]; 1xPhospho [S12]	4	104	119	1952,94
[K].EPMIGVNQELAYFYPELFR.[Q]		6	133	151	2316,14
**Beta-casein OS=Bubalus bubalis**					
[K].FQSEEQQQMEDELQDK.[I]		1	33	48	2011,85
[K].FQSEEQQQMEDELQDK.[I]	1xOxidation [M9]	1	33	48	2027,85
[K].FQSEEQQQMEDELQDK.[I]	1xPhospho [S3]	1	33	48	2091,82
[K].FQSEEQQQMEDELQDK.[I]	1xDeamidated [Q]; 1xPhospho [S3]	1	33	48	2092,81
[K].FQSEEQQQMEDELQDK.[I]	2xDeamidated [Q]; 1xPhospho [S3]	1	33	48	2093,79
[K].FQSEEQQQMEDELQDK.[I]	4xDeamidated [Q2; Q14; Q]; 1xPhospho [S3]	1	33	48	2095,76
[K].FQSEEQQQMEDELQDK.[I]	1xPhospho [S3]; 1xOxidation [M9]	1	33	48	2107,82
[K].FQSEEQQQMEDELQDK.[I]	1xDeamidated [Q]; 1xPhospho [S3]; 1xOxidation [M9]	1	33	48	2108,80
[K].FQSEEQQQMEDELQDK.[I]	2xDeamidated [Q2; Q6]; 1xPhospho [S3]; 1xOxidation [M9]	1	33	48	2109,78
[KR].DMPIQAFLLYQEPVLGPVR.[G]		17	184	202	2186,17
[KR].DMPIQAFLLYQEPVLGPVR.[G]	1xDeamidated [Q]	17	184	202	2187,15
[KR].DMPIQAFLLYQEPVLGPVR.[G]	1xOxidation [M2]	17	184	202	2202,16
[KR].DMPIQAFLLYQEPVLGPVR.[G]	1xDeamidated [Q5]; 1xOxidation [M2]	17	184	202	2203,15
[K-].IHPFAQTQSLVYPFPGPIPK.[S]		3	49	68	2237,21
[K-].IHPFAQTQSLVYPFPGPIPK.[S]	1xDeamidated [Q]	3	49	68	2238,20
[K].IEKFQSEEQQQMEDELQDK.[I]	1xPhospho [S6]	1	30	48	2462,04
[K].IEKFQSEEQQQMEDELQDK.[I]	1xDeamidated [Q5]; 1xPhospho [S6]	1	30	48	2463,03
[K].SLPQNIPPLTQTPVVVPPFLQPEIMGVSK.[V]		8	69	97	3126,73
[K].SLPQNIPPLTQTPVVVPPFLQPEIMGVSK.[V]	1xDeamidated [Q/N]	8	69	97	3127,71
[-].RELEELNVPGEIVESLSSSEESITHINK.[K]	2xPhospho [S18; S19]	2	1	28	3298,51
[-].RELEELNVPGEIVESLSSSEESITHINK.[K]	4xPhospho [S15; S17; S18; S19]	2	1	28	3458,45
[-].RELEELNVPGEIVESLSSSEESITHINK.[K]	1xDeamidated [N7]; 4xPhospho [S15; S17; S18; S19]	2	1	28	3459,43
[-].RELEELNVPGEIVESLSSSEESITHINKK.[I]	4xPhospho [S15; S17; S18; S19]	1	1	29	3586,54
**Alpha s2-casein OS=Bubalus bubalis**					
[K].ALNEINQFYQK.[F]		8	81	91	1367,70
[K].ALNEINQFYQK.[F]	1xDeamidated [Q/N]	8	81	91	1368,68
[K].TVDMESTEVITK.[K]	1xPhospho [S6]	5	138	149	1432,63
[K].KTVDMESTEVITK.[K]	1xPhospho [T/S]	5	137	149	1560,72
[K].FPQYLQYLYQGPIVLNPWDQVK.[R]		7	92	113	2709,41
**Kappa-casein OS=Bubalus bubalis**					
[R].HPHPHLSFMAIPPK.[K]		23	98	111	1608,85
[R].SPAQILQWQVLPNTVPAK.[S]		5	69	86	1990,11
[R].YPSYGLNYYQQKPVALINNQFLPYPYYAKPAAVR.[S]		20	35	68	4010,06
**Kappa-casein (Fragment) OS=Bos taurus OX=9913 GN=CSN3 PE=4 SV=1**					
[R].HPHPHLSFMAIPPK.[K]		23	98	111	1608,85
[R].SPAQILQWQVLSNTVPAK.[S]		18	69	86	1980,09
[R].SPAQILQWQVLSNTVPAK.[S]	3xDeamidated [Q9; N13; Q]	18	69	86	1983,04
[R].YPSYGLNYYQQKPVALINNQFLPYPYYAKPAAVR.[S]		20	35	68	4010,06
**Alpha-lactalbumin protein variant D OS=Bos taurus**					
[K].VGINYWLAHK.[A]		7	99	108	1200,65
**Beta-lactoglobulin OS=Bubalus bubalis**					
[R].VYVEELKPTPEGDLEILLQK.[W]		8	41	60	2313,26

Sample IM23

Description	Modifications	# PSMs	Start	End	Theor. MH+ [Da]
**Alpha-S1-casein (Fragment) OS=Bubalus bubalis**					
[R].YLGYLEQLLR.[L]		10	91	100	1267,70
[R].FFVAPFPEVFGK.[E]		9	23	34	1384,73
[K].HQGLPQGVLNENLLR.[F]		5	8	22	1687,92
[K].HQGLPQGVLNENLLR.[F]	1xDeamidated [Q6]	5	8	22	1688,91
[K].HQGLPQGVLNENLLR.[F]	2xDeamidated [Q/N]	5	8	22	1689,89
[K].EPMIGVNQELAYFYPQLFR.[Q]		5	133	151	2315,15
[K].EPMIGVNQELAYFYPQLFR.[Q]	1xDeamidated [Q16]	5	133	151	2316,14
[K].YNVPQLEIVPNLAEEQLHSMK.[E]		5	104	124	2452,25
[K].YNVPQLEIVPNLAEEQLHSMK.[E]	1xDeamidated [Q/N]	5	104	124	2453,24
[K].YNVPQLEIVPNLAEEQLHSMK.[E]	1xOxidation [M20]	5	104	124	2468,25
[K].VNELSTDIGSESTEDQAMEDIK.[Q]	1xPhospho [S10]	4	37	58	2491,04
[K].VNELSTDIGSESTEDQAMEDIK.[Q]	2xPhospho [S/T]	4	37	58	2571,01
[K].KYNVPQLEIVPNLAEEQLHSMK.[E]		5	103	124	2580,35
[K].KYNVPQLEIVPNLAEEQLHSMK.[E]	1xDeamidated [N/Q]	5	103	124	2581,33
[K].VNELSTDIGSESTEDQAMEDIK.[Q]	2xPhospho [T/S]; 1xOxidation [M18]	4	37	58	2587,00
[K].KYNVPQLEIVPNLAEEQLHSMK.[E]	1xOxidation [M21]	5	103	124	2596,34
[-].DAYPSGAWYYVPLGTQYPDAPLFSDIPNPIGSENSGK.[T]		1	1	37	3986,88
**Alpha S1 casein OS=Bos taurus**					
[K].EPMIGVNQELAYFYPELFR.[Q]		6	133	151	2316,14
[R].FFVAPFPEVFGK.[E]		9	23	34	1384,73
[K].HQGLPQEVLNENLLR.[F]		5	8	22	1759,94
[K].HQGLPQEVLNENLLR.[F]	1xDeamidated [Q/N]	5	8	22	1760,93
[K].YKVPQLEIVPNSAEER.[L]	1xPhospho [S12]	4	104	119	1951,95
[K].YKVPQLEIVPNSAEER.[L]	1xDeamidated [Q5]; 1xPhospho [S12]	4	104	119	1952,94
[R].YLGYLEQLLR.[L]		10	91	100	1267,70
**Beta-casein OS=Bubalus bubalis**					
[K].FQSEEQQQMEDELQDK.[I]		1	33	48	2011,85
[K].FQSEEQQQMEDELQDK.[I]	1xDeamidated [Q14]	1	33	48	2012,84
[K].FQSEEQQQMEDELQDK.[I]	2xDeamidated [Q2; Q]	1	33	48	2013,82
[K].FQSEEQQQMEDELQDK.[I]	1xOxidation [M9]	1	33	48	2027,85
[K].FQSEEQQQMEDELQDK.[I]	1xPhospho [S3]	1	33	48	2091,82
[K].FQSEEQQQMEDELQDK.[I]	1xDeamidated [Q]; 1xPhospho [S3]	1	33	48	2092,81
[K].FQSEEQQQMEDELQDK.[I]	2xDeamidated [Q2; Q]; 1xPhospho [S3]	1	33	48	2093,79
[K].FQSEEQQQMEDELQDK.[I]	3xDeamidated [Q2; Q]; 1xPhospho [S3]	1	33	48	2094,77
[K].FQSEEQQQMEDELQDK.[I]	5xDeamidated [Q2; Q6; Q7; Q8; Q14]; 1xPhospho [S3]	1	33	48	2096,74
[K].FQSEEQQQMEDELQDK.[I]	1xPhospho [S3]; 1xOxidation [M9]	1	33	48	2107,82
[K].FQSEEQQQMEDELQDK.[I]	1xDeamidated [Q]; 1xPhospho [S3]; 1xOxidation [M9]	1	33	48	2108,80
[KR].DMPIQAFLLYQEPVLGPVR.[G]		17	184	202	2186,17
[KR].DMPIQAFLLYQEPVLGPVR.[G]	1xDeamidated [Q11]	17	184	202	2187,15
[KR].DMPIQAFLLYQEPVLGPVR.[G]	1xOxidation [M2]	17	184	202	2202,16
[K-].IHPFAQTQSLVYPFPGPIPK.[S]		3	49	68	2237,21
[K-].IHPFAQTQSLVYPFPGPIPK.[S]	1xDeamidated [Q8]	3	49	68	2238,20
[K].IEKFQSEEQQQMEDELQDK.[I]	1xPhospho [S6]	1	30	48	2462,04
[K].SLPQNIPPLTQTPVVVPPFLQPEIMGVSK.[V]		8	69	97	3126,73
[-].RELEELNVPGEIVESLSSSEESITHINK.[K]	3xPhospho [S17; S18; S19]	2	1	28	3378,48
[-].RELEELNVPGEIVESLSSSEESITHINK.[K]	4xPhospho [S15; S17; S18; S19]	2	1	28	3458,45
**Alpha s2-casein OS=Bubalus bubalis**					
[K].KTVDMESTEVITK.[K]	1xPhospho [S7]	5	137	149	1560,72
[K].TVDMESTEVITK.[K]	1xPhospho [S/T]	5	138	149	1432,63
[K].ALNEINQFYQK.[F]		8	81	91	1367,70
[K].ALNEINQFYQK.[F]	1xDeamidated [N/Q]	8	81	91	1368,68
[K].FPQYLQYLYQGPIVLNPWDQVK.[R]	1xDeamidated [N/Q]	7	92	113	2710,39
[K].TVDMESTEVITK.[K]		5	138	149	1352,66
**Kappa-casein OS=Bubalus bubalis GN=CSN3 PE=1 SV=2**					
[R].HPHPHLSFMAIPPK.[K]		23	98	111	1608,85
[R].SPAQILQWQVLPNTVPAK.[S]		5	69	86	1990,11
**Alpha-lactalbumin protein variant D OS=Bos taurus**					
[K].VGINYWLAHK.[A]		7	99	108	1200,65
**Beta-lactoglobulin OS=Bubalus bubalis**					
[R].TPEVDDEALEK.[F]		7	125	135	1245,58
[R].TPEVDDEALEKFDK.[A]		7	125	138	1635,77
[R].VYVEELKPTPEGDLEILLQK.[W]		8	41	60	2313,26

Sample IM24

Description	Modifications	# PSMs	Start	End	Theor. MH+ [Da]
**Alpha-S1-casein (Fragment) OS=Bubalus bubalis**					
[R].YLGYLEQLLR.[L]		10	91	100	1267,70
[R].FFVAPFPEVFGK.[E]		9	23	34	1384,73
[K].HQGLPQGVLNENLLR.[F]		5	8	22	1687,92
[K].HQGLPQGVLNENLLR.[F]	1xDeamidated [N/Q]	5	8	22	1688,91
[K].HQGLPQGVLNENLLR.[F]	2xDeamidated [Q/N]	5	8	22	1689,89
[K].EPMIGVNQELAYFYPQLFR.[Q]		5	133	151	2315,15
[K].EPMIGVNQELAYFYPQLFR.[Q]	1xDeamidated [N/Q]	5	133	151	2316,14
[K].EPMIGVNQELAYFYPQLFR.[Q]	2xDeamidated [N/Q]	5	133	151	2317,12
[K].EPMIGVNQELAYFYPQLFR.[Q]	1xDeamidated [Q16]; 1xOxidation [M3]	5	133	151	2332,13
[K].YNVPQLEIVPNLAEEQLHSMK.[E]		5	104	124	2452,25
[K].YNVPQLEIVPNLAEEQLHSMK.[E]	1xDeamidated [N/Q]	5	104	124	2453,24
[K].YNVPQLEIVPNLAEEQLHSMK.[E]	2xDeamidated [N2; Q5]	5	104	124	2454,22
[K].YNVPQLEIVPNLAEEQLHSMK.[E]	1xOxidation [M20]	5	104	124	2468,25
[K].YNVPQLEIVPNLAEEQLHSMK.[E]	1xDeamidated [N11]; 1xOxidation [M20]	5	104	124	2469,23
[K].VNELSTDIGSESTEDQAMEDIK.[Q]	1xPhospho [S10]	4	37	58	2491,04
[K].VNELSTDIGSESTEDQAMEDIK.[Q]	2xDeamidated [N2; Q16]; 1xPhospho [S10]	4	37	58	2493,01
[K].VNELSTDIGSESTEDQAMEDIK.[Q]	2xPhospho [S10; S/T]	4	37	58	2571,01
[K].KYNVPQLEIVPNLAEEQLHSMK.[E]		5	103	124	2580,35
[K].VNELSTDIGSESTEDQAMEDIK.[Q]	2xPhospho [S10; T13]; 1xOxidation [M18]	4	37	58	2587,00
[K].KYNVPQLEIVPNLAEEQLHSMK.[E]	1xOxidation [M21]	5	103	124	2596,34
[K].KYNVPQLEIVPNLAEEQLHSMK.[E]	1xDeamidated [N3]; 1xOxidation [M21]	5	103	124	2597,33
[K].VNELSTDIGSESTEDQAMEDIK.[Q]	3xPhospho [T/S]	4	37	58	2650,98
[K].VNELSTDIGSESTEDQAMEDIK.[Q]	3xPhospho [S10; S/T]; 1xOxidation [M18]	4	37	58	2666,97
[K].EKVNELSTDIGSESTEDQAMEDIK.[Q]	1xPhospho [S12]	4	35	58	2748,18
**Alpha S1 casein OS=Bos taurus**					
[R].YLGYLEQLLR.[L]		10	91	100	1267,70
[R].FFVAPFPEVFGK.[E]		9	23	34	1384,73
[K].VPQLEIVPNSAEER.[L]	1xPhospho [S10]	4	106	119	1660,79
[K].HQGLPQEVLNENLLR.[F]		5	8	22	1759,94
[K].EPMIGVNQELAYFYPELFR.[Q]	1xDeamidated [Q/N]	6	133	151	2317,12
[K].EPMIGVNQELAYFYPELFR.[Q]	1xOxidation [M3]	6	133	151	2332,13
**Beta-casein OS=Bubalus bubalis**					
[K].FQSEEQQQMEDELQDK.[I]		1	33	48	2011,85
[K].FQSEEQQQMEDELQDK.[I]	4xDeamidated [Q2; Q6; Q7; Q8]	1	33	48	2015,79
[K].FQSEEQQQMEDELQDK.[I]	1xOxidation [M9]	1	33	48	2027,85
[K].FQSEEQQQMEDELQDK.[I]	1xPhospho [S3]	1	33	48	2091,82
[K].FQSEEQQQMEDELQDK.[I]	1xDeamidated [Q]; 1xPhospho [S3]	1	33	48	2092,81
[K].FQSEEQQQMEDELQDK.[I]	2xDeamidated [Q2; Q6]; 1xPhospho [S3]	1	33	48	2093,79
[K].FQSEEQQQMEDELQDK.[I]	3xDeamidated [Q2; Q]; 1xPhospho [S3]	1	33	48	2094,77
[K].FQSEEQQQMEDELQDK.[I]	1xPhospho [S3]; 1xOxidation [M9]	1	33	48	2107,82
[K].FQSEEQQQMEDELQDK.[I]	1xDeamidated [Q]; 1xPhospho [S3]; 1xOxidation [M9]	1	33	48	2108,80
[K].FQSEEQQQMEDELQDK.[I]	2xDeamidated [Q2; Q]; 1xPhospho [S3]; 1xOxidation [M9]	1	33	48	2109,78
[K].FQSEEQQQMEDELQDK.[I]	3xDeamidated [Q2; Q]; 1xPhospho [S3]; 1xOxidation [M9]	1	33	48	2110,77
[KR].DMPIQAFLLYQEPVLGPVR.[G]		17	184	202	2186,17
[KR].DMPIQAFLLYQEPVLGPVR.[G]	1xOxidation [M2]	17	184	202	2202,16
[KR].DMPIQAFLLYQEPVLGPVR.[G]	1xDeamidated [Q5]; 1xOxidation [M2]	17	184	202	2203,15
[K-].IHPFAQTQSLVYPFPGPIPK.[S]		3	49	68	2237,21
[K-].IHPFAQTQSLVYPFPGPIPK.[S]	1xDeamidated [Q]	3	49	68	2238,20
[K].IEKFQSEEQQQMEDELQDK.[I]	1xPhospho [S6]	1	30	48	2462,04
[K].SLPQNIPPLTQTPVVVPPFLQPEIMGVSK.[V]		8	69	97	3126,73
[K].SLPQNIPPLTQTPVVVPPFLQPEIMGVSK.[V]	1xDeamidated [Q/N]	8	69	97	3127,71
[K].SLPQNIPPLTQTPVVVPPFLQPEIMGVSK.[V]	1xOxidation [M25]	8	69	97	3142,72
[K].SLPQNIPPLTQTPVVVPPFLQPEIMGVSK.[V]	1xDeamidated [N/Q]; 1xOxidation [M25]	8	69	97	3143,71
[-].RELEELNVPGEIVESLSSSEESITHINK.[K]	3xPhospho [S/T]	2	1	28	3378,48
[-].RELEELNVPGEIVESLSSSEESITHINKK.[I]	2xPhospho [S18; S19]	1	1	29	3426,61
[-].RELEELNVPGEIVESLSSSEESITHINK.[K]	4xPhospho [S15; S17; S18; S19]	2	1	28	3458,45
**AS2-casein (Fragment) OS=Bubalus bubalis**					
[K].ALNEINQFYQK.[F]		8	81	91	1367,70
[K].ALNEINQFYQK.[F]	1xDeamidated [N6]	8	81	91	1368,68
[K].FPQYLQYLYQGPIVLNPWDQVK.[R]		7	92	113	2709,41
[K].FPQYLQYLYQGPIVLNPWDQVK.[R]	1xDeamidated [N/Q]	7	92	113	2710,39
[K].TVDMESTEVITK.[K]		5	138	149	1352,66
[K].TVDMESTEVITK.[K]	1xPhospho [T/S]	5	138	149	1432,63
[K].KTVDMESTEVITK.[K]	1xPhospho [S/T]	5	137	149	1560,72
**Kappa-casein OS=Bubalus bubalis**					
[R].HPHPHLSFMAIPPK.[K]		23	98	111	1608,85
[R].SPAQILQWQVLPNTVPAK.[S]		5	69	86	1990,11
**Alpha-lactalbumin protein variant D OS=Bos taurus**					
[K].VGINYWLAHK.[A]		7	99	108	1200,65
**Beta-lactoglobulin OS=Bubalus bubalis**					
[R].VYVEELKPTPEGDLEILLQK.[W]		8	41	60	2313,26
[R].TPEVDDEALEK.[F]		7	125	135	1245,58

Sample IM25

Description	Modifications	# PSMs	Start	End	Theor. MH+ [Da]
**Alpha-S1-casein (Fragment) OS=Bubalus bubalis**					
[R].YLGYLEQLLR.[L]		1	91	100	1267,70
[R].FFVAPFPEVFGK.[E]		3	23	34	1384,73
[K].HQGLPQGVLNENLLR.[F]		1	8	22	1687,92
[K].HQGLPQGVLNENLLR.[F]	1xDeamidated [Q/N]	41	8	22	1688,91
[K].HQGLPQGVLNENLLR.[F]	2xDeamidated [Q/N]	2	8	22	1689,89
[K].HQGLPQGVLNENLLR.[F]	4xDeamidated [Q2; Q6; N10; N12]	1	8	22	1691,86
[K].EPMIGVNQELAYFYPQLFR.[Q]		2	133	151	2315,15
[K].YNVPQLEIVPNLAEEQLHSMK.[E]		1	104	124	2452,25
[K].YNVPQLEIVPNLAEEQLHSMK.[E]	2xDeamidated [N2; Q5]	1	104	124	2454,22
[K].YNVPQLEIVPNLAEEQLHSMK.[E]	1xOxidation [M20]	1	104	124	2468,25
[K].VNELSTDIGSESTEDQAMEDIK.[Q]	1xPhospho [S10]	1	37	58	2491,04
[K].VNELSTDIGSESTEDQAMEDIK.[Q]	2xPhospho [S/T]	18	37	58	2571,01
[K].KYNVPQLEIVPNLAEEQLHSMK.[E]		1	103	124	2580,35
[K].KYNVPQLEIVPNLAEEQLHSMK.[E]	2xDeamidated [N3; Q6]	1	103	124	2582,32
[K].VNELSTDIGSESTEDQAMEDIK.[Q]	2xPhospho [S10; T13]; 1xOxidation [M18]	1	37	58	2587,00
[K].KYNVPQLEIVPNLAEEQLHSMK.[E]	1xOxidation [M21]	1	103	124	2596,34
[K].KYNVPQLEIVPNLAEEQLHSMK.[E]	1xDeamidated [N/Q]; 1xOxidation [M21]	2	103	124	2597,33
[K].VNELSTDIGSESTEDQAMEDIK.[Q]	3xPhospho [T/S]	6	37	58	2650,98
[K].VNELSTDIGSESTEDQAMEDIK.[Q]	3xPhospho [S10; S/T]; 1xOxidation [M18]	2	37	58	2666,97
**Alpha S1 casein OS=Bos taurus**					
[R].FFVAPFPEVFGK.[E]		3	23	34	1384,73
[K].HQGLPQEVLNENLLR.[F]		1	8	22	1759,94
[R].YLGYLEQLLR.[L]		50	91	100	1267,70
**Beta-casein OS=Bubalus bubalis**					
[K].FQSEEQQQMEDELQDK.[I]		1	33	48	2011,85
[K].FQSEEQQQMEDELQDK.[I]	1xOxidation [M9]	1	33	48	2027,85
[K].FQSEEQQQMEDELQDK.[I]	1xPhospho [S3]	48	33	48	2091,82
[K].FQSEEQQQMEDELQDK.[I]	1xDeamidated [Q]; 1xPhospho [S3]	17	33	48	2092,81
[K].FQSEEQQQMEDELQDK.[I]	2xDeamidated [Q]; 1xPhospho [S3]	6	33	48	2093,79
[K].FQSEEQQQMEDELQDK.[I]	3xDeamidated [Q2; Q]; 1xPhospho [S3]	5	33	48	2094,77
[K].FQSEEQQQMEDELQDK.[I]	4xDeamidated [Q2; Q]; 1xPhospho [S3]	3	33	48	2095,76
[K].FQSEEQQQMEDELQDK.[I]	1xPhospho [S3]; 1xOxidation [M9]	8	33	48	2107,82
[K].FQSEEQQQMEDELQDK.[I]	1xDeamidated [Q]; 1xPhospho [S3]; 1xOxidation [M9]	3	33	48	2108,80
[K].FQSEEQQQMEDELQDK.[I]	2xDeamidated [Q2; Q]; 1xPhospho [S3]; 1xOxidation [M9]	3	33	48	2109,78
[KR].DMPIQAFLLYQEPVLGPVR.[G]		13	184	202	2186,17
[KR].DMPIQAFLLYQEPVLGPVR.[G]	1xDeamidated [Q11]	1	184	202	2187,15
[KR].DMPIQAFLLYQEPVLGPVR.[G]	1xOxidation [M2]	1	184	202	2202,16
[KR].DMPIQAFLLYQEPVLGPVR.[G]	1xDeamidated [Q]; 1xOxidation [M2]	2	184	202	2203,15
[K-].IHPFAQTQSLVYPFPGPIPK.[S]		11	49	68	2237,21
[K-].IHPFAQTQSLVYPFPGPIPK.[S]	1xDeamidated [Q]	2	49	68	2238,20
[K].IEKFQSEEQQQMEDELQDK.[I]	1xPhospho [S6]	2	30	48	2462,04
[K].IEKFQSEEQQQMEDELQDK.[I]	1xDeamidated [Q5]; 1xPhospho [S6]	1	30	48	2463,03
[K].IEKFQSEEQQQMEDELQDK.[I]	1xPhospho [S6]; 1xOxidation [M12]	1	30	48	2478,04
[K].SLPQNIPPLTQTPVVVPPFLQPEIMGVSK.[V]		16	69	97	3126,73
[K].SLPQNIPPLTQTPVVVPPFLQPEIMGVSK.[V]	1xDeamidated [N/Q]	3	69	97	3127,71
[K].SLPQNIPPLTQTPVVVPPFLQPEIMGVSK.[V]	1xOxidation [M25]	1	69	97	3142,72
[-].RELEELNVPGEIVESLSSSEESITHINK.[K]	1xPhospho [S18]	2	1	28	3218,55
[R].ELEELNVPGEIVESLSSSEESITHINK.[K]	4xPhospho [S/T]	2	2	28	3302,34
[-].RELEELNVPGEIVESLSSSEESITHINK.[K]	3xPhospho [S17; S18; S19]	2	1	28	3378,48
[-].RELEELNVPGEIVESLSSSEESITHINK.[K]	4xPhospho [S15; S17; S18; S19]	18	1	28	3458,45
[-].RELEELNVPGEIVESLSSSEESITHINK.[K]	1xDeamidated [N7]; 4xPhospho [S15; S17; S18; S19]	2	1	28	3459,43
[-].RELEELNVPGEIVESLSSSEESITHINKK.[I]	3xPhospho [S17; S18; S19]	1	1	29	3506,57
**Alpha s2-casein OS=Bubalus bubalis**					
[K].TVDMESTEVITK.[K]		1	138	149	1352,66
[K].ALNEINQFYQK.[F]		1	81	91	1367,70
[K].ALNEINQFYQK.[F]	1xDeamidated [N/Q]	4	81	91	1368,68
[K].ALNEINQFYQK.[F]	2xDeamidated [N/Q]	2	81	91	1369,66
[K].TVDMESTEVITK.[K]	1xPhospho [T/S]	2	138	149	1432,63
[K].KTVDMESTEVITK.[K]	1xPhospho [S7]	1	137	149	1560,72
[K].KTVDMESTEVITK.[K]	1xPhospho [S7]; 1xOxidation [M5]	1	137	149	1576,72
[K].FPQYLQYLYQGPIVLNPWDQVK.[R]		1	92	113	2709,41
**Kappa-casein OS=Bubalus bubalis**					
[R].HPHPHLSFMAIPPK.[K]		1	98	111	1608,85
[R].SPAQILQWQVLPNTVPAK.[S]		7	69	86	1990,11
[R].SPAQILQWQVLPNTVPAK.[S]	1xDeamidated [N13]	1	69	86	1991,10
[R].SPAQILQWQVLPNTVPAK.[S]	2xDeamidated [Q9; N13]	1	69	86	1992,08
[R].SPAQILQWQVLPNTVPAK.[S]	3xDeamidated [Q7; Q9; N13]	1	69	86	1993,06
[R].YPSYGLNYYQQKPVALINNQFLPYPYYAKPAAVR.[S]		1	35	68	4010,06
[R].YPSYGLNYYQQKPVALINNQFLPYPYYAKPAAVR.[S]	1xDeamidated [Q10]	1	35	68	4011,05
**Alpha-lactalbumin protein variant D OS=Bos taurus**					
[K].VGINYWLAHK.[A]		1	99	108	1200,65
**Beta-lactoglobulin OS=Bubalus bubalis**					
[R].TPEVDDEALEK.[F]		1	125	135	1245,58

Supplementary Material Table S4. Complete list of peptides identified by nano-LC-ESI-MS/MS in PDO MdBC cheeses labelled TU1 to TU25. Details provided include parent protein, peptide sequence, modification(s), intensity value, localization within protein sequence, and experimental mass value.

Sample TU1

Description	Modifications	# PSMs	Start	End	Theor. MH+ [Da]
**Alpha-S1-casein (Fragment) OS=Bubalus bubalis**					
[K].EPMIGVNQELAYFYPQLFR.[Q]		5	133	151	2315,15
[K].HQGLPQGVLNENLLR.[F]		5	8	22	1687,92
[K].HQGLPQGVLNENLLR.[F]	1xDeamidated [Q/N]	5	8	22	1688,91
[K].HQGLPQGVLNENLLR.[F]	2xDeamidated [Q2; N10]	5	8	22	1689,89
[K].KYNVPQLEIVPNLAEEQLHSMK.[E]		5	103	124	2580,35
[K].KYNVPQLEIVPNLAEEQLHSMK.[E]	1xDeamidated [N12]	5	103	124	2581,33
[K].VNELSTDIGSESTEDQAMEDIK.[Q]	1xPhospho [S10]; 1xOxidation [M18]	4	37	58	2507,04
[K].YNVPQLEIVPNLAEEQLHSMK.[E]		5	104	124	2452,25
[K].YNVPQLEIVPNLAEEQLHSMK.[E]	1xOxidation [M20]	5	104	124	2468,25
[R].FFVAPFPEVFGK.[E]		9	23	34	1384,73
[R].YLGYLEQLLR.[L]		12	91	100	1267,70
**Alpha s2-casein OS=Bubalus bubalis**					
[K].TVDMESTEVITK.[K]	1xPhospho [S/T]	5	123	134	1432,63
**Beta-casein OS=Bubalus bubalis GN=CSN2 PE=2 SV=1**					
[K].FQSEEQQQMEDELQDK.[I]		2	18	33	2011,85
[K].FQSEEQQQMEDELQDK.[I]	1xOxidation [M9]	2	18	33	2027,85
[K].FQSEEQQQMEDELQDK.[I]	1xPhospho [S3]	2	18	33	2091,82
[K].FQSEEQQQMEDELQDK.[I]	1xDeamidated [Q14]; 1xPhospho [S3]	2	18	33	2092,81
[K].FQSEEQQQMEDELQDK.[I]	5xDeamidated [Q2; Q6; Q7; Q8; Q14]; 1xPhospho [S3]	2	18	33	2096,74
[K].FQSEEQQQMEDELQDK.[I]	1xPhospho [S3]; 1xOxidation [M9]	2	18	33	2107,82
[K].FQSEEQQQMEDELQDK.[I]	1xDeamidated [Q2]; 1xPhospho [S3]; 1xOxidation [M9]	2	18	33	2108,80
[KR].DMPIQAFLLYQEPVLGPVR.[G]		20	169	187	2186,17
[KR].DMPIQAFLLYQEPVLGPVR.[G]	1xOxidation [M2]	20	169	187	2202,16
[K-].IHPFAQTQSLVYPFPGPIPK.[S]		4	34	53	2237,21
[K].IEKFQSEEQQQMEDELQDK.[I]	1xPhospho [S6]	2	15	33	2462,04
[K].SLPQNIPPLTQTPVVVPPFLQPEIMGVSK.[V]		9	54	82	3126,73
[-].RELEELNVPGEIVESLSSSEESITHINK.[K]	4xPhospho [S15; S17; S18; S19]	2	1	28	3458,45
**Kappa-casein OS=Bubalus bubalis**					
[R].SPAQILQWQVLPNTVPAK.[FS]		15	75	92	1990,11
[R].YPSYGLNYYQQKPVALINNQFLPYPYYAKPAAVR.[S]		22	41	74	4010,06
[R].YPSYGLNYYQQKPVALINNQFLPYPYYAKPAAVR.[S]	1xDeamidated [N/Q]	22	41	74	4011,05
**Alpha-lactalbumin protein variant D OS=Bos taurus OX=9913 GN=LALBA PE=3 SV=1**					
[K].VGINYWLAHK.[A]		10	99	108	1200,65
**Beta-lactoglobulin OS=Bubalus bubalis GN=LGB PE=1 SV=2**					
[R].TPEVDDEALEKFDK.[A]		7	125	138	1635,77

Sample TU2

Description	Modifications	# PSMs	Start	End	Theor. MH+ [Da]
**Alpha-S1-casein (Fragment) OS=Bubalus bubalis GN=CSN1S PE=2 SV=1**					
[K].HQGLPQGVLNENLLR.[F]		17	8	22	1687,92
[K].HQGLPQGVLNENLLR.[F]	1xDeamidated [Q/N]	7	8	22	1688,91
[R].FFVAPFPEVFGK.[E]		12	23	34	1384,73
[K].VNELSTDIGSESTEDQAMEDIK.[Q]	1xPhospho [S10]	1	37	58	2491,04
[K].VNELSTDIGSESTEDQAMEDIK.[Q]	1xPhospho [S10]; 1xOxidation [M18]	1	37	58	2507,04
[K].VNELSTDIGSESTEDQAMEDIK.[Q]	2xPhospho [S/T]; 1xOxidation [M18]	5	37	58	2587,00
[R].YLGYLEQLLR.[L]		54	91	100	1267,70
[K].KYNVPQLEIVPNLAEEQLHSMK.[E]		1	103	124	2580,35
[K].KYNVPQLEIVPNLAEEQLHSMK.[E]	1xDeamidated [N/Q]	2	103	124	2581,33
[K].YNVPQLEIVPNLAEEQLHSMK.[E]		1	104	124	2452,25
[K].YNVPQLEIVPNLAEEQLHSMK.[E]	1xOxidation [M20]	1	104	124	2468,25
[K].EPMIGVNQELAYFYPQLFR.[Q]		3	133	151	2315,15
[K].EPMIGVNQELAYFYPQLFR.[Q]	1xOxidation [M3]	1	133	151	2331,15
**AS2-casein (Fragment) OS=Bubalus bubalis PE=2 SV=2**					
[K].ALNEINQFYQK.[F]		2	81	91	1367,70
[K].ALNEINQFYQK.[F]	1xDeamidated [N6]	1	81	91	1368,68
[K].FPQYLQYLYQGPIVLNPWDQVK.[R]		1	92	113	2709,41
[K].TVDMESTEVITK.[K]	1xPhospho [T/S]	2	126	137	1432,63
**Beta-casein OS=Bubalus bubalis GN=CSN2 PE=2 SV=1**					
[-].RELEELNVPGEIVESLSSSEESITHINK.[K]	4xPhospho [S15; S17; S18; S19]	12	1	28	3458,45
[K].IEKFQSEEQQQMEDELQDK.[I]	1xPhospho [S6]	2	15	33	2462,04
[K].FQSEEQQQMEDELQDK.[I]		1	18	33	2011,85
[K].FQSEEQQQMEDELQDK.[I]	1xOxidation [M9]	1	18	33	2027,85
[K].FQSEEQQQMEDELQDK.[I]	1xDeamidated [Q2]; 1xOxidation [M9]	1	18	33	2028,83
[K].FQSEEQQQMEDELQDK.[I]	1xPhospho [S3]	3	18	33	2091,82
[K].FQSEEQQQMEDELQDK.[I]	1xDeamidated [Q14]; 1xPhospho [S3]	1	18	33	2092,81
[K].FQSEEQQQMEDELQDK.[I]	2xDeamidated [Q2; Q14]; 1xPhospho [S3]	1	18	33	2093,79
[K].FQSEEQQQMEDELQDK.[I]	1xPhospho [S3]; 1xOxidation [M9]	2	18	33	2107,82
[K].FQSEEQQQMEDELQDK.[I]	1xDeamidated [Q]; 1xPhospho [S3]; 1xOxidation [M9]	3	18	33	2108,80
[K].FQSEEQQQMEDELQDK.[I]	3xDeamidated [Q2; Q8; Q]; 1xPhospho [S3]; 1xOxidation [M9]	2	18	33	2110,77
[K-].IHPFAQTQSLVYPFPGPIPK.[S]		5	34	53	2237,21
[K].SLPQNIPPLTQTPVVVPPFLQPEIMGVSK.[V]		13	54	82	3126,73
[KR].DMPIQAFLLYQEPVLGPVR.[G]		3	169	187	2186,17
[KR].DMPIQAFLLYQEPVLGPVR.[G]	1xDeamidated [Q]	2	169	187	2187,15
[KR].DMPIQAFLLYQEPVLGPVR.[G]	1xOxidation [M2]	1	169	187	2202,16
**Kappa-casein OS=Bubalus bubalis OX=89462 GN=CSN3 PE=1 SV=2**					
[R].YPSYGLNYYQQKPVALINNQFLPYPYYAKPAAVR.[S]		1	41	74	4010,06
[R].YPSYGLNYYQQKPVALINNQFLPYPYYAKPAAVR.[S]	1xDeamidated [N/Q]	2	41	74	4011,05
[R].SPAQILQWQVLPNTVPAK.[FS]		1	75	92	1990,11
[R].SPAQILQWQVLPNTVPAK.[FS]	1xDeamidated [N13]	1	75	92	1991,10
**Beta-lactoglobulin OS=Bubalus bubalis GN=LGB PE=1 SV=2**					
[R].VYVEELKPTPEGDLEILLQK.[W]		1	41	60	2313,26
[R].TPEVDDEALEKFDK.[A]		1	125	138	1635,77
**Alpha-lactalbumin protein variant D OS=Bos taurus OX=9913 GN=LALBA PE=3 SV=1**					
[K].VGINYWLAHK.[A]		1	99	108	1200,65

Sample TU3

Description	Modifications	PSMs	Start	End	Theor. MH+ [Da]
**Alpha-S1-casein (Fragment) OS=Bubalus bubalis GN=CSN1S PE=2 SV=1**					
[R].YLGYLEQLLR.[L]		12	91	100	1267,70
[R].FFVAPFPEVFGK.[E]		9	23	34	1384,73
[K].HQGLPQGVLNENLLR.[F]		5	8	22	1687,92
[K].HQGLPQGVLNENLLR.[F]	1xDeamidated [Q/N]	5	8	22	1688,91
[K].HQGLPQGVLNENLLR.[F]	3xDeamidated [Q2; Q6; N]	5	8	22	1690,88
[K].EPMIGVNQELAYFYPQLFR.[Q]		5	133	151	2315,15
[K].YNVPQLEIVPNLAEEQLHSMK.[E]		5	104	124	2452,25
[K].YNVPQLEIVPNLAEEQLHSMK.[E]	1xOxidation [M20]	5	104	124	2468,25
[K].VNELSTDIGSESTEDQAMEDIK.[Q]	1xPhospho [S]	4	37	58	2491,04
[K].VNELSTDIGSESTEDQAMEDIK.[Q]	1xPhospho [S/T]; 1xOxidation [M18]	4	37	58	2507,04
[K].VNELSTDIGSESTEDQAMEDIK.[Q]	2xPhospho [S10; T/S]	4	37	58	2571,01
[K].KYNVPQLEIVPNLAEEQLHSMK.[E]		5	103	124	2580,35
[K].VNELSKDIGSESTEDQAMEDIK.[Q]	2xPhospho [S10; T/S]	6	37	58	2598,06
[K].VNELSKDIGSESTEDQAMEDIK.[Q]	2xPhospho [S10; T13]; 1xOxidation [M18]	6	37	58	2614,05
[K].EKVNELSTDIGSESTEDQAMEDIK.[Q]	1xPhospho [S12]; 1xOxidation [M20]	4	35	58	2764,18
**Alpha s2-casein OS=Bubalus bubalis PE=2 SV=1**					
[K].KTVDMESTEVITK.[K]	1xPhospho [T/S]	5	122	134	1560,72
[K].TVDMESTEVITK.[K]		5	123	134	1352,66
[K].TVDMESTEVITK.[K]	1xPhospho [S6]	5	123	134	1432,63
**Beta-casein OS=Bubalus bubalis GN=CSN2 PE=2 SV=1**					
[K].FQSEEQQQMEDELQDK.[I]		2	18	33	2011,85
[K].FQSEEQQQMEDELQDK.[I]	1xOxidation [M9]	2	18	33	2027,85
[K].FQSEEQQQMEDELQDK.[I]	1xPhospho [S3]	2	18	33	2091,82
[K].FQSEEQQQMEDELQDK.[I]	1xDeamidated [Q14]; 1xPhospho [S3]	2	18	33	2092,81
[K].FQSEEQQQMEDELQDK.[I]	1xPhospho [S3]; 1xOxidation [M9]	2	18	33	2107,82
[KR].DMPIQAFLLYQEPVLGPVR.[G]		20	169	187	2186,17
[KR].DMPIQAFLLYQEPVLGPVR.[G]	1xDeamidated [Q5]; 1xOxidation [M2]	20	169	187	2203,15
[K-].IHPFAQTQSLVYPFPGPIPK.[S]		4	34	53	2237,21
[K-].IHPFAQTQSLVYPFPGPIPK.[S]	1xDeamidated [Q6]	4	34	53	2238,20
[K].IEKFQSEEQQQMEDELQDK.[I]	1xPhospho [S6]	2	15	33	2462,04
[K].IEKFQSEEQQQMEDELQDK.[I]	1xPhospho [S6]; 1xOxidation [M12]	2	15	33	2478,04
[K].SLPQNIPPLTQTPVVVPPFLQPEIMGVSK.[V]		9	54	82	3126,73
[R].ELEELNVPGEIVESLSSSEESITHINK.[K]	4xPhospho [S/T]	2	1	28	3302,34
[-].RELEELNVPGEIVESLSSSEESITHINK.[K]	4xPhospho [S15; S17; S18; S19]	2	1	28	3458,45
**Kappa-casein OS=Bubalus bubalis OX=89462 GN=CSN3 PE=1 SV=2**					
[R].HPHPHLSFMAIPPK.[K]		36	98	111	1608,85
[R].SPAQILQWQVLPNTVPAK.[FS]		15	69	86	1990,11
[R].SPAQILQWQVLPNTVPAK.[FS]	1xDeamidated [N13]	15	69	86	1991,10
[R].SPAQILQWQVLPNTVPAK.[FS]	2xDeamidated [Q/N]	15	69	86	1992,08
[R].YPSYGLNYYQQKPVALINNQFLPYPYYAKPAAVR.[S]		22	35	68	4010,06
[R].YPSYGLNYYQQKPVALINNQFLPYPYYAKPAAVR.[S]	1xDeamidated [Q/N]	22	35	68	4011,05
**Beta-lactoglobulin-1/B OS=Ovis aries OX=9940 PE=1 SV=1/Water Buffalo**					
[R].TPEVDNEALEK.[F] Water Buffalo	1xDeamidated [N6]	2	125	135	1245,58
[R].TPEVDNEALEKFDK.[A] Ovine A/B		2	125	138	1634,79
[R].TPEVDDEALEKFDK.[A] Water Buffalo		7	125	138	1635,77
[R].VYVEELKPTPEGDLEILLQK.[W] Water Buffalo		8	41	60	2313,26
[K-].VAGTWYSLAMAASDISLLDAQSAPLR.[V] Water Buffalo/ovine B		9	15	40	2707,38
**Alpha-lactalbumin protein variant D Bos taurus / Water Buffalo**					
[K].VGINYWLAHK.[A]		10	99	108	1200,65

Sample TU4

Description	Modifications	# PSMs	Start	End	Theor. MH+ [Da]
**Alpha-S1-casein (Fragment) OS=Bubalus bubalis GN=CSN1S PE=2 SV=1**					
[K].VNELSTDIGSESTEDQAMEDIK.[Q]	1xPhospho [S10]	1	37	58	2491,04
**Alpha s2-casein OS=Bubalus bubalis PE=2 SV=1**					
[K].KTVDMESTEVITK.[K]	1xPhospho [T/S]	2	122	134	1560,72
[K].KTVDMESTEVITK.[K]	1xPhospho [S7]; 1xOxidation [M5]	1	122	134	1576,72
[K].TVDMESTEVITK.[K]	1xPhospho [S/T]	2	123	134	1432,63
**Beta-casein OS=Bubalus bubalis GN=CSN2 PE=2 SV=1**					
[-].RELEELNVPGEIVESLSSSEESITHINK.[K]	4xPhospho [S15; S17; S18; S19]	8	1	28	3458,45
[K].IEKFQSEEQQQMEDELQDK.[I]	1xPhospho [S6]	1	15	33	2462,04
[K].IEKFQSEEQQQMEDELQDK.[I]	1xDeamidated [Q]; 1xPhospho [S6]	3	15	33	2463,03
[K].FQSEEQQQMEDELQDK.[I]	1xOxidation [M9]	1	18	33	2027,85
[K].FQSEEQRQTEDELQDK.[I]	1xDeamidated [Q8]; 1xPhospho [S3]	2	18	33	2090,86
[K].FQSEEQQQMEDELQDK.[I]	1xPhospho [S3]	12	18	33	2091,82
[K].FQSEEQQQMEDELQDK.[I]	1xDeamidated [Q]; 1xPhospho [S3]	8	18	33	2092,81
[K].FQSEEQQQMEDELQDK.[I]	2xDeamidated [Q]; 1xPhospho [S3]	10	18	33	2093,79
[K].FQSEEQQQMEDELQDK.[I]	1xPhospho [S3]; 1xOxidation [M9]	6	18	33	2107,82
[K].FQSEEQQQMEDELQDK.[I]	1xDeamidated [Q]; 1xPhospho [S3]; 1xOxidation [M9]	4	18	33	2108,80

Sample TU5

Description	Modifications	# PSMs	Start	End	Theor. MH+ [Da]
**Alpha-S1-casein (Fragment) OS=Bubalus bubalis GN=CSN1S PE=2 SV=1**					
[K].VNELSTDIGSESTEDQAMEDIK.[Q]	2xPhospho [S10; T13]; 1xOxidation [M18]	1	37	58	2587,00
Alpha s2-casein OS=Bubalus bubalis PE=2 SV=1					
[K].ALNEINQFYQK.[F]		3	69	79	1367,70
[K].ALNEINQFYQK.[F]	1xDeamidated [N]	2	69	79	1368,68
[K].FPQYLQYLYQGPIVLNPWDQVK.[R]		2	80	101	2709,41
[K].KTVDMESTEVITK.[K]	1xPhospho [S7]; 1xOxidation [M5]	1	122	134	1576,72
[K].TVDMESTEVITK.[K]	1xPhospho [T7]	1	126	137	1432,63
**Beta-casein OS=Bubalus bubalis GN=CSN2 PE=2 SV=1**					
[-].RELEELNVPGEIVESLSSSEESITHINK.[K]	4xPhospho [S15; S17; S18; S19]	2	1	28	3458,45
[K].FQSEEQQQMEDELQDK.[I]	1xPhospho [S3]	11	18	33	2091,82
[K].FQSEEQQQMEDELQDK.[I]	1xPhospho [S3]; 1xOxidation [M9]	4	18	33	2107,82
[K].FQSEEQQQMEDELQDK.[I]	1xDeamidated [Q]; 1xPhospho [S3]	6	18	33	2092,81
[K].FQSEEQQQMEDELQDK.[I]	2xDeamidated [Q]; 1xPhospho [S3]	4	18	33	2093,79
[K].FQSEEQQQMEDELQDK.[I]	2xDeamidated [Q2; Q]; 1xPhospho [S3]; 1xOxidation [M9]	2	18	33	2109,78
[K].IEKFQSEEQQQMEDELQDK.[I]	1xPhospho [S6]	1	15	33	2462,04
**Kappa-casein OS=Bubalus bubalis OX=89462 GN=CSN3 PE=1 SV=2**					
[R].SPAQILQWQVLPNTVPAK.[FS]		1	69	86	1990,11
[R].YPSYGLNYYQQKPVALINNQFLPYPYYAKPAAVR.[S]		1	35	68	4010,06
**Beta-lactoglobulin variant D (Fragment) OS=Bos taurus OX=9913 GN=LGB PE=3 SV=1**					
[R].VYVEQLKPTPEGDLEILLQK.[W]		1	43	62	2312,27
[R].VYVEQLKPTPEGDLEILLQK.[W]	1xDeamidated [Q5]	2	41	60	2313,26
**Beta-lactoglobulin OS=Bubalus bubalis GN=LGB PE=1 SV=2**					
[R].VYVEELKPTPEGDLEILLQK.[W]		2	41	60	2313,26
**Alpha-lactalbumin protein variant D OS=Bos taurus OX=9913 GN=LALBA PE=3 SV=1**					
[K].VGINYWLAHK.[A]		1	99	108	1200,65

Sample TU6

Description	Modifications	# PSMs	Start	End	Theor. MH+ [Da]
**Alpha-S1-casein (Fragment) OS=Bubalus bubalis GN=CSN1S PE=2 SV=1**					
[K].HQGLPQGVLNENLLR.[F]		23	8	22	1687,92
[K].HQGLPQGVLNENLLR.[F]	1xDeamidated [Q/N]	16	8	22	1688,91
[K].HQGLPQEVLNENLLR.[F]		1	8	22	1759,94
[R].FFVAPFPEVFGK.[E]		3	23	34	1384,73
[K].VNELSTDIGSESTEDQAMEDIK.[Q]	1xPhospho [S10]	1	37	58	2491,04
[K].VNELSTDIGSESTEDQAMEDIK.[Q]	1xPhospho [S12]; 1xOxidation [M18]	1	37	58	2507,04
[K].VNELSTDIGSESTEDQAMEDIK.[Q]	2xPhospho [S10; T13]	1	37	58	2571,01
[K].VNELSTDIGSESTEDQAMEDIK.[Q]	2xPhospho [S/T]; 1xOxidation [M18]	4	37	58	2587,00
[K].VNELSKDIGSESTEDQAMEDIK.[Q]	2xPhospho [S10; T13]; 1xOxidation [M18]	1	37	58	2614,05
[K].VNELSTDIGSESTEDQAMEDIK.[Q]	3xPhospho [S10; S12; T/S]	2	37	58	2650,98
[R].YLGYLEQLLR.[L]		57	91	100	1267,70
[K].KYNVPQLEIVPNLAEEQLHSMK.[E]		2	103	124	2580,35
[K].KYNVPQLEIVPNLAEEQLHSMK.[E]	1xOxidation [M21]	1	103	124	2596,34
[K].KYNVPQLEIVPNLAEEQLHSMK.[E]	1xDeamidated [Q6]; 1xOxidation [M21]	1	103	124	2597,33
[K].YNVPQLEIVPNLAEEQLHSMK.[E]		3	104	124	2452,25
[K].YNVPQLEIVPNLAEEQLHSMK.[E]	1xOxidation [M20]	2	104	124	2468,25
[K].EPMIGVNQELAYFYPQLFR.[Q]		6	133	151	2315,15
[K].EPMIGVNQELAYFYPQLFR.[Q]	1xDeamidated [N7]	1	133	151	2316,14
**Beta-casein OS=Bubalus bubalis GN=CSN2 PE=2 SV=1**					
[K].FQSEEQQQMEDELQDK.[I]		2	18	33	2011,85
[K].FQSEEQQQMEDELQDK.[I]	1xOxidation [M9]	1	18	33	2027,85
[K].FQSEEQQQTEDELQDK.[I]	1xPhospho [S3]	1	33	48	2061,83
[K].FQSEEQQQMEDELQDK.[I]	1xPhospho [S3]	2	18	33	2091,82
[K].FQSEEQQQMEDELQDK.[I]	1xDeamidated [Q]; 1xPhospho [S3]	3	18	33	2092,81
[K].FQSEEQQQMEDELQDK.[I]	2xDeamidated [Q]; 1xPhospho [S3]	6	18	33	2093,79
[K].FQSEEQQQMEDELQDK.[I]	3xDeamidated [Q]; 1xPhospho [S3]	6	18	33	2094,77
[K].FQSEEQQQMEDELQDK.[I]	1xPhospho [S3]; 1xOxidation [M9]	1	18	33	2107,82
[K].FQSEEQQQMEDELQDK.[I]	1xDeamidated [Q]; 1xPhospho [S3]; 1xOxidation [M9]	3	18	33	2108,80
[KR].DMPIQAFLLYQEPVLGPVR.[G]		23	182	200	2186,17
[KR].DMPIQAFLLYQEPVLGPVR.[G]	1xDeamidated [Q5]	1	182	200	2187,15
[KR].DMPIQAFLLYQEPVLGPVR.[G]	1xOxidation [M2]	2	182	200	2202,16
[KR].DMPIQAFLLYQEPVLGPVR.[G]	1xDeamidated [Q5]; 1xOxidation [M2]	1	182	200	2203,15
[K-].IHPFAQTQSLVYPFPGPIPK.[S]		6	34	53	2237,21
[K].IEKFQSEEQQQMEDELQDK.[I]	1xPhospho [S6]	1	15	33	2462,04
[K].IEKFQSEEQQQMEDELQDK.[I]	1xDeamidated [Q11]; 1xPhospho [S6]	1	15	33	2463,03
[K].SLPQNIPPLTQTPVVVPPFLQPEIMGVSK.[V]		11	54	82	3126,73
[K].SLPQNIPPLTQTPVVVPPFLQPEIMGVSK.[V]	1xDeamidated [N/Q]	2	54	82	3127,71
[-].RELEELNVPGEIVESLSSSEESITHINK.[K]	4xPhospho [S15; S17; S18; S19]	18	1	28	3458,45
[-].RELEELNVPGEIVESLSSSEESITHINKK.[I]	3xPhospho [S15; S17; S18]	1	1	28	3506,57
**Alpha s2-casein OS=Bubalus bubalis PE=2 SV=1**					
[K].KTVDMESTEVITK.[K]	1xPhospho [S7]; 1xOxidation [M5]	1	122	134	1576,72
[K].KTVDMESTEVITK.[K]	1xPhospho [T8]	1	122	134	1560,72
**Kappa-casein OS=Bubalus bubalis OX=89462 GN=CSN3 PE=1 SV=2**					
[R].SPAQILQWQVLPNTVPAK.[FS]		1	69	86	1990,11
[R].YPSYGLNYYQQKPVALINNQFLPYPYYAKPAAVR.[S]		1	35	68	4010,06
**Beta-lactoglobulin OS=Bubalus bubalis GN=LGB PE=1 SV=2**					
[R].TPEVDDEALEK.[F]		1	125	135	1245,58
**Beta-lactoglobulin OS=Capra hircus OX=9925 GN=LGB PE=1 SV=2**					
[R].VYVEELKPTPEGNLEILLQK.[W]		1	41	60	2312,27
**Alpha-lactalbumin protein variant D OS=Bos taurus OX=9913 GN=LALBA PE=3 SV=1**					
[K].VGINYWLAHK.[A]		1	99	108	1200,65

Sample TU7

Description	Modifications	# PSMs	Start	End	Theor. MH+ [Da]
**Alpha S1 casein OS=Bos taurus OX=9913 GN=CSN1S1 PE=2 SV=1**					
[K].HQGLPQGVLNENLLR.[F]		19	8	22	1687,92
[K].HQGLPQGVLNENLLR.[F]	1xDeamidated [Q/N]	5	8	22	1688,91
[R].FFVAPFPEVFGK.[E]		6	23	34	1384,73
[K].VNELSKDIGSESTEDQAMEDIK.[Q]	2xPhospho [S10; T13]; 1xOxidation [M18]	1	37	58	2614,05
[K].VNELSTDIGSESTEDQAMEDIK.[Q]	1xPhospho [S10]	1	37	58	2491,04
[K].VNELSTDIGSESTEDQAMEDIK.[Q]	1xPhospho [S12]; 1xOxidation [M18]	1	37	58	2507,04
[K].VNELSTDIGSESTEDQAMEDIK.[Q]	2xPhospho [S10; T13]	1	37	58	2571,01
[K].VNELSTDIGSESTEDQAMEDIK.[Q]	2xPhospho [S10; T13]; 1xOxidation [M18]	1	37	58	2587,00
[R].YLGYLEQLLR.[L]		57	91	100	1267,70
[K].KYNVPQLEIVPNLAEEQLHSMK.[E]		2	103	124	2580,35
[K].KYNVPQLEIVPNLAEEQLHSMK.[E]	1xDeamidated [N12]	1	103	124	2581,33
[K].KYNVPQLEIVPNLAEEQLHSMK.[E]	1xOxidation [M21]	1	103	124	2596,34
[K].YNVPQLEIVPNLAEEQLHSMK.[E]		2	104	124	2452,25
[K].YNVPQLEIVPNLAEEQLHSMK.[E]	1xDeamidated [Q16]	1	104	124	2453,24
[K].YNVPQLEIVPNLAEEQLHSMK.[E]	1xOxidation [M20]	1	104	124	2468,25
[K].EPMIGVNQELAYFYPQLFR.[Q]		12	133	151	2315,15
[K].EPMIGVNQELAYFYPQLFR.[Q]	1xDeamidated [N7]	1	133	151	2316,14
[K].EPMIGVNQELAYFYPQLFR.[Q]	1xOxidation [M3]	1	133	151	2331,15
**Alpha s2-casein OS=Bubalus bubalis PE=2 SV=1**					
[K].FPQYLQYLYQGPIVLNPWDQVK.[R]		6	92	113	2709,41
[K].KTVDMESTEVITK.[K]	1xPhospho [T/S]	2	122	134	1560,72
[K].KTVDMESTEVITK.[K]	1xPhospho [S7]; 1xOxidation [M5]	1	122	134	1576,72
**Beta-casein OS=Bubalus bubalis GN=CSN2 PE=2 SV=1**					
[K].FQSEEQQQMEDELQDK.[I]	1xOxidation [M9]	1	18	33	2027,85
[K].FQSEEQRQTEDELQDK.[I]	1xDeamidated [Q8]; 1xPhospho [S3]	2	18	33	2090,86
[K].FQSEEQQQMEDELQDK.[I]	1xPhospho [S3]	32	18	33	2091,82
[K].FQSEEQQQMEDELQDK.[I]	1xDeamidated [Q2]; 1xPhospho [S3]	1	18	33	2092,81
[K].FQSEEQQQMEDELQDK.[I]	2xDeamidated [Q]; 1xPhospho [S3]	10	18	33	2093,79
[K].FQSEEQQQMEDELQDK.[I]	3xDeamidated [Q2; Q6; Q14]; 1xPhospho [S3]	1	18	33	2094,77
[K].FQSEEQQQMEDELQDK.[I]	1xPhospho [S3]; 1xOxidation [M9]	7	18	33	2107,82
[K].FQSEEQQQMEDELQDK.[I]	1xDeamidated [Q]; 1xPhospho [S3]; 1xOxidation [M9]	4	18	33	2108,80
[KR].DMPIQAFLLYQEPVLGPVR.[G]		33	169	187	2186,17
[KR].DMPIQAFLLYQEPVLGPVR.[G]	1xDeamidated [Q]	5	169	187	2187,15
[K-].IHPFAQTQSLVYPFPGPIPK.[S]		9	34	53	2237,21
[K].IEKFQSEEQQQMEDELQDK.[I]	1xPhospho [S6]	1	15	33	2462,04
[K].IEKFQSEEQQQMEDELQDK.[I]	1xDeamidated [Q]; 1xPhospho [S6]	4	15	33	2463,03
[K].SLPQNIPPLTQTPVVVPPFLQPEIMGVSK.[V]		8	54	82	3126,73
[K].SLPQNIPPLTQTPVVVPPFLQPEIMGVSK.[V]	1xDeamidated [Q/N]	2	54	82	3127,71
[-].RELEELNVPGEIVESLSSSEESITHINK.[K]	4xPhospho [S15; S/T]	18	1	28	3458,45
[-].RELEELNVPGEIVESLSSSEESITHINK.[K]	1xDeamidated [N27]; 4xPhospho [S15; S19; S22; T24]	2	1	28	3459,43
**Kappa-casein OS=Bubalus bubalis OX=89462 GN=CSN3 PE=1 SV=2**					
[R].SPAQILQWQVLPNTVPAK.[FS]		2	69	86	1990,11
[R].SPAQILQWQVLPNTVPAK.[FS]	2xDeamidated [Q9; N13]	1	69	86	1992,08
[R].YPSYGLNYYQQKPVALINNQFLPYPYYAKPAAVR.[S]		1	35	68	4010,06
[R].YPSYGLNYYQQKPVALINNQFLPYPYYAKPAAVR.[S]	1xDeamidated [N/Q]	4	35	68	4011,05
**Beta-lactoglobulin OS=Bubalus bubalis GN=LGB PE=1 SV=2**					
[K-].VAGTWYSLAMAASDISLLDAQSAPLR.[V]		1	15	40	2707,38
[R].VYVEELKPTPEGDLEILLQK.[W]		2	41	60	2313,26
**Beta-lactoglobulin variant D (Fragment) OS=Bos taurus OX=9913 GN=LGB PE=3 SV=1**					
[K-].VAGTWYSLAMAASDISLLDAQSAPLR.[V]		1	15	40	2707,38
[R].VYVEQLKPTPEGDLEILLQK.[W]		1	9	28	2312,27
[R].VYVEQLKPTPEGDLEILLQK.[W]	1xDeamidated [Q5]	2	9	28	2313,26
**Alpha-lactalbumin protein variant D OS=Bos taurus OX=9913 GN=LALBA PE=3 SV=1**					
[K].VGINYWLAHK.[A]		1	99	108	1200,65

Sample TU8

Description	Modifications	# PSMs	Start	End	Theor. MH+ [Da]
**Alpha-S1-casein (Fragment) OS=Bubalus bubalis GN=CSN1S PE=2 SV=1**					
[K].HQGLPQGVLNENLLR.[F]		22	8	22	1687,92
[K].HQGLPQGVLNENLLR.[F]	1xDeamidated [Q/N]	3	8	22	1688,91
[K].HQGLPQGVLNENLLR.[F]	3xDeamidated [Q2; Q6; N]	4	8	22	1690,88
[R].FFVAPFPEVFGK.[E]		9	23	34	1384,73
[K].VNELSTDIGSESTEDQAMEDIK.[Q]	1xPhospho [S10]	1	37	58	2491,04
[K].VNELSTDIGSESTEDQAMEDIK.[Q]	1xPhospho [S12]; 1xOxidation [M18]	1	37	58	2507,04
[K].VNELSTDIGSESTEDQAMEDIK.[Q]	2xPhospho [S/T]	4	37	58	2571,01
[R].YLGYLEQLLR.[L]		60	91	100	1267,70
[K].KYNVPQLEIVPNLAEEQLHSMK.[E]		3	103	124	2580,35
[K].YNVPQLEIVPNLAEEQLHSMK.[E]		2	104	124	2452,25
[K].YNVPQLEIVPNLAEEQLHSMK.[E]	1xOxidation [M20]	1	104	124	2468,25
[K].EPMIGVNQELAYFYPQLFR.[Q]		20	133	151	2315,15
[K].EPMIGVNQELAYFYPQLFR.[Q]	1xDeamidated [Q/N]	3	133	151	2316,14
**AS2-casein (Fragment) OS=Bubalus bubalis PE=2 SV=2**					
[K].ALNEINQFYQK.[F]		1	81	91	1367,70
[K].FPQYLQYLYQGPIVLNPWDQVK.[R]		8	92	113	2709,41
[K].TVDMESTEVITK.[K]	1xPhospho [S/T]	2	126	137	1432,63
**Beta-casein OS=Bubalus bubalis GN=CSN2 PE=2 SV=1**					
[K].FQSEEQQQMEDELQDK.[I]		1	18	33	2011,85
[K].FQSEEQQQMEDELQDK.[I]	1xOxidation [M9]	2	18	33	2027,85
[K].FQSEEQRQTEDELQDK.[I]	1xDeamidated [Q]; 1xPhospho [S3]	3	18	33	2090,86
[K].FQSEEQQQMEDELQDK.[I]	1xPhospho [S3]	1	18	33	2091,82
[K].FQSEEQQQMEDELQDK.[I]	1xDeamidated [Q]; 1xPhospho [S3]	3	18	33	2092,81
[K].FQSEEQQQMEDELQDK.[I]	2xDeamidated [Q]; 1xPhospho [S3]	8	18	33	2093,79
[K].FQSEEQQQMEDELQDK.[I]	1xPhospho [S3]; 1xOxidation [M9]	2	18	33	2107,82
[K].FQSEEQQQMEDELQDK.[I]	1xDeamidated [Q2]; 1xPhospho [S3]; 1xOxidation [M9]	1	18	33	2108,80
[KR].DMPIQAFLLYQEPVLGPVR.[G]		42	169	187	2186,17
[KR].DMPIQAFLLYQEPVLGPVR.[G]	1xDeamidated [Q]	5	169	187	2187,15
[KR].DMPIQAFLLYQEPVLGPVR.[G]	1xOxidation [M2]	1	169	187	2202,16
[K-].IHPFAQTQSLVYPFPGPIPK.[S]		11	34	53	2237,21
[K-].IHPFAQTQSLVYPFPGPIPK.[S]	1xDeamidated [Q8]	1	34	53	2238,20
[K].IEKFQSEEQQQMEDELQDK.[I]	1xPhospho [S6]	1	15	33	2462,04
[K].IEKFQSEEQQQMEDELQDK.[I]	1xDeamidated [Q]; 1xPhospho [S6]	3	15	33	2463,03
[K].IEKFQSEEQQQMEDELQDK.[I]	1xPhospho [S6]; 1xOxidation [M12]	1	15	33	2478,04
[K].SLPQNIPPLTQTPVVVPPFLQPEIMGVSK.[V]		17	54	82	3126,73
[K].SLPQNIPPLTQTPVVVPPFLQPEIMGVSK.[V]	1xDeamidated [N5]	1	54	82	3127,71
[-].RELEELNVPGEIVESLSSSEESITHINK.[K]	4xPhospho [S15; S17; S18; S19]	4	1	28	3458,45
**Kappa-casein OS=Bubalus bubalis OX=89462 GN=CSN3 PE=1 SV=2**					
[R].SPAQILQWQVLPNTVPAK.[FS]		3	69	86	1990,11
[R].YPSYGLNYYQQKPVALINNQFLPYPYYAKPAAVR.[S]	2xDeamidated [N/Q]	9	35	68	4012,03
**Beta-lactoglobulin OS=Bubalus bubalis GN=LGB PE=1 SV=2**					
[R].VYVEELKPTPEGDLEILLQK.[W]		1	41	60	2313,26

TU9

Description	Modifications	# PSMs	Start	End	Theor. MH+ [Da]
**Alpha S1 casein OS=Bos taurus OX=9913 GN=CSN1S1 PE=2 SV=1**					
[R].YLGYLEQLLR.[L]		54	91	100	1267,70
[R].FFVAPFPEVFGK.[E]		7	23	34	1384,73
[K].HQGLPQGVLNENLLR.[F]		25	8	22	1687,92
[K].HQGLPQGVLNENLLR.[F]	1xDeamidated [Q/N]	5	8	22	1688,91
[K].HQGLPQGVLNENLLR.[F]	3xDeamidated [Q2; Q6; N]	2	8	22	1690,88
[K].EPMIGVNQELAYFYPQLFR.[Q]		6	133	151	2315,15
[K].EPMIGVNQELAYFYPQLFR.[Q]	1xDeamidated [Q/N]	3	133	151	2316,14
[K].YNVPQLEIVPNLAEEQLHSMK.[E]		1	104	124	2452,25
[K].YNVPQLEIVPNLAEEQLHSMK.[E]	1xOxidation [M20]	2	104	124	2468,25
[K].VNELSTDIGSESTEDQAMEDIK.[Q]	1xPhospho [S10]	1	37	58	2491,04
[K].VNELSTDIGSESTEDQAMEDIK.[Q]	1xPhospho [S12]; 1xOxidation [M18]	1	37	58	2507,04
[K].VNELSTDIGSESTEDQAMEDIK.[Q]	2xPhospho [S/T]	2	37	58	2571,01
[K].KYNVPQLEIVPNLAEEQLHSMK.[E]		1	103	124	2580,35
[K].KYNVPQLEIVPNLAEEQLHSMK.[E]	2xDeamidated [N/Q]	3	103	124	2582,32
[K].KYNVPQLEIVPNLAEEQLHSMK.[E]	3xDeamidated [N3; Q6; N12]	1	103	124	2583,30
[K].VNELSTDIGSESTEDQAMEDIK.[Q]	2xPhospho [S10; S12]; 1xOxidation [M18]	1	37	58	2587,00
[K].KYNVPQLEIVPNLAEEQLHSMK.[E]	1xOxidation [M21]	1	103	124	2596,34
[K].VNELSKDIGSESTEDQAMEDIK.[Q]	2xPhospho [S10; T13]; 1xOxidation [M18]	1	37	58	2614,05
[K].VNELSTDIGSESTEDQAMEDIK.[Q]	3xPhospho [S10; T/S]; 1xOxidation [M18]	2	37	58	2666,97
**AS2-casein (Fragment) OS=Bubalus bubalis PE=2 SV=2**					
[K].ALNEINQFYQK.[F]		3	81	91	1367,70
[K].ALNEINQFYQK.[F]	1xDeamidated [N6]	1	81	91	1368,68
[K].FPQYLQYLYQGPIVLNPWDQVK.[R]		2	92	113	2709,41
[K].TVDMESTEVITK.[K]		1	126	137	1352,66
**Beta-casein OS=Bubalus bubalis GN=CSN2 PE=2 SV=1**					
[K].FQSEEQQQMEDELQDK.[I]		1	18	33	2011,85
[K].FQSEEQQQMEDELQDK.[I]	3xDeamidated [Q2; Q]	6	18	33	2014,81
[K].FQSEEQQQMEDELQDK.[I]	1xOxidation [M9]	1	18	33	2027,85
[K].FQSEEQRQTEDELQDK.[I]	1xDeamidated [Q8]; 1xPhospho [S3]	1	18	33	2090,86
[K].FQSEEQQQMEDELQDK.[I]	1xPhospho [S3]	4	18	33	2091,82
[K].FQSEEQQQMEDELQDK.[I]	1xDeamidated [Q]; 1xPhospho [S3]	10	18	33	2092,81
[K].FQSEEQQQMEDELQDK.[I]	2xDeamidated [Q]; 1xPhospho [S3]	2	18	33	2093,79
[K].FQSEEQQQMEDELQDK.[I]	3xDeamidated [Q]; 1xPhospho [S3]	2	18	33	2094,77
[K].FQSEEQQQMEDELQDK.[I]	5xDeamidated [Q2; Q6; Q7; Q8; Q14]; 1xPhospho [S3]	2	18	33	2096,74
[K].FQSEEQQQMEDELQDK.[I]	1xPhospho [S3]; 1xOxidation [M9]	1	18	33	2107,82
[K].FQSEEQQQMEDELQDK.[I]	1xDeamidated [Q]; 1xPhospho [S3]; 1xOxidation [M9]	2	18	33	2108,80
[K].FQSEEQQQMEDELQDK.[I]	3xDeamidated [Q]; 1xPhospho [S3]; 1xOxidation [M9]	5	18	33	2110,77
[KR].DMPIQAFLLYQEPVLGPVR.[G]		24	169	187	2186,17
[KR].DMPIQAFLLYQEPVLGPVR.[G]	1xDeamidated [Q5]	1	169	187	2187,15
[KR].DMPIQAFLLYQEPVLGPVR.[G]	1xOxidation [M2]	1	169	187	2202,16
[K-].IHPFAQTQSLVYPFPGPIPK.[S]		11	34	53	2237,21
[K].IEKFQSEEQQQMEDELQDK.[I]	1xPhospho [S6]	1	15	33	2462,04
[K].IEKFQSEEQQQMEDELQDK.[I]	1xPhospho [S6]; 1xOxidation [M12]	1	15	33	2478,04
[K].SLPQNIPPLTQTPVVVPPFLQPEIMGVSK.[V]		11	54	82	3126,73
[-].RELEELNVPGEIVESLSSSEESITHINK.[K]	2xPhospho [S]	8	1	28	3298,51
[-].RELEELNVPGEIVESLSSSEESITHINK.[K]	4xPhospho [S15; S17; S18; S19]	16	1	28	3458,45
**Kappa-casein OS=Bubalus bubalis OX=89462 GN=CSN3 PE=1 SV=2**					
[R].SPAQILQWQVLPNTVPAK.[FS]		2	69	86	1990,11
[R].SPAQILQWQVLPNTVPAK.[FS]	2xDeamidated [Q/N]	4	69	86	1992,08
[R].YPSYGLNYYQQKPVALINNQFLPYPYYAKPAAVR.[S]		1	35	68	4010,06
**Beta-lactoglobulin OS=Bubalus bubalis GN=LGB PE=1 SV=2**					
[K-].VAGTWYSLAMAASDISLLDAQSAPLR.[V]		1	15	40	2707,38
[R].TPEVDDEALEK.[F]		1	125	135	1245,58
[R].VYVEELKPTPEGDLEILLQK.[W]		1	41	60	2313,26

TU10

Description	Modifications	# PSMs	Start	End	Theor. MH+ [Da]
**Alpha-S1-casein (Fragment) OS=Bubalus bubalis GN=CSN1S PE=2 SV=1**					
[K].HQGLPQGVLNENLLR.[F]		34	8	22	1687,92
[K].HQGLPQGVLNENLLR.[F]	1xDeamidated [Q/N]	15	8	22	1688,91
[K].HQGLPQGVLNENLLR.[F]	2xDeamidated [Q/N]	3	8	22	1689,89
[R].FFVAPFPEVFGK.[E]		3	23	34	1384,73
[K].VNELSTDIGSESTEDQAMEDIK.[Q]	1xPhospho [S]	2	37	58	2491,04
[K].VNELSTDIGSESTEDQAMEDIK.[Q]	1xPhospho [S12]; 1xOxidation [M18]	1	37	58	2507,04
[K].VNELSTDIGSESTEDQAMEDIK.[Q]	2xPhospho [S/T]	3	37	58	2571,01
[K].VNELSTDIGSESTEDQAMEDIK.[Q]	2xPhospho [S/T]; 1xOxidation [M18]	4	37	58	2587,00
[R].YLGYLEQLLR.[L]		60	91	100	1267,70
[K].KYNVPQLEIVPNLAEEQLHSMK.[E]		2	103	124	2580,35
[K].KYNVPQLEIVPNLAEEQLHSMK.[E]	1xDeamidated [N/Q]	2	103	124	2581,33
[K].KYNVPQLEIVPNLAEEQLHSMK.[E]	1xOxidation [M21]	2	103	124	2596,34
[K].YNVPQLEIVPNLAEEQLHSMK.[E]		2	104	124	2452,25
[K].YNVPQLEIVPNLAEEQLHSMK.[E]	1xOxidation [M20]	1	104	124	2468,25
[K].EPMIGVNQELAYFYPQLFR.[Q]		3	133	151	2315,15
[K].EPMIGVNQELAYFYPQLFR.[Q]	1xDeamidated [Q/N]	2	133	151	2316,14
**Alpha s2-casein OS=Bubalus bubalis PE=2 SV=1**					
[K].ALNEINQFYQK.[F]		3	81	91	1367,70
[K].ALNEINQFYQK.[F]	1xDeamidated [N6]	1	81	91	1368,68
[K].FPQYLQYLYQGPIVLNPWDQVK.[R]		4	92	113	2709,41
[K].FPQYLQYLYQGPIVLNPWDQVK.[R]	1xDeamidated [Q6]	1	92	113	2710,39
[K].HTMEHVSSSEESIISQETYK.[Q]	2xPhospho [S8; S9]	1	2	21	2481,99
[K].KTVDMESTEVITK.[K]	1xPhospho [S7]; 1xOxidation [M5]	1	122	134	1576,72
[K].TVDMESTEVITK.[K]	1xPhospho [S/T]	4	123	134	1432,63
**Beta-casein OS=Bubalus bubalis GN=CSN2 PE=2 SV=1**					
[K].FQSEEQQQMEDELQDK.[I]		1	18	33	2011,85
[K].FQSEEQQQMEDELQDK.[I]	1xOxidation [M9]	1	18	33	2027,85
[K].FQSEEQQQMEDELQDK.[I]	1xPhospho [S3]	3	18	33	2091,82
[K].FQSEEQRQTEDELQDK.[I]	2xDeamidated [Q8; Q]; 1xPhospho [S3]	5	18	33	2091,84
[K].FQSEEQQQMEDELQDK.[I]	1xDeamidated [Q]; 1xPhospho [S3]	8	18	33	2092,81
[K].FQSEEQQQMEDELQDK.[I]	2xDeamidated [Q]; 1xPhospho [S3]	7	18	33	2093,79
[K].FQSEEQQQMEDELQDK.[I]	3xDeamidated [Q]; 1xPhospho [S3]	2	18	33	2094,77
[K].FQSEEQQQMEDELQDK.[I]	1xPhospho [S3]; 1xOxidation [M9]	25	18	33	2107,82
[K].FQSEEQQQMEDELQDK.[I]	1xDeamidated [Q]; 1xPhospho [S3]; 1xOxidation [M9]	13	18	33	2108,80
[KR].DMPIQAFLLYQEPVLGPVR.[G]		9	169	187	2186,17
[KR].DMPIQAFLLYQEPVLGPVR.[G]	1xDeamidated [Q5]	1	169	187	2187,15
[KR].DMPIQAFLLYQEPVLGPVR.[G]	1xOxidation [M2]	2	169	187	2202,16
[K-].IHPFAQTQSLVYPFPGPIPK.[S]		10	34	53	2237,21
[K-].IHPFAQTQSLVYPFPGPIPK.[S]	1xDeamidated [Q]	2	34	53	2238,20
[K].IEKFQSEEQQQMEDELQDK.[I]	1xPhospho [S6]	1	15	33	2462,04
[K].IEKFQSEEQQQMEDELQDK.[I]	1xDeamidated [Q]; 1xPhospho [S6]	3	15	33	2463,03
[K].SLPQNIPPLTQTPVVVPPFLQPEIMGVSK.[V]		13	54	82	3126,73
[K].SLPQNIPPLTQTPVVVPPFLQPEIMGVSK.[V]	1xDeamidated [Q/N]	3	54	82	3127,71
[K].SLPQNIPPLTQTPVVVPPFLQPEIMGVSK.[V]	1xOxidation [M25]	1	54	82	3142,72
[-].RELEELNVPGEIVESLSSSEESITHINKK.[I]	2xPhospho [S18; S19]	1	1	28	3426,61
[-].RELEELNVPGEIVESLSSSEESITHINK.[K]	4xPhospho [S15; S17; S18; S19]	8	1	28	3458,45
**Kappa-casein OS=Bubalus bubalis OX=89462 GN=CSN3 PE=1 SV=2**					
[R].SPAQILQWQVLPNTVPAK.[FS]		2	69	86	1990,11
[R].SPAQILQWQVLPNTVPAK.[FS]	1xDeamidated [N13]	1	69	86	1991,10
[R].YPSYGLNYYQQKPVALINNQFLPYPYYAKPAAVR.[S]		1	35	68	4010,06
**Beta-lactoglobulin OS=Bubalus bubalis GN=LGB PE=1 SV=2**					
[K-].VAGTWYSLAMAASDISLLDAQSAPLR.[V]		1	15	40	2707,38
[R].VYVEELKPTPEGDLEILLQK.[W]		1	41	60	2313,26
[R].TPEVDDEALEK.[F]		1	125	135	1245,58
[R].TPEVDNEALEKFDK.[A]		1	125	138	1634,79
**Alpha-lactalbumin protein variant D OS=Bos taurus OX=9913 GN=LALBA PE=3 SV=1**					
[K].VGINYWLAHK.[A]		1	99	108	1200,65

TU11

Description	Modifications	# PSMs	Start	End	Theor. MH+ [Da]
**Alpha-S1-casein (Fragment) OS=Bubalus bubalis GN=CSN1S PE=2 SV=1**					
[K].HQGLPQGVLNENLLR.[F]		17	8	22	1687,92
[K].HQGLPQGVLNENLLR.[F]	1xDeamidated [Q/N]	7	8	22	1688,91
[K].HQGLPQGVLNENLLR.[F]	3xDeamidated [Q2; Q6; N]	2	8	22	1690,88
[R].FFVAPFPEVFGK.[E]		1	23	34	1384,73
[K].EKVNELSTDIGSESTEDQAMEDIK.[Q]	1xPhospho [S12]	1	35	58	2748,18
[K].EKVNELSTDIGSESTEDQAMEDIK.[Q]	1xPhospho [S12]; 1xOxidation [M20]	1	35	58	2764,18
[K].VNELSTDIGSESTEDQAMEDIK.[Q]	1xPhospho [S10]	1	37	58	2491,04
[K].VNELSTDIGSESTEDQAMEDIK.[Q]	1xPhospho [S12]; 1xOxidation [M18]	1	37	58	2507,04
[K].VNELSTDIGSESTEDQAMEDIK.[Q]	2xPhospho [S/T]	5	37	58	2571,01
[R].YLGYLEQLLR.[L]		43	91	100	1267,70
[K].KYNVPQLEIVPNLAEEQLHSMK.[E]		1	103	124	2580,35
[K].YNVPQLEIVPNLAEEQLHSMK.[E]		2	104	124	2452,25
[K].EPMIGVNQELAYFYPQLFR.[Q]		4	133	151	2315,15
[K].EPMIGVNQELAYFYPQLFR.[Q]	1xDeamidated [N/Q]	3	133	151	2316,14
[K].EPMIGVNQELAYFYPQLFR.[Q]	1xOxidation [M3]	1	133	151	2331,15
**Alpha s2-casein OS=Bubalus bubalis PE=2 SV=1**					
[K].ALNEINQFYQK.[F]		4	81	91	1367,70
[K].ALNEINQFYQK.[F]	1xDeamidated [N]	2	81	91	1368,68
[K].FPQYLQYLYQGPIVLNPWDQVK.[R]		2	92	113	2709,41
[K].KTVDMESTEVITK.[K]	1xPhospho [S/T]	2	122	134	1560,72
[K].TVDMESTEVITK.[K]	1xPhospho [T/S]	2	126	137	1432,63
**Beta-casein OS=Bubalus bubalis GN=CSN2 PE=2 SV=1**					
[-].RELEELNVPGEIVESLSSSEESITHINK.[K]	4xPhospho [S15; S17; S18; S19]	6	1	28	3458,45
[K].IEKFQSEEQQQMEDELQDK.[I]	1xPhospho [S6]	1	15	33	2462,04
[K].IEKFQSEEQQQMEDELQDK.[I]	1xDeamidated [Q]; 1xPhospho [S6]	3	15	33	2463,03
[K].FQSEEQQQMEDELQDK.[I]		1	18	33	2011,85
[K].FQSEEQRQTEDELQDK.[I]	1xDeamidated [Q]; 1xPhospho [S3]	4	18	33	2090,86
[K].FQSEEQQQMEDELQDK.[I]	1xPhospho [S3]	2	18	33	2091,82
[K].FQSEEQQQMEDELQDK.[I]	1xDeamidated [Q]; 1xPhospho [S3]	20	18	33	2092,81
[K].FQSEEQQQMEDELQDK.[I]	2xDeamidated [Q]; 1xPhospho [S3]	9	18	33	2093,79
[K].FQSEEQQQMEDELQDK.[I]	1xPhospho [S3]; 1xOxidation [M9]	12	18	33	2107,82
[K].FQSEEQQQMEDELQDK.[I]	1xDeamidated [Q2]; 1xPhospho [S3]; 1xOxidation [M9]	1	18	33	2108,80
[K-].IHPFAQTQSLVYPFPGPIPK.[S]		7	34	53	2237,21
[K].SLPQNIPPLTQTPVVVPPFLQPEIMGVSK.[V]		12	54	82	3126,73
[KR].DMPIQAFLLYQEPVLGPVR.[G]		15	169	187	2186,17
[KR].DMPIQAFLLYQEPVLGPVR.[G]	1xDeamidated [Q]	5	169	187	2187,15
[KR].DMPIQAFLLYQEPVLGPVR.[G]	1xOxidation [M2]	1	169	187	2202,16
[KR].DMPIQAFLLYQEPVLGPVR.[G]	1xDeamidated [Q5]; 1xOxidation [M2]	1	169	187	2203,15
**Kappa-casein OS=Cervus nippon OX=9863 GN=CSN3 PE=2 SV=1**					
[R].SPAQILQWQVLPNTVPAK.[FS]		2	69	86	1990,11
**Beta-lactoglobulin OS=Bubalus bubalis GN=LGB PE=1 SV=2**					
[K-].VAGTWYSLAMAASDISLLDAQSAPLR.[V]		1	15	40	2707,38
[R].VYVEELKPTPEGDLEILLQK.[W]		1	41	60	2313,26
[R].TPEVDNEALEKFDK.[A]		1	125	138	1634,79
[R].TPEVDNEALEKFDK.[A]	1xDeamidated [N6]	1	125	138	1635,77

TU12

Description	Modifications	# PSMs	Start	End	Theor. MH+ [Da]
**Alpha-S1-casein (Fragment) OS=Bubalus bubalis GN=CSN1S PE=2 SV=1**					
[R].YLGYLEQLLR.[L]		12	91	100	1267,70
[K].VNELSTDIGSESTEDQAMEDIK.[Q]	1xPhospho [S10]	4	37	58	2491,04
[K].VNELSTDIGSESTEDQAMEDIK.[Q]	1xPhospho [S12]; 1xOxidation [M18]	4	37	58	2507,04
[K].VNELSTDIGSESTEDQAMEDIK.[Q]	2xPhospho [S10; T13]	4	37	58	2571,01
[K].VNELSTDIGSESTEDQAMEDIK.[Q]	2xPhospho [S10; T/S]; 1xOxidation [M18]	4	37	58	2587,00
**Alpha s2-casein OS=Bubalus bubalis PE=2 SV=1**					
[K].TVDMESTEVITK.[K]	1xPhospho [S/T]	5	123	134	1432,63
**Beta-casein OS=Bubalus bubalis GN=CSN2 PE=2 SV=1**					
[K].FQSEEQQQMEDELQDK.[I]		2	18	33	2011,85
[K].FQSEEQQQMEDELQDK.[I]	1xOxidation [M9]	2	18	33	2027,85
[K].FQSEEQRQTEDELQDK.[I]	1xDeamidated [Q8]; 1xPhospho [S3]	1	18	33	2090,86
[K].FQSEEQQQMEDELQDK.[I]	1xPhospho [S3]	2	18	33	2091,82
[K].FQSEEQQQMEDELQDK.[I]	1xDeamidated [Q]; 1xPhospho [S3]	2	18	33	2092,81
[K].FQSEEQQQMEDELQDK.[I]	2xDeamidated [Q2; Q]; 1xPhospho [S3]	2	18	33	2093,79
[K].FQSEEQQQMEDELQDK.[I]	1xPhospho [S3]; 1xOxidation [M9]	2	18	33	2107,82
[K].FQSEEQQQMEDELQDK.[I]	1xDeamidated [Q]; 1xPhospho [S3]; 1xOxidation [M9]	2	18	33	2108,80
[K].IEKFQSEEQQQMEDELQDK.[I]	1xPhospho [S6]	2	15	33	2462,04
[K].IEKFQSEEQQQMEDELQDK.[I]	1xDeamidated [Q9]; 1xPhospho [S6]	2	15	33	2463,03
[-].RELEELNVPGEIVESLSSSEESITHINK.[K]	4xPhospho [S15; S17; S18; S19]	2	1	28	3458,45

TU13

Description	Modifications	# PSMs	Start	End	Theor. MH+ [Da]
**Alpha-S1-casein (Fragment) OS=Bubalus bubalis GN=CSN1S PE=2 SV=1**					
[K].EPMIGVNQELAYFYPQLFR.[Q]		8	133	151	2315,15
[K].EPMIGVNQELAYFYPQLFR.[Q]	1xDeamidated [N7]	1	133	151	2316,14
[K].HQGLPQGVLNENLLR.[F]		34	8	22	1687,92
[K].HQGLPQGVLNENLLR.[F]	1xDeamidated [Q/N]	11	8	22	1688,91
[K].KYNVPQLEIVPNLAEEQLHSMK.[E]		1	103	124	2580,35
[K].KYNVPQLEIVPNLAEEQLHSMK.[E]	1xDeamidated [N12]	1	103	124	2581,33
[K].KYNVPQLEIVPNLAEEQLHSMK.[E]	1xOxidation [M21]	1	103	124	2596,34
[K].VNELSTDIGSESTEDQAMEDIK.[Q]	1xPhospho [T/S]	2	37	58	2491,04
[K].VNELSTDIGSESTEDQAMEDIK.[Q]	1xDeamidated [N2]; 1xPhospho [T/S]	2	37	58	2492,03
[K].VNELSTDIGSESTEDQAMEDIK.[Q]	2xPhospho [S10; T13]	1	37	58	2571,01
[K].VNELSTDIGSESTEDQAMEDIK.[Q]	1xPhospho [S/T]	2	37	58	2491,04
[K].VNELSTDIGSESTEDQAMEDIK.[Q]	1xPhospho [S]; 1xOxidation [M18]	2	37	58	2507,04
[K].VNELSTDIGSESTEDQAMEDIK.[Q]	2xPhospho [S/T]	7	37	58	2571,01
[K].VNELSTDIGSESTEDQAMEDIK.[Q]	2xPhospho [S/T]; 1xOxidation [M18]	3	37	58	2587,00
[K].YNVPQLEIVPNLAEEQLHSMK.[E]	1xDeamidated [N11]	1	104	124	2453,24
[K].YNVPQLEIVPNLAEEQLHSMK.[E]	1xOxidation [M20]	1	104	124	2468,25
[R].FFVAPFPEVFGK.[E]		2	23	34	1384,73
[R].YLGYLEQLLR.[L]		48	91	100	1267,70
**Alpha s2-casein OS=Bubalus bubalis PE=2 SV=1**					
[K].ALNEINQFYQK.[F]		5	81	91	1367,70
[K].ALNEINQFYQK.[F]	1xDeamidated [N6]	1	81	91	1368,68
[K].FPQYLQYLYQGPIVLNPWDQVK.[R]	1xDeamidated [Q10]	1	92	113	2710,39
[K].KTVDMESTEVITK.[K]	1xPhospho [S7]; 1xOxidation [M5]	1	125	137	1576,72
[K].TVDMESTEVITK.[K]	1xPhospho [T/S]	2	126	137	1432,63
[K].TVDMESTEVITK.[K]	1xPhospho [S6]	1	123	134	1432,63
**Beta-casein OS=Bubalus bubalis GN=CSN2 PE=2 SV=1**					
[-].RELEELNVPGEIVESLSSSEESITHINK.[K]	2xPhospho [S18; S19]	2	1	28	3298,51
[-].RELEELNVPGEIVESLSSSEESITHINK.[K]	4xPhospho [S15; S17; S18; S19]	2	1	15	3458,45
[-].RELEELNVPGEIVESLSSSEESITHINK.[K]	4xPhospho [S15; S17; S18; S19]	4	1	15	3458,45
[-].RELEELNVPGEIVESLSSSEESITHINKK.[I]	2xPhospho [S18; S19]	1	1	15	3426,61
[K].FQSEEQQQMEDELQDK.[I]		1	18	33	2011,85
[K].FQSEEQQQMEDELQDK.[I]	1xOxidation [M9]	2	18	33	2027,85
[K].FQSEEQQQMEDELQDK.[I]	1xPhospho [S3]	5	18	33	2091,82
[K].FQSEEQQQMEDELQDK.[I]	1xDeamidated [Q]; 1xPhospho [S3]	6	18	33	2092,81
[K].FQSEEQQQMEDELQDK.[I]	1xPhospho [S3]; 1xOxidation [M9]	2	18	33	2107,82
[K].FQSEEQQQMEDELQDK.[I]	1xDeamidated [Q]; 1xPhospho [S3]; 1xOxidation [M9]	3	18	33	2108,80
[K].FQSEEQQQMEDELQDK.[I]	1xOxidation [M9]	1	18	33	2027,85
[K].FQSEEQQQMEDELQDK.[I]	1xPhospho [S3]	30	18	33	2091,82
[K].FQSEEQQQMEDELQDK.[I]	1xDeamidated [Q]; 1xPhospho [S3]	4	18	33	2092,81
[K].FQSEEQQQMEDELQDK.[I]	2xDeamidated [Q]; 1xPhospho [S3]	8	18	33	2093,79
[K].FQSEEQQQMEDELQDK.[I]	1xPhospho [S3]; 1xOxidation [M9]	16	18	33	2107,82
[K].FQSEEQQQMEDELQDK.[I]	1xDeamidated [Q2]; 1xPhospho [S3]; 1xOxidation [M9]	1	18	33	2108,80
[K].FQSEEQQQMEDELQDK.[I]	2xDeamidated [Q]; 1xPhospho [S3]; 1xOxidation [M9]	3	18	33	2109,78
[K].FQSEEQQQMEDELQDK.[I]	4xDeamidated [Q2; Q6; Q7; Q14]; 1xPhospho [S3]; 1xOxidation [M9]	1	18	33	2111,75
[K].IEKFQSEEQQQMEDELQDK.[I]	1xPhospho [S6]	1	15	33	2462,04
[K-].IHPFAQTQSLVYPFPGPIPK.[S]		5	34	53	2237,21
[K-].IHPFAQTQSLVYPFPGPIPK.[S]	1xDeamidated [Q]	2	34	53	2238,20
[K].SLPQNIPPLTQTPVVVPPFLQPEIMGVSK.[V]		7	54	82	3126,73
[KR].DMPIQAFLLYQEPVLGPVR.[G]		16	169	187	2186,17
[KR].DMPIQAFLLYQEPVLGPVR.[G]	1xDeamidated [Q11]	2	169	187	2187,15
[KR].DMPIQAFLLYQEPVLGPVR.[G]	1xOxidation [M2]	1	169	187	2202,16
[KR].DMPIQAFLLYQEPVLGPVR.[G]	1xDeamidated [Q5]; 1xOxidation [M2]	1	169	187	2203,15
**Kappa-casein OS=Bubalus bubalis OX=89462 GN=CSN3 PE=1 SV=2**					
[R].SPAQILQWQVLPNTVPAK.[FS]		2	69	86	1990,11
[R].YPSYGLNYYQQKPVALINNQFLPYPYYAKPAAVR.[S]		1	41	74	4010,06

TU14

Description	Modifications	# PSMs	Start	End	Theor. MH+ [Da]
**Alpha S1 casein OS=Bos taurus OX=9913 GN=CSN1S1 PE=2 SV=1**					
[R].YLGYLEQLLR.[L]		12	91	100	1267,70
[K].HQGLPQGVLNENLLR.[F]		5	8	22	1687,92
[K].HQGLPQGVLNENLLR.[F]	1xDeamidated [Q/N]	5	8	22	1688,91
[K].HQGLPQGVLNENLLR.[F]	3xDeamidated [Q2; Q6; N]	5	8	22	1690,88
[K].HQGLPQEVLNENLLR.[F]		5	8	22	1759,94
[K].EPMIGVNQELAYFYPQLFR.[Q]		5	133	151	2315,15
[K].YNVPQLEIVPNLAEEQLHSMK.[E]	1xDeamidated [N11]	5	104	124	2453,24
[K].VNELSTDIGSESTEDQAMEDIK.[Q]	1xPhospho [S12]; 1xOxidation [M18]	4	37	58	2507,04
[K].VNELSTDIGSESTEDQAMEDIK.[Q]	2xPhospho [S/T]	4	37	58	2571,01
[K].KYNVPQLEIVPNLAEEQLHSMK.[E]		5	103	124	2580,35
[K].KYNVPQLEIVPNLAEEQLHSMK.[E]	1xDeamidated [Q/N]	5	103	124	2581,33
[K].VNELSTDIGSESTEDQAMEDIK.[Q]	2xPhospho [S10; T13]; 1xOxidation [M18]	4	37	58	2587,00
[K].VNELSKDIGSESTEDQAMEDIK.[Q]	2xPhospho [S10; T13]; 1xOxidation [M18]	6	37	58	2614,05
[K].VNELSTDIGSESTEDQAMEDIK.[Q]	3xPhospho [S5; S10; S12]; 1xOxidation [M18]	4	37	58	2666,97
[K].EKVNELSTDIGSESTEDQAMEDIK.[Q]	1xPhospho [S12]	4	35	58	2748,18
**Alpha s2-casein OS=Bubalus bubalis PE=2 SV=1**					
[K].TVDMESTEVITK.[K]	1xPhospho [S6]	5	123	134	1432,63
**Beta-casein OS=Bubalus bubalis GN=CSN2 PE=2 SV=1**					
[K].FQSEEQQQMEDELQDK.[I]		2	18	33	2011,85
[K].FQSEEQQQMEDELQDK.[I]	1xOxidation [M9]	2	18	33	2027,85
[K].FQSEEQQQTEDELQDK.[I]	1xPhospho [S3]	9	33	48	2061,83
[K].FQSEEQRQTEDELQDK.[I]	1xDeamidated [Q8]; 1xPhospho [S3]	1	18	33	2090,86
[K].FQSEEQQQMEDELQDK.[I]	1xPhospho [S3]	2	18	33	2091,82
[K].FQSEEQQQMEDELQDK.[I]	1xPhospho [S3]	2	18	33	2091,82
[K].FQSEEQQQMEDELQDK.[I]	1xDeamidated [Q]; 1xPhospho [S3]	2	18	33	2092,81
[K].FQSEEQQQMEDELQDK.[I]	3xDeamidated [Q2; Q]; 1xPhospho [S3]	2	18	33	2094,77
[K].FQSEEQQQMEDELQDK.[I]	1xPhospho [S3]; 1xOxidation [M9]	2	18	33	2107,82
[K].FQSEEQQQMEDELQDK.[I]	1xDeamidated [Q]; 1xPhospho [S3]; 1xOxidation [M9]	2	18	33	2108,80
[KR].DMPIQAFLLYQEPVLGPVR.[G]		20	182	200	2186,17
[KR].DMPIQAFLLYQEPVLGPVR.[G]	1xDeamidated [Q5]	20	182	200	2187,15
[KR].DMPIQAFLLYQEPVLGPVR.[G]	1xOxidation [M2]	20	182	200	2202,16
[KR].DMPIQAFLLYQEPVLGPVR.[G]	1xDeamidated [Q5]; 1xOxidation [M2]	20	182	200	2203,15
[K-].IHPFAQTQSLVYPFPGPIPK.[S]		4	34	53	2237,21
[K].IEKFQSEEQQQMEDELQDK.[I]	1xPhospho [S6]	2	15	33	2462,04
[K].IEKFQSEEQQQMEDELQDK.[I]	1xDeamidated [Q]; 1xPhospho [S6]	2	15	33	2463,03
[K].IEKFQSEEQQQMEDELQDK.[I]	1xPhospho [S6]; 1xOxidation [M12]	2	15	33	2478,04
[K].SLPQNIPPLTQTPVVVPPFLQPEIMGVSK.[V]		9	54	82	3126,73
[-].RELEELNVPGEIVESLSSSEESITHINK.[K]	4xPhospho [S15; S17; S18; S19]	2	1	28	3458,45
**Kappa-casein OS=Bubalus bubalis OX=89462 GN=CSN3 PE=1 SV=2**					
[R].HPHPHLSFMAIPPK.[K]		36	98	111	1608,85
[R].SPAQILQWQVLPNTVPAK.[FS]		15	69	86	1990,11
[R].YPSYGLNYYQQKPVALINNQFLPYPYYAKPAAVR.[S]	1xDeamidated [N]	22	35	68	4011,05
**Beta-lactoglobulin variant D (Fragment) OS=Bos taurus OX=9913 GN=LGB PE=3 SV=1**					
[K-].VAGTWYSLAMAASDISLLDAQSAPLR.[V]		1	15	40	2707,38
[R].VYVEELKPTPEGDLEILLQK.[W]		1	41	60	2313,26

TU15

Description	Modifications	# PSMs	Start	End	Theor. MH+ [Da]
**Alpha S1 casein OS=Bos taurus OX=9913 GN=CSN1S1 PE=2 SV=1**					
[R].YLGYLEQLLR.[L]		50	91	100	1267,70
[R].FFVAPFPEVFGK.[E]		8	23	34	1384,73
[K].HQGLPQGVLNENLLR.[F]		35	8	22	1687,92
[K].HQGLPQGVLNENLLR.[F]	1xDeamidated [Q/N]	12	8	22	1688,91
[K].HQGLPQEVLNENLLR.[F]		1	8	22	1759,94
[K].EPMIGVNQELAYFYPQLFR.[Q]		6	133	151	2315,15
[K].EPMIGVNQELAYFYPQLFR.[Q]	1xDeamidated [N/Q]	2	133	151	2316,14
[K].YNVPQLEIVPNLAEEQLHSMK.[E]		2	104	124	2452,25
[K].YNVPQLEIVPNLAEEQLHSMK.[E]	1xOxidation [M20]	1	104	124	2468,25
[K].VNELSTDIGSESTEDQAMEDIK.[Q]	1xPhospho [S10]	1	37	58	2491,04
[K].VNELSTDIGSESTEDQAMEDIK.[Q]	1xPhospho [S]; 1xOxidation [M18]	2	37	58	2507,04
[K].VNELSTDIGSESTEDQAMEDIK.[Q]	2xPhospho [S/T]	2	37	58	2571,01
[K].KYNVPQLEIVPNLAEEQLHSMK.[E]		10	103	124	2580,35
[K].KYNVPQLEIVPNLAEEQLHSMK.[E]	1xDeamidated [Q/N]	4	103	124	2581,33
[K].VNELSTDIGSESTEDQAMEDIK.[Q]	2xPhospho [S/T]; 1xOxidation [M18]	5	37	58	2587,00
[K].VNELSTDIGSESTEDQAMEDIK.[Q]	3xPhospho [S10; T/S]	3	37	58	2650,98
[K].EGIHAQQKEPMIGVNQELAYFYPQLFR.[Q]		3	125	151	3206,61
[K].EGIHAQQKEPMIGVNQELAYFYPQLFR.[Q]	1xDeamidated [Q/N]	7	125	151	3207,59
[K].EGIHAQQKEPMIGVNQELAYFYPQLFR.[Q]	2xDeamidated [Q6; Q7]	1	125	151	3208,58
**Alpha s2-casein OS=Bubalus bubalis PE=2 SV=1**					
[K].KTVDMESTEVITK.[K]	1xPhospho [S7]; 1xOxidation [M5]	1	122	134	1576,72
**Beta-casein OS=Bubalus bubalis GN=CSN2 PE=2 SV=1**					
[K].FQSEEQQQMEDELQDK.[I]		1	33	48	2011,85
[K].FQSEEQQQMEDELQDK.[I]	1xOxidation [M9]	1	33	48	2027,85
[K].FQSEEQQQTEDELQDK.[I]	1xPhospho [S3]	1	33	48	2061,83
[K].FQSEEQRQTEDELQDK.[I]	1xDeamidated [Q]; 1xPhospho [S3]	4	33	48	2090,86
[K].FQSEEQQQMEDELQDK.[I]	1xPhospho [S3]	45	33	48	2091,82
[K].FQSEEQQQMEDELQDK.[I]	1xPhospho [S3]	1	33	48	2091,82
[K].FQSEEQQQMEDELQDK.[I]	1xDeamidated [Q]; 1xPhospho [S3]	9	33	48	2092,81
[K].FQSEEQQQMEDELQDK.[I]	2xDeamidated [Q]; 1xPhospho [S3]	10	33	48	2093,79
[K].FQSEEQQQMEDELQDK.[I]	1xPhospho [S3]; 1xOxidation [M9]	2	33	48	2107,82
[K].FQSEEQQQMEDELQDK.[I]	1xDeamidated [Q]; 1xPhospho [S3]; 1xOxidation [M9]	4	33	48	2108,80
[KR].DMPIQAFLLYQEPVLGPVR.[G]		15	184	202	2186,17
[KR].DMPIQAFLLYQEPVLGPVR.[G]	1xOxidation [M2]	4	184	202	2202,16
[K-].IHPFAQTQSLVYPFPGPIPK.[S]		9	49	68	2237,21
[K].IEKFQSEEQQQMEDELQDK.[I]	1xPhospho [S6]	1	30	48	2462,04
[K].IEKFQSEEQQQMEDELQDK.[I]	1xDeamidated [Q11]; 1xPhospho [S6]	1	30	48	2463,03
[K].IEKFQSEEQQQMEDELQDK.[I]	1xPhospho [S6]; 1xOxidation [M12]	1	30	48	2478,04
[R].DMPIQAFLLYQEPVLGPVRGPFPIIV.[-]	1xOxidation [M2]	2	184	209	2925,59
[K].SLPQNIPPLTQTPVVVPPFLQPEIMGVSK.[V]		9	69	97	3126,73
[K].SLPQNIPPLTQTPVVVPPFLQPEIMGVSK.[V]	1xDeamidated [Q/N]	3	69	97	3127,71
[-].RELEELNVPGEIVESLSSSEESITHINK.[K]	4xPhospho [S15; S17; S18; S19]	12	1	28	3458,45
**Kappa-casein OS=Bubalus bubalis OX=89462 GN=CSN3 PE=1 SV=2**					
[R].SPAQILQWQVLPNTVPAK.[FS]		6	75	92	1990,11
[R].SPAQILQWQVLPNTVPAK.[FS]	1xDeamidated [Q/N]	2	75	92	1991,10
[R].YPSYGLNYYQQKPVALINNQFLPYPYYAKPAAVR.[S]		7	41	74	4010,06
[R].YPSYGLNYYQQKPVALINNQFLPYPYYAKPAAVR.[S]	1xDeamidated [N/Q]	23	41	74	4011,05
[R].YPSYGLNYYQQKPVALINNQFLPYPYYAKPAAVR.[S]	2xDeamidated [N/Q]	7	41	74	4012,03
**Beta-lactoglobulin OS=Bubalus bubalis GN=LGB PE=1 SV=2**					
[R].TPEVDDEALEK.[F]		1	125	135	1245,58

TU16

Description	Modifications	# PSMs	Start	End	Theor. MH+ [Da]
**Alpha-S1-casein (Fragment) OS=Bubalus bubalis GN=CSN1S PE=2 SV=1**					
[R].YLGYLEQLLR.[L]		40	91	100	1267,70
[R].FFVAPFPEVFGK.[E]		12	23	34	1384,73
[K].HQGLPQGVLNENLLR.[F]		28	8	22	1687,92
[K].HQGLPQGVLNENLLR.[F]	1xDeamidated [Q/N]	8	8	22	1688,91
[K].EPMIGVNQELAYFYPQLFR.[Q]		17	133	151	2315,15
[K].EPMIGVNQELAYFYPQLFR.[Q]	1xDeamidated [Q/N]	2	133	151	2316,14
[K].YNVPQLEIVPNLAEEQLHSMK.[E]		3	104	124	2452,25
[K].YNVPQLEIVPNLAEEQLHSMK.[E]	1xDeamidated [Q16]	1	104	124	2453,24
[K].YNVPQLEIVPNLAEEQLHSMK.[E]	1xOxidation [M20]	1	104	124	2468,25
[K].VNELSTDIGSESTEDQAMEDIK.[Q]	1xPhospho [T/S]	2	37	58	2491,04
[K].KYNVPQLEIVPNLAEEQLHSMK.[E]		9	103	124	2580,35
[K].KYNVPQLEIVPNLAEEQLHSMK.[E]	1xDeamidated [N12]	2	103	124	2581,33
[K].VNELSTDIGSESTEDQAMEDIK.[Q]	2xPhospho [S/T]; 1xOxidation [M18]	3	37	58	2587,00
[K].EGIHAQQKEPMIGVNQELAYFYPQLFR.[Q]		4	125	151	3206,61
**Alpha s2-casein OS=Bubalus bubalis PE=2 SV=1**					
[K].ALNEINQFYQK.[F]		4	81	91	1367,70
[K].ALNEINQFYQK.[F]	1xDeamidated [N/Q]	2	81	91	1368,68
[K].TVDMESTEVITK.[K]	1xPhospho [S6]	2	126	137	1432,63
[K].TVDMESTEVITKK.[T]	1xPhospho [S6]	1	123	135	1560,72
[K].KTVDMESTEVITKK.[T]	1xPhospho [S7]	1	122	135	1688,82
[K].FPQYLQYLYQGPIVLNPWDQVK.[R]		3	92	113	2709,41
**Beta-casein OS=Bubalus bubalis GN=CSN2 PE=2 SV=1**					
[K].FQSEEQQQMEDELQDK.[I]		1	18	33	2011,85
[K].FQSEEQQQMEDELQDK.[I]	1xOxidation [M9]	1	18	33	2027,85
[K].FQSEEQRQTEDELQDK.[I]	1xDeamidated [Q8]; 1xPhospho [S3]	3	18	33	2090,86
[K].FQSEEQQQMEDELQDK.[I]	1xPhospho [S3]	25	18	33	2091,82
[K].FQSEEQQQMEDELQDK.[I]	1xDeamidated [Q2]; 1xPhospho [S3]	1	18	33	2092,81
[K].FQSEEQQQMEDELQDK.[I]	2xDeamidated [Q]; 1xPhospho [S3]	3	18	33	2093,79
[K].FQSEEQQQMEDELQDK.[I]	1xPhospho [S3]; 1xOxidation [M9]	2	18	33	2107,82
[KR].DMPIQAFLLYQEPVLGPVR.[G]		50	169	187	2186,17
[KR].DMPIQAFLLYQEPVLGPVR.[G]	1xDeamidated [Q]	6	169	187	2187,15
[KR].DMPIQAFLLYQEPVLGPVR.[G]	1xOxidation [M2]	27	169	187	2202,16
[KR].DMPIQAFLLYQEPVLGPVR.[G]	1xDeamidated [Q]; 1xOxidation [M2]	5	169	187	2203,15
[K-].IHPFAQTQSLVYPFPGPIPK.[S]		9	34	53	2237,21
[K].IEKFQSEEQQQMEDELQDK.[I]	1xPhospho [S6]	1	15	33	2462,04
[K].IEKFQSEEQQQMEDELQDK.[I]	1xDeamidated [Q]; 1xPhospho [S6]	3	15	33	2463,03
[K].IEKFQSEEQQQMEDELQDK.[I]	1xPhospho [S6]; 1xOxidation [M12]	1	15	33	2478,04
[R].DMPIQAFLLYQEPVLGPVRGPFPIIV.[-]		2	169	194	2909,60
[K].SLPQNIPPLTQTPVVVPPFLQPEIMGVSK.[V]		7	54	82	3126,73
[-].RELEELNVPGEIVESLSSSEESITHINK.[K]	4xPhospho [S15; S17; S18; S19]	10	1	28	3458,45
[-].RELEELNVPGEIVESLSSSEESITHINKK.[I]	3xPhospho [S17; S18; S19]	1	1	29	3506,57
[-].RELEELNVPGEIVESLSSSEESITHINKK.[I]	3xPhospho [S17; S18; S19]	1	1	29	3506,57
**Kappa-casein OS=Bubalus bubalis OX=89462 GN=CSN3 PE=1 SV=2**					
[K-].YIPIQYVLSR.[Y]		1	25	34	1251,71
[R].SPAQILQWQVLPNTVPAK.[FS]		16	69	86	1990,11
[R].SPAQILQWQVLPNTVPAK.[FS]	2xDeamidated [Q9; N13]	1	69	86	1992,08
[R].YPSYGLNYYQQKPVALINNQFLPYPYYAKPAAVR.[S]	1xDeamidated [Q/N]	9	35	68	4011,05
[R].YPSYGLNYYQQKPVALINNQFLPYPYYAKPAAVR.[S]		8	35	68	4010,06
[R].YPSYGLNYYQQKPVALINNQFLPYPYYAKPAAVR.[S]	3xDeamidated [N7; Q10; Q20]	1	35	68	4013,02
[R].YPSYGLNYYQQKPVALINNQFLPYPYYAKPAAVR.[S]	2xDeamidated [Q/N]	6	35	68	4012,03
**Beta-lactoglobulin OS=Capra hircus OX=9925 GN=LGB PE=1 SV=2**					
[K-].VAGTWYSLAMAASDISLLDAQSAPLR.[V]		1	15	40	2707,38
[R].VYVEELKPTPEGNLEILLQK.[W]		1	41	60	2312,27
**Alpha-lactalbumin protein variant D OS=Bos taurus OX=9913 GN=LALBA PE=3 SV=1**					
[K].VGINYWLAHK.[A]		1	99	108	1200,65

TU17

Description	Modifications	# PSMs	Start	End	Theor. MH+ [Da]
**Alpha-S1-casein (Fragment) OS=Bubalus bubalis GN=CSN1S PE=2 SV=1**					
[K].VNELSTDIGSESTEDQAMEDIK.[Q]	1xPhospho [S10]	1	37	58	2491,04
[K].VNELSTDIGSESTEDQAMEDIK.[Q]	2xPhospho [S10; T13]	1	37	58	2571,01
[K].VNELSTDIGSESTEDQAMEDIK.[Q]	2xPhospho [S10; T/S]; 1xOxidation [M18]	2	37	58	2587,00
**Alpha S1 casein OS=Bos taurus OX=9913 GN=CSN1S1 PE=2 SV=1**					
[K].HQGLPQEVLNENLLR.[F]		1	8	22	1759,94
[R].FFVAPFPEVFGK.[E]		12	23	34	1384,73
[R].YLGYLEQLLR.[L]		49	91	100	1267,70
**Alpha-S1-casein OS=Ovis aries OX=9940 GN=CSN1S1 PE=1 SV=3**					
[K].QPMIAVNQELAYFYPQLFR.[Q]		1	133	151	2328,18
[R].YLGYLEQLLR.[L]		49	91	100	1267,70
**Alpha s2-casein OS=Bubalus bubalis PE=2 SV=1**					
[K].TVDMESTEVITK.[K]	1xPhospho [S/T]	3	123	134	1432,63
**Beta-casein OS=Bubalus bubalis GN=CSN2 PE=2 SV=1**					
[-].RELEELNVPGEIVESLSSSEESITHINK.[K]	4xPhospho [S15; S17; S18; S19]	10	1	28	3458,45
[K].FQSEEQQQMEDELQDK.[I]		1	18	33	2011,85
[K].FQSEEQQQMEDELQDK.[I]	1xPhospho [S3]	30	18	33	2091,82
[K].FQSEEQQQMEDELQDK.[I]	1xDeamidated [Q]; 1xPhospho [S3]	4	18	33	2092,81
[K].FQSEEQQQMEDELQDK.[I]	2xDeamidated [Q2; Q]; 1xPhospho [S3]	10	18	33	2093,79
[K].FQSEEQQQMEDELQDK.[I]	1xPhospho [S3]; 1xOxidation [M9]	3	18	33	2107,82
[K].FQSEEQQQMEDELQDK.[I]	1xDeamidated [Q]; 1xPhospho [S3]; 1xOxidation [M9]	3	18	33	2108,80
[K].IEKFQSEEQQQMEDELQDK.[I]	1xPhospho [S6]	1	15	33	2462,04
[K].IEKFQSEEQQQMEDELQDK.[I]	1xDeamidated [Q9]; 1xPhospho [S6]	1	15	33	2463,03
**Kappa-casein OS=Bubalus bubalis OX=89462 GN=CSN3 PE=1 SV=2**					
[R].SPAQILQWQVLPNTVPAK.[FS]		4	69	86	1990,11
[R].SPAQILQWQVLPNTVPAK.[FS]	1xDeamidated [N/Q]	3	69	86	1991,10
[R].SPAQILQWQVLPNTVPAK.[FS]	2xDeamidated [Q9; N13]	1	69	86	1992,08
[R].YPSYGLNYYQQKPVALINNQFLPYPYYAKPAAVR.[S]		3	2	35	4010,06
[R].YPSYGLNYYQQKPVALINNQFLPYPYYAKPAAVR.[S]	1xDeamidated [Q/N]	3	2	35	4011,05
[R].YPSYGLNYYQQKPVALINNQFLPYPYYAKPAAVR.[S]	2xDeamidated [N7; Q/N]	3	2	35	4012,03
**Kappa-casein (Fragment) OS=Bos taurus OX=9913 GN=CSN3 PE=4 SV=1**					
[R].SPAQILQWQVLSNTVPAK.[S]		1	69	86	1980,09
[R].YPSYGLNYYQQKPVALINNQFLPYPYYAKPAAVR.[S]		3	2	35	4010,06
[R].YPSYGLNYYQQKPVALINNQFLPYPYYAKPAAVR.[S]	1xDeamidated [Q/N]	3	2	35	4011,05
[R].YPSYGLNYYQQKPVALINNQFLPYPYYAKPAAVR.[S]	2xDeamidated [N7; Q/N]	3	2	35	4012,03
**Kappa-casein OS=Ovis aries OX=9940 GN=CSN3 PE=1 SV=2**					
[R].SPAQTLQWQVLPNAVPAK.[S]		1	69	86	1948,07
[R].YPSYGLNYYQQRPVALINNQFLPYPYYAKPVAVR.[S]		1	35	68	4066,10
**Beta-lactoglobulin OS=Bubalus bubalis GN=LGB PE=1 SV=2**					
[K-].VAGTWYSLAMAASDISLLDAQSAPLR.[V]		1	15	40	2707,38
[R].TPEVDDEALEK.[F]		1	125	135	1245,58
**Alpha-lactalbumin protein variant D OS=Bos taurus OX=9913 GN=LALBA PE=3 SV=1**					
[K].VGINYWLAHK.[A]		1	99	108	1200,65

TU18

Description	Modifications	# PSMs	Start	End	Theor. MH+ [Da]
**Alpha S1 casein OS=Bos taurus OX=9913 GN=CSN1S1 PE=2 SV=1**					
[R].YLGYLEQLLR.[L]		43	91	100	1267,70
[R].FFVAPFPEVFGK.[E]		10	23	34	1384,73
[K].HQGLPQGVLNENLLR.[F]		11	8	22	1687,92
[K].HQGLPQGVLNENLLR.[F]	1xDeamidated [Q2]	1	8	22	1688,91
[K].YKVPQLEIVPNSAEER.[L]	1xPhospho [S12]	1	104	119	1951,95
[K].EPMIGVNQELAYFYPQLFR.[Q]		16	133	151	2315,15
[K].YNVPQLEIVPNLAEEQLHSMK.[E]		1	104	124	2452,25
[K].YNVPQLEIVPNLAEEQLHSMK.[E]	1xOxidation [M20]	1	104	124	2468,25
[K].YNVPQLEIVPNLAEEQLHSMK.[E]	1xDeamidated [Q16]; 1xOxidation [M20]	1	104	124	2469,23
[K].KYNVPQLEIVPNLAEEQLHSMK.[E]		5	103	124	2580,35
[K].KYNVPQLEIVPNLAEEQLHSMK.[E]	1xDeamidated [N/Q]	5	103	124	2581,33
[K].VNELSTDIGSESTEDQAMEDIK.[Q]	2xPhospho [S10; T/S]; 1xOxidation [M18]	2	37	58	2587,00
[K].KYNVPQLEIVPNLAEEQLHSMK.[E]	1xOxidation [M21]	1	103	124	2596,34
[K].EGIHAQQKEPMIGVNQELAYFYPQLFR.[Q]	1xOxidation [M11]	1	125	151	3222,60
[K].EGIHAQQKEPMIGVNQELAYFYPQLFR.[Q]	1xDeamidated [Q]; 1xOxidation [M11]	2	125	151	3223,59
**Beta casein (Fragment) OS=Bubalus bubalis PE=4 SV=1**					
[K].FQSEEQQQMEDELQDK.[I]		1	33	48	2011,85
[K].FQSEEQQQMEDELQDK.[I]	4xDeamidated [Q2; Q6; Q7; Q8]	1	33	48	2015,79
[K].FQSEEQQQMEDELQDK.[I]	1xOxidation [M9]	1	33	48	2027,85
[K].FQSEEQQQMEDELQDK.[I]	1xDeamidated [Q2]; 1xOxidation [M9]	1	33	48	2028,83
[K].FQSEEQQQTEDELQDK.[I]	1xPhospho [S3]	1	48	63	2061,83
[K].FQSEEQQQMEDELQDK.[I]	1xPhospho [S3]	4	33	48	2091,82
[K].FQSEEQQQMEDELQDK.[I]	1xDeamidated [Q2]; 1xPhospho [S3]	7	33	48	2092,81
[K].FQSEEQQQMEDELQDK.[I]	2xDeamidated [Q]; 1xPhospho [S3]	2	33	48	2093,79
[K].FQSEEQQQMEDELQDK.[I]	1xPhospho [S3]; 1xOxidation [M9]	1	33	48	2107,82
[K].FQSEEQQQMEDELQDK.[I]	1xDeamidated [Q]; 1xPhospho [S3]; 1xOxidation [M9]	6	33	48	2108,80
[KR].DMPIQAFLLYQEPVLGPVR.[G]		40	184	202	2186,17
[KR].DMPIQAFLLYQEPVLGPVR.[G]	1xDeamidated [Q]	13	184	202	2187,15
[KR].DMPIQAFLLYQEPVLGPVR.[G]	1xOxidation [M2]	17	184	202	2202,16
[KR].DMPIQAFLLYQEPVLGPVR.[G]	1xDeamidated [Q]; 1xOxidation [M2]	8	184	202	2203,15
[K-].IHPFAQTQSLVYPFPGPIPK.[S]		12	49	68	2237,21
[K].NHPFAQTQSLVYPFPGPIPK.[S]		1	49	68	2238,17
[K].IEKFQSEEQQQMEDELQDK.[I]	1xPhospho [S6]	1	30	48	2462,04
[K].SLPQNIPPLTQTPVVVPPFLQPEIMGVSK.[V]		3	69	97	3126,73
[-].RELEELNVPGEIVESLSSSEESITHINK.[K]	4xPhospho [S15; S17; S18; S19]	6	1	28	3458,45
**Kappa-casein OS=Bubalus bubalis OX=89462 GN=CSN3 PE=1 SV=2**					
[R].SPAQILQWQVLPNTVPAK.[FS]		8	69	86	1990,11
[R].YPSYGLNYYQQKPVALINNQFLPYPYYAKPAAVR.[S]		1	35	68	4010,06
[R].YPSYGLNYYQQKPVALINNQFLPYPYYAKPAAVR.[S]	3xDeamidated [N7; Q10; Q11]	1	35	68	4013,02

TU19

Description	Modifications	# PSMs	Start	End	Theor. MH+ [Da]
**Alpha-S1-casein (Fragment) OS=Bubalus bubalis GN=CSN1S PE=2 SV=1**					
[R].YLGYLEQLLR.[L]		41	91	100	1267,70
[R].FFVAPFPEVFGK.[E]		7	23	34	1384,73
[K].HQGLPQGVLNENLLR.[F]		16	8	22	1687,92
[K].HQGLPQGVLNENLLR.[F]	1xDeamidated [Q/N]	2	8	22	1688,91
[K].EPMIGVNQELAYFYPQLFR.[Q]		19	133	151	2315,15
[K].EPMIGVNQELAYFYPQLFR.[Q]	1xDeamidated [Q/N]	2	133	151	2316,14
[K].YNVPQLEIVPNLAEEQLHSMK.[E]		2	104	124	2452,25
[K].YNVPQLEIVPNLAEEQLHSMK.[E]	1xOxidation [M20]	1	104	124	2468,25
[K].KYNVPQLEIVPNLAEEQLHSMK.[E]		2	103	124	2580,35
[K].KYNVPQLEIVPNLAEEQLHSMK.[E]	1xDeamidated [N/Q]	2	103	124	2581,33
[K].VNELSTDIGSESTEDQAMEDIK.[Q]	2xPhospho [S10; T13]; 1xOxidation [M18]	1	37	58	2587,00
[K].KYNVPQLEIVPNLAEEQLHSMK.[E]	1xOxidation [M21]	2	103	124	2596,34
[K].KYNVPQLEIVPNLAEEQLHSMK.[E]	1xDeamidated [N3]; 1xOxidation [M21]	1	103	124	2597,33
**Beta-casein OS=Bubalus bubalis GN=CSN2 PE=2 SV=1**					
[-].RELEELNVPGEIVESLSSSEESITHINK.[K]	4xPhospho [S/T]	10	1	28	3458,45
[K].IEKFQSEEQQQMEDELQDK.[I]	1xPhospho [S6]	1	15	33	2462,04
[K].IEKFQSEEQQQMEDELQDK.[I]	1xDeamidated [Q5]; 1xPhospho [S6]	1	15	33	2463,03
[K].FQSEEQQQMEDELQDK.[I]		1	18	33	2011,85
[K].FQSEEQQQMEDELQDK.[I]	1xOxidation [M9]	1	18	33	2027,85
[K].FQSEEQQQMEDELQDK.[I]	1xDeamidated [Q2]; 1xOxidation [M9]	1	18	33	2028,83
[K].FQSEEQRQTEDELQDK.[I]	1xDeamidated [Q8]; 1xPhospho [S3]	1	18	33	2090,86
[K].FQSEEQQQMEDELQDK.[I]	1xPhospho [S3]	1	18	33	2091,82
[K].FQSEEQRQTEDELQDK.[I]	2xDeamidated [Q2; Q8]; 1xPhospho [S3]	1	18	33	2091,84
[K].FQSEEQQQMEDELQDK.[I]	1xDeamidated [Q]; 1xPhospho [S3]	3	18	33	2092,81
[K].FQSEEQQQMEDELQDK.[I]	2xDeamidated [Q]; 1xPhospho [S3]	8	18	33	2093,79
[K].FQSEEQQQMEDELQDK.[I]	1xPhospho [S3]; 1xOxidation [M9]	2	18	33	2107,82
[K].FQSEEQQQMEDELQDK.[I]	1xDeamidated [Q2]; 1xPhospho [S3]; 1xOxidation [M9]	1	18	33	2108,80
[K-].IHPFAQTQSLVYPFPGPIPK.[S]		9	34	53	2237,21
[K].SLPQNIPPLTQTPVVVPPFLQPEIMGVSK.[V]		9	54	82	3126,73
[KR].DMPIQAFLLYQEPVLGPVR.[G]		42	182	200	2186,17
[KR].DMPIQAFLLYQEPVLGPVR.[G]	1xDeamidated [Q]	8	182	200	2187,15
[KR].DMPIQAFLLYQEPVLGPVR.[G]	2xDeamidated [Q5; Q11]	1	182	200	2188,14
[KR].DMPIQAFLLYQEPVLGPVR.[G]	1xOxidation [M2]	19	182	200	2202,16
[KR].DMPIQAFLLYQEPVLGPVR.[G]	1xDeamidated [Q]; 1xOxidation [M2]	8	182	200	2203,15
**Beta-casein OS=Ovis aries OX=9940 GN=CSN2 PE=1 SV=3**					
[KR].DMPIQAFLLYQEPVLGPVR.[G]		42	182	200	2186,17
[KR].DMPIQAFLLYQEPVLGPVR.[G]	1xDeamidated [Q]	8	182	200	2187,15
[KR].DMPIQAFLLYQEPVLGPVR.[G]	2xDeamidated [Q5; Q11]	1	182	200	2188,14
[KR].DMPIQAFLLYQEPVLGPVR.[G]	1xOxidation [M2]	19	182	200	2202,16
[KR].DMPIQAFLLYQEPVLGPVR.[G]	1xDeamidated [Q]; 1xOxidation [M2]	8	182	200	2203,15
[K].LHLPLPLVQSWMHQPPQPLPPTVMFPPQSVLSLSQPK.[V]		1	133	169	4165,25
**Kappa-casein OS=Bubalus bubalis OX=89462 GN=CSN3 PE=1 SV=2**					
[R].YPSYGLNYYQQKPVALINNQFLPYPYYAKPAAVR.[S]	1xDeamidated [Q/N]	6	35	68	4011,05
[R].SPAQILQWQVLPNTVPAK.[FS]		10	69	86	1990,11
[R].SPAQILQWQVLPNTVPAK.[FS]	1xDeamidated [N13]	1	69	86	1991,10
**Beta-lactoglobulin variant D (Fragment) OS=Bos taurus OX=9913 GN=LGB PE=3 SV=1**					
[R].VYVEQLKPTPEGDLEILLQK.[W]		1	9	28	2312,27

TU20

Description	Modifications	# PSMs	Start	End	Theor. MH+ [Da]
**Alpha-S1-casein (Fragment) OS=Bubalus bubalis GN=CSN1S PE=2 SV=1**					
[K].HQGLPQGVLNENLLR.[F]		5	8	22	1687,92
[K].HQGLPQGVLNENLLR.[F]	1xDeamidated [Q/N]	5	8	22	1688,91
[K].HQGLPQGVLNENLLR.[F]	3xDeamidated [Q2; Q6; N]	5	8	22	1690,88
[K].HQGLPQGVLNENLLR.[F]	4xDeamidated [Q2; Q6; N10; N12]	5	8	22	1691,86
[R].FFVAPFPEVFGK.[E]		9	23	34	1384,73
[K].VNELSTDIGSESTEDQAMEDIK.[Q]	1xPhospho [S/T]	4	37	58	2491,04
[K].VNELSTDIGSESTEDQAMEDIK.[Q]	2xPhospho [S/T]	4	37	58	2571,01
[K].VNELSTDIGSESTEDQAMEDIK.[Q]	2xPhospho [S/T]; 1xOxidation [M18]	4	37	58	2587,00
[K].VNELSTDIGSESTEDQAMEDIK.[Q]	3xPhospho [S10; S/T]	4	37	58	2650,98
[R].YLGYLEQLLR.[L]		12	91	100	1267,70
[K].KYNVPQLEIVPNLAEEQLHSMK.[E]		5	103	124	2580,35
[K].KYNVPQLEIVPNLAEEQLHSMK.[E]	1xDeamidated [Q/N]	5	103	124	2581,33
[K].KYNVPQLEIVPNLAEEQLHSMK.[E]	1xDeamidated [Q/N]; 1xOxidation [M21]	5	103	124	2597,33
[K].YNVPQLEIVPNLAEEQLHSMK.[E]		5	104	124	2452,25
[K].YNVPQLEIVPNLAEEQLHSMK.[E]	1xDeamidated [N11]	5	104	124	2453,24
[K].YNVPQLEIVPNLAEEQLHSMK.[E]	1xOxidation [M20]	5	104	124	2468,25
[K].EPMIGVNQELAYFYPQLFR.[Q]		5	133	151	2315,15
[K].EPMIGVNQELAYFYPQLFR.[Q]	1xDeamidated [N7]	5	133	151	2316,14
[K].EPMIGVNQELAYFYPQLFR.[Q]	1xOxidation [M3]	5	133	151	2331,15
**AS2-casein (Fragment) OS=Bubalus bubalis PE=2 SV=2**					
[K].ALNEINQFYQK.[F]	1xDeamidated [N6]	10	81	91	1368,68
[K].KTVDMESTEVITK.[K]	1xPhospho [S7]	5	122	134	1560,72
[K].TVDMESTEVITK.[K]		5	123	134	1352,66
[K].KTVDMESTEVITK.[K]	1xPhospho [S7]; 1xOxidation [M5]	5	125	137	1576,72
**Beta-casein OS=Bubalus bubalis GN=CSN2 PE=2 SV=1**					
[-].RELEELNVPGEIVESLSSSEESITHINK.[K]	4xPhospho [S15; S17; S18; S19]	2	1	28	3458,45
[K].IEKFQSEEQQQMEDELQDK.[I]	1xPhospho [S6]	2	15	33	2462,04
[K].IEKFQSEEQQQMEDELQDK.[I]	1xDeamidated [Q5]; 1xPhospho [S6]	2	15	33	2463,03
[K].IEKFQSEEQQQMEDELQDK.[I]	1xPhospho [S6]; 1xOxidation [M12]	2	15	33	2478,04
[K].IEKFQSEEQQQMEDELQDK.[I]	1xDeamidated [Q5]; 1xPhospho [S6]; 1xOxidation [M12]	2	15	33	2479,02
[K].FQSEEQQQMEDELQDK.[I]		2	18	33	2011,85
[K].FQSEEQQQMEDELQDK.[I]	1xOxidation [M9]	2	18	33	2027,85
[K].FQSEEQQQMEDELQDK.[I]	1xPhospho [S3]	2	18	33	2091,82
[K].FQSEEQQQMEDELQDK.[I]	1xDeamidated [Q]; 1xPhospho [S3]	2	18	33	2092,81
[K].FQSEEQQQMEDELQDK.[I]	2xDeamidated [Q]; 1xPhospho [S3]	2	18	33	2093,79
[K].FQSEEQQQMEDELQDK.[I]	1xPhospho [S3]; 1xOxidation [M9]	2	18	33	2107,82
[K].FQSEEQQQMEDELQDK.[I]	1xDeamidated [Q]; 1xPhospho [S3]; 1xOxidation [M9]	2	18	33	2108,80
[K].FQSEEQQQMEDELQDK.[I]	3xDeamidated [Q]; 1xPhospho [S3]; 1xOxidation [M9]	2	18	33	2110,77
[K-].IHPFAQTQSLVYPFPGPIPK.[S]		4	34	53	2237,21
[K].SLPQNIPPLTQTPVVVPPFLQPEIMGVSK.[V]		9	54	82	3126,73
[KR].DMPIQAFLLYQEPVLGPVR.[G]		20	182	200	2186,17
[KR].DMPIQAFLLYQEPVLGPVR.[G]	1xDeamidated [Q]	20	182	200	2187,15
[KR].DMPIQAFLLYQEPVLGPVR.[G]	1xOxidation [M2]	20	182	200	2202,16
[KR].DMPIQAFLLYQEPVLGPVR.[G]	1xDeamidated [Q]; 1xOxidation [M2]	20	182	200	2203,15
**Beta-casein OS=Ovis aries OX=9940 GN=CSN2 PE=1 SV=3**					
[K].FQSEEQQQTEDELQDK.[I]		9	33	48	1981,86
[K].FQSEEQQQTEDELQDK.[I]	1xPhospho [S3]	9	33	48	2061,83
[K].YPVEPFTESQSLTLTDVEK.[L]		2	114	132	2183,08
[KR].DMPIQAFLLYQEPVLGPVR.[G]		20	182	200	2186,17
[KR].DMPIQAFLLYQEPVLGPVR.[G]	1xDeamidated [Q]	20	182	200	2187,15
[KR].DMPIQAFLLYQEPVLGPVR.[G]	1xOxidation [M2]	20	182	200	2202,16
[KR].DMPIQAFLLYQEPVLGPVR.[G]	1xDeamidated [Q]; 1xOxidation [M2]	20	182	200	2203,15
**Kappa-casein OS=Bubalus bubalis OX=89462 GN=CSN3 PE=1 SV=2**					
[R].SPAQILQWQVLPNTVPAK.[FS]		15	69	86	1990,11
[R].YPSYGLNYYQQKPVALINNQFLPYPYYAKPAAVR.[S]		22	41	74	4010,06
[R].YPSYGLNYYQQKPVALINNQFLPYPYYAKPAAVR.[S]	1xDeamidated [N/Q]	22	41	74	4011,05
**Beta-lactoglobulin OS=Bubalus bubalis GN=LGB PE=1 SV=2**					
[K-].VAGTWYSLAMAASDISLLDAQSAPLR.[V]		9	15	40	2707,38

TU21

Desccription	Modifications	# PSMs	Start	End	Theor. MH+ [Da]
**Alpha-S1-casein (Fragment) OS=Bubalus bubalis GN=CSN1S PE=2 SV=1**					
[K].VNELSTDIGSESTEDQAMEDIK.[Q]	1xPhospho [S10]	1	37	58	2491,04
[K].VNELSTDIGSESTEDQAMEDIK.[Q]	1xPhospho [T/S]; 1xOxidation [M18]	2	37	58	2507,04
[K].VNELSTDIGSESTEDQAMEDIK.[Q]	2xPhospho [S/T]	10	37	58	2571,01
[K].VNELSTDIGSESTEDQAMEDIK.[Q]	2xPhospho [S10; T13]; 1xOxidation [M18]	1	37	58	2587,00
**Beta-casein OS=Bubalus bubalis GN=CSN2 PE=2 SV=1**					
[-].RELEELNVPGEIVESLSSSEESITHINK.[K]	4xPhospho [S15; S17; S18; S19]	16	1	28	3458,45
[K].FQSEEQRQTEDELQDK.[I]	1xDeamidated [Q8]; 1xPhospho [S3]	2	33	33	2090,86
[K].FQSEEQQQMEDELQDK.[I]	1xDeamidated [Q]; 1xPhospho [S3]	16	33	33	2092,81
[K].FQSEEQQQMEDELQDK.[I]	4xDeamidated [Q2; Q]; 1xPhospho [S3]	2	33	33	2095,76
[K].FQSEEQQQMEDELQDK.[I]	1xPhospho [S3]; 1xOxidation [M9]	13	33	33	2107,82
[K].IEKFQSEEQQQMEDELQDK.[I]	1xPhospho [S6]	1	30	33	2462,04
[K].IEKFQSEEQQQMEDELQDK.[I]	1xDeamidated [Q]; 1xPhospho [S6]	2	30	33	2463,03
[K].IEKFQSEEQQQMEDELQDK.[I]	1xPhospho [S6]; 1xOxidation [M12]	1	30	33	2478,04

TU22

Description	Modifications	# PSMs	Start	End	Theor. MH+ [Da]
**Alpha-S1-casein (Fragment) OS=Bubalus bubalis GN=CSN1S PE=2 SV=1**					
[R].YLGYLEQLLR.[L]		1	91	100	1267,70
[R].FFVAPFPEVFGK.[E]		2	23	34	1384,73
[K].HQGLPQGVLNENLLR.[F]		23	8	22	1687,92
[K].HQGLPQGVLNENLLR.[F]	1xDeamidated [Q/N]	7	8	22	1688,91
[K].EPMIGVNQELAYFYPQLFR.[Q]		1	133	151	2315,15
[K].YNVPQLEIVPNLAEEQLHSMK.[E]		1	104	124	2452,25
[K].YNVPQLEIVPNLAEEQLHSMK.[E]	1xOxidation [M20]	1	104	124	2468,25
[K].VNELSTDIGSESTEDQAMEDIK.[Q]	1xPhospho [T/S]	2	37	58	2491,04
[K].VNELSTDIGSESTEDQAMEDIK.[Q]	1xDeamidated [N2]; 1xPhospho [T/S]	2	37	58	2492,03
[K].VNELSTDIGSESTEDQAMEDIK.[Q]	1xPhospho [S12]; 1xOxidation [M18]	1	37	58	2507,04
[K].VNELSTDIGSESTEDQAMEDIK.[Q]	2xPhospho [S10; S/T]	2	37	58	2571,01
[K].KYNVPQLEIVPNLAEEQLHSMK.[E]		1	103	124	2580,35
[K].VNELSTDIGSESTEDQAMEDIK.[Q]	2xPhospho [S10; T13]; 1xOxidation [M18]	2	37	58	2587,00
**AS2-casein (Fragment) OS=Bubalus bubalis PE=2 SV=2**					
[K].ALNEINQFYQK.[F]		3	81	91	1367,70
[K].ALNEINQFYQK.[F]	1xDeamidated [N/Q]	2	81	91	1368,68
[K].FPQYLQYLYQGPIVLNPWDQVK.[R]		2	92	113	2709,41
[K].KTVDMESTEVITK.[K]	1xPhospho [S7]	1	125	137	1560,72
[K].TVDMESTEVITK.[K]	1xPhospho [T/S]	2	123	134	1432,63
**Beta-casein OS=Bubalus bubalis GN=CSN2 PE=2 SV=1**					
[K].FQSEEQQQMEDELQDK.[I]		1	33	48	2011,85
[K].FQSEEQQQMEDELQDK.[I]	1xOxidation [M9]	1	33	48	2027,85
[K].FQSEEQQQTEDELQDK.[I]	1xPhospho [S3]	1	33	48	2061,83
[K].FQSEEQQQMEDELQDK.[I]	1xPhospho [S3]	49	33	48	2091,82
[K].FQSEEQQQMEDELQDK.[I]	1xDeamidated [Q]; 1xPhospho [S3]	19	33	48	2092,81
[K].FQSEEQQQMEDELQDK.[I]	1xDeamidated [Q]; 1xPhospho [S3]	5	33	48	2092,81
[K].FQSEEQQQMEDELQDK.[I]	3xDeamidated [Q]; 1xPhospho [S3]	8	33	48	2094,77
[K].FQSEEQQQMEDELQDK.[I]	4xDeamidated [Q2; Q]; 1xPhospho [S3]	3	33	48	2095,76
[K].FQSEEQQQMEDELQDK.[I]	1xPhospho [S3]; 1xOxidation [M9]	16	33	48	2107,82
[K].FQSEEQQQMEDELQDK.[I]	1xDeamidated [Q2]; 1xPhospho [S3]; 1xOxidation [M9]	6	33	48	2108,80
[KR].DMPIQAFLLYQEPVLGPVR.[G]		7	182	200	2186,17
[KR].DMPIQAFLLYQEPVLGPVR.[G]	1xDeamidated [Q11]	1	182	200	2187,15
[KR].DMPIQAFLLYQEPVLGPVR.[G]	1xDeamidated [Q]; 1xOxidation [M2]	2	182	200	2203,15
[K-].IHPFAQTQSLVYPFPGPIPK.[S]		3	49	68	2237,21
[K].IEKFQSEEQQQMEDELQDK.[I]	1xPhospho [S6]	1	30	48	2462,04
[K].IEKFQSEEQQQMEDELQDK.[I]	1xDeamidated [Q5]; 1xPhospho [S6]	1	30	48	2463,03
[K].SLPQNIPPLTQTPVVVPPFLQPEIMGVSK.[V]		4	69	97	3126,73
[-].RELEELNVPGEIVESLSSSEESITHINK.[K]	4xPhospho [S15; S17; S18; S19]	10	1	28	3458,45
**Kappa-casein OS=Bubalus bubalis OX=89462 GN=CSN3 PE=1 SV=2**					
[R].SPAQILQWQVLPNTVPAK.[FS]		1	69	86	1990,11
[R].YPSYGLNYYQQKPVALINNQFLPYPYYAKPAAVR.[S]		1	35	68	4010,06
[R].YPSYGLNYYQQKPVALINNQFLPYPYYAKPAAVR.[S]	1xDeamidated [N/Q]	3	35	68	4011,05
**Beta-lactoglobulin OS=Bubalus bubalis GN=LGB PE=1 SV=2**					
[R].TPEVDDEALEK.[F]		1	125	135	1245,58
[R].TPEVDDEALEKFDK.[A]		1	128	141	1635,77
[R].VYVEELKPTPEGDLEILLQK.[W]		1	44	63	2313,26

TU23

Description	Modifications	# PSMs	Start	End	Theor. MH+ [Da]
**Alpha-S1-casein (Fragment) OS=Bubalus bubalis GN=CSN1S PE=2 SV=1**					
[R].YLGYLEQLLR.[L]		35	91	100	1267,70
[K].HQGLPQGVLNENLLR.[F]		21	8	22	1687,92
[K].HQGLPQGVLNENLLR.[F]	1xDeamidated [Q/N]	15	8	22	1688,91
[K].EPMIGVNQELAYFYPQLFR.[Q]		3	133	151	2315,15
[K].YNVPQLEIVPNLAEEQLHSMK.[E]		3	104	124	2452,25
[K].YNVPQLEIVPNLAEEQLHSMK.[E]	3xDeamidated [N2; N11; Q16]	1	104	124	2455,21
[K].YNVPQLEIVPNLAEEQLHSMK.[E]	1xOxidation [M20]	2	104	124	2468,25
[K].VNELSTDIGSESTEDQAMEDIK.[Q]	1xPhospho [S10]	1	37	58	2491,04
[K].VNELSTDIGSESTEDQAMEDIK.[Q]	1xPhospho [S12]; 1xOxidation [M18]	1	37	58	2507,04
[K].VNELSTDIGSESTEDQAMEDIK.[Q]	2xPhospho [T/S]	6	37	58	2571,01
[K].KYNVPQLEIVPNLAEEQLHSMK.[E]		1	103	124	2580,35
[K].VNELSTDIGSESTEDQAMEDIK.[Q]	2xPhospho [S10; T13]; 1xOxidation [M18]	2	37	58	2587,00
[K].EKVNELSTDIGSESTEDQAMEDIK.[Q]	1xPhospho [S12]	1	35	58	2748,18
**Alpha s2-casein OS=Bubalus bubalis PE=2 SV=1**					
[K].ALNEINQFYQK.[F]		3	81	91	1367,70
[K].ALNEINQFYQK.[F]	1xDeamidated [N6]	1	81	91	1368,68
[K].FPQYLQYLYQGPIVLNPWDQVK.[R]		2	92	113	2709,41
[K].KTVDMESTEVITK.[K]	1xPhospho [T/S]	2	122	134	1560,72
[K].TVDMESTEVITK.[K]	1xPhospho [S/T]	2	123	134	1432,63
[K].TVDMESTEVITK.[K]	1xPhospho [S/T]	2	126	137	1432,63
**Beta-casein OS=Bubalus bubalis GN=CSN2 PE=2 SV=1**					
[K].FQSEEQQQMEDELQDK.[I]		2	33	48	2011,85
[K].FQSEEQQQMEDELQDK.[I]	1xOxidation [M9]	1	33	48	2027,85
[K].FQSEEQQQTEDELQDK.[I]	1xPhospho [S3]	1	33	48	2061,83
[K].FQSEEQQQMEDELQDK.[I]	1xPhospho [S3]	46	33	48	2091,82
[K].FQSEEQQQMEDELQDK.[I]	1xDeamidated [Q]; 1xPhospho [S3]	3	33	48	2092,81
[K].FQSEEQQQMEDELQDK.[I]	2xDeamidated [Q]; 1xPhospho [S3]	7	33	48	2093,79
[K].FQSEEQQQMEDELQDK.[I]	3xDeamidated [Q2; Q]; 1xPhospho [S3]	3	33	48	2094,77
[K].FQSEEQQQMEDELQDK.[I]	1xPhospho [S3]; 1xOxidation [M9]	5	33	48	2107,82
[K].FQSEEQQQMEDELQDK.[I]	1xDeamidated [Q]; 1xPhospho [S3]; 1xOxidation [M9]	3	33	48	2108,80
[K].FQSEEQQQMEDELQDK.[I]	2xDeamidated [Q2; Q]; 1xPhospho [S3]; 1xOxidation [M9]	2	33	48	2109,78
[K].FQSEEQQQMEDELQDK.[I]	4xDeamidated [Q2; Q]; 1xPhospho [S3]; 1xOxidation [M9]	3	33	48	2111,75
[KR].DMPIQAFLLYQEPVLGPVR.[G]		7	182	200	2186,17
[KR].DMPIQAFLLYQEPVLGPVR.[G]	1xDeamidated [Q11]	1	182	200	2187,15
[KR].DMPIQAFLLYQEPVLGPVR.[G]	1xOxidation [M2]	1	182	200	2202,16
[K-].IHPFAQTQSLVYPFPGPIPK.[S]		5	49	68	2237,21
[K-].IHPFAQTQSLVYPFPGPIPK.[S]	1xDeamidated [Q]	2	49	68	2238,20
[K].IEKFQSEEQQQMEDELQDK.[I]	1xPhospho [S6]	1	30	48	2462,04
[K].IEKFQSEEQQQMEDELQDK.[I]	1xDeamidated [Q5]; 1xPhospho [S6]	1	30	48	2463,03
[K].SLPQNIPPLTQTPVVVPPFLQPEIMGVSK.[V]		8	69	97	3126,73
[-].RELEELNVPGEIVESLSSSEESITHINK.[K]	4xPhospho [S/T]	16	1	28	3458,45
**Kappa-casein OS=Bubalus bubalis OX=89462 GN=CSN3 PE=1 SV=2**					
[R].SPAQILQWQVLPNTVPAK.[FS]		3	69	86	1990,11
[R].YPSYGLNYYQQKPVALINNQFLPYPYYAKPAAVR.[S]		1	35	68	4010,06
**Beta-lactoglobulin OS=Capra hircus OX=9925 GN=LGB PE=1 SV=2**					
[R].VYVEELKPTPEGNLEILLQK.[W]		1	41	60	2312,27
**Alpha-lactalbumin protein variant D OS=Bos taurus OX=9913 GN=LALBA PE=3 SV=1**					
[K].VGINYWLAHK.[A]		1	99	108	1200,65

TU24

Description	Modifications	# PSMs	Start	End	Theor. MH+ [Da]
**Alpha-S1-casein (Fragment) OS=Bubalus bubalis GN=CSN1S PE=2 SV=1**					
[K].HQGLPQGVLNENLLR.[F]		26	8	22	1687,92
[K].HQGLPQGVLNENLLR.[F]	1xDeamidated [N/Q]	11	8	22	1688,91
[R].FFVAPFPEVFGK.[E]		1	23	34	1384,73
[K].VNELSTDIGSESTEDQAMEDIK.[Q]	2xPhospho [S10; S12]	1	37	58	2571,01
[R].YLGYLEQLLR.[L]		46	91	100	1267,70
[K].KYNVPQLEIVPNLAEEQLHSMK.[E]		1	103	124	2580,35
[K].YNVPQLEIVPNLAEEQLHSMK.[E]	1xOxidation [M20]	1	104	124	2468,25
[K].EPMIGVNQELAYFYPQLFR.[Q]	1xDeamidated [N7]	1	133	151	2316,14
**AS2-casein (Fragment) OS=Bubalus bubalis PE=2 SV=2**					
[K].ALNEINQFYQK.[F]		1	81	91	1367,70
[K].FPQYLQYLYQGPIVLNPWDQVK.[R]		2	92	113	2709,41
[K].KTVDMESTEVITK.[K]	1xPhospho [S7]; 1xOxidation [M5]	1	125	137	1576,72
[K].TVDMESTEVITK.[K]		1	126	137	1352,66
[K].TVDMESTEVITK.[K]	1xPhospho [S6]	1	126	137	1432,63
**Beta-casein OS=Bubalus bubalis GN=CSN2 PE=2 SV=1**					
[K].FQSEEQQQMEDELQDK.[I]		1	33	48	2011,85
[K].FQSEEQQQMEDELQDK.[I]	3xDeamidated [Q14; Q]	5	33	48	2014,81
[K].FQSEEQQQMEDELQDK.[I]	1xOxidation [M9]	1	33	48	2027,85
[K].FQSEEQQQMEDELQDK.[I]	1xDeamidated [Q]; 1xOxidation [M9]	3	33	48	2028,83
[K].FQSEEQQQMEDELQDK.[I]	1xPhospho [S3]	4	33	48	2091,82
[K].FQSEEQQQMEDELQDK.[I]	1xDeamidated [Q]; 1xPhospho [S3]	5	33	48	2092,81
[K].FQSEEQQQMEDELQDK.[I]	1xPhospho [S3]; 1xOxidation [M9]	1	33	48	2107,82
[KR].DMPIQAFLLYQEPVLGPVR.[G]		5	184	202	2186,17
[K-].IHPFAQTQSLVYPFPGPIPK.[S]		6	49	68	2237,21
[K-].IHPFAQTQSLVYPFPGPIPK.[S]	1xDeamidated [Q]	2	49	68	2238,20
[K].IEKFQSEEQQQMEDELQDK.[I]	1xPhospho [S6]	1	30	48	2462,04
[K].SLPQNIPPLTQTPVVVPPFLQPEIMGVSK.[V]		5	69	97	3126,73
[-].RELEELNVPGEIVESLSSSEESITHINK.[K]	4xPhospho [S15; S/T]	9	1	28	3458,45
[-].RELEELNVPGEIVESLSSSEESITHINK.[K]	1xDeamidated [N27]; 4xPhospho [S15; S17; S22; T24]	1	1	28	3459,43
**Kappa-casein OS=Bubalus bubalis OX=89462 GN=CSN3 PE=1 SV=2**					
[R].SPAQILQWQVLPNTVPAK.[FS]		1	69	86	1990,11
[R].YPSYGLNYYQQKPVALINNQFLPYPYYAKPAAVR.[S]		1	35	68	4010,06
[R].YPSYGLNYYQQKPVALINNQFLPYPYYAKPAAVR.[S]	1xDeamidated [Q/N]	5	35	68	4011,05

TU25

Description	Modifications	# PSMs	Start	End	Theor. MH+ [Da]
**Alpha-S1-casein (Fragment) OS=Bubalus bubalis GN=CSN1S PE=2 SV=1**					
[K].HQGLPQGVLNENLLR.[F]	1xDeamidated [Q/N]	11	8	22	1688,91
[K].HQGLPQGVLNENLLR.[F]	3xDeamidated [Q2; Q6; N]	2	8	22	1690,88
[R].FFVAPFPEVFGK.[E]		1	23	34	1384,73
[K].VNELSTDIGSESTEDQAMEDIK.[Q]	1xPhospho [S10]	1	37	58	2491,04
[K].VNELSTDIGSESTEDQAMEDIK.[Q]	1xPhospho [S12]; 1xOxidation [M18]	1	37	58	2507,04
[K].VNELSTDIGSESTEDQAMEDIK.[Q]	2xPhospho [S/T]	10	37	58	2571,01
[R].YLGYLEQLLR.[L]		46	91	100	1267,70
[K].KYNVPQLEIVPNLAEEQLHSMK.[E]		1	103	124	2580,35
[K].YKVPQLEIVPNSAEER.[L]	1xPhospho [S12]	1	104	119	1951,95
[K].YNVPQLEIVPNLAEEQLHSMK.[E]	1xOxidation [M20]	1	104	124	2468,25
[K].EPMIGVNQELAYFYPELFR.[Q]		1	133	151	2316,14
**Alpha s2-casein OS=Bubalus bubalis PE=2 SV=1**					
[K].TVDMESTEVITK.[K]		1	123	134	1352,66
[K].ALNEINQFYQK.[F]		9	81	91	1367,70
[K].ALNEINQFYQK.[F]	1xDeamidated [N]	2	81	91	1368,68
[K].TVDMESTEVITK.[K]	1xPhospho [S6]	1	123	134	1432,63
[K].TVDMESTEVITKK.[T]	1xPhospho [S6]	1	123	135	1560,72
[K].KTVDMESTEVITK.[K]	1xPhospho [S7]; 1xOxidation [M5]	1	122	134	1576,72
**Beta-casein OS=Bubalus bubalis GN=CSN2 PE=2 SV=1**					
[K].FQSEEQQQMEDELQDK.[I]		1	33	48	2011,85
[K].FQSEEQQQMEDELQDK.[I]	1xOxidation [M9]	1	33	48	2027,85
[K].FQSEEQRQTEDELQDK.[I]	1xDeamidated [Q]; 1xPhospho [S3]	4	33	48	2090,86
[K].FQSEEQRQTEDELQDK.[I]	2xDeamidated [Q2; Q8]; 1xPhospho [S3]	1	33	48	2091,84
[K].FQSEEQQQMEDELQDK.[I]	1xDeamidated [Q]; 1xPhospho [S3]	10	33	48	2092,81
[K].FQSEEQQQMEDELQDK.[I]	2xDeamidated [Q]; 1xPhospho [S3]	2	33	48	2093,79
[K].FQSEEQQQMEDELQDK.[I]	3xDeamidated [Q2; Q]; 1xPhospho [S3]	4	33	48	2094,77
[K].FQSEEQQQMEDELQDK.[I]	1xPhospho [S3]; 1xOxidation [M9]	18	33	48	2107,82
[K].FQSEEQQQMEDELQDK.[I]	1xDeamidated [Q]; 1xPhospho [S3]; 1xOxidation [M9]	10	33	48	2108,80
[KR].DMPIQAFLLYQEPVLGPVR.[G]		1	184	202	2186,17
[KR].DMPIQAFLLYQEPVLGPVR.[G]	1xDeamidated [Q]	2	184	202	2187,15
[KR].DMPIQAFLLYQEPVLGPVR.[G]	1xDeamidated [Q5]; 1xOxidation [M2]	1	184	202	2203,15
[K-].IHPFAQTQSLVYPFPGPIPK.[S]		6	49	68	2237,21
[K].NHPFAQTQSLVYPFPGPIPK.[S]		1	49	68	2238,17
[K].SLPQNIPPLTQTPVVVPPFLQPEIMGVSK.[V]		11	69	97	3126,73
[R].ELEELNVPGEIVESLSSSEESITHINK.[K]	4xPhospho [S14; S/T]	3	2	28	3302,34
[-].RELEELNVPGEIVESLSSSEESITHINK.[K]	4xPhospho [S15; S17; S18; S19]	14	1	28	3458,45
**Kappa-casein OS=Bubalus bubalis OX=89462 GN=CSN3 PE=1 SV=2**					
[R].SPAQILQWQVLPNTVPAK.[FS]		1	75	92	1990,11
[R].SPAQILQWQVLPNTVPAK.[FS]	1xDeamidated [Q/N]	2	75	92	1991,10
[R].YPSYGLNYYQQKPVALINNQFLPYPYYAKPAAVR.[S]		1	41	74	4010,06
**Beta-lactoglobulin variant D (Fragment) OS=Bos taurus OX=9913 GN=LGB PE=3 SV=1**					
[K-].VAGTWYSLAMAASDISLLDAQSAPLR.[V]		1	18	40	2707,38
[R].VYVEQLKPTPEGDLEILLQK.[W]	1xDeamidated [Q5]	1	41	60	2313,26

Supplementary Material Table S5. Complete list of peptides identified by nano-LC-ESI-MS/MS in the 10 kDa permeate of the pH 4.6 water-soluble fraction extracted from a 65-day-old buffalo blue cheese. This sample was investigated for the occurrence of putative adulteration with bovine milk. Information reported includes protein accession code, peptide sequence, experimental mass value, localization within the protein sequence and intensity value. This analysis enabled quantification of adulteration with bovine milk based on signal intensity ratio of proteotypic β-casein A1 peptides relative to the corresponding buffalo counterparts.

Protein	Sequence	Mass	Start	End	Intensity
sp|P02662|CASA1_BOVIN	RPKHPIKHQGLPQ	1534,88	1	13	1,47E+07
sp|P02662|CASA1_BOVIN	RPKHPIKHQGLPQE	1663,93	1	14	2,82E+06
sp|P02662|CASA1_BOVIN	GLPQEV	641,34	10	15	2,07E+06
sp|P02662|CASA1_BOVIN	RPKHPIKHQGLPQEVL	1876,08	1	16	3,08E+06
sp|P02662|CASA1_BOVIN	HQGLPQEVLN	1133,58	8	17	3,07E+06
sp|P02662|CASA1_BOVIN	RPKHPIKHQGLPQEVLN	1990,12	1	17	4,27E+07
sp|P02662|CASA1_BOVIN	RPKHPIKHQGLPQEVLNEN	2233,21	1	19	2,05E+06
sp|P02662|CASA1_BOVIN	EVLNENLLR	1098,60	14	22	2,16E+06
sp|P02662|CASA1_BOVIN	GKEKVNELSK	1130,63	33	42	3,10E+06
sp|P02662|CASA1_BOVIN	SKDIGSESTEDQAME	1625,67	41	55	2,00E+06
sp|P02662|CASA1_BOVIN	KYKVPQ	761,44	103	108	2,10E+06
sp|P02662|CASA1_BOVIN	IVPNSAEE	857,41	111	118	1,15E+06
sp|P02662|CASA1_BOVIN	KVPQLEIVPNSAEE	1551,81	105	118	1,18E+06
sp|P02662|CASA1_BOVIN	QLEIVPNSAEERLHSMKEGIHAQQK	2871,47	108	132	1,67E+06
sp|O62823|CASA1_BUBBU	RPKQPIK	865,55	1	7	1,51E+07
sp|O62823|CASA1_BUBBU	RPKQPIKH	1002,61	1	8	3,17E+06
sp|O62823|CASA1_BUBBU	RPKQPIKHQGLP	1397,83	1	12	0,00E+00
sp|O62823|CASA1_BUBBU	QGLPQ	541,29	9	13	0,00E+00
sp|O62823|CASA1_BUBBU	HQGLPQ	678,34	8	13	1,57E+07
sp|O62823|CASA1_BUBBU	KHQGLPQ	806,44	7	13	1,86E+07
sp|O62823|CASA1_BUBBU	PIKHQGLPQ	1016,58	5	13	2,25E+07
sp|O62823|CASA1_BUBBU	QPIKHQGLPQ	1144,64	4	13	0,00E+00
sp|O62823|CASA1_BUBBU	KQPIKHQGLPQ	1272,73	3	13	6,24E+07
sp|O62823|CASA1_BUBBU	RPKQPIKHQGLPQ	1525,88	1	13	1,41E+08
sp|O62823|CASA1_BUBBU	GLPQGV	569,32	10	15	7,09E+06
sp|O62823|CASA1_BUBBU	RPKQPIKHQGLPQGV	1681,97	1	15	1,08E+07
sp|O62823|CASA1_BUBBU	RPKQPIKHQGLPQGVL	1795,06	1	16	2,02E+07
sp|O62823|CASA1_BUBBU	RPKQPIKHQGLPQGVLN	1909,10	1	17	2,43E+07
sp|O62823|CASA1_BUBBU	QGVLNE	658,33	13	18	1,76E+07
sp|O62823|CASA1_BUBBU	RPKQPIKHQGLPQGVLNE	2038,14	1	18	8,90E+06
sp|O62823|CASA1_BUBBU	QGVLNENL	885,46	13	20	1,70E+06
sp|O62823|CASA1_BUBBU	RPKQPIKHQGLPQGVLNENL	2265,27	1	20	1,71E+07
sp|O62823|CASA1_BUBBU	NENLL	601,31	17	21	3,67E+06
sp|O62823|CASA1_BUBBU	GVLNENLL	870,48	14	21	2,10E+07
sp|O62823|CASA1_BUBBU	PQGVLNENLL	1095,59	12	21	1,68E+07
sp|O62823|CASA1_BUBBU	LPQGVLNENLL	1208,68	11	21	4,68E+06
sp|O62823|CASA1_BUBBU	NENLLR	757,41	17	22	2,87E+07
sp|O62823|CASA1_BUBBU	VLNENLLR	969,56	15	22	1,40E+07
sp|O62823|CASA1_BUBBU	GVLNENLLR	1026,58	14	22	2,85E+08
sp|O62823|CASA1_BUBBU	QGVLNENLLR	1154,64	13	22	7,12E+06
sp|O62823|CASA1_BUBBU	ENLLRF	790,43	18	23	5,67E+07
sp|O62823|CASA1_BUBBU	NENLLRF	904,48	17	23	1,21E+06
sp|O62823|CASA1_BUBBU	LNENLLRF	1017,56	16	23	2,58E+06
sp|O62823|CASA1_BUBBU	VLNENLLRF	1116,63	15	23	1,58E+06
sp|O62823|CASA1_BUBBU	GVLNENLLRF	1173,65	14	23	5,39E+07
sp|O62823|CASA1_BUBBU	FVAPFP	676,36	24	29	3,65E+06
sp|O62823|CASA1_BUBBU	VAPFPEV	757,40	25	31	6,97E+05
sp|O62823|CASA1_BUBBU	FPEVF	637,31	28	32	1,48E+07
sp|O62823|CASA1_BUBBU	VAPFPEVF	904,47	25	32	5,40E+06
sp|O62823|CASA1_BUBBU	FVAPFPEVF	1051,54	24	32	5,39E+06
sp|O62823|CASA1_BUBBU	PEVFG	547,26	29	33	3,12E+07
sp|O62823|CASA1_BUBBU	APFPEVFG	862,42	26	33	0,00E+00
sp|O62823|CASA1_BUBBU	FPEVFGK	822,43	28	34	5,25E+08
sp|O62823|CASA1_BUBBU	APFPEVFGK	990,52	26	34	5,64E+07
sp|O62823|CASA1_BUBBU	VAPFPEVFGK	1089,59	25	34	4,69E+07
sp|O62823|CASA1_BUBBU	FVAPFPEVFGK	1236,65	24	34	3,80E+07
sp|O62823|CASA1_BUBBU	FFVAPFPEVFGK	1383,72	23	34	6,69E+06
sp|O62823|CASA1_BUBBU	VFGKE	578,31	31	35	3,11E+07
sp|O62823|CASA1_BUBBU	EVFGKE	707,35	30	35	1,33E+07
sp|O62823|CASA1_BUBBU	PEVFGKE	804,40	29	35	2,91E+07
sp|O62823|CASA1_BUBBU	FPEVFGKE	951,47	28	35	3,72E+07
sp|O62823|CASA1_BUBBU	VAPFPEVFGKE	1218,63	25	35	6,20E+06
sp|O62823|CASA1_BUBBU	FVAPFPEVFGKE	1365,70	24	35	6,45E+06
sp|O62823|CASA1_BUBBU	EVFGKEK	835,44	30	36	5,75E+07
sp|O62823|CASA1_BUBBU	PEVFGKEK	932,50	29	36	7,10E+06
sp|O62823|CASA1_BUBBU	FPEVFGKEK	1079,57	28	36	1,08E+08
sp|O62823|CASA1_BUBBU	PFPEVFGKEK	1176,62	27	36	0,00E+00
sp|O62823|CASA1_BUBBU	APFPEVFGKEK	1247,66	26	36	9,34E+07
sp|O62823|CASA1_BUBBU	VAPFPEVFGKEK	1346,72	25	36	4,90E+07
sp|O62823|CASA1_BUBBU	FVAPFPEVFGKEK	1493,79	24	36	6,37E+07
sp|O62823|CASA1_BUBBU	FFVAPFPEVFGKEK	1640,86	23	36	8,38E+06
sp|O62823|CASA1_BUBBU	VFGKEKV	805,47	31	37	1,17E+08
sp|O62823|CASA1_BUBBU	EVFGKEKV	934,51	30	37	3,45E+07
sp|O62823|CASA1_BUBBU	PEVFGKEKV	1031,57	29	37	1,22E+07
sp|O62823|CASA1_BUBBU	FPEVFGKEKV	1178,63	28	37	2,49E+08
sp|O62823|CASA1_BUBBU	PFPEVFGKEKV	1275,69	27	37	5,02E+06
sp|O62823|CASA1_BUBBU	APFPEVFGKEKV	1346,72	26	37	2,59E+08
sp|O62823|CASA1_BUBBU	VAPFPEVFGKEKV	1445,79	25	37	1,26E+08
sp|O62823|CASA1_BUBBU	FVAPFPEVFGKEKV	1592,86	24	37	9,32E+07
sp|O62823|CASA1_BUBBU	FGKEKVN	820,44	32	38	2,38E+08
sp|O62823|CASA1_BUBBU	VFGKEKVN	919,51	31	38	3,15E+08
sp|O62823|CASA1_BUBBU	EVFGKEKVN	1048,56	30	38	4,25E+08
sp|O62823|CASA1_BUBBU	PEVFGKEKVN	1145,61	29	38	4,49E+07
sp|O62823|CASA1_BUBBU	FPEVFGKEKVN	1292,68	28	38	3,14E+08

sp|O62823|CASA1_BUBBU	PFPEVFGKEKVN	1389,73	27	38	2,64E+06
sp|O62823|CASA1_BUBBU	APFPEVFGKEKVN	1460,77	26	38	1,15E+08
sp|O62823|CASA1_BUBBU	VAPFPEVFGKEKVN	1559,83	25	38	1,41E+08
sp|O62823|CASA1_BUBBU	FVAPFPEVFGKEKVN	1706,90	24	38	1,54E+08
sp|O62823|CASA1_BUBBU	FFVAPFPEVFGKEKVN	1853,97	23	38	7,50E+06
sp|O62823|CASA1_BUBBU	FGKEKVNE	949,49	32	39	4,59E+07
sp|O62823|CASA1_BUBBU	VFGKEKVNE	1048,56	31	39	1,22E+08
sp|O62823|CASA1_BUBBU	EVFGKEKVNE	1177,60	30	39	1,51E+08
sp|O62823|CASA1_BUBBU	PEVFGKEKVNE	1274,65	29	39	2,80E+06
sp|O62823|CASA1_BUBBU	FPEVFGKEKVNE	1421,72	28	39	9,71E+07
sp|O62823|CASA1_BUBBU	PFPEVFGKEKVNE	1518,77	27	39	0,00E+00
sp|O62823|CASA1_BUBBU	APFPEVFGKEKVNE	1589,81	26	39	4,70E+07
sp|O62823|CASA1_BUBBU	VAPFPEVFGKEKVNE	1688,88	25	39	3,52E+06
sp|O62823|CASA1_BUBBU	FVAPFPEVFGKEKVNE	1835,95	24	39	4,42E+07
sp|O62823|CASA1_BUBBU	EKVNEL	730,39	35	40	5,54E+07
sp|O62823|CASA1_BUBBU	KEKVNEL	858,48	34	40	2,47E+08
sp|O62823|CASA1_BUBBU	GKEKVNEL	915,50	33	40	3,55E+08
sp|O62823|CASA1_BUBBU	FGKEKVNEL	1062,57	32	40	1,18E+08
sp|O62823|CASA1_BUBBU	VFGKEKVNEL	1161,64	31	40	2,73E+08
sp|O62823|CASA1_BUBBU	EVFGKEKVNEL	1290,68	30	40	2,06E+08
sp|O62823|CASA1_BUBBU	PEVFGKEKVNEL	1387,73	29	40	5,85E+07
sp|O62823|CASA1_BUBBU	FPEVFGKEKVNEL	1534,80	28	40	1,05E+09
sp|O62823|CASA1_BUBBU	PFPEVFGKEKVNEL	1631,86	27	40	5,67E+06
sp|O62823|CASA1_BUBBU	APFPEVFGKEKVNEL	1702,89	26	40	2,62E+08
sp|O62823|CASA1_BUBBU	VAPFPEVFGKEKVNEL	1801,96	25	40	4,68E+08
sp|O62823|CASA1_BUBBU	FVAPFPEVFGKEKVNEL	1949,03	24	40	5,48E+08
sp|O62823|CASA1_BUBBU	FFVAPFPEVFGKEKVNEL	2096,10	23	40	9,52E+06
sp|O62823|CASA1_BUBBU	GKEKVNELS	1002,53	33	41	4,47E+06
sp|O62823|CASA1_BUBBU	KEKVNELST	1046,56	34	42	5,40E+06
sp|O62823|CASA1_BUBBU	GKEKVNELST	1103,58	33	42	2,55E+07
sp|O62823|CASA1_BUBBU	FPEVFGKEKVNELSK	1749,93	28	42	1,66E+06
sp|O62823|CASA1_BUBBU	GKEKVNELSTD	1218,61	33	43	0,00E+00
sp|O62823|CASA1_BUBBU	ELSTDIGSE	949,42	39	47	1,35E+06
sp|O62823|CASA1_BUBBU	IGSEST	592,27	44	49	2,48E+06
sp|O62823|CASA1_BUBBU	IGSESTE	721,31	44	50	1,12E+08
sp|O62823|CASA1_BUBBU	IGSESTEDQ	964,40	44	52	9,53E+07
sp|O62823|CASA1_BUBBU	IGSESTEDQA	1035,44	44	53	0,00E+00
sp|O62823|CASA1_BUBBU	GSESTEDQAM	1053,39	45	54	0,00E+00
sp|O62823|CASA1_BUBBU	IGSESTEDQAM	1166,48	44	54	1,40E+08
sp|O62823|CASA1_BUBBU	TDIGSESTEDQAM	1382,55	42	54	0,00E+00
sp|O62823|CASA1_BUBBU	SESTEDQAME	1125,41	46	55	8,30E+06
sp|O62823|CASA1_BUBBU	GSESTEDQAME	1182,43	45	55	0,00E+00
sp|O62823|CASA1_BUBBU	IGSESTEDQAME	1295,52	44	55	4,03E+08
sp|O62823|CASA1_BUBBU	DIGSESTEDQAME	1410,55	43	55	3,88E+07
sp|O62823|CASA1_BUBBU	TDIGSESTEDQAME	1511,59	42	55	1,86E+07
sp|O62823|CASA1_BUBBU	STDIGSESTEDQAME	1598,63	41	55	0,00E+00
sp|O62823|CASA1_BUBBU	ELSTDIGSESTEDQAME	1840,75	39	55	0,00E+00
sp|O62823|CASA1_BUBBU	NELSTDIGSESTEDQAME	1954,80	38	55	1,03E+06
sp|O62823|CASA1_BUBBU	GKEKVNELSTDIGSESTEDQAME	2496,12	33	55	0,00E+00
sp|O62823|CASA1_BUBBU	IGSESTEDQAMED	1410,55	44	56	0,00E+00
sp|O62823|CASA1_BUBBU	MEDIK	634,30	54	58	2,65E+06
sp|O62823|CASA1_BUBBU	DQAMEDIK	948,42	51	58	2,14E+07
sp|O62823|CASA1_BUBBU	TEDQAMEDIK	1178,51	49	58	3,58E+06
sp|O62823|CASA1_BUBBU	IGSESTEDQAMEDIK	1651,72	44	58	1,05E+07
sp|O62823|CASA1_BUBBU	DIGSESTEDQAMEDIK	1766,75	43	58	1,19E+06
sp|O62823|CASA1_BUBBU	TDIGSESTEDQAMEDIK	1867,80	42	58	0,00E+00
sp|O62823|CASA1_BUBBU	STDIGSESTEDQAMEDIK	1954,83	41	58	0,00E+00
sp|O62823|CASA1_BUBBU	LSTDIGSESTEDQAMEDIK	2067,92	40	58	0,00E+00
sp|O62823|CASA1_BUBBU	ELSTDIGSESTEDQAMEDIK	2196,96	39	58	0,00E+00
sp|O62823|CASA1_BUBBU	GKEKVNELSTDIGSESTEDQAMEDIK	2852,32	33	58	0,00E+00
sp|O62823|CASA1_BUBBU	EDIKQ	631,32	55	59	2,34E+06
sp|O62823|CASA1_BUBBU	AMEDIKQ	833,40	53	59	1,28E+08
sp|O62823|CASA1_BUBBU	DQAMEDIKQ	1076,48	51	59	1,98E+08
sp|O62823|CASA1_BUBBU	TEDQAMEDIKQ	1306,57	49	59	5,52E+06
sp|O62823|CASA1_BUBBU	STEDQAMEDIKQ	1393,60	48	59	2,55E+06
sp|O62823|CASA1_BUBBU	IGSESTEDQAMEDIKQ	1779,78	44	59	1,27E+07
sp|O62823|CASA1_BUBBU	DIGSESTEDQAMEDIKQ	1894,81	43	59	2,32E+06
sp|O62823|CASA1_BUBBU	TDIGSESTEDQAMEDIKQ	1995,86	42	59	2,59E+06
sp|O62823|CASA1_BUBBU	STDIGSESTEDQAMEDIKQ	2082,89	41	59	5,81E+05
sp|O62823|CASA1_BUBBU	LSTDIGSESTEDQAMEDIKQ	2195,97	40	59	0,00E+00
sp|O62823|CASA1_BUBBU	ELSTDIGSESTEDQAMEDIKQ	2325,02	39	59	0,00E+00
sp|O62823|CASA1_BUBBU	NELSTDIGSESTEDQAMEDIKQ	2439,06	38	59	0,00E+00
sp|O62823|CASA1_BUBBU	EKVNELSTDIGSESTEDQAMEDIKQ	2795,27	35	59	0,00E+00
sp|O62823|CASA1_BUBBU	GKEKVNELSTDIGSESTEDQAMEDIKQ	2980,38	33	59	0,00E+00
sp|O62823|CASA1_BUBBU	EDIKQM	762,36	55	60	2,84E+08
sp|O62823|CASA1_BUBBU	MEDIKQM	893,40	54	60	3,84E+07
sp|O62823|CASA1_BUBBU	AMEDIKQM	964,44	53	60	2,55E+07
sp|O62823|CASA1_BUBBU	QAMEDIKQM	1092,49	52	60	1,89E+07
sp|O62823|CASA1_BUBBU	DQAMEDIKQM	1207,52	51	60	0,00E+00
sp|O62823|CASA1_BUBBU	IGSESTEDQAMEDIKQM	1910,82	44	60	0,00E+00
sp|O62823|CASA1_BUBBU	DIKQME	762,36	56	61	3,67E+07
sp|O62823|CASA1_BUBBU	DIKQMEAE	962,44	56	63	7,45E+07
sp|O62823|CASA1_BUBBU	AMEDIKQMEAE	1293,56	53	63	6,56E+06
sp|O62823|CASA1_BUBBU	SSEEIVPI	872,45	67	74	0,00E+00
sp|O62823|CASA1_BUBBU	SISSSEEIVPI	1159,60	64	74	0,00E+00
sp|O62823|CASA1_BUBBU	IVPISVE	755,44	71	77	2,12E+06
sp|O62823|CASA1_BUBBU	ISSSEEIVPISVE	1387,71	65	77	3,72E+06
sp|O62823|CASA1_BUBBU	SISSSEEIVPISVE	1474,74	64	77	0,00E+00
sp|O62823|CASA1_BUBBU	ISVEQ	574,30	74	78	5,57E+06
sp|O62823|CASA1_BUBBU	IVPISVEQK	1011,60	71	79	2,90E+06
sp|O62823|CASA1_BUBBU	EEIVPISVEQK	1269,68	69	79	4,24E+06
sp|O62823|CASA1_BUBBU	SISSSEEIVPISVEQK	1730,89	64	79	0,00E+00
sp|O62823|CASA1_BUBBU	VEQKHIQ	880,48	76	82	1,74E+06
sp|O62823|CASA1_BUBBU	QKHIQKED	1024,53	78	85	0,00E+00
sp|O62823|CASA1_BUBBU	QKEDVP	714,35	82	87	0,00E+00
sp|O62823|CASA1_BUBBU	HIQKEDVP	964,50	80	87	0,00E+00
sp|O62823|CASA1_BUBBU	KEDVPS	673,33	83	88	7,85E+06
sp|O62823|CASA1_BUBBU	QKEDVPS	801,39	82	88	1,05E+06

sp|O62823|CASA1_BUBBU	IQKEDVPS	914,47	81	88	5,69E+08
sp|O62823|CASA1_BUBBU	HIQKEDVPS	1051,53	80	88	3,24E+09
sp|O62823|CASA1_BUBBU	KHIQKEDVPS	1179,62	79	88	3,24E+06
sp|O62823|CASA1_BUBBU	QKHIQKEDVPS	1307,68	78	88	4,10E+06
sp|O62823|CASA1_BUBBU	KEDVPSE	802,37	83	89	5,15E+07
sp|O62823|CASA1_BUBBU	QKEDVPSE	930,43	82	89	3,50E+07
sp|O62823|CASA1_BUBBU	IQKEDVPSE	1043,51	81	89	2,82E+08
sp|O62823|CASA1_BUBBU	HIQKEDVPSE	1180,57	80	89	7,82E+08
sp|O62823|CASA1_BUBBU	QKHIQKEDVPSE	1436,73	78	89	0,00E+00
sp|O62823|CASA1_BUBBU	EDVPSER	830,38	84	90	2,81E+08
sp|O62823|CASA1_BUBBU	KEDVPSER	958,47	83	90	2,84E+07
sp|O62823|CASA1_BUBBU	QKEDVPSER	1086,53	82	90	2,89E+07
sp|O62823|CASA1_BUBBU	IQKEDVPSER	1199,61	81	90	2,13E+08
sp|O62823|CASA1_BUBBU	HIQKEDVPSER	1336,67	80	90	1,07E+09
sp|O62823|CASA1_BUBBU	QKHIQKEDVPSER	1592,83	78	90	0,00E+00
sp|O62823|CASA1_BUBBU	VPSERY	749,37	86	91	0,00E+00
sp|O62823|CASA1_BUBBU	DVPSERY	864,40	85	91	1,27E+09
sp|O62823|CASA1_BUBBU	EDVPSERY	993,44	84	91	8,50E+07
sp|O62823|CASA1_BUBBU	KEDVPSERY	1121,54	83	91	3,49E+08
sp|O62823|CASA1_BUBBU	QKEDVPSERY	1249,59	82	91	3,03E+08
sp|O62823|CASA1_BUBBU	IQKEDVPSERY	1362,68	81	91	8,70E+07
sp|O62823|CASA1_BUBBU	HIQKEDVPSERY	1499,74	80	91	1,91E+08
sp|O62823|CASA1_BUBBU	QKHIQKEDVPSERY	1755,89	78	91	1,25E+06
sp|O62823|CASA1_BUBBU	SERYL	666,33	88	92	1,89E+07
sp|O62823|CASA1_BUBBU	PSERYL	763,39	87	92	3,42E+07
sp|O62823|CASA1_BUBBU	VPSERYL	862,45	86	92	4,89E+07
sp|O62823|CASA1_BUBBU	DVPSERYL	977,48	85	92	9,73E+08
sp|O62823|CASA1_BUBBU	EDVPSERYL	1106,52	84	92	3,60E+08
sp|O62823|CASA1_BUBBU	KEDVPSERYL	1234,62	83	92	1,02E+09
sp|O62823|CASA1_BUBBU	QKEDVPSERYL	1362,68	82	92	6,90E+08
sp|O62823|CASA1_BUBBU	IQKEDVPSERYL	1475,76	81	92	2,35E+08
sp|O62823|CASA1_BUBBU	HIQKEDVPSERYL	1612,82	80	92	3,48E+08
sp|O62823|CASA1_BUBBU	QKHIQKEDVPSERYL	1868,97	78	92	0,00E+00
sp|O62823|CASA1_BUBBU	PSERYLG	820,41	87	93	4,28E+06
sp|O62823|CASA1_BUBBU	VPSERYLG	919,48	86	93	3,69E+07
sp|O62823|CASA1_BUBBU	DVPSERYLG	1034,50	85	93	2,21E+08
sp|O62823|CASA1_BUBBU	EDVPSERYLG	1163,55	84	93	2,55E+07
sp|O62823|CASA1_BUBBU	KEDVPSERYLG	1291,64	83	93	9,67E+07
sp|O62823|CASA1_BUBBU	QKEDVPSERYLG	1419,70	82	93	7,38E+07
sp|O62823|CASA1_BUBBU	IQKEDVPSERYLG	1532,78	81	93	6,32E+07
sp|O62823|CASA1_BUBBU	HIQKEDVPSERYLG	1669,84	80	93	4,45E+07
sp|O62823|CASA1_BUBBU	ERYLGY	799,39	89	94	3,20E+07
sp|O62823|CASA1_BUBBU	SERYLGY	886,42	88	94	1,01E+07
sp|O62823|CASA1_BUBBU	VPSERYLGY	1082,54	86	94	2,04E+07
sp|O62823|CASA1_BUBBU	DVPSERYLGY	1197,57	85	94	1,56E+08
sp|O62823|CASA1_BUBBU	EDVPSERYLGY	1326,61	84	94	4,34E+07
sp|O62823|CASA1_BUBBU	KEDVPSERYLGY	1454,70	83	94	1,67E+08
sp|O62823|CASA1_BUBBU	QKEDVPSERYLGY	1582,76	82	94	1,46E+08
sp|O62823|CASA1_BUBBU	IQKEDVPSERYLGY	1695,85	81	94	1,95E+07
sp|O62823|CASA1_BUBBU	HIQKEDVPSERYLGY	1832,91	80	94	2,07E+06
sp|O62823|CASA1_BUBBU	QKHIQKEDVPSERYLGY	2089,06	78	94	0,00E+00
sp|O62823|CASA1_BUBBU	RYLGYL	783,43	90	95	0,00E+00
sp|O62823|CASA1_BUBBU	ERYLGYL	912,47	89	95	2,75E+07
sp|O62823|CASA1_BUBBU	SERYLGYL	999,50	88	95	0,00E+00
sp|O62823|CASA1_BUBBU	PSERYLGYL	1096,56	87	95	7,44E+06
sp|O62823|CASA1_BUBBU	VPSERYLGYL	1195,62	86	95	5,62E+07
sp|O62823|CASA1_BUBBU	DVPSERYLGYL	1310,65	85	95	6,89E+08
sp|O62823|CASA1_BUBBU	EDVPSERYLGYL	1439,69	84	95	7,39E+08
sp|O62823|CASA1_BUBBU	KEDVPSERYLGYL	1567,79	83	95	3,60E+08
sp|O62823|CASA1_BUBBU	QKEDVPSERYLGYL	1695,85	82	95	3,25E+08
sp|O62823|CASA1_BUBBU	IQKEDVPSERYLGYL	1808,93	81	95	7,61E+06
sp|O62823|CASA1_BUBBU	HIQKEDVPSERYLGYL	1945,99	80	95	2,94E+08
sp|O62823|CASA1_BUBBU	QKHIQKEDVPSERYLGYL	2202,14	78	95	0,00E+00
sp|O62823|CASA1_BUBBU	RYLGYLE	912,47	90	96	2,26E+07
sp|O62823|CASA1_BUBBU	ERYLGYLE	1041,51	89	96	3,96E+07
sp|O62823|CASA1_BUBBU	SERYLGYLE	1128,55	88	96	4,76E+06
sp|O62823|CASA1_BUBBU	PSERYLGYLE	1225,60	87	96	3,08E+06
sp|O62823|CASA1_BUBBU	VPSERYLGYLE	1324,67	86	96	7,32E+07
sp|O62823|CASA1_BUBBU	DVPSERYLGYLE	1439,69	85	96	7,39E+08
sp|O62823|CASA1_BUBBU	EDVPSERYLGYLE	1568,74	84	96	9,89E+07
sp|O62823|CASA1_BUBBU	KEDVPSERYLGYLE	1696,83	83	96	4,93E+08
sp|O62823|CASA1_BUBBU	QKEDVPSERYLGYLE	1824,89	82	96	4,11E+08
sp|O62823|CASA1_BUBBU	IQKEDVPSERYLGYLE	1937,97	81	96	7,25E+07
sp|O62823|CASA1_BUBBU	HIQKEDVPSERYLGYLE	2075,03	80	96	2,22E+08
sp|O62823|CASA1_BUBBU	QKHIQKEDVPSERYLGYLE	2331,19	78	96	0,00E+00
sp|O62823|CASA1_BUBBU	LGYLEQ	721,36	92	97	5,69E+07
sp|O62823|CASA1_BUBBU	YLGYLEQ	884,43	91	97	2,61E+07
sp|O62823|CASA1_BUBBU	RYLGYLEQ	1040,53	90	97	3,22E+07
sp|O62823|CASA1_BUBBU	ERYLGYLEQ	1169,57	89	97	7,49E+07
sp|O62823|CASA1_BUBBU	SERYLGYLEQ	1256,60	88	97	8,67E+06
sp|O62823|CASA1_BUBBU	VPSERYLGYLEQ	1452,72	86	97	5,30E+07
sp|O62823|CASA1_BUBBU	DVPSERYLGYLEQ	1567,75	85	97	3,46E+08
sp|O62823|CASA1_BUBBU	EDVPSERYLGYLEQ	1696,79	84	97	4,22E+07
sp|O62823|CASA1_BUBBU	KEDVPSERYLGYLEQ	1824,89	83	97	1,72E+07
sp|O62823|CASA1_BUBBU	QKEDVPSERYLGYLEQ	1952,95	82	97	1,59E+08
sp|O62823|CASA1_BUBBU	IQKEDVPSERYLGYLEQ	2066,03	81	97	5,68E+07
sp|O62823|CASA1_BUBBU	HIQKEDVPSERYLGYLEQ	2203,09	80	97	3,12E+08
sp|O62823|CASA1_BUBBU	YLEQL	664,34	94	98	1,26E+08
sp|O62823|CASA1_BUBBU	YLGYLEQL	997,51	91	98	1,66E+08
sp|O62823|CASA1_BUBBU	RYLGYLEQL	1153,61	90	98	1,06E+08
sp|O62823|CASA1_BUBBU	ERYLGYLEQL	1282,66	89	98	2,26E+08
sp|O62823|CASA1_BUBBU	SERYLGYLEQL	1369,69	88	98	1,38E+07
sp|O62823|CASA1_BUBBU	PSERYLGYLEQL	1466,74	87	98	2,10E+06
sp|O62823|CASA1_BUBBU	VPSERYLGYLEQL	1565,81	86	98	4,32E+08
sp|O62823|CASA1_BUBBU	DVPSERYLGYLEQL	1680,84	85	98	1,51E+09
sp|O62823|CASA1_BUBBU	EDVPSERYLGYLEQL	1809,88	84	98	3,30E+08
sp|O62823|CASA1_BUBBU	KEDVPSERYLGYLEQL	1937,97	83	98	2,53E+09
sp|O62823|CASA1_BUBBU	QKEDVPSERYLGYLEQL	2066,03	82	98	1,26E+09

sp|O62823|CASA1_BUBBU	IQKEDVPSERYLGYLEQL	2179,12	81	98	6,40E+08
sp|O62823|CASA1_BUBBU	HIQKEDVPSERYLGYLEQL	2316,18	80	98	1,19E+09
sp|O62823|CASA1_BUBBU	QKHIQKEDVPSERYLGYLEQL	2572,33	78	98	2,60E+07
sp|O62823|CASA1_BUBBU	GYLEQLL	834,45	93	99	5,34E+07
sp|O62823|CASA1_BUBBU	LGYLEQLL	947,53	92	99	6,82E+06
sp|O62823|CASA1_BUBBU	YLGYLEQLL	1110,60	91	99	8,76E+06
sp|O62823|CASA1_BUBBU	VPSERYLGYLEQLL	1678,89	86	99	2,97E+07
sp|O62823|CASA1_BUBBU	DVPSERYLGYLEQLL	1793,92	85	99	9,35E+07
sp|O62823|CASA1_BUBBU	KEDVPSERYLGYLEQLL	2051,06	83	99	6,68E+07
sp|O62823|CASA1_BUBBU	QKEDVPSERYLGYLEQLL	2179,12	82	99	3,82E+07
sp|O62823|CASA1_BUBBU	YLEQLLR	933,53	94	100	2,29E+07
sp|O62823|CASA1_BUBBU	GYLEQLLR	990,55	93	100	1,36E+08
sp|O62823|CASA1_BUBBU	LGYLEQLLR	1103,63	92	100	7,58E+07
sp|O62823|CASA1_BUBBU	YLGYLEQLLR	1266,70	91	100	4,04E+07
sp|O62823|CASA1_BUBBU	RYLGYLEQLLR	1422,80	90	100	3,02E+06
sp|O62823|CASA1_BUBBU	VPSERYLGYLEQLLR	1834,99	86	100	2,98E+08
sp|O62823|CASA1_BUBBU	DVPSERYLGYLEQLLR	1950,02	85	100	1,57E+08
sp|O62823|CASA1_BUBBU	EDVPSERYLGYLEQLLR	2079,06	84	100	1,51E+07
sp|O62823|CASA1_BUBBU	KEDVPSERYLGYLEQLLR	2207,16	83	100	2,41E+08
sp|O62823|CASA1_BUBBU	QKEDVPSERYLGYLEQLLR	2335,22	82	100	2,08E+07
sp|O62823|CASA1_BUBBU	IQKEDVPSERYLGYLEQLLR	2448,30	81	100	1,92E+08
sp|O62823|CASA1_BUBBU	HIQKEDVPSERYLGYLEQLLR	2585,36	80	100	1,82E+08
sp|O62823|CASA1_BUBBU	LEQLLRL	883,55	95	101	1,83E+07
sp|O62823|CASA1_BUBBU	YLEQLLRL	1046,61	94	101	2,24E+07
sp|O62823|CASA1_BUBBU	GYLEQLLRL	1103,63	93	101	5,52E+07
sp|O62823|CASA1_BUBBU	QLLRLK	769,52	97	102	6,28E+07
sp|O62823|CASA1_BUBBU	EQLLRLK	898,56	96	102	1,02E+07
sp|O62823|CASA1_BUBBU	LEQLLRLK	1011,64	95	102	1,20E+06
sp|O62823|CASA1_BUBBU	YLEQLLRLK	1174,71	94	102	3,97E+06
sp|O62823|CASA1_BUBBU	GYLEQLLRLK	1231,73	93	102	4,59E+07
sp|O62823|CASA1_BUBBU	LGYLEQLLRLK	1344,81	92	102	2,84E+07
sp|O62823|CASA1_BUBBU	DVPSERYLGYLEQLLRLK	2191,20	85	102	8,90E+06
sp|O62823|CASA1_BUBBU	GYLEQLLRLKK	1359,82	93	103	1,91E+06
sp|O62823|CASA1_BUBBU	LGYLEQLLRLKK	1472,91	92	103	7,24E+05
sp|O62823|CASA1_BUBBU	LRLKKY	819,53	99	104	1,07E+07
sp|O62823|CASA1_BUBBU	LRLKKYN	933,58	99	105	3,99E+06
sp|O62823|CASA1_BUBBU	KYNVP	619,33	103	107	2,26E+08
sp|O62823|CASA1_BUBBU	YNVPQ	619,30	104	108	4,27E+07
sp|O62823|CASA1_BUBBU	KKYNVPQ	875,49	102	108	1,02E+08
sp|O62823|CASA1_BUBBU	LKKYNVPQ	988,57	101	108	5,53E+07
sp|O62823|CASA1_BUBBU	RLKKYNVPQ	1144,67	100	108	5,31E+07
sp|O62823|CASA1_BUBBU	YNVPQL	732,38	104	109	3,23E+06
sp|O62823|CASA1_BUBBU	KYNVPQL	860,48	103	109	4,08E+07
sp|O62823|CASA1_BUBBU	VPQLE	584,32	106	110	1,90E+07
sp|O62823|CASA1_BUBBU	NVPQLE	698,36	105	110	2,89E+07
sp|O62823|CASA1_BUBBU	KYNVPQLE	989,52	103	110	5,16E+06
sp|O62823|CASA1_BUBBU	VPQLEI	697,40	106	111	8,40E+06
sp|O62823|CASA1_BUBBU	VPQLEIV	796,47	106	112	1,12E+07
sp|O62823|CASA1_BUBBU	NVPQLEIV	910,51	105	112	1,33E+07
sp|O62823|CASA1_BUBBU	QLEIVP	697,40	108	113	2,32E+08
sp|O62823|CASA1_BUBBU	PQLEIVP	794,45	107	113	8,78E+05
sp|O62823|CASA1_BUBBU	VPQLEIVP	893,52	106	113	2,16E+08
sp|O62823|CASA1_BUBBU	NVPQLEIVP	1007,57	105	113	1,81E+08
sp|O62823|CASA1_BUBBU	YNVPQLEIVP	1170,63	104	113	1,02E+08
sp|O62823|CASA1_BUBBU	KYNVPQLEIVP	1298,72	103	113	3,75E+07
sp|O62823|CASA1_BUBBU	KKYNVPQLEIVP	1426,82	102	113	1,28E+07
sp|O62823|CASA1_BUBBU	LKKYNVPQLEIVP	1539,90	101	113	1,54E+07
sp|O62823|CASA1_BUBBU	QLEIVPN	811,44	108	114	3,42E+07
sp|O62823|CASA1_BUBBU	VPQLEIVPN	1007,57	106	114	3,00E+08
sp|O62823|CASA1_BUBBU	NVPQLEIVPN	1121,61	105	114	2,19E+08
sp|O62823|CASA1_BUBBU	YNVPQLEIVPN	1284,67	104	114	4,78E+07
sp|O62823|CASA1_BUBBU	KYNVPQLEIVPN	1412,77	103	114	7,77E+07
sp|O62823|CASA1_BUBBU	KKYNVPQLEIVPN	1540,86	102	114	1,66E+06
sp|O62823|CASA1_BUBBU	LKKYNVPQLEIVPN	1653,95	101	114	2,10E+07
sp|O62823|CASA1_BUBBU	LEIVPNL	796,47	109	115	7,20E+06
sp|O62823|CASA1_BUBBU	QLEIVPNL	924,53	108	115	0,00E+00
sp|O62823|CASA1_BUBBU	VPQLEIVPNL	1120,65	106	115	1,55E+08
sp|O62823|CASA1_BUBBU	NVPQLEIVPNL	1234,69	105	115	7,53E+07
sp|O62823|CASA1_BUBBU	YNVPQLEIVPNL	1397,76	104	115	1,79E+07
sp|O62823|CASA1_BUBBU	EIVPNLA	754,42	110	116	2,59E+07
sp|O62823|CASA1_BUBBU	QLEIVPNLA	995,57	108	116	6,07E+06
sp|O62823|CASA1_BUBBU	VPQLEIVPNLA	1191,69	106	116	7,33E+07
sp|O62823|CASA1_BUBBU	NVPQLEIVPNLA	1305,73	105	116	4,23E+07
sp|O62823|CASA1_BUBBU	KYNVPQLEIVPNLA	1596,89	103	116	1,11E+07
sp|O62823|CASA1_BUBBU	PNLAE	542,27	113	117	6,09E+06
sp|O62823|CASA1_BUBBU	IVPNLAE	754,42	111	117	1,62E+08
sp|O62823|CASA1_BUBBU	EIVPNLAE	883,47	110	117	2,73E+07
sp|O62823|CASA1_BUBBU	LEIVPNLAE	996,55	109	117	5,36E+07
sp|O62823|CASA1_BUBBU	QLEIVPNLAE	1124,61	108	117	7,94E+07
sp|O62823|CASA1_BUBBU	VPQLEIVPNLAE	1320,73	106	117	3,04E+08
sp|O62823|CASA1_BUBBU	NVPQLEIVPNLAE	1434,77	105	117	1,73E+08
sp|O62823|CASA1_BUBBU	YNVPQLEIVPNLAE	1597,84	104	117	5,75E+07
sp|O62823|CASA1_BUBBU	KYNVPQLEIVPNLAE	1725,93	103	117	5,51E+07
sp|O62823|CASA1_BUBBU	LKKYNVPQLEIVPNLAE	1967,11	101	117	2,08E+07
sp|O62823|CASA1_BUBBU	VPNLAEE	770,38	112	118	4,01E+06
sp|O62823|CASA1_BUBBU	IVPNLAEE	883,47	111	118	2,60E+07
sp|O62823|CASA1_BUBBU	EIVPNLAEE	1012,51	110	118	1,43E+07
sp|O62823|CASA1_BUBBU	VPQLEIVPNLAEE	1449,77	106	118	6,27E+07
sp|O62823|CASA1_BUBBU	NVPQLEIVPNLAEE	1563,81	105	118	8,69E+07
sp|O62823|CASA1_BUBBU	KYNVPQLEIVPNLAEE	1854,97	103	118	1,91E+07
sp|O62823|CASA1_BUBBU	KKYNVPQLEIVPNLAEE	1983,07	102	118	5,82E+06
sp|O62823|CASA1_BUBBU	LKKYNVPQLEIVPNLAEE	2096,15	101	118	7,69E+06
sp|O62823|CASA1_BUBBU	VPNLAEEQ	898,44	112	119	2,85E+07
sp|O62823|CASA1_BUBBU	IVPNLAEEQ	1011,52	111	119	8,52E+07
sp|O62823|CASA1_BUBBU	EIVPNLAEEQ	1140,57	110	119	2,70E+07
sp|O62823|CASA1_BUBBU	LEIVPNLAEEQ	1253,65	109	119	4,73E+06
sp|O62823|CASA1_BUBBU	QLEIVPNLAEEQ	1381,71	108	119	2,74E+07
sp|O62823|CASA1_BUBBU	VPQLEIVPNLAEEQ	1577,83	106	119	4,94E+07

sp|O62823|CASA1_BUBBU	NVPQLEIVPNLAEEQ	1691,87	105	119	1,62E+07
sp|O62823|CASA1_BUBBU	KYNVPQLEIVPNLAEEQ	1983,03	103	119	8,74E+06
sp|O62823|CASA1_BUBBU	QLEIVPNLAEEQL	1494,79	108	120	0,00E+00
sp|O62823|CASA1_BUBBU	VPQLEIVPNLAEEQL	1690,91	106	120	8,81E+07
sp|O62823|CASA1_BUBBU	NVPQLEIVPNLAEEQL	1804,96	105	120	6,09E+07
sp|O62823|CASA1_BUBBU	YNVPQLEIVPNLAEEQL	1968,02	104	120	2,41E+07
sp|O62823|CASA1_BUBBU	EEQLH	654,30	117	121	7,92E+06
sp|O62823|CASA1_BUBBU	AEEQLH	725,33	116	121	3,01E+06
sp|O62823|CASA1_BUBBU	LAEEQLH	838,42	115	121	5,99E+08
sp|O62823|CASA1_BUBBU	NLAEEQLH	952,46	114	121	2,21E+08
sp|O62823|CASA1_BUBBU	PNLAEEQLH	1049,51	113	121	1,02E+07
sp|O62823|CASA1_BUBBU	VPNLAEEQLH	1148,58	112	121	4,11E+08
sp|O62823|CASA1_BUBBU	IVPNLAEEQLH	1261,67	111	121	8,47E+08
sp|O62823|CASA1_BUBBU	EIVPNLAEEQLH	1390,71	110	121	2,13E+09
sp|O62823|CASA1_BUBBU	LEIVPNLAEEQLH	1503,79	109	121	4,61E+08
sp|O62823|CASA1_BUBBU	QLEIVPNLAEEQLH	1631,85	108	121	2,49E+09
sp|O62823|CASA1_BUBBU	PQLEIVPNLAEEQLH	1728,90	107	121	6,27E+06
sp|O62823|CASA1_BUBBU	VPQLEIVPNLAEEQLH	1827,97	106	121	5,16E+09
sp|O62823|CASA1_BUBBU	NVPQLEIVPNLAEEQLH	1942,02	105	121	9,93E+08
sp|O62823|CASA1_BUBBU	YNVPQLEIVPNLAEEQLH	2105,08	104	121	9,57E+08
sp|O62823|CASA1_BUBBU	KYNVPQLEIVPNLAEEQLH	2233,17	103	121	1,31E+09
sp|O62823|CASA1_BUBBU	KKYNVPQLEIVPNLAEEQLH	2361,27	102	121	7,40E+07
sp|O62823|CASA1_BUBBU	LKKYNVPQLEIVPNLAEEQLH	2474,35	101	121	1,95E+08
sp|O62823|CASA1_BUBBU	RLKKYNVPQLEIVPNLAEEQLH	2630,45	100	121	3,76E+07
sp|O62823|CASA1_BUBBU	VPNLAEEQLHS	1235,61	112	122	1,88E+06
sp|O62823|CASA1_BUBBU	IVPNLAEEQLHS	1348,70	111	122	3,68E+06
sp|O62823|CASA1_BUBBU	EIVPNLAEEQLHS	1477,74	110	122	1,23E+07
sp|O62823|CASA1_BUBBU	QLEIVPNLAEEQLHS	1718,88	108	122	9,11E+06
sp|O62823|CASA1_BUBBU	VPQLEIVPNLAEEQLHS	1915,01	106	122	1,97E+07
sp|O62823|CASA1_BUBBU	KYNVPQLEIVPNLAEEQLHS	2320,21	103	122	1,69E+07
sp|O62823|CASA1_BUBBU	EEQLHSM	872,37	117	123	1,37E+08
sp|O62823|CASA1_BUBBU	AEEQLHSM	943,41	116	123	3,84E+08
sp|O62823|CASA1_BUBBU	LAEEQLHSM	1056,49	115	123	1,60E+08
sp|O62823|CASA1_BUBBU	NLAEEQLHSM	1170,53	114	123	5,48E+07
sp|O62823|CASA1_BUBBU	VPNLAEEQLHSM	1366,66	112	123	0,00E+00
sp|O62823|CASA1_BUBBU	IVPNLAEEQLHSM	1479,74	111	123	7,95E+07
sp|O62823|CASA1_BUBBU	EIVPNLAEEQLHSM	1608,78	110	123	1,95E+08
sp|O62823|CASA1_BUBBU	LEIVPNLAEEQLHSM	1721,87	109	123	6,18E+07
sp|O62823|CASA1_BUBBU	QLEIVPNLAEEQLHSM	1849,92	108	123	2,05E+08
sp|O62823|CASA1_BUBBU	VPQLEIVPNLAEEQLHSM	2046,05	106	123	5,90E+08
sp|O62823|CASA1_BUBBU	NVPQLEIVPNLAEEQLHSM	2160,09	105	123	2,37E+07
sp|O62823|CASA1_BUBBU	YNVPQLEIVPNLAEEQLHSM	2323,15	104	123	2,62E+07
sp|O62823|CASA1_BUBBU	KYNVPQLEIVPNLAEEQLHSM	2451,25	103	123	2,78E+08
sp|O62823|CASA1_BUBBU	KKYNVPQLEIVPNLAEEQLHSM	2579,34	102	123	1,86E+07
sp|O62823|CASA1_BUBBU	LKKYNVPQLEIVPNLAEEQLHSM	2692,43	101	123	4,09E+07
sp|O62823|CASA1_BUBBU	QLEIVPNLAEEQLHSMK	1978,02	108	124	1,64E+07
sp|O62823|CASA1_BUBBU	VPQLEIVPNLAEEQLHSMK	2174,14	106	124	2,14E+07
sp|O62823|CASA1_BUBBU	KYNVPQLEIVPNLAEEQLHSMK	2579,34	103	124	5,36E+06
sp|O62823|CASA1_BUBBU	QLEIVPNLAEEQLHSMKE	2107,06	108	125	3,79E+06
sp|O62823|CASA1_BUBBU	MKEGIH	713,35	123	128	1,12E+07
sp|O62823|CASA1_BUBBU	SMKEGIH	800,39	122	128	8,80E+07
sp|O62823|CASA1_BUBBU	HSMKEGIH	937,44	121	128	1,14E+07
sp|O62823|CASA1_BUBBU	LHSMKEGIH	1050,53	120	128	2,57E+06
sp|O62823|CASA1_BUBBU	EQLHSMKEGIH	1307,63	118	128	2,08E+06
sp|O62823|CASA1_BUBBU	EIVPNLAEEQLHSMKEGIH	2173,08	110	128	6,39E+06
sp|O62823|CASA1_BUBBU	QLEIVPNLAEEQLHSMKEGIH	2414,23	108	128	2,15E+07
sp|O62823|CASA1_BUBBU	VPQLEIVPNLAEEQLHSMKEGIH	2610,35	106	128	4,77E+07
sp|O62823|CASA1_BUBBU	NVPQLEIVPNLAEEQLHSMKEGIH	2724,39	105	128	1,35E+07
sp|O62823|CASA1_BUBBU	KYNVPQLEIVPNLAEEQLHSMKEGIH	3015,55	103	128	1,10E+07
sp|O62823|CASA1_BUBBU	MKEGIHA	784,39	123	129	3,54E+08
sp|O62823|CASA1_BUBBU	SMKEGIHA	871,42	122	129	5,24E+08
sp|O62823|CASA1_BUBBU	HSMKEGIHA	1008,48	121	129	1,66E+07
sp|O62823|CASA1_BUBBU	LHSMKEGIHA	1121,57	120	129	5,71E+06
sp|O62823|CASA1_BUBBU	QLHSMKEGIHA	1249,62	119	129	0,00E+00
sp|O62823|CASA1_BUBBU	EQLHSMKEGIHA	1378,67	118	129	3,36E+06
sp|O62823|CASA1_BUBBU	EEQLHSMKEGIHA	1507,71	117	129	1,07E+07
sp|O62823|CASA1_BUBBU	AEEQLHSMKEGIHA	1578,75	116	129	8,46E+06
sp|O62823|CASA1_BUBBU	LAEEQLHSMKEGIHA	1691,83	115	129	2,26E+07
sp|O62823|CASA1_BUBBU	NLAEEQLHSMKEGIHA	1805,87	114	129	4,04E+07
sp|O62823|CASA1_BUBBU	PNLAEEQLHSMKEGIHA	1902,93	113	129	1,06E+06
sp|O62823|CASA1_BUBBU	EIVPNLAEEQLHSMKEGIHA	2244,12	110	129	1,53E+08
sp|O62823|CASA1_BUBBU	LEIVPNLAEEQLHSMKEGIHA	2357,20	109	129	5,52E+07
sp|O62823|CASA1_BUBBU	QLEIVPNLAEEQLHSMKEGIHA	2485,26	108	129	2,59E+08
sp|O62823|CASA1_BUBBU	VPQLEIVPNLAEEQLHSMKEGIHA	2681,38	106	129	8,20E+08
sp|O62823|CASA1_BUBBU	NVPQLEIVPNLAEEQLHSMKEGIHA	2795,43	105	129	9,13E+07
sp|O62823|CASA1_BUBBU	YNVPQLEIVPNLAEEQLHSMKEGIHA	2958,49	104	129	4,61E+07
sp|O62823|CASA1_BUBBU	KYNVPQLEIVPNLAEEQLHSMKEGIHA	3086,59	103	129	1,54E+08
sp|O62823|CASA1_BUBBU	EGIHAQ	653,31	125	130	5,11E+06
sp|O62823|CASA1_BUBBU	KEGIHAQ	781,41	124	130	4,00E+07
sp|O62823|CASA1_BUBBU	MKEGIHAQ	912,45	123	130	9,52E+07
sp|O62823|CASA1_BUBBU	SMKEGIHAQ	999,48	122	130	5,59E+08
sp|O62823|CASA1_BUBBU	HSMKEGIHAQ	1136,54	121	130	5,83E+07
sp|O62823|CASA1_BUBBU	LHSMKEGIHAQ	1249,62	120	130	2,57E+07
sp|O62823|CASA1_BUBBU	QLHSMKEGIHAQ	1377,68	119	130	4,80E+06
sp|O62823|CASA1_BUBBU	EQLHSMKEGIHAQ	1506,72	118	130	4,15E+07
sp|O62823|CASA1_BUBBU	AEEQLHSMKEGIHAQ	1706,80	116	130	1,08E+07
sp|O62823|CASA1_BUBBU	LAEEQLHSMKEGIHAQ	1819,89	115	130	1,77E+07
sp|O62823|CASA1_BUBBU	NLAEEQLHSMKEGIHAQ	1933,93	114	130	3,18E+08
sp|O62823|CASA1_BUBBU	EIVPNLAEEQLHSMKEGIHAQ	2372,18	110	130	1,19E+08
sp|O62823|CASA1_BUBBU	LEIVPNLAEEQLHSMKEGIHAQ	2485,26	109	130	9,28E+07
sp|O62823|CASA1_BUBBU	QLEIVPNLAEEQLHSMKEGIHAQ	2613,32	108	130	2,34E+08
sp|O62823|CASA1_BUBBU	VPQLEIVPNLAEEQLHSMKEGIHAQ	2809,44	106	130	4,37E+08
sp|O62823|CASA1_BUBBU	NVPQLEIVPNLAEEQLHSMKEGIHAQ	2923,49	105	130	9,12E+07
sp|O62823|CASA1_BUBBU	KYNVPQLEIVPNLAEEQLHSMKEGIHAQ	3214,64	103	130	5,60E+07
sp|O62823|CASA1_BUBBU	MKEGIHAQQ	1040,51	123	131	2,24E+07
sp|O62823|CASA1_BUBBU	SMKEGIHAQQ	1127,54	122	131	1,27E+08
sp|O62823|CASA1_BUBBU	HSMKEGIHAQQ	1264,60	121	131	7,13E+06
sp|O62823|CASA1_BUBBU	NLAEEQLHSMKEGIHAQQ	2061,99	114	131	4,61E+06

sp|O62823|CASA1_BUBBU	GIHAQQK	780,42	126	132	3,77E+06
sp|O62823|CASA1_BUBBU	EGIHAQQK	909,47	125	132	1,98E+07
sp|O62823|CASA1_BUBBU	KEGIHAQQK	1037,56	124	132	2,77E+06
sp|O62823|CASA1_BUBBU	MKEGIHAQQK	1168,60	123	132	1,13E+08
sp|O62823|CASA1_BUBBU	SMKEGIHAQQK	1255,63	122	132	2,37E+08
sp|O62823|CASA1_BUBBU	HSMKEGIHAQQK	1392,69	121	132	1,58E+07
sp|O62823|CASA1_BUBBU	EQLHSMKEGIHAQQK	1762,88	118	132	8,23E+05
sp|O62823|CASA1_BUBBU	NLAEEQLHSMKEGIHAQQK	2190,09	114	132	8,18E+06
sp|O62823|CASA1_BUBBU	IVPNLAEEQLHSMKEGIHAQQK	2499,29	111	132	3,30E+06
sp|O62823|CASA1_BUBBU	EIVPNLAEEQLHSMKEGIHAQQK	2628,33	110	132	2,26E+07
sp|O62823|CASA1_BUBBU	LEIVPNLAEEQLHSMKEGIHAQQK	2741,42	109	132	1,04E+07
sp|O62823|CASA1_BUBBU	QLEIVPNLAEEQLHSMKEGIHAQQK	2869,48	108	132	2,14E+07
sp|O62823|CASA1_BUBBU	VPQLEIVPNLAEEQLHSMKEGIHAQQK	3065,60	106	132	1,70E+07
sp|O62823|CASA1_BUBBU	KEGIHAQQKEP	1263,66	124	134	7,91E+06
sp|O62823|CASA1_BUBBU	SMKEGIHAQQKEP	1481,73	122	134	3,22E+07
sp|O62823|CASA1_BUBBU	QQKEPM	759,36	130	135	0,00E+00
sp|O62823|CASA1_BUBBU	AQQKEPM	830,40	129	135	3,26E+06
sp|O62823|CASA1_BUBBU	IHAQQKEPM	1080,54	127	135	1,04E+07
sp|O62823|CASA1_BUBBU	GIHAQQKEPM	1137,56	126	135	1,19E+07
sp|O62823|CASA1_BUBBU	EGIHAQQKEPM	1266,60	125	135	2,01E+06
sp|O62823|CASA1_BUBBU	KEGIHAQQKEPM	1394,70	124	135	1,12E+07
sp|O62823|CASA1_BUBBU	SMKEGIHAQQKEPM	1612,77	122	135	2,38E+06
sp|O62823|CASA1_BUBBU	EPMIG	545,25	133	137	1,99E+06
sp|O62823|CASA1_BUBBU	KEPMIG	673,35	132	137	2,62E+06
sp|O62823|CASA1_BUBBU	QKEPMIG	801,41	131	137	0,00E+00
sp|O62823|CASA1_BUBBU	EPMIGV	644,32	133	138	1,33E+06
sp|O62823|CASA1_BUBBU	QKEPMIGV	900,47	131	138	5,41E+06
sp|O62823|CASA1_BUBBU	QQKEPMIGV	1028,53	130	138	1,97E+06
sp|O62823|CASA1_BUBBU	EPMIGVN	758,36	133	139	2,92E+07
sp|O62823|CASA1_BUBBU	KEPMIGVN	886,46	132	139	2,99E+07
sp|O62823|CASA1_BUBBU	QKEPMIGVN	1014,52	131	139	1,77E+08
sp|O62823|CASA1_BUBBU	QQKEPMIGVN	1142,58	130	139	1,21E+08
sp|O62823|CASA1_BUBBU	AQQKEPMIGVN	1213,61	129	139	3,29E+07
sp|O62823|CASA1_BUBBU	IHAQQKEPMIGVN	1463,76	127	139	1,46E+07
sp|O62823|CASA1_BUBBU	GIHAQQKEPMIGVN	1520,78	126	139	6,27E+06
sp|O62823|CASA1_BUBBU	EGIHAQQKEPMIGVN	1649,82	125	139	1,65E+07
sp|O62823|CASA1_BUBBU	KEGIHAQQKEPMIGVN	1777,91	124	139	1,08E+07
sp|O62823|CASA1_BUBBU	MKEGIHAQQKEPMIGVN	1908,96	123	139	3,98E+06
sp|O62823|CASA1_BUBBU	SMKEGIHAQQKEPMIGVN	1995,99	122	139	7,48E+07
sp|O62823|CASA1_BUBBU	HSMKEGIHAQQKEPMIGVN	2133,05	121	139	5,21E+06
sp|O62823|CASA1_BUBBU	NLAEEQLHSMKEGIHAQQKEPMIGVN	2930,44	114	139	4,63E+06
sp|O62823|CASA1_BUBBU	VPQLEIVPNLAEEQLHSMKEGIHAQQKEPMIGVN	3805,95	106	139	1,81E+08
sp|O62823|CASA1_BUBBU	KYNVPQLEIVPNLAEEQLHSMKEGIHAQQKEPMIGVN	4211,15	103	139	2,58E+07
sp|O62823|CASA1_BUBBU	MIGVNQ	660,33	135	140	1,66E+07
sp|O62823|CASA1_BUBBU	EPMIGVNQ	886,42	133	140	3,34E+07
sp|O62823|CASA1_BUBBU	KEPMIGVNQ	1014,52	132	140	6,11E+07
sp|O62823|CASA1_BUBBU	QKEPMIGVNQ	1142,58	131	140	1,21E+08
sp|O62823|CASA1_BUBBU	QQKEPMIGVNQ	1270,63	130	140	2,32E+07
sp|O62823|CASA1_BUBBU	AQQKEPMIGVNQ	1341,67	129	140	5,75E+06
sp|O62823|CASA1_BUBBU	IHAQQKEPMIGVNQ	1591,81	127	140	9,33E+06
sp|O62823|CASA1_BUBBU	GIHAQQKEPMIGVNQ	1648,84	126	140	1,28E+06
sp|O62823|CASA1_BUBBU	EGIHAQQKEPMIGVNQ	1777,88	125	140	2,18E+06
sp|O62823|CASA1_BUBBU	KEGIHAQQKEPMIGVNQ	1905,97	124	140	5,29E+06
sp|O62823|CASA1_BUBBU	SMKEGIHAQQKEPMIGVNQ	2124,05	122	140	3,12E+07
sp|O62823|CASA1_BUBBU	VPQLEIVPNLAEEQLHSMKEGIHAQQKEPMIGVNQ	3934,01	106	140	9,19E+06
sp|O62823|CASA1_BUBBU	EPMIGVNQE	1015,46	133	141	0,00E+00
sp|O62823|CASA1_BUBBU	KEPMIGVNQE	1143,56	132	141	4,21E+06
sp|O62823|CASA1_BUBBU	QKEPMIGVNQE	1271,62	131	141	2,26E+07
sp|O62823|CASA1_BUBBU	QQKEPMIGVNQE	1399,68	130	141	0,00E+00
sp|O62823|CASA1_BUBBU	AQQKEPMIGVNQE	1470,71	129	141	3,16E+06
sp|O62823|CASA1_BUBBU	SMKEGIHAQQKEPMIGVNQE	2253,09	122	141	9,42E+06
sp|O62823|CASA1_BUBBU	GVNQEL	658,33	137	142	3,38E+07
sp|O62823|CASA1_BUBBU	EPMIGVNQEL	1128,55	133	142	3,08E+08
sp|O62823|CASA1_BUBBU	KEPMIGVNQEL	1256,64	132	142	9,94E+07
sp|O62823|CASA1_BUBBU	QKEPMIGVNQEL	1384,70	131	142	8,99E+08
sp|O62823|CASA1_BUBBU	QQKEPMIGVNQEL	1512,76	130	142	1,28E+08
sp|O62823|CASA1_BUBBU	AQQKEPMIGVNQEL	1583,80	129	142	2,94E+07
sp|O62823|CASA1_BUBBU	HAQQKEPMIGVNQEL	1720,86	128	142	6,72E+06
sp|O62823|CASA1_BUBBU	IHAQQKEPMIGVNQEL	1833,94	127	142	3,68E+06
sp|O62823|CASA1_BUBBU	GIHAQQKEPMIGVNQEL	1890,96	126	142	2,10E+06
sp|O62823|CASA1_BUBBU	EGIHAQQKEPMIGVNQEL	2020,00	125	142	1,60E+08
sp|O62823|CASA1_BUBBU	KEGIHAQQKEPMIGVNQEL	2148,10	124	142	1,26E+08
sp|O62823|CASA1_BUBBU	MKEGIHAQQKEPMIGVNQEL	2279,14	123	142	3,19E+07
sp|O62823|CASA1_BUBBU	SMKEGIHAQQKEPMIGVNQEL	2366,17	122	142	4,45E+08
sp|O62823|CASA1_BUBBU	HSMKEGIHAQQKEPMIGVNQEL	2503,23	121	142	2,38E+07
sp|O62823|CASA1_BUBBU	NLAEEQLHSMKEGIHAQQKEPMIGVNQEL	3300,62	114	142	7,26E+06
sp|O62823|CASA1_BUBBU	QLEIVPNLAEEQLHSMKEGIHAQQKEPMIGVNQEL	3980,01	108	142	4,20E+07
sp|O62823|CASA1_BUBBU	IGVNQELA	842,45	136	143	2,19E+07
sp|O62823|CASA1_BUBBU	MIGVNQELA	973,49	135	143	2,88E+06
sp|O62823|CASA1_BUBBU	EPMIGVNQELA	1199,59	133	143	3,61E+07
sp|O62823|CASA1_BUBBU	KEPMIGVNQELA	1327,68	132	143	0,00E+00
sp|O62823|CASA1_BUBBU	QKEPMIGVNQELA	1455,74	131	143	1,53E+08
sp|O62823|CASA1_BUBBU	QQKEPMIGVNQELA	1583,80	130	143	1,22E+07
sp|O62823|CASA1_BUBBU	AQQKEPMIGVNQELA	1654,83	129	143	4,69E+06
sp|O62823|CASA1_BUBBU	IHAQQKEPMIGVNQELA	1904,98	127	143	0,00E+00
sp|O62823|CASA1_BUBBU	GIHAQQKEPMIGVNQELA	1962,00	126	143	1,34E+07
sp|O62823|CASA1_BUBBU	EGIHAQQKEPMIGVNQELA	2091,04	125	143	2,08E+07
sp|O62823|CASA1_BUBBU	KEGIHAQQKEPMIGVNQELA	2219,14	124	143	1,34E+07
sp|O62823|CASA1_BUBBU	MKEGIHAQQKEPMIGVNQELA	2350,18	123	143	4,83E+06
sp|O62823|CASA1_BUBBU	SMKEGIHAQQKEPMIGVNQELA	2437,21	122	143	5,44E+07
sp|O62823|CASA1_BUBBU	IGVNQELAY	1005,51	136	144	7,24E+07
sp|O62823|CASA1_BUBBU	EPMIGVNQELAY	1362,65	133	144	2,29E+07
sp|O62823|CASA1_BUBBU	KEPMIGVNQELAY	1490,74	132	144	1,01E+07
sp|O62823|CASA1_BUBBU	QKEPMIGVNQELAY	1618,80	131	144	6,55E+07
sp|O62823|CASA1_BUBBU	QQKEPMIGVNQELAY	1746,86	130	144	0,00E+00
sp|O62823|CASA1_BUBBU	GIHAQQKEPMIGVNQELAY	2125,06	126	144	4,87E+06
sp|O62823|CASA1_BUBBU	EGIHAQQKEPMIGVNQELAY	2254,11	125	144	9,29E+06
sp|O62823|CASA1_BUBBU	KEGIHAQQKEPMIGVNQELAY	2382,20	124	144	1,60E+07

sp|O62823|CASA1_BUBBU	ELAYF	641,31	141	145	2,51E+06
sp|O62823|CASA1_BUBBU	QELAYF	769,36	140	145	1,21E+06
sp|O62823|CASA1_BUBBU	IGVNQELAYF	1152,58	136	145	8,95E+06
sp|O62823|CASA1_BUBBU	SMKEGIHAQQKEPMIGVNQELAYF	2747,34	122	145	2,55E+06
sp|O62823|CASA1_BUBBU	AYFYP	659,30	143	147	5,84E+07
sp|O62823|CASA1_BUBBU	AYFYPQ	787,35	143	148	5,03E+07
sp|O62823|CASA1_BUBBU	LAYFYPQ	900,44	142	148	3,84E+06
sp|O62823|CASA1_BUBBU	ELAYFYPQ	1029,48	141	148	0,00E+00
sp|O62823|CASA1_BUBBU	QELAYFYPQ	1157,54	140	148	2,07E+07
sp|O62823|CASA1_BUBBU	NQELAYFYPQ	1271,58	139	148	1,54E+06
sp|O62823|CASA1_BUBBU	IGVNQELAYFYPQ	1540,76	136	148	9,85E+06
sp|O62823|CASA1_BUBBU	FYPEL	667,32	145	149	1,48E+07
sp|O62823|CASA1_BUBBU	YFYPQL	829,40	144	149	1,77E+07
sp|O62823|CASA1_BUBBU	AYFYPQL	900,44	143	149	9,27E+07
sp|O62823|CASA1_BUBBU	AYFYPEL	901,42	143	149	6,17E+06
sp|O62823|CASA1_BUBBU	ELAYFYPQL	1142,56	141	149	1,88E+07
sp|O62823|CASA1_BUBBU	FRQFY	759,37	150	154	0,00E+00
sp|O62823|CASA1_BUBBU	QFYQL	697,34	152	156	2,81E+06
sp|O62823|CASA1_BUBBU	FRQFYQL	1000,51	150	156	3,54E+06
sp|O62823|CASA1_BUBBU	PQLFRQFYQLDAY	1687,84	147	159	1,92E+07
sp|O62823|CASA1_BUBBU	AYPSG	493,22	158	162	2,09E+07
sp|O62823|CASA1_BUBBU	QLDAYPSG	849,39	155	162	1,43E+06
sp|O62823|CASA1_BUBBU	YPSGA	493,22	159	163	3,10E+06
sp|O62823|CASA1_BUBBU	YQLDAYPSGA	1083,49	154	163	2,13E+06
sp|O62823|CASA1_BUBBU	YPSGAW	679,30	159	164	3,64E+07
sp|O62823|CASA1_BUBBU	AYPSGAW	750,33	158	164	3,55E+07
sp|O62823|CASA1_BUBBU	LDAYPSGAW	978,44	156	164	1,02E+08
sp|O62823|CASA1_BUBBU	QLDAYPSGAW	1106,50	155	164	1,19E+08
sp|O62823|CASA1_BUBBU	YQLDAYPSGAW	1269,57	154	164	2,35E+06
sp|O62823|CASA1_BUBBU	QYPDAPL	802,39	172	178	9,65E+05
sp|O62823|CASA1_BUBBU	GTQYPDAPS	934,40	170	178	3,08E+06
sp|O62823|CASA1_BUBBU	GTQYPDAPL	960,46	170	178	4,18E+06
sp|O62823|CASA1_BUBBU	VPLGTQYPDAPS	1243,61	167	178	6,88E+06
sp|O62823|CASA1_BUBBU	VPLGTQYPDAPL	1269,66	167	178	7,84E+06
sp|O62823|CASA1_BUBBU	YVPLGTQYPDAPS	1406,67	166	178	1,58E+07
sp|O62823|CASA1_BUBBU	TQYPDAPSF	1024,45	171	179	2,41E+06
sp|O62823|CASA1_BUBBU	TQYPDAPLF	1050,50	171	179	9,36E+05
sp|O62823|CASA1_BUBBU	GTQYPDAPSF	1081,47	170	179	8,47E+06
sp|O62823|CASA1_BUBBU	GTQYPDAPLF	1107,52	170	179	9,48E+06
sp|O62823|CASA1_BUBBU	VPLGTQYPDAPSF	1390,68	167	179	3,57E+06
sp|O62823|CASA1_BUBBU	YVPLGTQYPDAPLF	1579,79	166	179	1,07E+07
sp|O62823|CASA1_BUBBU	GTQYPDAPSFS	1168,50	170	180	9,38E+06
sp|O62823|CASA1_BUBBU	LGTQYPDAPSFS	1281,59	169	180	8,05E+06
sp|O62823|CASA1_BUBBU	LGTQYPDAPLFS	1307,64	169	180	1,93E+06
sp|O62823|CASA1_BUBBU	VPLGTQYPDAPSFS	1477,71	167	180	7,27E+06
sp|O62823|CASA1_BUBBU	YVPLGTQYPDAPSFS	1640,77	166	180	1,01E+07
sp|O62823|CASA1_BUBBU	PNPIGS	583,30	183	188	3,04E+06
sp|O62823|CASA1_BUBBU	DIPNPIGS	811,41	181	188	0,00E+00
sp|O62823|CASA1_BUBBU	NPIGSE	615,29	184	189	3,73E+07
sp|O62823|CASA1_BUBBU	PNPIGSE	712,34	183	189	1,21E+06
sp|O62823|CASA1_BUBBU	IPNPIGSE	825,42	182	189	5,31E+06
sp|O62823|CASA1_BUBBU	DIPNPIGSE	940,45	181	189	1,40E+08
sp|O62823|CASA1_BUBBU	SDIPNPIGSE	1027,48	180	189	1,08E+07
sp|O62823|CASA1_BUBBU	FSDIPNPIGSE	1174,55	179	189	1,11E+07
sp|O62823|CASA1_BUBBU	YVPLGTQYPDAPLFSDIPNPIGSE	2589,26	166	189	1,88E+07
sp|O62823|CASA1_BUBBU	DIPNPIGSEN	1054,49	181	190	3,51E+06
sp|O62823|CASA1_BUBBU	PIGSENSG	759,34	185	192	9,97E+05
sp|O62823|CASA1_BUBBU	IGSENSGK	790,38	186	193	6,63E+06
sp|O62823|CASA1_BUBBU	PNPIGSENSGK	1098,53	183	193	5,95E+06
sp|O62823|CASA1_BUBBU	IPNPIGSENSGK	1211,61	182	193	6,96E+06
tr|Q4F6X6|Q4F6X6_CASA1_BUBBU	DIPNPIGSENSEK	1398,66	181	193	2,33E+06
sp|O62823|CASA1_BUBBU	DIPNPIGSENSGK	1326,64	181	193	6,79E+07
sp|O62823|CASA1_BUBBU	SDIPNPIGSENSGK	1413,67	180	193	2,48E+06
sp|O62823|CASA1_BUBBU	NPIGSENSGKT	1102,53	184	194	2,42E+07
sp|O62823|CASA1_BUBBU	DIPNPIGSENSGKT	1427,69	181	194	5,14E+06
sp|O62823|CASA1_BUBBU	IGSENSGKTT	992,48	186	195	3,61E+07
sp|O62823|CASA1_BUBBU	PIGSENSGKTT	1089,53	185	195	3,64E+06
sp|O62823|CASA1_BUBBU	NPIGSENSGKTT	1203,57	184	195	4,98E+07
sp|O62823|CASA1_BUBBU	IPNPIGSENSGKTT	1413,71	182	195	1,38E+06
sp|O62823|CASA1_BUBBU	DIPNPIGSENSGKTT	1528,74	181	195	9,21E+06
sp|O62823|CASA1_BUBBU	IGSENSGKTTM	1123,52	186	196	6,00E+06
sp|O62823|CASA1_BUBBU	NPIGSENSGKTTM	1334,61	184	196	6,39E+06
sp|O62823|CASA1_BUBBU	IPNPIGSENSGKTTM	1544,75	182	196	1,94E+07
sp|O62823|CASA1_BUBBU	DIPNPIGSENSGKTTM	1659,78	181	196	3,16E+06
sp|O62823|CASA1_BUBBU	SDIPNPIGSENSGKTTM	1746,81	180	196	1,76E+06
sp|O62823|CASA1_BUBBU	GKTTMP	633,32	192	197	2,30E+06
sp|O62823|CASA1_BUBBU	NPIGSENSGKTTMP	1431,67	184	197	3,38E+06
sp|O62823|CASA1_BUBBU	DIPNPIGSENSGKTTMP	1756,83	181	197	8,94E+06
sp|O62823|CASA1_BUBBU	YYVPLGTQYPDAPSFSDIPNPIGSENSGKTTMP	3542,66	165	197	3,07E+07
sp|O62823|CASA1_BUBBU	TTMPL	561,28	194	198	3,82E+06
tr|Q4F6X6|Q4F6X6_CASA1_BUBBU	SEKTTMPL	905,45	191	198	9,81E+06
sp|O62823|CASA1_BUBBU	SGKTTMPL	833,43	191	198	1,58E+08
sp|O62823|CASA1_BUBBU	NSGKTTMPL	947,47	190	198	6,80E+07
sp|O62823|CASA1_BUBBU	IGSENSGKTTMPL	1333,65	186	198	2,29E+07
sp|O62823|CASA1_BUBBU	PIGSENSGKTTMPL	1430,71	185	198	2,17E+06
sp|O62823|CASA1_BUBBU	NPIGSENSGKTTMPL	1544,75	184	198	1,94E+07
sp|O62823|CASA1_BUBBU	IPNPIGSENSGKTTMPL	1754,89	182	198	1,07E+07
sp|O62823|CASA1_BUBBU	DIPNPIGSENSGKTTMPL	1869,91	181	198	1,01E+08
sp|O62823|CASA1_BUBBU	SDIPNPIGSENSGKTTMPL	1956,95	180	198	5,15E+07
sp|O62823|CASA1_BUBBU	FSDIPNPIGSENSGKTTMPL	2104,01	179	198	1,01E+07
sp|O62823|CASA1_BUBBU	APSFSDIPNPIGSENSGKTTMPL	2359,14	176	198	6,48E+06
sp|O62823|CASA1_BUBBU	GTQYPDAPSFSDIPNPIGSENSGKTTMPL	3020,41	170	198	3,56E+07
sp|O62823|CASA1_BUBBU	LGTQYPDAPSFSDIPNPIGSENSGKTTMPL	3133,49	169	198	1,88E+08
sp|O62823|CASA1_BUBBU	LGTQYPDAPLFSDIPNPIGSENSGKTTMPL	3159,54	169	198	5,25E+07
sp|O62823|CASA1_BUBBU	YYVPLGTQYPDAPSFSDIPNPIGSENSGKTTMPL	3655,74	165	198	5,90E+08
sp|O62823|CASA1_BUBBU	YYVPLGTQYPDAPLFSDIPNPIGSENSGKTTMPL	3681,79	165	198	2,08E+08
sp|O62823|CASA1_BUBBU	GKTTMPLW	932,48	192	199	2,33E+07
sp|O62823|CASA1_BUBBU	SGKTTMPLW	1019,51	191	199	1,80E+07

sp|O62823|CASA1_BUBBU	NSGKTTMPLW	1133,55	190	199	6,13E+06
sp|O62823|CASA1_BUBBU	IPNPIGSENSGKTTMPLW	1940,97	182	199	2,10E+06
sp|O62823|CASA1_BUBBU	DIPNPIGSENSGKTTMPLW	2055,99	181	199	6,10E+07
sp|O62823|CASA1_BUBBU	SDIPNPIGSENSGKTTMPLW	2143,03	180	199	3,14E+07
sp|O62823|CASA1_BUBBU	GTQYPDAPSFSDIPNPIGSENSGKTTMPLW	3206,49	170	199	1,55E+07
sp|O62823|CASA1_BUBBU	LGTQYPDAPSFSDIPNPIGSENSGKTTMPLW	3319,57	169	199	2,69E+07
sp|O62823|CASA1_BUBBU	VPLGTQYPDAPSFSDIPNPIGSENSGKTTMPLW	3515,69	167	199	1,71E+07
sp|O62823|CASA1_BUBBU	YVPLGTQYPDAPSFSDIPNPIGSENSGKTTMPLW	3678,76	166	199	8,38E+07
sp|O62823|CASA1_BUBBU	YYVPLGTQYPDAPSFSDIPNPIGSENSGKTTMPLW	3841,82	165	199	7,82E+07
sp|O62823|CASA1_BUBBU	YYVPLGTQYPDAPLFSDIPNPIGSENSGKTTMPLW	3867,87	165	199	1,34E+07
sp|P02663|CASA2_BOVIN	EVVRNANEEEY	1350,61	42	52	1,62E+06
sp|P02663|CASA2_BOVIN	KTKLTEEEKNRLNF	1748,94	150	163	1,60E+06
sp|P02663|CASA2_BOVIN	KTKLTEEEKNRLN	1601,87	150	162	5,37E+07
sp|P02663|CASA2_BOVIN	KTKLTEEEK	1104,60	150	158	1,51E+06
sp|P02663|CASA2_BOVIN	TKLTEEEKNRLNFLK	1862,03	151	165	5,58E+06
sp|P02663|CASA2_BOVIN	TKLTEEEKNRLN	1473,78	151	162	4,59E+07
sp|P02663|CASA2_BOVIN	TKLTEEEKNRL	1359,74	151	161	7,79E+06
sp|P02663|CASA2_BOVIN	TKLTEEEKNR	1246,65	151	160	1,56E+06
sp|P02663|CASA2_BOVIN	TKLTEEEKN	1090,55	151	159	4,13E+06
sp|P02663|CASA2_BOVIN	TKLTEEEK	976,51	151	158	5,28E+06
sp|P02663|CASA2_BOVIN	LTEEEKNRLNF	1391,70	153	163	7,17E+06
sp|P02663|CASA2_BOVIN	LTEEEKNRLN	1244,64	153	162	4,96E+07
sp|P02663|CASA2_BOVIN	TEEEKNRLNF	1278,62	154	163	3,07E+07
sp|P02663|CASA2_BOVIN	TEEEKNRLN	1131,55	154	162	2,85E+07
sp|P02663|CASA2_BOVIN	QKFALPQ	830,47	172	178	1,55E+07
sp|P02663|CASA2_BOVIN	ALPQY	590,31	175	179	1,20E+08
sp|P02663|CASA2_BOVIN	LPQYLKT	861,50	176	182	1,70E+06
sp|P02663|CASA2_BOVIN	LKTVYQHQKAMK	1473,81	180	191	8,28E+06
sp|P02663|CASA2_BOVIN	KPWIQPKT	996,58	191	198	3,79E+06
sp|P02663|CASA2_BOVIN	KPWIQPK	895,53	191	197	1,13E+07
sp|P02663|CASA2_BOVIN	PWIQPKT	868,48	192	198	3,77E+06
sp|P02663|CASA2_BOVIN	WIQPKTKVIPYVR	1626,96	193	205	1,53E+06
sp|P02663|CASA2_BOVIN	WIQPKTKVIPY	1371,79	193	203	1,24E+07
sp|P02663|CASA2_BOVIN	WIQPKTKVIP	1208,73	193	202	1,00E+07
sp|P02663|CASA2_BOVIN	WIQPKTK	899,52	193	199	3,84E+07
sp|P02663|CASA2_BOVIN	WIQPKT	771,43	193	198	1,57E+08
sp|P02663|CASA2_BOVIN	WIQPK	670,38	193	197	4,91E+06
sp|P02663|CASA2_BOVIN	IQPKTKVIPY	1185,71	194	203	1,93E+07
sp|P02663|CASA2_BOVIN	IQPKT	585,35	194	198	2,68E+07
tr|O62825|O62825_CAS2_BUBBU	KHTMEHVSSSEESIISQETYKQEK	2834,34	1	24	0,00E+00
tr|O62825|O62825_CAS2_BUBBU	KHTMEHVSSSEESIISQETYK	2449,14	1	21	0,00E+00
tr|O62825|O62825_CAS2_BUBBU	HTMEHVSSSEESIISQE	1928,84	2	18	0,00E+00
tr|O62825|O62825_CAS2_BUBBU	HVSSSEESIISQETYK	1822,86	6	21	0,00E+00
tr|O62825|O62825_CAS2_BUBBU	HVSSSEESIISQE	1430,65	6	18	7,36E+05
tr|O62825|O62825_CAS2_BUBBU	HVSSSEESIISQ	1301,61	6	17	0,00E+00
tr|O62825|O62825_CAS2_BUBBU	VSSSEESIISQETYK	1685,80	7	21	0,00E+00
tr|O62825|O62825_CAS2_BUBBU	SEESIISQE	1020,46	10	18	1,56E+06
tr|O62825|O62825_CAS2_BUBBU	SIISQETYKQEK	1452,75	13	24	0,00E+00
tr|O62825|O62825_CAS2_BUBBU	SIISQETYKQE	1324,65	13	23	0,00E+00
tr|O62825|O62825_CAS2_BUBBU	SIISQETYKQ	1195,61	13	22	0,00E+00
tr|O62825|O62825_CAS2_BUBBU	SIISQETYK	1067,55	13	21	0,00E+00
tr|O62825|O62825_CAS2_BUBBU	SIISQETY	939,45	13	20	0,00E+00
tr|O62825|O62825_CAS2_BUBBU	SIISQE	675,34	13	18	0,00E+00
tr|O62825|O62825_CAS2_BUBBU	IISQETYKQEK	1365,71	14	24	0,00E+00
tr|O62825|O62825_CAS2_BUBBU	IISQETYKQE	1237,62	14	23	0,00E+00
tr|O62825|O62825_CAS2_BUBBU	IISQETYK	980,52	14	21	1,35E+07
tr|O62825|O62825_CAS2_BUBBU	TYKQEK	795,41	19	24	1,81E+06
tr|O62825|O62825_CAS2_BUBBU	YKQEKNM	939,45	20	26	8,37E+06
tr|O62825|O62825_CAS2_BUBBU	QEKNMAIHPS	1153,56	22	31	0,00E+00
tr|O62825|O62825_CAS2_BUBBU	QEKNMAIHP	1066,52	22	30	0,00E+00
tr|O62825|O62825_CAS2_BUBBU	AIHPSKENL	1007,54	27	35	0,00E+00
tr|O62825|O62825_CAS2_BUBBU	AIHPSKE	780,41	27	33	0,00E+00
tr|O62825|O62825_CAS2_BUBBU	IHPSKENL	936,50	28	35	0,00E+00
tr|O62825|O62825_CAS2_BUBBU	HPSKENL	823,42	29	35	4,52E+07
tr|O62825|O62825_CAS2_BUBBU	EVIRNANEEEY	1364,62	42	52	2,99E+07
tr|O62825|O62825_CAS2_BUBBU	EVIRNANEEE	1201,56	42	51	1,28E+07
tr|O62825|O62825_CAS2_BUBBU	EVIRNANEE	1072,51	42	50	1,94E+07
tr|O62825|O62825_CAS2_BUBBU	EVIRNANE	943,47	42	49	1,74E+07
tr|O62825|O62825_CAS2_BUBBU	EVIRN	629,35	42	46	8,86E+06
tr|O62825|O62825_CAS2_BUBBU	VIRNANEE	943,47	43	50	3,73E+06
tr|O62825|O62825_CAS2_BUBBU	VIRNANE	814,43	43	49	1,17E+06
tr|O62825|O62825_CAS2_BUBBU	IRNANEEEY	1136,51	44	52	3,93E+07
tr|O62825|O62825_CAS2_BUBBU	RNANEEEY	1023,43	45	52	9,84E+06
tr|O62825|O62825_CAS2_BUBBU	VATEEVKIT	988,54	64	72	1,22E+08
tr|O62825|O62825_CAS2_BUBBU	VATEEVKI	887,50	64	71	1,10E+07
tr|O62825|O62825_CAS2_BUBBU	VATEEVK	774,41	64	70	1,60E+07
tr|O62825|O62825_CAS2_BUBBU	ATEEVKIT	889,48	65	72	1,08E+07
tr|O62825|O62825_CAS2_BUBBU	TEEVKITVDDKHYQKALN	2130,10	66	83	3,88E+06
tr|O62825|O62825_CAS2_BUBBU	TEEVKITVDDKHY	1575,78	66	78	0,00E+00
tr|O62825|O62825_CAS2_BUBBU	TEEVKITVDDK	1275,66	66	76	3,31E+06
tr|O62825|O62825_CAS2_BUBBU	TEEVKITVD	1032,53	66	74	7,80E+06
tr|O62825|O62825_CAS2_BUBBU	TEEVKIT	818,44	66	72	2,57E+08
tr|O62825|O62825_CAS2_BUBBU	EEVKITVDDKHYQKALN	2029,05	67	83	4,64E+06
tr|O62825|O62825_CAS2_BUBBU	EEVKITVD	931,49	67	74	1,31E+07
tr|O62825|O62825_CAS2_BUBBU	EVKITVDDKHYQKALNEINQFYQK	2950,52	68	91	2,16E+07
tr|O62825|O62825_CAS2_BUBBU	EVKITVDDKHYQKALNEINQFY	2694,37	68	89	2,33E+07
tr|O62825|O62825_CAS2_BUBBU	EVKITVDDKHYQKALNEINQF	2531,30	68	88	2,99E+07
tr|O62825|O62825_CAS2_BUBBU	EVKITVDDKHYQKALNEINQ	2384,23	68	87	5,71E+07
tr|O62825|O62825_CAS2_BUBBU	EVKITVDDKHYQKALNEIN	2256,18	68	86	7,39E+07
tr|O62825|O62825_CAS2_BUBBU	EVKITVDDKHYQKALN	1900,01	68	83	1,09E+08
tr|O62825|O62825_CAS2_BUBBU	EVKITVDDKHYQKAL	1785,96	68	82	3,75E+06
tr|O62825|O62825_CAS2_BUBBU	EVKITVDDKHYQKA	1672,88	68	81	5,08E+06
tr|O62825|O62825_CAS2_BUBBU	EVKITVDDKHYQK	1601,84	68	80	1,58E+06
tr|O62825|O62825_CAS2_BUBBU	EVKITVDDKHYQ	1473,75	68	79	2,31E+07
tr|O62825|O62825_CAS2_BUBBU	EVKITVDDKHY	1345,69	68	78	1,27E+08
tr|O62825|O62825_CAS2_BUBBU	EVKITVDDKH	1182,62	68	77	7,14E+07
tr|O62825|O62825_CAS2_BUBBU	EVKITVDDK	1045,57	68	76	6,70E+07
tr|O62825|O62825_CAS2_BUBBU	EVKITVD	802,44	68	74	2,16E+08

tr|O62825|O62825_CAS2_BUBBU	VKITVDDKHYQKALN	1770,96	69	83	7,48E+06
tr|O62825|O62825_CAS2_BUBBU	VKITVDDKHYQK	1472,80	69	80	1,09E+07
tr|O62825|O62825_CAS2_BUBBU	VKITVDDKHYQ	1344,70	69	79	2,28E+07
tr|O62825|O62825_CAS2_BUBBU	VKITVDDKHY	1216,65	69	78	4,59E+07
tr|O62825|O62825_CAS2_BUBBU	VKITVDDKH	1053,58	69	77	1,13E+07
tr|O62825|O62825_CAS2_BUBBU	VKITVDD	788,43	69	75	5,30E+06
tr|O62825|O62825_CAS2_BUBBU	KITVDDK	817,45	70	76	7,06E+07
tr|O62825|O62825_CAS2_BUBBU	ITVDDKHYQKALNEINQFYQK	2594,31	71	91	3,66E+07
tr|O62825|O62825_CAS2_BUBBU	ITVDDKHYQKALNEINQFYQ	2466,22	71	90	1,05E+07
tr|O62825|O62825_CAS2_BUBBU	ITVDDKHYQKALNEINQF	2175,10	71	88	1,30E+08
tr|O62825|O62825_CAS2_BUBBU	ITVDDKHYQKALNEINQ	2028,03	71	87	6,03E+07
tr|O62825|O62825_CAS2_BUBBU	ITVDDKHYQKALNEIN	1899,97	71	86	1,12E+08
tr|O62825|O62825_CAS2_BUBBU	ITVDDKHYQKALN	1543,80	71	83	6,68E+07
tr|O62825|O62825_CAS2_BUBBU	ITVDDKHYQKAL	1429,76	71	82	5,06E+06
tr|O62825|O62825_CAS2_BUBBU	ITVDDKHYQKA	1316,67	71	81	2,23E+07
tr|O62825|O62825_CAS2_BUBBU	ITVDDKHYQK	1245,64	71	80	2,08E+07
tr|O62825|O62825_CAS2_BUBBU	ITVDDKHYQ	1117,54	71	79	3,82E+07
tr|O62825|O62825_CAS2_BUBBU	ITVDDKHY	989,48	71	78	1,73E+08
tr|O62825|O62825_CAS2_BUBBU	ITVDDKH	826,42	71	77	2,08E+07
tr|O62825|O62825_CAS2_BUBBU	ITVDD	561,26	71	75	5,39E+06
tr|O62825|O62825_CAS2_BUBBU	TVDDKHYQKALNEINQFYQK	2481,23	72	91	1,63E+07
tr|O62825|O62825_CAS2_BUBBU	TVDDKHYQKALNEINQFYQ	2353,13	72	90	3,00E+06
tr|O62825|O62825_CAS2_BUBBU	TVDDKHYQKALNEINQFY	2225,08	72	89	5,85E+06
tr|O62825|O62825_CAS2_BUBBU	TVDDKHYQKALNEINQF	2062,01	72	88	4,02E+07
tr|O62825|O62825_CAS2_BUBBU	TVDDKHYQKALNEINQ	1914,94	72	87	1,04E+07
tr|O62825|O62825_CAS2_BUBBU	TVDDKHYQKALNEIN	1786,89	72	86	9,21E+06
tr|O62825|O62825_CAS2_BUBBU	TVDDKHYQKALNE	1559,76	72	84	1,86E+06
tr|O62825|O62825_CAS2_BUBBU	TVDDKHYQKALN	1430,72	72	83	7,42E+07
tr|O62825|O62825_CAS2_BUBBU	TVDDKHYQKA	1203,59	72	81	2,57E+06
tr|O62825|O62825_CAS2_BUBBU	TVDDKHYQK	1132,55	72	80	3,16E+06
tr|O62825|O62825_CAS2_BUBBU	TVDDKHYQ	1004,46	72	79	9,36E+06
tr|O62825|O62825_CAS2_BUBBU	TVDDKHY	876,40	72	78	1,21E+08
tr|O62825|O62825_CAS2_BUBBU	VDDKHYQKALNEINQFYQKFPQY	2915,42	73	95	2,89E+07
tr|O62825|O62825_CAS2_BUBBU	VDDKHYQKALNEINQFYQKFPQ	2752,36	73	94	7,84E+06
tr|O62825|O62825_CAS2_BUBBU	VDDKHYQKALNEINQFYQKFP	2624,30	73	93	5,22E+07
tr|O62825|O62825_CAS2_BUBBU	VDDKHYQKALNEINQFYQK	2380,18	73	91	8,37E+07
tr|O62825|O62825_CAS2_BUBBU	VDDKHYQKALNEINQFYQ	2252,09	73	90	1,43E+07
tr|O62825|O62825_CAS2_BUBBU	VDDKHYQKALNEINQFY	2124,03	73	89	8,42E+07
tr|O62825|O62825_CAS2_BUBBU	VDDKHYQKALNEINQF	1960,96	73	88	3,08E+08
tr|O62825|O62825_CAS2_BUBBU	VDDKHYQKALNEINQ	1813,90	73	87	6,93E+06
tr|O62825|O62825_CAS2_BUBBU	VDDKHYQKALNEIN	1685,84	73	86	2,91E+08
tr|O62825|O62825_CAS2_BUBBU	VDDKHYQKALNE	1458,71	73	84	1,83E+07
tr|O62825|O62825_CAS2_BUBBU	VDDKHYQKALN	1329,67	73	83	8,82E+08
tr|O62825|O62825_CAS2_BUBBU	VDDKHYQKAL	1215,62	73	82	9,90E+07
tr|O62825|O62825_CAS2_BUBBU	VDDKHYQKA	1102,54	73	81	2,00E+07
tr|O62825|O62825_CAS2_BUBBU	VDDKHYQK	1031,50	73	80	1,61E+07
tr|O62825|O62825_CAS2_BUBBU	DDKHYQKALNEINQFYQ	2153,02	74	90	1,53E+06
tr|O62825|O62825_CAS2_BUBBU	DDKHYQKALNEINQFY	2024,96	74	89	1,66E+07
tr|O62825|O62825_CAS2_BUBBU	DDKHYQKALNEINQF	1861,90	74	88	1,56E+07
tr|O62825|O62825_CAS2_BUBBU	DDKHYQKALNEINQ	1714,83	74	87	1,80E+07
tr|O62825|O62825_CAS2_BUBBU	DDKHYQKALNEIN	1586,77	74	86	1,31E+07
tr|O62825|O62825_CAS2_BUBBU	DDKHYQKALNE	1359,64	74	84	2,51E+06
tr|O62825|O62825_CAS2_BUBBU	DDKHYQKALN	1230,60	74	83	6,64E+07
tr|O62825|O62825_CAS2_BUBBU	DDKHYQKAL	1116,56	74	82	0,00E+00
tr|O62825|O62825_CAS2_BUBBU	DDKHYQ	804,34	74	79	4,96E+06
tr|O62825|O62825_CAS2_BUBBU	DKHYQKALNEINQFY	1909,93	75	89	4,47E+06
tr|O62825|O62825_CAS2_BUBBU	DKHYQKALNEINQF	1746,87	75	88	3,35E+06
tr|O62825|O62825_CAS2_BUBBU	DKHYQKALNEINQ	1599,80	75	87	6,00E+06
tr|O62825|O62825_CAS2_BUBBU	DKHYQKALNEIN	1471,74	75	86	1,83E+07
tr|O62825|O62825_CAS2_BUBBU	DKHYQKALNE	1244,61	75	84	6,92E+06
tr|O62825|O62825_CAS2_BUBBU	DKHYQKALN	1115,57	75	83	2,18E+07
tr|O62825|O62825_CAS2_BUBBU	DKHYQKAL	1001,53	75	82	1,02E+07
tr|O62825|O62825_CAS2_BUBBU	KHYQKALNEINQFYQK	2051,06	76	91	0,00E+00
tr|O62825|O62825_CAS2_BUBBU	HYQKALNEINQFYQKFP	2167,09	77	93	6,69E+06
tr|O62825|O62825_CAS2_BUBBU	HYQKALNEINQFYQK	1922,96	77	91	6,67E+06
tr|O62825|O62825_CAS2_BUBBU	HYQKALNEINQFYQ	1794,87	77	90	4,78E+06
tr|O62825|O62825_CAS2_BUBBU	HYQKALNEINQFY	1666,81	77	89	0,00E+00
tr|O62825|O62825_CAS2_BUBBU	HYQKALNEINQF	1503,75	77	88	5,21E+07
tr|O62825|O62825_CAS2_BUBBU	HYQKALNEINQ	1356,68	77	87	8,93E+06
tr|O62825|O62825_CAS2_BUBBU	HYQKALNEIN	1228,62	77	86	5,93E+07
tr|O62825|O62825_CAS2_BUBBU	HYQKALN	872,45	77	83	4,03E+07
tr|O62825|O62825_CAS2_BUBBU	HYQKAL	758,41	77	82	9,41E+07
tr|O62825|O62825_CAS2_BUBBU	YQKALNEINQFYQK	1785,91	78	91	7,72E+06
tr|O62825|O62825_CAS2_BUBBU	YQKALNEINQFYQ	1657,81	78	90	3,35E+06
tr|O62825|O62825_CAS2_BUBBU	YQKALNEINQFY	1529,75	78	89	3,44E+06
tr|O62825|O62825_CAS2_BUBBU	YQKALNEINQF	1366,69	78	88	5,47E+07
tr|O62825|O62825_CAS2_BUBBU	YQKALNEINQ	1219,62	78	87	8,93E+07
tr|O62825|O62825_CAS2_BUBBU	YQKALNEIN	1091,56	78	86	3,91E+07
tr|O62825|O62825_CAS2_BUBBU	YQKALNE	864,43	78	84	6,59E+07
tr|O62825|O62825_CAS2_BUBBU	QKALNEINQFYQKFP	1866,96	79	93	0,00E+00
tr|O62825|O62825_CAS2_BUBBU	QKALNEINQFYQK	1622,84	79	91	5,01E+07
tr|O62825|O62825_CAS2_BUBBU	QKALNEINQFYQ	1494,75	79	90	1,05E+07
tr|O62825|O62825_CAS2_BUBBU	QKALNEINQFY	1366,69	79	89	3,80E+07
tr|O62825|O62825_CAS2_BUBBU	QKALNEINQF	1203,62	79	88	2,02E+08
tr|O62825|O62825_CAS2_BUBBU	QKALNEINQ	1056,56	79	87	1,64E+08
tr|O62825|O62825_CAS2_BUBBU	QKALNEIN	928,50	79	86	1,42E+08
tr|O62825|O62825_CAS2_BUBBU	QKALNE	701,37	79	84	0,00E+00
tr|O62825|O62825_CAS2_BUBBU	KALNEINQFYQK	1494,78	80	91	1,10E+07
tr|O62825|O62825_CAS2_BUBBU	KALNEINQFY	1238,63	80	89	1,00E+07
tr|O62825|O62825_CAS2_BUBBU	KALNEINQF	1075,57	80	88	6,51E+07
tr|O62825|O62825_CAS2_BUBBU	KALNEINQ	928,50	80	87	1,14E+08
tr|O62825|O62825_CAS2_BUBBU	KALNEIN	800,44	80	86	2,88E+07
tr|O62825|O62825_CAS2_BUBBU	ALNEINQFYQKFPQY	1901,93	81	95	6,74E+06
tr|O62825|O62825_CAS2_BUBBU	ALNEINQFYQKFPQ	1738,87	81	94	0,00E+00
tr|O62825|O62825_CAS2_BUBBU	ALNEINQFYQKFP	1610,81	81	93	5,80E+06
tr|O62825|O62825_CAS2_BUBBU	ALNEINQFYQK	1366,69	81	91	7,92E+07
tr|O62825|O62825_CAS2_BUBBU	ALNEINQFYQ	1238,59	81	90	3,00E+07
tr|O62825|O62825_CAS2_BUBBU	ALNEINQFY	1110,53	81	89	2,83E+07

tr|O62825|O62825_CAS2_BUBBU	ALNEINQF	947,47	81	88	1,63E+08
tr|O62825|O62825_CAS2_BUBBU	ALNEINQ	800,40	81	87	3,00E+07
tr|O62825|O62825_CAS2_BUBBU	LNEINQFYQKFPQY	1830,89	82	95	3,25E+06
tr|O62825|O62825_CAS2_BUBBU	LNEINQFYQKFPQ	1667,83	82	94	6,86E+06
tr|O62825|O62825_CAS2_BUBBU	LNEINQFYQKFP	1539,77	82	93	3,13E+07
tr|O62825|O62825_CAS2_BUBBU	LNEINQFYQK	1295,65	82	91	1,21E+08
tr|O62825|O62825_CAS2_BUBBU	LNEINQFYQ	1167,56	82	90	1,15E+07
tr|O62825|O62825_CAS2_BUBBU	LNEINQFY	1039,50	82	89	1,86E+07
tr|O62825|O62825_CAS2_BUBBU	LNEINQF	876,43	82	88	1,04E+08
tr|O62825|O62825_CAS2_BUBBU	NEINQFYQKFPQYLQ	1958,95	83	97	1,42E+07
tr|O62825|O62825_CAS2_BUBBU	NEINQFYQKFPQYL	1830,89	83	96	6,05E+06
tr|O62825|O62825_CAS2_BUBBU	NEINQFYQKFPQY	1717,81	83	95	2,37E+07
tr|O62825|O62825_CAS2_BUBBU	NEINQFYQKFPQ	1554,75	83	94	2,53E+07
tr|O62825|O62825_CAS2_BUBBU	NEINQFYQKFP	1426,69	83	93	4,18E+07
tr|O62825|O62825_CAS2_BUBBU	NEINQFYQKF	1329,64	83	92	8,66E+06
tr|O62825|O62825_CAS2_BUBBU	NEINQFYQK	1182,57	83	91	4,17E+08
tr|O62825|O62825_CAS2_BUBBU	NEINQFYQ	1054,47	83	90	2,24E+07
tr|O62825|O62825_CAS2_BUBBU	NEINQFY	926,41	83	89	4,24E+07
tr|O62825|O62825_CAS2_BUBBU	NEINQF	763,35	83	88	1,00E+08
tr|O62825|O62825_CAS2_BUBBU	NEINQ	616,28	83	87	1,38E+06
tr|O62825|O62825_CAS2_BUBBU	EINQFYQKFPQYLQ	1844,91	84	97	1,30E+07
tr|O62825|O62825_CAS2_BUBBU	EINQFYQKFPQYL	1716,85	84	96	9,88E+06
tr|O62825|O62825_CAS2_BUBBU	EINQFYQKFPQY	1603,77	84	95	2,19E+07
tr|O62825|O62825_CAS2_BUBBU	EINQFYQKFPQ	1440,70	84	94	1,14E+07
tr|O62825|O62825_CAS2_BUBBU	EINQFYQKFP	1312,65	84	93	3,22E+07
tr|O62825|O62825_CAS2_BUBBU	EINQFYQK	1068,52	84	91	2,01E+08
tr|O62825|O62825_CAS2_BUBBU	EINQFYQ	940,43	84	90	1,38E+07
tr|O62825|O62825_CAS2_BUBBU	INQFYQKFPQYLQ	1715,87	85	97	2,45E+06
tr|O62825|O62825_CAS2_BUBBU	INQFYQKFPQY	1474,72	85	95	5,92E+06
tr|O62825|O62825_CAS2_BUBBU	INQFYQKF	1086,55	85	92	2,65E+06
tr|O62825|O62825_CAS2_BUBBU	INQFYQK	939,48	85	91	3,70E+08
tr|O62825|O62825_CAS2_BUBBU	INQFYQ	811,39	85	90	5,23E+07
tr|O62825|O62825_CAS2_BUBBU	INQFY	683,33	85	89	2,46E+08
tr|O62825|O62825_CAS2_BUBBU	NQFYQKFPQY	1361,64	86	95	4,14E+06
tr|O62825|O62825_CAS2_BUBBU	NQFYQKFPQ	1198,58	86	94	1,34E+06
tr|O62825|O62825_CAS2_BUBBU	NQFYQKF	973,47	86	92	2,52E+06
tr|O62825|O62825_CAS2_BUBBU	NQFYQK	826,40	86	91	8,81E+07
tr|O62825|O62825_CAS2_BUBBU	QFYQKFPQ	1084,53	87	94	3,12E+06
tr|O62825|O62825_CAS2_BUBBU	QFYQKF	859,42	87	92	5,71E+06
tr|O62825|O62825_CAS2_BUBBU	QFYQK	712,35	87	91	0,00E+00
tr|O62825|O62825_CAS2_BUBBU	FYQKFPQYLQY	1523,74	88	98	1,28E+06
tr|O62825|O62825_CAS2_BUBBU	FYQKFPQYLQ	1360,68	88	97	1,06E+06
tr|O62825|O62825_CAS2_BUBBU	FYQKFPQY	1119,54	88	95	3,30E+06
tr|O62825|O62825_CAS2_BUBBU	FYQKFPQ	956,48	88	94	3,66E+06
tr|O62825|O62825_CAS2_BUBBU	YQKFPQYLQ	1213,61	89	97	7,64E+07
tr|O62825|O62825_CAS2_BUBBU	YQKFPQYL	1085,55	89	96	7,36E+06
tr|O62825|O62825_CAS2_BUBBU	YQKFPQY	972,47	89	95	1,55E+08
tr|O62825|O62825_CAS2_BUBBU	YQKFPQ	809,41	89	94	2,25E+08
tr|O62825|O62825_CAS2_BUBBU	YQKFP	681,35	89	93	3,02E+07
tr|O62825|O62825_CAS2_BUBBU	QKFPQYLQY	1213,61	90	98	1,67E+08
tr|O62825|O62825_CAS2_BUBBU	QKFPQYLQ	1050,55	90	97	6,57E+07
tr|O62825|O62825_CAS2_BUBBU	QKFPQYL	922,49	90	96	3,87E+07
tr|O62825|O62825_CAS2_BUBBU	QKFPQY	809,41	90	95	1,92E+08
tr|O62825|O62825_CAS2_BUBBU	QKFPQ	646,34	90	94	0,00E+00
tr|O62825|O62825_CAS2_BUBBU	KFPQYL	794,43	91	96	1,01E+07
tr|O62825|O62825_CAS2_BUBBU	FPQYLQY	957,46	92	98	2,09E+08
tr|O62825|O62825_CAS2_BUBBU	FPQYLQ	794,40	92	97	1,02E+08
tr|O62825|O62825_CAS2_BUBBU	PQYLQY	810,39	93	98	3,69E+06
tr|O62825|O62825_CAS2_BUBBU	LQYLYQGPIVLNPWDQVKRNAVPITPTLN	3349,82	96	124	4,13E+07
tr|O62825|O62825_CAS2_BUBBU	LQYLYQGPIVLNPWDQVKRN	2443,30	96	115	1,54E+08
tr|O62825|O62825_CAS2_BUBBU	LQYLYQGPIVLNPWDQVKR	2329,26	96	114	2,64E+08
tr|O62825|O62825_CAS2_BUBBU	LQYLYQ	826,42	96	101	3,85E+06
tr|O62825|O62825_CAS2_BUBBU	QYLYQGPIVLNPWDQVKRNAVPITPTLN	3236,73	97	124	8,19E+07
tr|O62825|O62825_CAS2_BUBBU	QYLYQGPIVLNPWDQVKRNAVPITPT	3009,61	97	122	1,36E+08
tr|O62825|O62825_CAS2_BUBBU	QYLYQGPIVLNPWDQVKRNA	2401,25	97	116	1,49E+07
tr|O62825|O62825_CAS2_BUBBU	QYLYQGPIVLNPWDQVKRN	2330,22	97	115	1,77E+08
tr|O62825|O62825_CAS2_BUBBU	QYLYQGPIVLNPWDQVKR	2216,17	97	114	3,32E+08
tr|O62825|O62825_CAS2_BUBBU	QYLYQGPIVLNPWDQVK	2060,07	97	113	0,00E+00
tr|O62825|O62825_CAS2_BUBBU	QYLYQGPIVLNPW	1589,82	97	109	0,00E+00
tr|O62825|O62825_CAS2_BUBBU	YLYQGPIVLNPWDQVKRNAVPITPT	2881,55	98	122	9,52E+07
tr|O62825|O62825_CAS2_BUBBU	YLYQGPIVLNPWDQVKRNA	2273,20	98	116	3,78E+07
tr|O62825|O62825_CAS2_BUBBU	YLYQGPIVLNPWDQVKRN	2202,16	98	115	2,12E+08
tr|O62825|O62825_CAS2_BUBBU	YLYQGPIVLNPWDQVKR	2088,12	98	114	5,66E+08
tr|O62825|O62825_CAS2_BUBBU	YLYQGPIVLNPWDQVK	1932,01	98	113	2,21E+08
tr|O62825|O62825_CAS2_BUBBU	LYQGPIVLNPWDQVKRNAVPITPTLNREQ	3358,82	99	127	4,04E+06
tr|O62825|O62825_CAS2_BUBBU	LYQGPIVLNPWDQVKRNAVPITPTLNRE	3230,76	99	126	6,69E+07
tr|O62825|O62825_CAS2_BUBBU	LYQGPIVLNPWDQVKRNAVPITPTLNR	3101,71	99	125	5,24E+08
tr|O62825|O62825_CAS2_BUBBU	LYQGPIVLNPWDQVKRNAVPITPTLN	2945,61	99	124	3,55E+09
tr|O62825|O62825_CAS2_BUBBU	LYQGPIVLNPWDQVKRNAVPITPTL	2831,57	99	123	5,08E+08
tr|O62825|O62825_CAS2_BUBBU	LYQGPIVLNPWDQVKRNAVPITPT	2718,49	99	122	1,73E+08
tr|O62825|O62825_CAS2_BUBBU	LYQGPIVLNPWDQVKRNAVPITP	2617,44	99	121	5,64E+08
tr|O62825|O62825_CAS2_BUBBU	LYQGPIVLNPWDQVKRNAVPIT	2520,39	99	120	9,29E+07
tr|O62825|O62825_CAS2_BUBBU	LYQGPIVLNPWDQVKRNAVPI	2419,34	99	119	4,94E+07
tr|O62825|O62825_CAS2_BUBBU	LYQGPIVLNPWDQVKRNAVP	2306,25	99	118	3,67E+07
tr|O62825|O62825_CAS2_BUBBU	LYQGPIVLNPWDQVKRNAV	2209,20	99	117	1,09E+07
tr|O62825|O62825_CAS2_BUBBU	LYQGPIVLNPWDQVKRNA	2110,13	99	116	1,98E+08
tr|O62825|O62825_CAS2_BUBBU	LYQGPIVLNPWDQVKRN	2039,10	99	115	1,94E+09
tr|O62825|O62825_CAS2_BUBBU	LYQGPIVLNPWDQVKR	1925,05	99	114	3,43E+09
tr|O62825|O62825_CAS2_BUBBU	LYQGPIVLNPWDQVK	1768,95	99	113	5,02E+08
tr|O62825|O62825_CAS2_BUBBU	LYQGP	576,29	99	103	3,69E+08
tr|O62825|O62825_CAS2_BUBBU	YQGPIVLNPWDQVKRNAVPITPTLNRE	3117,67	100	126	2,22E+07
tr|O62825|O62825_CAS2_BUBBU	YQGPIVLNPWDQVKRNAVPITPTLNR	2988,63	100	125	1,18E+08
tr|O62825|O62825_CAS2_BUBBU	YQGPIVLNPWDQVKRNAVPITPTLN	2832,53	100	124	3,81E+07
tr|O62825|O62825_CAS2_BUBBU	YQGPIVLNPWDQVKRNAVPITPTL	2718,49	100	123	4,67E+08
tr|O62825|O62825_CAS2_BUBBU	YQGPIVLNPWDQVKRNAVPITPT	2605,40	100	122	1,56E+09
tr|O62825|O62825_CAS2_BUBBU	YQGPIVLNPWDQVKRNAVPITP	2504,35	100	121	2,42E+08
tr|O62825|O62825_CAS2_BUBBU	YQGPIVLNPWDQVKRNAVPIT	2407,30	100	120	4,50E+07
tr|O62825|O62825_CAS2_BUBBU	YQGPIVLNPWDQVKRNAVPI	2306,25	100	119	6,89E+07

tr|O62825|O62825_CAS2_BUBBU	YQGPIVLNPWDQVKRNAVP	2193,17	100	118	1,05E+08
tr|O62825|O62825_CAS2_BUBBU	YQGPIVLNPWDQVKRNAV	2096,12	100	117	7,02E+06
tr|O62825|O62825_CAS2_BUBBU	YQGPIVLNPWDQVKRNA	1997,05	100	116	1,95E+08
tr|O62825|O62825_CAS2_BUBBU	YQGPIVLNPWDQVKRN	1926,01	100	115	2,84E+09
tr|O62825|O62825_CAS2_BUBBU	YQGPIVLNPWDQVKR	1811,97	100	114	5,75E+09
tr|O62825|O62825_CAS2_BUBBU	YQGPIVLNPWDQVK	1655,87	100	113	1,25E+09
tr|O62825|O62825_CAS2_BUBBU	YQGPIVL	788,44	100	106	1,50E+07
tr|O62825|O62825_CAS2_BUBBU	QGPIVLNPWDQVKRNAVPITPTLNR	2825,57	101	125	1,71E+07
tr|O62825|O62825_CAS2_BUBBU	QGPIVLNPWDQVKRNAVPITPTLN	2669,47	101	124	2,32E+08
tr|O62825|O62825_CAS2_BUBBU	QGPIVLNPWDQVKRNAVPITPT	2442,34	101	122	1,96E+08
tr|O62825|O62825_CAS2_BUBBU	QGPIVLNPWDQVKRNAV	1933,05	101	117	5,74E+06
tr|O62825|O62825_CAS2_BUBBU	QGPIVLNPWDQVKRNA	1833,99	101	116	1,85E+08
tr|O62825|O62825_CAS2_BUBBU	QGPIVLNPWDQVKRN	1762,95	101	115	2,26E+09
tr|O62825|O62825_CAS2_BUBBU	QGPIVLNPWDQVKR	1648,90	101	114	4,08E+09
tr|O62825|O62825_CAS2_BUBBU	QGPIVLNPWDQVK	1492,80	101	113	2,59E+09
tr|O62825|O62825_CAS2_BUBBU	QGPIVLNPWDQV	1364,71	101	112	1,30E+08
tr|O62825|O62825_CAS2_BUBBU	QGPIVLNPWDQ	1265,64	101	111	3,16E+07
tr|O62825|O62825_CAS2_BUBBU	QGPIVLNPWD	1137,58	101	110	3,33E+07
tr|O62825|O62825_CAS2_BUBBU	QGPIVLNPW	1022,55	101	109	1,41E+07
tr|O62825|O62825_CAS2_BUBBU	QGPIVLNP	836,48	101	108	1,39E+07
tr|O62825|O62825_CAS2_BUBBU	GPIVLNPWDQVKRNAVPITPTLNR	2697,51	102	125	3,41E+07
tr|O62825|O62825_CAS2_BUBBU	GPIVLNPWDQVKRNAVPITPTLN	2541,41	102	124	9,90E+08
tr|O62825|O62825_CAS2_BUBBU	GPIVLNPWDQVKRNAVPITPTL	2427,36	102	123	1,66E+08
tr|O62825|O62825_CAS2_BUBBU	GPIVLNPWDQVKRNAVPITPT	2314,28	102	122	5,37E+08
tr|O62825|O62825_CAS2_BUBBU	GPIVLNPWDQVKRNAVPITP	2213,23	102	121	4,92E+07
tr|O62825|O62825_CAS2_BUBBU	GPIVLNPWDQVKRNAVPIT	2116,18	102	120	1,32E+07
tr|O62825|O62825_CAS2_BUBBU	GPIVLNPWDQVKRNAVPI	2015,13	102	119	1,94E+07
tr|O62825|O62825_CAS2_BUBBU	GPIVLNPWDQVKRNAVP	1902,05	102	118	3,66E+06
tr|O62825|O62825_CAS2_BUBBU	GPIVLNPWDQVKRNA	1705,93	102	116	1,66E+08
tr|O62825|O62825_CAS2_BUBBU	GPIVLNPWDQVKRN	1634,89	102	115	2,66E+09
tr|O62825|O62825_CAS2_BUBBU	GPIVLNPWDQVKR	1520,85	102	114	5,37E+09
tr|O62825|O62825_CAS2_BUBBU	GPIVLNPWDQVK	1364,75	102	113	4,62E+09
tr|O62825|O62825_CAS2_BUBBU	GPIVLNPWDQV	1236,65	102	112	2,62E+08
tr|O62825|O62825_CAS2_BUBBU	GPIVLNPWDQ	1137,58	102	111	5,46E+07
tr|O62825|O62825_CAS2_BUBBU	GPIVLNPWD	1009,52	102	110	3,91E+07
tr|O62825|O62825_CAS2_BUBBU	GPIVLNPW	894,50	102	109	4,61E+07
tr|O62825|O62825_CAS2_BUBBU	PIVLNPWDQVKRN	1577,87	103	115	7,65E+06
tr|O62825|O62825_CAS2_BUBBU	PIVLNPWDQVKR	1463,82	103	114	2,23E+06
tr|O62825|O62825_CAS2_BUBBU	PIVLNPWDQVK	1307,72	103	113	1,18E+07
tr|O62825|O62825_CAS2_BUBBU	IVLNPWDQVKRNAVPITPTLNRE	2672,48	104	126	4,03E+07
tr|O62825|O62825_CAS2_BUBBU	IVLNPWDQVKRNAVPITPTLNR	2543,43	104	125	4,04E+08
tr|O62825|O62825_CAS2_BUBBU	IVLNPWDQVKRNAVPITPTLN	2387,33	104	124	2,33E+09
tr|O62825|O62825_CAS2_BUBBU	IVLNPWDQVKRNAVPITPTL	2273,29	104	123	1,00E+09
tr|O62825|O62825_CAS2_BUBBU	IVLNPWDQVKRNAVPITPT	2160,21	104	122	3,15E+09
tr|O62825|O62825_CAS2_BUBBU	IVLNPWDQVKRNAVPITP	2059,16	104	121	2,38E+08
tr|O62825|O62825_CAS2_BUBBU	IVLNPWDQVKRNAVPIT	1962,11	104	120	5,56E+07
tr|O62825|O62825_CAS2_BUBBU	IVLNPWDQVKRNAVPI	1861,06	104	119	3,71E+07
tr|O62825|O62825_CAS2_BUBBU	IVLNPWDQVKRNAVP	1747,97	104	118	6,36E+07
tr|O62825|O62825_CAS2_BUBBU	IVLNPWDQVKRNAV	1650,92	104	117	3,54E+05
tr|O62825|O62825_CAS2_BUBBU	IVLNPWDQVKRNA	1551,85	104	116	3,98E+08
tr|O62825|O62825_CAS2_BUBBU	IVLNPWDQVKRN	1480,82	104	115	4,88E+09
tr|O62825|O62825_CAS2_BUBBU	IVLNPWDQVKR	1366,77	104	114	5,82E+09
tr|O62825|O62825_CAS2_BUBBU	IVLNPWDQVK	1210,67	104	113	3,68E+09
tr|O62825|O62825_CAS2_BUBBU	IVLNPWDQV	1082,58	104	112	8,69E+07
tr|O62825|O62825_CAS2_BUBBU	IVLNPWDQ	983,51	104	111	1,24E+07
tr|O62825|O62825_CAS2_BUBBU	IVLNPWD	855,45	104	110	5,75E+07
tr|O62825|O62825_CAS2_BUBBU	IVLNP	554,34	104	108	1,50E+06
tr|O62825|O62825_CAS2_BUBBU	VLNPWDQVKRNAVPITPTLN	2274,25	105	124	4,73E+07
tr|O62825|O62825_CAS2_BUBBU	VLNPWDQVKRNAVPITPT	2047,12	105	122	1,76E+06
tr|O62825|O62825_CAS2_BUBBU	VLNPWDQVKRNA	1438,77	105	116	1,18E+08
tr|O62825|O62825_CAS2_BUBBU	VLNPWDQVKRN	1367,73	105	115	5,60E+08
tr|O62825|O62825_CAS2_BUBBU	VLNPWDQVKR	1253,69	105	114	8,24E+08
tr|O62825|O62825_CAS2_BUBBU	VLNPWDQVK	1097,59	105	113	9,41E+08
tr|O62825|O62825_CAS2_BUBBU	VLNPWDQV	969,49	105	112	3,25E+07
tr|O62825|O62825_CAS2_BUBBU	LNPWDQVKRNA	1339,70	106	116	0,00E+00
tr|O62825|O62825_CAS2_BUBBU	LNPWDQVKRN	1268,66	106	115	1,29E+08
tr|O62825|O62825_CAS2_BUBBU	LNPWDQVKR	1154,62	106	114	6,41E+08
tr|O62825|O62825_CAS2_BUBBU	LNPWDQVK	998,52	106	113	3,70E+08
tr|O62825|O62825_CAS2_BUBBU	LNPWDQV	870,42	106	112	1,69E+07
tr|O62825|O62825_CAS2_BUBBU	LNPWDQ	771,36	106	111	1,73E+06
tr|O62825|O62825_CAS2_BUBBU	LNPWD	643,30	106	110	8,84E+06
tr|O62825|O62825_CAS2_BUBBU	NPWDQVKRNAVPITPTLN	2062,10	107	124	1,10E+07
tr|O62825|O62825_CAS2_BUBBU	NPWDQVKRNAVPITPTL	1948,05	107	123	5,11E+06
tr|O62825|O62825_CAS2_BUBBU	NPWDQVKRNAVPITPT	1834,97	107	122	2,41E+07
tr|O62825|O62825_CAS2_BUBBU	NPWDQVKRNA	1226,62	107	116	4,04E+06
tr|O62825|O62825_CAS2_BUBBU	NPWDQVKRN	1155,58	107	115	8,27E+07
tr|O62825|O62825_CAS2_BUBBU	NPWDQVKR	1041,54	107	114	9,70E+07
tr|O62825|O62825_CAS2_BUBBU	NPWDQVK	885,43	107	113	6,74E+08
tr|O62825|O62825_CAS2_BUBBU	NPWDQV	757,34	107	112	1,62E+07
tr|O62825|O62825_CAS2_BUBBU	PWDQVKRN	1041,54	108	115	1,50E+07
tr|O62825|O62825_CAS2_BUBBU	PWDQVKR	927,49	108	114	5,12E+07
tr|O62825|O62825_CAS2_BUBBU	PWDQVK	771,39	108	113	1,29E+06
tr|O62825|O62825_CAS2_BUBBU	WDQVKRNAVPITPTL	1736,96	109	123	0,00E+00
tr|O62825|O62825_CAS2_BUBBU	WDQVKRNAVPITPT	1623,87	109	122	2,87E+07
tr|O62825|O62825_CAS2_BUBBU	WDQVKRNAVPITP	1522,83	109	121	0,00E+00
tr|O62825|O62825_CAS2_BUBBU	WDQVKRNA	1015,52	109	116	7,56E+07
tr|O62825|O62825_CAS2_BUBBU	WDQVKRN	944,48	109	115	4,10E+08
tr|O62825|O62825_CAS2_BUBBU	WDQVKR	830,44	109	114	6,97E+08
tr|O62825|O62825_CAS2_BUBBU	WDQVK	674,34	109	113	7,92E+05
tr|O62825|O62825_CAS2_BUBBU	DQVKRNAVPITPTLN	1664,92	110	124	2,79E+06
tr|O62825|O62825_CAS2_BUBBU	DQVKRNAVPITPT	1437,79	110	122	5,01E+06
tr|O62825|O62825_CAS2_BUBBU	DQVKRN	758,40	110	115	5,36E+07
tr|O62825|O62825_CAS2_BUBBU	QVKRNAVPITPT	1322,77	111	122	3,94E+06
tr|O62825|O62825_CAS2_BUBBU	VKRNAVPITPTLN	1421,84	112	124	3,07E+06
tr|O62825|O62825_CAS2_BUBBU	VKRNAVPITPTL	1307,79	112	123	2,51E+06
tr|O62825|O62825_CAS2_BUBBU	VKRNAVPITPT	1194,71	112	122	1,65E+07
tr|O62825|O62825_CAS2_BUBBU	RNAVPITPTLNR	1350,77	114	125	6,28E+05
tr|O62825|O62825_CAS2_BUBBU	RNAVPITPTLN	1194,67	114	124	2,38E+08

tr|O62825|O62825_CAS2_BUBBU	RNAVPITPTL	1080,63	114	123	4,51E+06
tr|O62825|O62825_CAS2_BUBBU	RNAVPITPT	967,55	114	122	2,28E+07
tr|O62825|O62825_CAS2_BUBBU	NAVPITPTLNREQLSTSEENSKK	2555,32	115	137	8,29E+06
tr|O62825|O62825_CAS2_BUBBU	NAVPITPTLNREQLSTSEENSK	2427,22	115	136	6,78E+06
tr|O62825|O62825_CAS2_BUBBU	NAVPITPTLNREQLSTS	1839,97	115	131	1,09E+06
tr|O62825|O62825_CAS2_BUBBU	NAVPITPTLNREQLST	1752,94	115	130	0,00E+00
tr|O62825|O62825_CAS2_BUBBU	NAVPITPTLNREQL	1564,86	115	128	3,52E+06
tr|O62825|O62825_CAS2_BUBBU	NAVPITPTLNREQ	1451,77	115	127	1,49E+06
tr|O62825|O62825_CAS2_BUBBU	NAVPITPTLNRE	1323,71	115	126	4,44E+07
tr|O62825|O62825_CAS2_BUBBU	NAVPITPTLNR	1194,67	115	125	2,17E+08
tr|O62825|O62825_CAS2_BUBBU	NAVPITPTLN	1038,57	115	124	3,84E+08
tr|O62825|O62825_CAS2_BUBBU	NAVPITPTL	924,53	115	123	1,67E+07
tr|O62825|O62825_CAS2_BUBBU	NAVPITPT	811,44	115	122	9,68E+07
tr|O62825|O62825_CAS2_BUBBU	NAVPITP	710,40	115	121	3,88E+07
tr|O62825|O62825_CAS2_BUBBU	NAVPI	512,30	115	119	7,69E+06
tr|O62825|O62825_CAS2_BUBBU	AVPITPTLNREQLSTSEENSK	2313,18	116	136	4,33E+06
tr|O62825|O62825_CAS2_BUBBU	AVPITPTLNREQL	1450,81	116	128	1,36E+07
tr|O62825|O62825_CAS2_BUBBU	AVPITPTLNREQ	1337,73	116	127	3,52E+06
tr|O62825|O62825_CAS2_BUBBU	AVPITPTLNRE	1209,67	116	126	9,14E+07
tr|O62825|O62825_CAS2_BUBBU	AVPITPTLNR	1080,63	116	125	1,33E+09
tr|O62825|O62825_CAS2_BUBBU	AVPITPTLN	924,53	116	124	7,56E+08
tr|O62825|O62825_CAS2_BUBBU	AVPITPTL	810,49	116	123	1,24E+08
tr|O62825|O62825_CAS2_BUBBU	AVPITPT	697,40	116	122	5,18E+08
tr|O62825|O62825_CAS2_BUBBU	VPITPTLNREQ	1266,69	117	127	1,34E+06
tr|O62825|O62825_CAS2_BUBBU	VPITPTLNRE	1138,63	117	126	2,89E+07
tr|O62825|O62825_CAS2_BUBBU	VPITPTLNR	1009,59	117	125	1,99E+08
tr|O62825|O62825_CAS2_BUBBU	VPITPTLN	853,49	117	124	8,78E+07
tr|O62825|O62825_CAS2_BUBBU	VPITPTL	739,45	117	123	1,92E+07
tr|O62825|O62825_CAS2_BUBBU	VPITPT	626,36	117	122	7,82E+07
tr|O62825|O62825_CAS2_BUBBU	PITPTLNR	910,52	118	125	5,18E+07
tr|O62825|O62825_CAS2_BUBBU	PITPTLN	754,42	118	124	2,59E+07
tr|O62825|O62825_CAS2_BUBBU	ITPTLNREQLSTS	1458,77	119	131	1,06E+06
tr|O62825|O62825_CAS2_BUBBU	ITPTLNRE	942,51	119	126	3,66E+07
tr|O62825|O62825_CAS2_BUBBU	ITPTLNR	813,47	119	125	3,32E+08
tr|O62825|O62825_CAS2_BUBBU	TPTLNREQ	957,49	120	127	0,00E+00
tr|O62825|O62825_CAS2_BUBBU	TPTLNRE	829,43	120	126	1,15E+07
tr|O62825|O62825_CAS2_BUBBU	TPTLNR	700,39	120	125	1,62E+06
tr|O62825|O62825_CAS2_BUBBU	TPTLN	544,29	120	124	2,46E+06
tr|O62825|O62825_CAS2_BUBBU	NREQLSTSEENSKK	1648,80	124	137	0,00E+00
tr|O62825|O62825_CAS2_BUBBU	REQLSTSEENSKKTVDMESTEVITKKTKLTEEDKNRLN	4438,25	125	162	0,00E+00
tr|E9NZN2|E9NZN2_CASA2_BUBBU	REQLSTSEENSKKTVDMESTEVFTKK	3030,48	125	150	0,00E+00
tr|O62825|O62825_CAS2_BUBBU	REQLSTSEENSKKTVDMESTEVITKK	2996,50	125	150	0,00E+00
tr|E9NZN2|E9NZN2_CASA2_BUBBU	REQLSTSEENSKKTVDMESTEVFTK	2902,39	125	149	0,00E+00
tr|O62825|O62825_CAS2_BUBBU	REQLSTSEENSKKTVDMESTEVITK	2868,40	125	149	0,00E+00
tr|O62825|O62825_CAS2_BUBBU	REQLSTSEENSKKTVDME	2109,98	125	142	0,00E+00
tr|O62825|O62825_CAS2_BUBBU	REQLSTSEENSKKTVD	1849,90	125	140	0,00E+00
tr|O62825|O62825_CAS2_BUBBU	REQLSTSEENSKKTV	1734,87	125	139	0,00E+00
tr|O62825|O62825_CAS2_BUBBU	REQLSTSEENSKKT	1635,81	125	138	3,65E+07
tr|O62825|O62825_CAS2_BUBBU	REQLSTSEENSKK	1534,76	125	137	2,87E+07
tr|O62825|O62825_CAS2_BUBBU	REQLSTSEENSK	1406,66	125	136	6,14E+06
tr|O62825|O62825_CAS2_BUBBU	REQLSTSEEN	1191,54	125	134	3,66E+06
tr|E9NZN2|E9NZN2_CASA2_BUBBU	EQLSTSEENSKKTVDMESTEVFTK	2746,29	126	149	0,00E+00
tr|O62825|O62825_CAS2_BUBBU	EQLSTSEENSKKTVDMESTEVITK	2712,30	126	149	0,00E+00
tr|O62825|O62825_CAS2_BUBBU	EQLSTSEENSKKTVDME	1953,88	126	142	1,14E+07
tr|O62825|O62825_CAS2_BUBBU	EQLSTSEENSKKTVD	1693,80	126	140	0,00E+00
tr|O62825|O62825_CAS2_BUBBU	EQLSTSEENSKKT	1479,71	126	138	0,00E+00
tr|O62825|O62825_CAS2_BUBBU	EQLSTSEENSKK	1378,66	126	137	0,00E+00
tr|O62825|O62825_CAS2_BUBBU	EQLSTSEENSK	1250,56	126	136	8,56E+06
tr|O62825|O62825_CAS2_BUBBU	QLSTSEENSKKTVDME	1824,84	127	142	9,04E+06
tr|O62825|O62825_CAS2_BUBBU	QLSTSEENSKKTVD	1564,76	127	140	0,00E+00
tr|O62825|O62825_CAS2_BUBBU	QLSTSEENSKKTV	1449,73	127	139	0,00E+00
tr|O62825|O62825_CAS2_BUBBU	QLSTSEENSKKT	1350,66	127	138	1,50E+06
tr|O62825|O62825_CAS2_BUBBU	QLSTSEENSKK	1249,62	127	137	0,00E+00
tr|O62825|O62825_CAS2_BUBBU	QLSTSEENSK	1121,52	127	136	0,00E+00
tr|O62825|O62825_CAS2_BUBBU	LSTSEENSKKTVDMESTEVITKKTKLTEEDKNRLN	4025,05	128	162	0,00E+00
tr|O62825|O62825_CAS2_BUBBU	LSTSEENSKKTVDMESTEVITKK	2583,29	128	150	3,99E+06
tr|O62825|O62825_CAS2_BUBBU	LSTSEENSKKTVDMESTEVITK	2455,20	128	149	0,00E+00
tr|O62825|O62825_CAS2_BUBBU	LSTSEENSKKTVDMESTE	2013,90	128	145	0,00E+00
tr|O62825|O62825_CAS2_BUBBU	LSTSEENSKKTVDME	1696,78	128	142	2,75E+07
tr|O62825|O62825_CAS2_BUBBU	LSTSEENSKKTVD	1436,70	128	140	3,13E+06
tr|O62825|O62825_CAS2_BUBBU	LSTSEENSKKTV	1321,67	128	139	0,00E+00
tr|O62825|O62825_CAS2_BUBBU	LSTSEENSKKT	1222,60	128	138	2,76E+07
tr|O62825|O62825_CAS2_BUBBU	LSTSEENSKK	1121,56	128	137	4,46E+06
tr|O62825|O62825_CAS2_BUBBU	LSTSEENSK	993,46	128	136	2,23E+06
tr|O62825|O62825_CAS2_BUBBU	STSEENSKKTVDME	1583,70	129	142	0,00E+00
tr|O62825|O62825_CAS2_BUBBU	STSEENSKKTVD	1323,62	129	140	1,57E+06
tr|O62825|O62825_CAS2_BUBBU	STSEENSKKTV	1208,59	129	139	0,00E+00
tr|O62825|O62825_CAS2_BUBBU	TSEENSKKTVDME	1496,67	130	142	6,89E+06
tr|O62825|O62825_CAS2_BUBBU	TSEENSKKTVD	1236,58	130	140	1,85E+06
tr|O62825|O62825_CAS2_BUBBU	TSEENSKKTV	1121,56	130	139	0,00E+00
tr|O62825|O62825_CAS2_BUBBU	SEENSKKTVDME	1395,62	131	142	7,20E+06
tr|O62825|O62825_CAS2_BUBBU	EENSKKTVDME	1308,59	132	142	7,26E+06
tr|O62825|O62825_CAS2_BUBBU	NSKKTVDME	1050,50	134	142	3,15E+06
tr|O62825|O62825_CAS2_BUBBU	SKKTVDME	936,46	135	142	3,31E+06
tr|O62825|O62825_CAS2_BUBBU	KTVDMESTEVITKKTKLTEEDKNRLN	3049,60	137	162	0,00E+00
tr|O62825|O62825_CAS2_BUBBU	KTVDMESTEVITKKTK	1836,99	137	152	0,00E+00
tr|E9NZN2|E9NZN2_CASA2_BUBBU	KTVDMESTEVFTKK	1641,83	137	150	0,00E+00
tr|O62825|O62825_CAS2_BUBBU	KTVDMESTEVITKK	1607,84	137	150	0,00E+00
tr|E9NZN2|E9NZN2_CASA2_BUBBU	KTVDMESTEVFTK	1513,73	137	149	0,00E+00
tr|O62825|O62825_CAS2_BUBBU	TVDMESTEVITKKTKLTEEDKNRLNFLK	3309,75	138	165	0,00E+00
tr|O62825|O62825_CAS2_BUBBU	TVDMESTEVITKKTKLTEEDKNRLN	2921,50	138	162	0,00E+00
tr|E9NZN2|E9NZN2_CASA2_BUBBU	TVDMESTEVFTKK	1513,73	138	150	0,00E+00
tr|O62825|O62825_CAS2_BUBBU	TVDMESTEVITKK	1479,75	138	150	7,01E+06
tr|E9NZN2|E9NZN2_CASA2_BUBBU	TVDMESTEVFTK	1385,64	138	149	0,00E+00
tr|O62825|O62825_CAS2_BUBBU	TVDMESTEVITK	1351,65	138	149	0,00E+00
tr|O62825|O62825_CAS2_BUBBU	TVDMESTEVIT	1223,56	138	148	0,00E+00
tr|O62825|O62825_CAS2_BUBBU	VDMESTEVITKKTKLTEEDKNRLN	2820,45	139	162	0,00E+00
tr|O62825|O62825_CAS2_BUBBU	VDMESTEVITKKT	1479,75	139	151	0,00E+00

tr|E9NZN2|E9NZN2_CASA2_BUBBU	VDMESTEVFTKK	1412,69	139	150	0,00E+00
tr|O62825|O62825_CAS2_BUBBU	VDMESTEVITKK	1378,70	139	150	1,01E+06
tr|E9NZN2|E9NZN2_CASA2_BUBBU	VDMESTEVFTK	1284,59	139	149	0,00E+00
tr|O62825|O62825_CAS2_BUBBU	VDMESTEVITK	1250,61	139	149	1,20E+07
tr|O62825|O62825_CAS2_BUBBU	VDMESTEVIT	1122,51	139	148	1,71E+06
tr|O62825|O62825_CAS2_BUBBU	VDMESTE	809,31	139	145	3,04E+06
tr|O62825|O62825_CAS2_BUBBU	DMESTEVITKK	1279,63	140	150	0,00E+00
tr|O62825|O62825_CAS2_BUBBU	DMESTEVITK	1151,54	140	149	0,00E+00
tr|O62825|O62825_CAS2_BUBBU	MESTEVITKKT	1265,65	141	151	0,00E+00
tr|E9NZN2|E9NZN2_CASA2_BUBBU	MESTEVFTKK	1198,59	141	150	0,00E+00
tr|O62825|O62825_CAS2_BUBBU	MESTEVITKK	1164,61	141	150	0,00E+00
tr|E9NZN2|E9NZN2_CASA2_BUBBU	MESTEVFTK	1070,50	141	149	0,00E+00
tr|O62825|O62825_CAS2_BUBBU	MESTEVITK	1036,51	141	149	0,00E+00
tr|O62825|O62825_CAS2_BUBBU	MESTEVIT	908,42	141	148	0,00E+00
tr|E9NZN2|E9NZN2_CASA2_BUBBU	ESTEVFTKK	1067,55	142	150	0,00E+00
tr|O62825|O62825_CAS2_BUBBU	ESTEVITKK	1033,57	142	150	0,00E+00
tr|O62825|O62825_CAS2_BUBBU	ESTEVITK	905,47	142	149	0,00E+00
tr|O62825|O62825_CAS2_BUBBU	ESTEVIT	777,38	142	148	0,00E+00
tr|O62825|O62825_CAS2_BUBBU	STEVITKKTK	1133,67	143	152	0,00E+00
tr|O62825|O62825_CAS2_BUBBU	STEVITKKT	1005,57	143	151	2,75E+06
tr|E9NZN2|E9NZN2_CASA2_BUBBU	STEVFTKK	938,51	143	150	0,00E+00
tr|O62825|O62825_CAS2_BUBBU	STEVITKK	904,52	143	150	0,00E+00
tr|E9NZN2|E9NZN2_CASA2_BUBBU	STEVFTK	810,41	143	149	8,54E+05
tr|O62825|O62825_CAS2_BUBBU	STEVITK	776,43	143	149	0,00E+00
tr|E9NZN2|E9NZN2_CASA2_BUBBU	STEVF	581,27	143	147	1,43E+06
tr|O62825|O62825_CAS2_BUBBU	STEVI	547,29	143	147	0,00E+00
tr|E9NZN2|E9NZN2_CASA2_BUBBU	TEVFT	595,29	144	148	2,71E+06
tr|O62825|O62825_CAS2_BUBBU	VITKKTKLTEEDKNRLN	2029,15	146	162	2,22E+06
tr|O62825|O62825_CAS2_BUBBU	VITKKTKLTEE	1288,76	146	156	8,01E+06
tr|E9NZN2|E9NZN2_CASA2_BUBBU	VFTKKTKLTE	1193,70	146	155	1,03E+06
tr|O62825|O62825_CAS2_BUBBU	TKKTKLTEEDKNRLN	1817,00	148	162	1,06E+07
tr|O62825|O62825_CAS2_BUBBU	TKKTKLTEE	1076,61	148	156	8,29E+05
tr|O62825|O62825_CAS2_BUBBU	KTKLTEEDKNRLNFLK	1976,11	150	165	2,74E+07
tr|O62825|O62825_CAS2_BUBBU	KTKLTEEDKNRLNFL	1848,01	150	164	7,75E+06
tr|O62825|O62825_CAS2_BUBBU	KTKLTEEDKNRLNF	1734,93	150	163	4,04E+07
tr|O62825|O62825_CAS2_BUBBU	KTKLTEEDKNRLN	1587,86	150	162	8,58E+08
tr|O62825|O62825_CAS2_BUBBU	KTKLTEEDKNRL	1473,82	150	161	2,13E+06
tr|O62825|O62825_CAS2_BUBBU	KTKLTEEDKNR	1360,73	150	160	5,85E+05
tr|O62825|O62825_CAS2_BUBBU	KTKLTEEDKN	1204,63	150	159	2,64E+06
tr|O62825|O62825_CAS2_BUBBU	KTKLTEEDK	1090,59	150	158	2,06E+06
tr|O62825|O62825_CAS2_BUBBU	KTKLTEED	962,49	150	157	7,16E+07
tr|O62825|O62825_CAS2_BUBBU	TKLTEEDKNRLNFLKKISQHYQ	2732,46	151	172	4,13E+07
tr|O62825|O62825_CAS2_BUBBU	TKLTEEDKNRLNFLKKISQHY	2604,40	151	171	6,79E+07
tr|O62825|O62825_CAS2_BUBBU	TKLTEEDKNRLNFLKKISQH	2441,34	151	170	4,80E+07
tr|O62825|O62825_CAS2_BUBBU	TKLTEEDKNRLNFLKK	1976,11	151	166	2,32E+08
tr|O62825|O62825_CAS2_BUBBU	TKLTEEDKNRLNFLK	1848,01	151	165	4,48E+08
tr|O62825|O62825_CAS2_BUBBU	TKLTEEDKNRLNFL	1719,92	151	164	4,91E+08
tr|O62825|O62825_CAS2_BUBBU	TKLTEEDKNRLNF	1606,83	151	163	3,88E+08
tr|O62825|O62825_CAS2_BUBBU	TKLTEEDKNRLN	1459,76	151	162	3,28E+09
tr|O62825|O62825_CAS2_BUBBU	TKLTEEDKNRL	1345,72	151	161	1,82E+08
tr|O62825|O62825_CAS2_BUBBU	TKLTEEDKNR	1232,64	151	160	8,21E+07
tr|O62825|O62825_CAS2_BUBBU	TKLTEEDKN	1076,54	151	159	9,24E+07
tr|O62825|O62825_CAS2_BUBBU	KLTEEDKNRLNFLKK	1875,06	152	166	1,94E+07
tr|O62825|O62825_CAS2_BUBBU	KLTEEDKNRLNFLK	1746,96	152	165	5,74E+07
tr|O62825|O62825_CAS2_BUBBU	KLTEEDKNRLNFL	1618,87	152	164	7,80E+07
tr|O62825|O62825_CAS2_BUBBU	KLTEEDKNRLNF	1505,78	152	163	6,51E+07
tr|O62825|O62825_CAS2_BUBBU	KLTEEDKNRLN	1358,72	152	162	9,59E+08
tr|O62825|O62825_CAS2_BUBBU	KLTEEDKNRL	1244,67	152	161	5,91E+07
tr|O62825|O62825_CAS2_BUBBU	KLTEEDKNR	1131,59	152	160	4,15E+06
tr|O62825|O62825_CAS2_BUBBU	LTEEDKNRLNFLKKISQHYQK	2631,41	153	173	7,26E+06
tr|O62825|O62825_CAS2_BUBBU	LTEEDKNRLNFLKKISQHY	2375,26	153	171	3,07E+07
tr|O62825|O62825_CAS2_BUBBU	LTEEDKNRLNFLKKISQH	2212,20	153	170	9,96E+07
tr|O62825|O62825_CAS2_BUBBU	LTEEDKNRLNFLKK	1746,96	153	166	1,80E+08
tr|O62825|O62825_CAS2_BUBBU	LTEEDKNRLNFLK	1618,87	153	165	2,79E+08
tr|O62825|O62825_CAS2_BUBBU	LTEEDKNRLNFL	1490,77	153	164	1,87E+08
tr|O62825|O62825_CAS2_BUBBU	LTEEDKNRLNF	1377,69	153	163	2,41E+08
tr|O62825|O62825_CAS2_BUBBU	LTEEDKNRLN	1230,62	153	162	9,47E+08
tr|O62825|O62825_CAS2_BUBBU	LTEEDKNRL	1116,58	153	161	1,93E+07
tr|O62825|O62825_CAS2_BUBBU	TEEDKNRLNFLKKISQHYQ	2390,23	154	172	2,72E+07
tr|O62825|O62825_CAS2_BUBBU	TEEDKNRLNFLKKISQHY	2262,18	154	171	5,03E+07
tr|O62825|O62825_CAS2_BUBBU	TEEDKNRLNFLKKISQH	2099,11	154	170	3,26E+07
tr|O62825|O62825_CAS2_BUBBU	TEEDKNRLNFLKK	1633,88	154	166	2,29E+08
tr|O62825|O62825_CAS2_BUBBU	TEEDKNRLNFLK	1505,78	154	165	4,61E+08
tr|O62825|O62825_CAS2_BUBBU	TEEDKNRLNFL	1377,69	154	164	3,47E+08
tr|O62825|O62825_CAS2_BUBBU	TEEDKNRLNF	1264,60	154	163	6,26E+08
tr|O62825|O62825_CAS2_BUBBU	TEEDKNRLN	1117,54	154	162	5,06E+08
tr|O62825|O62825_CAS2_BUBBU	TEEDKNRL	1003,49	154	161	1,15E+07
tr|O62825|O62825_CAS2_BUBBU	EEDKNRLNFL	1276,64	155	164	1,82E+06
tr|O62825|O62825_CAS2_BUBBU	EEDKNRLNF	1163,56	155	163	3,56E+07
tr|O62825|O62825_CAS2_BUBBU	EEDKNRLN	1016,49	155	162	5,34E+06
tr|O62825|O62825_CAS2_BUBBU	EDKNRLNFLKK	1403,79	156	166	2,86E+06
tr|O62825|O62825_CAS2_BUBBU	EDKNRLNFLK	1275,69	156	165	3,92E+07
tr|O62825|O62825_CAS2_BUBBU	EDKNRLNFL	1147,60	156	164	3,13E+07
tr|O62825|O62825_CAS2_BUBBU	EDKNRLNF	1034,51	156	163	8,03E+07
tr|O62825|O62825_CAS2_BUBBU	DKNRLNFL	1018,56	157	164	2,05E+07
tr|O62825|O62825_CAS2_BUBBU	DKNRLNF	905,47	157	163	1,61E+08
tr|O62825|O62825_CAS2_BUBBU	DKNRLN	758,40	157	162	0,00E+00
tr|O62825|O62825_CAS2_BUBBU	KNRLNFL	903,53	158	164	2,39E+06
tr|O62825|O62825_CAS2_BUBBU	KNRLNF	790,44	158	163	1,68E+07
tr|O62825|O62825_CAS2_BUBBU	NRLNFL	775,43	159	164	3,84E+06
tr|O62825|O62825_CAS2_BUBBU	RLNFLK	789,49	160	165	4,95E+06
tr|O62825|O62825_CAS2_BUBBU	LNFLKK	761,48	161	166	8,23E+06
tr|O62825|O62825_CAS2_BUBBU	NFLKKISQHY	1276,69	162	171	2,27E+06
tr|O62825|O62825_CAS2_BUBBU	NFLKKISQH	1113,63	162	170	1,74E+06
tr|O62825|O62825_CAS2_BUBBU	NFLKKISQ	976,57	162	169	2,51E+06
tr|O62825|O62825_CAS2_BUBBU	FLKKISQHYQK	1418,80	163	173	7,41E+05
tr|O62825|O62825_CAS2_BUBBU	FLKKISQHYQ	1290,71	163	172	9,54E+07
tr|O62825|O62825_CAS2_BUBBU	FLKKISQHY	1162,65	163	171	1,36E+08

tr|O62825|O62825_CAS2_BUBBU	FLKKISQH	999,59	163	170	1,93E+08
tr|O62825|O62825_CAS2_BUBBU	FLKKISQ	862,53	163	169	2,52E+08
tr|O62825|O62825_CAS2_BUBBU	LKKISQHYQ	1143,64	164	172	4,52E+08
tr|O62825|O62825_CAS2_BUBBU	LKKISQHY	1015,58	164	171	1,57E+08
tr|O62825|O62825_CAS2_BUBBU	KKISQHYQ	1030,56	165	172	3,03E+07
tr|O62825|O62825_CAS2_BUBBU	KKISQHY	902,50	165	171	4,10E+07
tr|O62825|O62825_CAS2_BUBBU	KISQHYQK	1030,56	166	173	3,03E+07
tr|O62825|O62825_CAS2_BUBBU	KISQHYQ	902,46	166	172	4,43E+08
tr|O62825|O62825_CAS2_BUBBU	KISQHY	774,40	166	171	6,74E+08
tr|O62825|O62825_CAS2_BUBBU	ISQHYQK	902,46	167	173	3,95E+08
tr|O62825|O62825_CAS2_BUBBU	ISQHYQ	774,37	167	172	3,78E+09
tr|O62825|O62825_CAS2_BUBBU	ISQHY	646,31	167	171	2,84E+08
tr|O62825|O62825_CAS2_BUBBU	SQHYQ	661,28	168	172	6,65E+07
tr|O62825|O62825_CAS2_BUBBU	QHYQK	702,34	169	173	0,00E+00
tr|O62825|O62825_CAS2_BUBBU	QKFTWPQYLK	1337,71	172	181	0,00E+00
tr|E9NZN2|E9NZN2_CASA2_BUBBU	QKFAWPQY	1066,52	172	179	0,00E+00
tr|O62825|O62825_CAS2_BUBBU	QKFTWPQY	1096,53	172	179	0,00E+00
tr|E9NZN2|E9NZN2_CASA2_BUBBU	QKFAWPQ	903,46	172	178	6,93E+07
tr|O62825|O62825_CAS2_BUBBU	QKFTWPQ	933,47	172	178	2,79E+08
tr|O62825|O62825_CAS2_BUBBU	QKFTWP	805,41	172	177	3,63E+07
tr|E9NZN2|E9NZN2_CASA2_BUBBU	KFAWPQY	938,47	173	179	5,18E+06
tr|O62825|O62825_CAS2_BUBBU	KFTWPQY	968,48	173	179	8,52E+06
tr|O62825|O62825_CAS2_BUBBU	KFTWPQ	805,41	173	178	2,26E+08
tr|E9NZN2|E9NZN2_CASA2_BUBBU	KFAWPQ	775,40	173	178	6,42E+07
tr|O62825|O62825_CAS2_BUBBU	FTWPQYLKTV	1281,68	174	183	5,83E+06
tr|O62825|O62825_CAS2_BUBBU	FTWPQYLKT	1182,61	174	182	8,08E+07
tr|O62825|O62825_CAS2_BUBBU	FTWPQYLK	1081,56	174	181	5,67E+06
tr|O62825|O62825_CAS2_BUBBU	FTWPQY	840,38	174	179	2,49E+06
tr|O62825|O62825_CAS2_BUBBU	FTWPQ	677,32	174	178	1,67E+08
tr|E9NZN2|E9NZN2_CASA2_BUBBU	AWPQYLKTV	1104,60	175	183	1,65E+07
tr|O62825|O62825_CAS2_BUBBU	TWPQYLKTV	1134,61	175	183	3,97E+07
tr|E9NZN2|E9NZN2_CASA2_BUBBU	AWPQYLKT	1005,53	175	182	1,34E+08
tr|O62825|O62825_CAS2_BUBBU	TWPQYLKT	1035,54	175	182	3,26E+08
tr|O62825|O62825_CAS2_BUBBU	TWPQYLK	934,49	175	181	1,73E+08
tr|E9NZN2|E9NZN2_CASA2_BUBBU	AWPQYLK	904,48	175	181	7,10E+07
tr|E9NZN2|E9NZN2_CASA2_BUBBU	AWPQYL	776,39	175	180	3,40E+07
tr|O62825|O62825_CAS2_BUBBU	TWPQYL	806,40	175	180	1,65E+08
tr|O62825|O62825_CAS2_BUBBU	WPQYLKTV	1033,56	176	183	1,39E+07
tr|O62825|O62825_CAS2_BUBBU	WPQYLKT	934,49	176	182	1,36E+08
tr|O62825|O62825_CAS2_BUBBU	WPQYLK	833,44	176	181	3,75E+07
tr|O62825|O62825_CAS2_BUBBU	WPQYL	705,35	176	180	3,50E+07
tr|O62825|O62825_CAS2_BUBBU	PQYLKT	748,41	177	182	9,40E+06
tr|O62825|O62825_CAS2_BUBBU	PQYLK	647,36	177	181	3,82E+06
tr|O62825|O62825_CAS2_BUBBU	QYLKTVYQYQK	1460,77	178	188	1,08E+06
tr|O62825|O62825_CAS2_BUBBU	QYLKTVYQYQ	1332,67	178	187	1,20E+08
tr|O62825|O62825_CAS2_BUBBU	QYLKTV	750,43	178	183	6,38E+07
tr|O62825|O62825_CAS2_BUBBU	YLKTVYQYQK	1332,71	179	188	5,97E+06
tr|O62825|O62825_CAS2_BUBBU	YLKTVYQHQK	1306,70	179	188	5,41E+06
tr|O62825|O62825_CAS2_BUBBU	YLKTVYQ	913,49	179	185	9,42E+06
tr|O62825|O62825_CAS2_BUBBU	YLKTVY	785,43	179	184	4,19E+07
tr|O62825|O62825_CAS2_BUBBU	KTVYQYQK	1056,56	181	188	2,16E+08
tr|O62825|O62825_CAS2_BUBBU	KTVYQYQ	928,47	181	187	1,41E+08
tr|O62825|O62825_CAS2_BUBBU	KTVYQY	800,41	181	186	1,80E+08
tr|O62825|O62825_CAS2_BUBBU	KTVYQ	637,34	181	185	4,32E+07
tr|O62825|O62825_CAS2_BUBBU	TVYQYQKAMKPWTQPKT	2097,07	182	198	3,78E+06
tr|O62825|O62825_CAS2_BUBBU	TVYQYQKAMKPWTQP	1867,93	182	196	0,00E+00
tr|O62825|O62825_CAS2_BUBBU	TVYQYQKAM	1130,54	182	190	7,69E+07
tr|O62825|O62825_CAS2_BUBBU	TVYQYQKA	999,50	182	189	1,95E+08
tr|O62825|O62825_CAS2_BUBBU	TVYQYQK	928,47	182	188	7,15E+09
tr|O62825|O62825_CAS2_BUBBU	TVYQYQ	800,37	182	187	3,04E+08
tr|O62825|O62825_CAS2_BUBBU	VYQYQK	827,42	183	188	3,39E+09
tr|O62825|O62825_CAS2_BUBBU	YQYQKAMKPWTQPKTNVIPYVR	2738,44	184	205	3,59E+06
tr|O62825|O62825_CAS2_BUBBU	YQYQKAMKPWTQPKTNVIPY	2483,27	184	203	7,60E+06
tr|O62825|O62825_CAS2_BUBBU	YQYQKAMKPWTQPK	1795,91	184	197	2,76E+07
tr|O62825|O62825_CAS2_BUBBU	YQYQKA	799,39	184	189	1,71E+08
tr|O62825|O62825_CAS2_BUBBU	YQYQK	728,35	184	188	8,80E+07
tr|O62825|O62825_CAS2_BUBBU	QYQKAMKPWTQPKTNVIPYVR	2575,37	185	205	3,86E+06
tr|O62825|O62825_CAS2_BUBBU	QYQKAMKPWTQPK	1632,84	185	197	3,99E+07
tr|O62825|O62825_CAS2_BUBBU	QYQKAMKP	992,51	185	192	1,81E+07
tr|O62825|O62825_CAS2_BUBBU	YQKAMKPWTQPKTNVIP	2029,08	186	202	1,16E+07
tr|O62825|O62825_CAS2_BUBBU	YQKAMKPWTQPKTN	1719,88	186	199	3,28E+07
tr|O62825|O62825_CAS2_BUBBU	YQKAMKPWTQPKT	1605,83	186	198	3,70E+07
tr|O62825|O62825_CAS2_BUBBU	YQKAMKPWTQPK	1504,79	186	197	6,20E+07
tr|O62825|O62825_CAS2_BUBBU	YQKAMKPWTQP	1376,69	186	196	1,46E+06
tr|O62825|O62825_CAS2_BUBBU	YQKAMKP	864,45	186	192	9,46E+06
tr|O62825|O62825_CAS2_BUBBU	QKAMKPWTQPKTNVIPYVR	2284,25	187	205	0,00E+00
tr|O62825|O62825_CAS2_BUBBU	QKAMKPWTQPKTNVIPYV	2128,15	187	204	0,00E+00
tr|O62825|O62825_CAS2_BUBBU	QKAMKPWTQPKTNVIPY	2029,08	187	203	8,64E+06
tr|O62825|O62825_CAS2_BUBBU	QKAMKPWTQPKTN	1556,81	187	199	3,05E+06
tr|O62825|O62825_CAS2_BUBBU	QKAMKPWTQPKT	1442,77	187	198	7,86E+06
tr|O62825|O62825_CAS2_BUBBU	QKAMKPWTQPK	1341,72	187	197	6,28E+07
tr|O62825|O62825_CAS2_BUBBU	QKAMKPW	887,47	187	193	0,00E+00
tr|O62825|O62825_CAS2_BUBBU	KAMKPWTQPKT	1314,71	188	198	2,91E+06
tr|O62825|O62825_CAS2_BUBBU	KAMKPWTQPK	1213,66	188	197	4,57E+07
tr|O62825|O62825_CAS2_BUBBU	AMKPWTQPKTNVIPYVRY	2191,16	189	206	2,34E+07
tr|O62825|O62825_CAS2_BUBBU	AMKPWTQPKTNVIPYVR	2028,10	189	205	1,75E+08
tr|O62825|O62825_CAS2_BUBBU	AMKPWTQPKTNVIPYV	1872,00	189	204	4,55E+06
tr|O62825|O62825_CAS2_BUBBU	AMKPWTQPKTNVIPY	1772,93	189	203	2,27E+07
tr|O62825|O62825_CAS2_BUBBU	AMKPWTQPKTN	1300,66	189	199	3,55E+08
tr|O62825|O62825_CAS2_BUBBU	AMKPWTQPKT	1186,62	189	198	2,34E+07
tr|O62825|O62825_CAS2_BUBBU	AMKPWTQPK	1085,57	189	197	0,00E+00
tr|O62825|O62825_CAS2_BUBBU	MKPWTQPKTNVIPYVRY	2120,12	190	206	2,72E+07
tr|O62825|O62825_CAS2_BUBBU	MKPWTQPKTNVIPYVR	1957,06	190	205	7,90E+07
tr|O62825|O62825_CAS2_BUBBU	MKPWTQPKTNV	1328,69	190	200	1,74E+06
tr|O62825|O62825_CAS2_BUBBU	MKPWTQPKTN	1229,62	190	199	1,31E+08
tr|O62825|O62825_CAS2_BUBBU	MKPWTQPKT	1115,58	190	198	1,05E+08
tr|O62825|O62825_CAS2_BUBBU	MKPWTQPK	1014,53	190	197	1,26E+08
tr|O62825|O62825_CAS2_BUBBU	KPWTQPKTNVIPYVRY	1989,08	191	206	3,02E+06

tr|O62825|O62825_CAS2_BUBBU	KPWTQPKTNVIPYV	1669,92	191	204	9,69E+06
tr|O62825|O62825_CAS2_BUBBU	KPWTQPKTNVIPY	1570,85	191	203	9,47E+07
tr|O62825|O62825_CAS2_BUBBU	KPWTQPKTNVIP	1407,79	191	202	3,59E+07
tr|O62825|O62825_CAS2_BUBBU	KPWTQPKTNV	1197,65	191	200	7,94E+06
tr|O62825|O62825_CAS2_BUBBU	KPWTQPKTN	1098,58	191	199	7,38E+07
tr|O62825|O62825_CAS2_BUBBU	KPWTQPKT	984,54	191	198	4,02E+08
tr|O62825|O62825_CAS2_BUBBU	KPWTQPK	883,49	191	197	1,07E+09
tr|O62825|O62825_CAS2_BUBBU	KPWTQP	755,40	191	196	7,38E+07
tr|O62825|O62825_CAS2_BUBBU	PWTQPKTNVIPYVRY	1860,99	192	206	1,24E+07
tr|O62825|O62825_CAS2_BUBBU	PWTQPKTNVIPYVR	1697,93	192	205	3,13E+07
tr|O62825|O62825_CAS2_BUBBU	PWTQPKTNVIPYV	1541,82	192	204	1,07E+07
tr|O62825|O62825_CAS2_BUBBU	PWTQPKTNVIPY	1442,76	192	203	4,21E+07
tr|O62825|O62825_CAS2_BUBBU	PWTQPKTNVIP	1279,69	192	202	3,71E+07
tr|O62825|O62825_CAS2_BUBBU	PWTQPKTN	970,49	192	199	3,42E+07
tr|O62825|O62825_CAS2_BUBBU	PWTQPKT	856,44	192	198	1,12E+08
tr|O62825|O62825_CAS2_BUBBU	PWTQPK	755,40	192	197	1,00E+08
tr|O62825|O62825_CAS2_BUBBU	WTQPKTNVIPYVRY	1763,94	193	206	3,19E+08
tr|O62825|O62825_CAS2_BUBBU	WTQPKTNVIPYVR	1600,87	193	205	7,61E+08
tr|O62825|O62825_CAS2_BUBBU	WTQPKTNVIPYV	1444,77	193	204	3,47E+08
tr|O62825|O62825_CAS2_BUBBU	WTQPKTNVIPY	1345,70	193	203	7,48E+08
tr|O62825|O62825_CAS2_BUBBU	WTQPKTNVIP	1182,64	193	202	6,01E+08
tr|O62825|O62825_CAS2_BUBBU	WTQPKTNV	972,50	193	200	8,46E+07
tr|O62825|O62825_CAS2_BUBBU	WTQPKTN	873,43	193	199	2,82E+09
tr|O62825|O62825_CAS2_BUBBU	WTQPKT	759,39	193	198	1,80E+09
tr|O62825|O62825_CAS2_BUBBU	TQPKTNVIPYVRY	1577,86	194	206	1,89E+08
tr|O62825|O62825_CAS2_BUBBU	TQPKTNVIPYVR	1414,79	194	205	2,66E+08
tr|O62825|O62825_CAS2_BUBBU	TQPKTNVIPYV	1258,69	194	204	1,36E+08
tr|O62825|O62825_CAS2_BUBBU	TQPKTNVIPY	1159,62	194	203	3,85E+08
tr|O62825|O62825_CAS2_BUBBU	TQPKTNVIP	996,56	194	202	8,94E+07
tr|O62825|O62825_CAS2_BUBBU	TQPKTNVI	899,51	194	201	1,29E+07
tr|O62825|O62825_CAS2_BUBBU	TQPKTNV	786,42	194	200	1,25E+08
tr|O62825|O62825_CAS2_BUBBU	TQPKTN	687,36	194	199	4,55E+06
tr|O62825|O62825_CAS2_BUBBU	QPKTNVIPYVRY	1476,81	195	206	4,46E+07
tr|O62825|O62825_CAS2_BUBBU	QPKTNVIPYVR	1313,75	195	205	4,21E+07
tr|O62825|O62825_CAS2_BUBBU	QPKTNVIPY	1058,58	195	203	1,25E+08
tr|O62825|O62825_CAS2_BUBBU	QPKTNVIP	895,51	195	202	5,01E+07
tr|O62825|O62825_CAS2_BUBBU	KTNVIPYVRY	1251,70	197	206	1,37E+07
tr|O62825|O62825_CAS2_BUBBU	KTNVIPYVR	1088,63	197	205	3,48E+07
tr|O62825|O62825_CAS2_BUBBU	KTNVIPYV	932,53	197	204	4,88E+07
tr|O62825|O62825_CAS2_BUBBU	KTNVIPY	833,46	197	203	3,46E+08
tr|O62825|O62825_CAS2_BUBBU	KTKVIPY	847,52	197	203	1,27E+07
tr|O62825|O62825_CAS2_BUBBU	TKVIPYVRY	1137,65	198	206	2,02E+06
tr|O62825|O62825_CAS2_BUBBU	TNVIPYVRY	1123,60	198	206	1,66E+08
tr|O62825|O62825_CAS2_BUBBU	TKVIPYVR	974,59	198	205	9,09E+06
tr|O62825|O62825_CAS2_BUBBU	TNVIPYVR	960,54	198	205	1,04E+08
tr|O62825|O62825_CAS2_BUBBU	TNVIPYV	804,44	198	204	9,86E+06
tr|O62825|O62825_CAS2_BUBBU	TKVIPYV	818,49	198	204	3,00E+06
tr|O62825|O62825_CAS2_BUBBU	TNVIPY	705,37	198	203	7,91E+07
tr|O62825|O62825_CAS2_BUBBU	TNVIP	542,31	198	202	1,54E+08
tr|O62825|O62825_CAS2_BUBBU	NVIPYVRYL	1135,64	199	207	4,64E+06
tr|O62825|O62825_CAS2_BUBBU	NVIPYVRY	1022,55	199	206	2,65E+07
tr|O62825|O62825_CAS2_BUBBU	KVIPYVR	873,54	199	205	2,33E+06
tr|O62825|O62825_CAS2_BUBBU	NVIPYVR	859,49	199	205	2,21E+08
tr|O62825|O62825_CAS2_BUBBU	VIPYVRYL	1021,60	200	207	7,50E+06
tr|O62825|O62825_CAS2_BUBBU	VIPYVRY	908,51	200	206	1,13E+09
tr|O62825|O62825_CAS2_BUBBU	IPYVRYL	922,53	201	207	3,97E+06
tr|O62825|O62825_CAS2_BUBBU	IPYVRY	809,44	201	206	4,53E+08
tr|O62825|O62825_CAS2_BUBBU	IPYVR	646,38	201	205	3,18E+06
sp|P02666|CASB_BOVIN	SITRINK	830,50	22	28	7,68E+06
sp|P02666|CASB_BOVIN	IEKFQSEEQQQTE	1622,74	30	42	0,00E+00
sp|P02666|CASB_BOVIN	IEKFQSEEQQQTEDELQ	2107,95	30	46	1,68E+06
sp|P02666|CASB_BOVIN	IEKFQSEEQQQTEDELQDKIHPF	2845,34	30	52	0,00E+00
sp|P02666|CASB_BOVIN	IEKFQSEEQQQTEDELQDKIHPFAQTQ	3273,54	30	56	0,00E+00
sp|P02666|CASB_BOVIN	FQSEEQQQTE	1252,52	33	42	0,00E+00
sp|P02666|CASB_BOVIN	FQSEEQQQTEDELQ	1737,73	33	46	0,00E+00
sp|P02666|CASB_BOVIN	QSEEQQQTEDELQ	1590,66	34	46	7,69E+05
sp|P02666|CASB_BOVIN	SEEQQQTEDELQ	1462,61	35	46	1,40E+06
sp|P02666|CASB_BOVIN	TEDELQDKIHPF	1470,70	41	52	1,03E+07
sp|P02666|CASB_BOVIN	VPPFLQPEV	1024,56	84	92	9,96E+05
sp|P02666|CASB_BOVIN	LQPEVMGVSK	1086,57	88	97	3,83E+06
sp|P02666|CASB_BOVIN	EVMGVSK	748,38	91	97	1,53E+07
sp|P02666|CASB_BOVIN	EVMGVSKVK	975,54	91	99	1,01E+07
sp|P02666|CASB_BOVIN	VMGVS	491,24	92	96	1,88E+07
sp|P02666|CASB_BOVIN	VKGAMAPKHKEMPFPKYPVEPL	2493,33	98	119	0,00E+00
sp|P02666|CASB_BOVIN	HKEMPFPKYPVEPL	1710,88	106	119	2,32E+07
sp|P02666|CASB_BOVIN	MPFPKYPVEPL	1316,68	109	119	1,36E+07
sp|P02666|CASB_BOVIN	FPKYPVEPL	1088,59	111	119	9,28E+07
sp|P02666|CASB_BOVIN	YPVEPL	716,37	114	119	5,65E+06
sp|P02666|CASB_BOVIN	PVEPL	553,31	115	119	3,02E+07
sp|P02666|CASB_BOVIN_VAR_A1	DKIHPFAQTQSLVYPFPGPIH	2391,24	47	67	2,61E+07
sp|P02666|CASB_BOVIN_VAR_A1	DKIHPFAQTQSLVYPFPGPIHN	2505,28	47	68	1,86E+06
sp|P02666|CASB_BOVIN_VAR_A1	AQTQSLVYPFPGPIH	1653,85	52	67	7,54E+06
sp|P02666|CASB_BOVIN_VAR_A1	SLVYPFPGPIH	1225,65	57	67	7,89E+07
sp|P02666|CASB_BOVIN_VAR_A1	SLVYPFPGPIHN	1339,69	57	68	1,18E+07
sp|P02666|CASB_BOVIN_VAR_A1	SLVYPFPGPIHNSLPQ	1764,92	57	72	6,59E+06
sp|P02666|CASB_BOVIN_VAR_A1	LVYPFPGPIH	1138,62	58	67	4,49E+07
sp|P02666|CASB_BOVIN_VAR_A1	LVYPFPGPIHN	1252,66	58	68	5,92E+06
sp|P02666|CASB_BOVIN_VAR_A1	VYPFPGPIH	1025,53	59	67	6,00E+07
sp|P02666|CASB_BOVIN_VAR_A1	YPFPGPIH	926,47	60	67	2,17E+07
sp|P02666|CASB_BOVIN_VAR_A1	YPFPGPIHN	1040,51	60	68	0,00E+00
sp|P02666|CASB_BOVIN_VAR_A1	YPFPGPIHNSLPQ	1465,74	60	72	7,65E+06
sp|P02666|CASB_BOVIN_VAR_A1	FPGPIHN	780,39	62	68	8,71E+06
sp|P02666|CASB_BOVIN_VAR_A1	FPGPIHNSLPQ	1205,62	62	72	5,97E+06
sp|P02666|CASB_BOVIN_VAR_A1	GPIHNSLPQ	961,50	64	72	3,97E+07
sp|P02666|CASB_BOVIN;sp|P02666|CASB_BOVIN_VAR_A2	QTQSLVYPFPGPIP	1542,81	54	67	0,00E+00
sp|P02666|CASB_BOVIN;sp|P02666|CASB_BOVIN_VAR_A2	QSLVYPFPGPIP	1313,70	56	67	0,00E+00
sp|P02666|CASB_BOVIN;sp|P02666|CASB_BOVIN_VAR_A2	SLVYPFPGPIP	1185,64	57	67	2,04E+07
sp|P02666|CASB_BOVIN;sp|P02666|CASB_BOVIN_VAR_A2	LVYPFPGPIP	1098,61	58	67	3,83E+07

sp|P02666|CASB_BOVIN;sp|P02666|CASB_BOVIN_VAR_A2	VYPFPGPIP	985,53	59	67	1,55E+07
sp|P02666|CASB_BOVIN;sp|P02666|CASB_BOVIN_VAR_A2	YPFPGPIP	886,46	60	67	2,82E+07
sp|P02666|CASB_BOVIN;sp|P02666|CASB_BOVIN_VAR_A2	YPFPGPIPN	1000,50	60	68	0,00E+00
sp|P02666|CASB_BOVIN;sp|P02666|CASB_BOVIN_VAR_A2	YPFPGPIPNSLPQ	1425,73	60	72	1,16E+07
sp|P02666|CASB_BOVIN;sp|P02666|CASB_BOVIN_VAR_A2	FPGPIP	626,34	62	67	3,10E+07
sp|P02666|CASB_BOVIN;sp|P02666|CASB_BOVIN_VAR_A2	FPGPIPNSLPQ	1165,61	62	72	2,71E+06
sp|P02666|CASB_BOVIN;sp|P02666|CASB_BOVIN_VAR_A2	GPIPNSLPQ	921,49	64	72	0,00E+00
sp|Q9TSI0|CASB_BUBBU	RELEEL	787,41	1	6	5,86E+08
sp|Q9TSI0|CASB_BUBBU	RELEELNVPG	1154,59	1	10	1,18E+07
sp|Q9TSI0|CASB_BUBBU	RELEELNVPGE	1283,64	1	11	2,11E+06
sp|Q9TSI0|CASB_BUBBU	RELEELNVPGEIV	1495,79	1	13	0,00E+00
sp|Q9TSI0|CASB_BUBBU	RELEELNVPGEIVE	1624,83	1	14	5,14E+07
sp|Q9TSI0|CASB_BUBBU	ELEEL	631,31	2	6	3,64E+08
sp|Q9TSI0|CASB_BUBBU	ELEELNVPG	998,49	2	10	1,52E+07
sp|Q9TSI0|CASB_BUBBU	ELEELNVPGEIV	1339,69	2	13	1,04E+07
sp|Q9TSI0|CASB_BUBBU	ELEELNVPGEIVE	1468,73	2	14	2,03E+08
sp|Q9TSI0|CASB_BUBBU	LEELNVPG	869,45	3	10	2,45E+07
sp|Q9TSI0|CASB_BUBBU	LEELNVPGE	998,49	3	11	8,53E+06
sp|Q9TSI0|CASB_BUBBU	LEELNVPGEIVE	1339,69	3	14	6,26E+07
sp|Q9TSI0|CASB_BUBBU	LEELNVPGEIVESL	1539,80	3	16	4,50E+06
sp|Q9TSI0|CASB_BUBBU	EELNVPGE	885,41	4	11	1,93E+06
sp|Q9TSI0|CASB_BUBBU	EELNVPGEIVE	1226,60	4	14	1,01E+08
sp|Q9TSI0|CASB_BUBBU	EELNVPGEIVES	1313,64	4	15	1,95E+06
sp|Q9TSI0|CASB_BUBBU	ELNVP	570,30	5	9	2,29E+07
sp|Q9TSI0|CASB_BUBBU	ELNVPG	627,32	5	10	2,49E+07
sp|Q9TSI0|CASB_BUBBU	ELNVPGE	756,37	5	11	6,23E+05
sp|Q9TSI0|CASB_BUBBU	ELNVPGEIVE	1097,56	5	14	6,35E+07
sp|Q9TSI0|CASB_BUBBU	ELNVPGEIVESL	1297,68	5	16	8,74E+06
sp|Q9TSI0|CASB_BUBBU	LNVPGEIVE	968,52	6	14	1,73E+07
sp|Q9TSI0|CASB_BUBBU	NVPGEIVE	855,43	7	14	4,16E+07
sp|Q9TSI0|CASB_BUBBU	NVPGEIVESL	1055,55	7	16	3,74E+07
sp|Q9TSI0|CASB_BUBBU	VPGEIVE	741,39	8	14	2,42E+07
sp|Q9TSI0|CASB_BUBBU	VPGEIVESL	941,51	8	16	1,91E+06
sp|Q9TSI0|CASB_BUBBU	PGEIVE	642,32	9	14	1,02E+06
sp|Q9TSI0|CASB_BUBBU	GEIVE	545,27	10	14	4,94E+07
sp|Q9TSI0|CASB_BUBBU	GEIVES	632,30	10	15	2,74E+06
sp|Q9TSI0|CASB_BUBBU	GEIVESL	745,39	10	16	3,47E+07
sp|Q9TSI0|CASB_BUBBU	IVESL	559,32	12	16	1,58E+07
sp|Q9TSI0|CASB_BUBBU	IVESLSSSEESITH	1516,73	12	25	0,00E+00
sp|Q9TSI0|CASB_BUBBU	IVESLSSSEESITHIN	1743,85	12	27	0,00E+00
sp|Q9TSI0|CASB_BUBBU	IVESLSSSEESITHINK	1871,95	12	28	0,00E+00
sp|Q9TSI0|CASB_BUBBU	SLSSSEESITH	1175,53	15	25	0,00E+00
sp|Q9TSI0|CASB_BUBBU	SLSSSEESITHIN	1402,66	15	27	0,00E+00
sp|Q9TSI0|CASB_BUBBU	SLSSSEESITHINK	1530,75	15	28	0,00E+00
sp|Q9TSI0|CASB_BUBBU	SLSSSEESITHINKK	1658,85	15	29	0,00E+00
sp|Q9TSI0|CASB_BUBBU	LSSSEESITH	1088,50	16	25	0,00E+00
sp|Q9TSI0|CASB_BUBBU	LSSSEESITHIN	1315,63	16	27	0,00E+00
sp|Q9TSI0|CASB_BUBBU	LSSSEESITHINK	1443,72	16	28	0,00E+00
sp|Q9TSI0|CASB_BUBBU	SSSEESITH	975,41	17	25	2,32E+06
sp|Q9TSI0|CASB_BUBBU	SSSEESITHI	1088,50	17	26	0,00E+00
sp|Q9TSI0|CASB_BUBBU	SSSEESITHIN	1202,54	17	27	0,00E+00
sp|Q9TSI0|CASB_BUBBU	SSSEESITHINK	1330,64	17	28	0,00E+00
sp|Q9TSI0|CASB_BUBBU	SSEESITH	888,38	18	25	0,00E+00
sp|Q9TSI0|CASB_BUBBU	SSEESITHI	1001,47	18	26	0,00E+00
sp|Q9TSI0|CASB_BUBBU	SSEESITHIN	1115,51	18	27	0,00E+00
sp|Q9TSI0|CASB_BUBBU	SSEESITHINK	1243,60	18	28	0,00E+00
sp|Q9TSI0|CASB_BUBBU	SEESITHI	914,43	19	26	0,00E+00
sp|Q9TSI0|CASB_BUBBU	SEESITHIN	1028,48	19	27	2,01E+07
sp|Q9TSI0|CASB_BUBBU	EESITHIN	941,45	20	27	1,46E+07
sp|Q9TSI0|CASB_BUBBU	SITHINK	811,46	22	28	1,32E+07
sp|Q9TSI0|CASB_BUBBU	KKIEKFQSEEQQQME	1908,93	28	42	0,00E+00
sp|Q9TSI0|CASB_BUBBU	KKIEKFQSEEQQQMEDELQ	2394,14	28	46	0,00E+00
sp|Q9TSI0|CASB_BUBBU	KKIEKFQSEEQQQMEDELQDKIHPFAQTQ	3559,73	28	56	0,00E+00
sp|Q9TSI0|CASB_BUBBU	KIEKFQSE	1007,53	29	36	0,00E+00
sp|Q9TSI0|CASB_BUBBU	KIEKFQSEE	1136,57	29	37	0,00E+00
sp|Q9TSI0|CASB_BUBBU	KIEKFQSEEQQQ	1520,75	29	40	0,00E+00
sp|Q9TSI0|CASB_BUBBU	KIEKFQSEEQQQME	1780,83	29	42	0,00E+00
sp|Q9TSI0|CASB_BUBBU	KIEKFQSEEQQQMEDEL	2137,98	29	45	0,00E+00
sp|Q9TSI0|CASB_BUBBU	KIEKFQSEEQQQMEDELQ	2266,04	29	46	0,00E+00
sp|Q9TSI0|CASB_BUBBU	KIEKFQSEEQQQTEDELQ	2236,05	29	46	0,00E+00
sp|Q9TSI0|CASB_BUBBU	KIEKFQSEEQQQMEDELQDKIHPF	3003,43	29	52	0,00E+00
sp|Q9TSI0|CASB_BUBBU	KIEKFQSEEQQQMEDELQDKIHPFAQTQ	3431,63	29	56	8,11E+06
sp|Q9TSI0|CASB_BUBBU	KIEKFQSEEQQQMEDELQDKIHPFAQTQSL	3631,75	29	58	0,00E+00
sp|Q9TSI0|CASB_BUBBU	IEKFQSE	879,43	30	36	1,57E+08
sp|Q9TSI0|CASB_BUBBU	IEKFQSEE	1008,48	30	37	0,00E+00
sp|Q9TSI0|CASB_BUBBU	IEKFQSEEQ	1136,54	30	38	4,77E+07
sp|Q9TSI0|CASB_BUBBU	IEKFQSEEQQ	1264,59	30	39	6,79E+07
sp|Q9TSI0|CASB_BUBBU	IEKFQSEEQQQ	1392,65	30	40	0,00E+00
sp|Q9TSI0|CASB_BUBBU	IEKFQSEEQQQM	1523,69	30	41	0,00E+00
sp|Q9TSI0|CASB_BUBBU	IEKFQSEEQQQME	1652,74	30	42	2,34E+07
sp|Q9TSI0|CASB_BUBBU	IEKFQSEEQQQMED	1767,76	30	43	0,00E+00
sp|Q9TSI0|CASB_BUBBU	IEKFQSEEQQQMEDE	1896,80	30	44	0,00E+00
sp|Q9TSI0|CASB_BUBBU	IEKFQSEEQQQMEDEL	2009,89	30	45	0,00E+00
sp|Q9TSI0|CASB_BUBBU	IEKFQSEEQQQMEDELQ	2137,95	30	46	2,68E+07
sp|Q9TSI0|CASB_BUBBU	IEKFQSEEQQQMEDELQDK	2381,07	30	48	0,00E+00
sp|Q9TSI0|CASB_BUBBU	IEKFQSEEQQQMEDELQDKIH	2631,21	30	50	0,00E+00
sp|Q9TSI0|CASB_BUBBU	IEKFQSEEQQQMEDELQDKIHPF	2875,33	30	52	5,29E+06
sp|Q9TSI0|CASB_BUBBU	IEKFQSEEQQQMEDELQDKIHPFAQTQ	3303,54	30	56	6,13E+07
sp|Q9TSI0|CASB_BUBBU	IEKFQSEEQQQMEDELQDKIHPFAQTQS	3390,57	30	57	0,00E+00
sp|Q9TSI0|CASB_BUBBU	IEKFQSEEQQQMEDELQDKIHPFAQTQSL	3503,65	30	58	1,87E+06
sp|Q9TSI0|CASB_BUBBU	EKFQSEEQQ	1151,51	31	39	1,09E+08
sp|Q9TSI0|CASB_BUBBU	EKFQSEEQQQ	1279,57	31	40	3,03E+07
sp|Q9TSI0|CASB_BUBBU	EKFQSEEQQQM	1410,61	31	41	0,00E+00
sp|Q9TSI0|CASB_BUBBU	EKFQSEEQQQME	1539,65	31	42	1,49E+07
sp|Q9TSI0|CASB_BUBBU	EKFQSEEQQQMED	1654,68	31	43	0,00E+00
sp|Q9TSI0|CASB_BUBBU	EKFQSEEQQQMEDE	1783,72	31	44	0,00E+00
sp|Q9TSI0|CASB_BUBBU	EKFQSEEQQQMEDEL	1896,80	31	45	0,00E+00
sp|Q9TSI0|CASB_BUBBU	EKFQSEEQQQMEDELQ	2024,86	31	46	0,00E+00

sp|Q9TSI0|CASB_BUBBU	EKFQSEEQQQTEDELQ	1994,87	31	46	0,00E+00
sp|Q9TSI0|CASB_BUBBU	EKFQSEEQQQMEDELQDKIHPF	2762,25	31	52	0,00E+00
sp|Q9TSI0|CASB_BUBBU	EKFQSEEQQQTEDELQDKIHPF	2732,26	31	52	0,00E+00
sp|Q9TSI0|CASB_BUBBU	EKFQSEEQQQMEDELQDKIHPFAQ	2961,35	31	54	0,00E+00
sp|Q9TSI0|CASB_BUBBU	EKFQSEEQQQMEDELQDKIHPFAQT	3062,39	31	55	0,00E+00
sp|Q9TSI0|CASB_BUBBU	EKFQSEEQQQMEDELQDKIHPFAQTQ	3190,45	31	56	1,26E+06
sp|Q9TSI0|CASB_BUBBU	EKFQSEEQQQMEDELQDKIHPFAQTQSL	3390,57	31	58	0,00E+00
sp|Q9TSI0|CASB_BUBBU	KFQSE	637,31	32	36	2,84E+06
sp|Q9TSI0|CASB_BUBBU	KFQSEEQ	894,41	32	38	0,00E+00
sp|Q9TSI0|CASB_BUBBU	KFQSEEQQ	1022,47	32	39	2,17E+07
sp|Q9TSI0|CASB_BUBBU	KFQSEEQQQ	1150,53	32	40	2,05E+07
sp|Q9TSI0|CASB_BUBBU	KFQSEEQQQM	1281,57	32	41	0,00E+00
sp|Q9TSI0|CASB_BUBBU	KFQSEEQQQME	1410,61	32	42	3,70E+06
sp|Q9TSI0|CASB_BUBBU	KFQSEEQQQMEDEL	1767,76	32	45	0,00E+00
sp|Q9TSI0|CASB_BUBBU	KFQSEEQQQMEDELQ	1895,82	32	46	5,65E+05
sp|Q9TSI0|CASB_BUBBU	KFQSEEQQQTEDELQ	1865,83	32	46	0,00E+00
sp|Q9TSI0|CASB_BUBBU	KFQSEEQQQMEDELQDKIHPF	2633,21	32	52	0,00E+00
sp|Q9TSI0|CASB_BUBBU	KFQSEEQQQMEDELQDKIHPFAQTQ	3061,41	32	56	0,00E+00
sp|Q9TSI0|CASB_BUBBU	KFQSEEQQQMEDELQDKIHPFAQTQSL	3261,52	32	58	0,00E+00
sp|Q9TSI0|CASB_BUBBU	FQSEEQQ	894,37	33	39	0,00E+00
sp|Q9TSI0|CASB_BUBBU	FQSEEQQQ	1022,43	33	40	0,00E+00
sp|Q9TSI0|CASB_BUBBU	FQSEEQQQM	1153,47	33	41	0,00E+00
sp|Q9TSI0|CASB_BUBBU	FQSEEQQQME	1282,51	33	42	0,00E+00
sp|Q9TSI0|CASB_BUBBU	FQSEEQQQMEDEL	1639,67	33	45	0,00E+00
sp|Q9TSI0|CASB_BUBBU	FQSEEQQQMEDELQ	1767,73	33	46	0,00E+00
sp|Q9TSI0|CASB_BUBBU	FQSEEQQQMEDELQDKIHPF	2505,11	33	52	0,00E+00
sp|Q9TSI0|CASB_BUBBU	FQSEEQQQMEDELQDKIHPFAQTQ	2933,31	33	56	0,00E+00
sp|Q9TSI0|CASB_BUBBU	QSEEQQ	747,30	34	39	0,00E+00
sp|Q9TSI0|CASB_BUBBU	QSEEQQQM	1006,40	34	41	0,00E+00
sp|Q9TSI0|CASB_BUBBU	QSEEQQQME	1135,45	34	42	0,00E+00
sp|Q9TSI0|CASB_BUBBU	QSEEQQQMEDEL	1492,60	34	45	0,00E+00
sp|Q9TSI0|CASB_BUBBU	QSEEQQQMEDELQ	1620,66	34	46	7,02E+06
sp|Q9TSI0|CASB_BUBBU	SEEQQQMEDEL	1364,54	35	45	5,32E+06
sp|Q9TSI0|CASB_BUBBU	SEEQQQMEDELQ	1492,60	35	46	1,74E+07
sp|Q9TSI0|CASB_BUBBU	SEEQQQMEDELQDKIHPF	2229,98	35	52	6,51E+06
sp|Q9TSI0|CASB_BUBBU	EEQQQMEDEL	1277,51	36	45	2,83E+06
sp|Q9TSI0|CASB_BUBBU	EEQQQMEDELQ	1405,57	36	46	1,11E+07
sp|Q9TSI0|CASB_BUBBU	EQQQMEDELQ	1276,52	37	46	1,06E+07
sp|Q9TSI0|CASB_BUBBU	QQMED	649,24	39	43	0,00E+00
sp|Q9TSI0|CASB_BUBBU	QQMEDEL	891,36	39	45	0,00E+00
sp|Q9TSI0|CASB_BUBBU	QQMEDELQ	1019,42	39	46	0,00E+00
sp|Q9TSI0|CASB_BUBBU	QQMEDELQDKIHPFAQTQ	2185,01	39	56	0,00E+00
sp|Q9TSI0|CASB_BUBBU	QQMEDELQDKIHPFAQTQSL	2385,13	39	58	0,00E+00
sp|Q9TSI0|CASB_BUBBU	QMEDELQ	891,36	40	46	2,12E+06
sp|Q9TSI0|CASB_BUBBU	QMEDELQDKIHPF	1628,75	40	52	0,00E+00
sp|Q9TSI0|CASB_BUBBU	QMEDELQDKIHPFAQTQ	2056,95	40	56	6,93E+06
sp|Q9TSI0|CASB_BUBBU	MEDELQ	763,31	41	46	4,67E+06
sp|Q9TSI0|CASB_BUBBU	MEDELQDKIH	1256,57	41	50	3,77E+06
sp|Q9TSI0|CASB_BUBBU	MEDELQDKIHPF	1500,69	41	52	1,38E+08
sp|Q9TSI0|CASB_BUBBU	MEDELQDKIHPFAQTQ	1928,89	41	56	7,56E+07
sp|Q9TSI0|CASB_BUBBU	MEDELQDKIHPFAQTQSL	2129,01	41	58	1,53E+07
sp|Q9TSI0|CASB_BUBBU	EDELQ	632,27	42	46	6,89E+06
sp|Q9TSI0|CASB_BUBBU	EDELQDKIH	1125,53	42	50	4,13E+06
sp|Q9TSI0|CASB_BUBBU	EDELQDKIHPF	1369,65	42	52	1,45E+08
sp|Q9TSI0|CASB_BUBBU	EDELQDKIHPFAQTQ	1797,85	42	56	3,73E+07
sp|Q9TSI0|CASB_BUBBU	EDELQDKIHPFAQTQSL	1997,97	42	58	1,20E+07
sp|Q9TSI0|CASB_BUBBU	DELQDKIH	996,49	43	50	5,39E+07
sp|Q9TSI0|CASB_BUBBU	DELQDKIHP	1093,54	43	51	0,00E+00
sp|Q9TSI0|CASB_BUBBU	DELQDKIHPF	1240,61	43	52	1,41E+09
sp|Q9TSI0|CASB_BUBBU	DELQDKIHPFA	1311,65	43	53	2,78E+07
sp|Q9TSI0|CASB_BUBBU	DELQDKIHPFAQ	1439,70	43	54	1,22E+08
sp|Q9TSI0|CASB_BUBBU	DELQDKIHPFAQT	1540,75	43	55	1,13E+08
sp|Q9TSI0|CASB_BUBBU	DELQDKIHPFAQTQ	1668,81	43	56	2,34E+09
sp|Q9TSI0|CASB_BUBBU	DELQDKIHPFAQTQS	1755,84	43	57	2,10E+08
sp|Q9TSI0|CASB_BUBBU	DELQDKIHPFAQTQSL	1868,93	43	58	1,60E+09
sp|Q9TSI0|CASB_BUBBU	DELQDKIHPFAQTQSLVYPFPGPIPK	2964,54	43	68	5,83E+07
sp|Q9TSI0|CASB_BUBBU	DELQDKIHPFAQTQSLVYPFPGPIPKSL	3164,65	43	70	1,93E+07
sp|Q9TSI0|CASB_BUBBU	DELQDKIHPFAQTQSLVYPFPGPIPKSLPQ	3389,77	43	72	6,02E+07
sp|Q9TSI0|CASB_BUBBU	DELQDKIHPFAQTQSLVYPFPGPIPKSLPQNIPPLTQTPV	4450,36	43	82	2,27E+07
sp|Q9TSI0|CASB_BUBBU	ELQDKIHPF	1125,58	44	52	3,19E+06
sp|Q9TSI0|CASB_BUBBU	ELQDKIHPFAQTQ	1553,78	44	56	1,77E+08
sp|Q9TSI0|CASB_BUBBU	ELQDKIHPFAQTQS	1640,82	44	57	4,88E+06
sp|Q9TSI0|CASB_BUBBU	ELQDKIHPFAQTQSL	1753,90	44	58	3,23E+07
sp|Q9TSI0|CASB_BUBBU	LQDKIHPF	996,54	45	52	1,01E+07
sp|Q9TSI0|CASB_BUBBU	LQDKIHPFAQ	1195,64	45	54	8,04E+05
sp|Q9TSI0|CASB_BUBBU	LQDKIHPFAQT	1296,68	45	55	4,37E+06
sp|Q9TSI0|CASB_BUBBU	LQDKIHPFAQTQ	1424,74	45	56	1,63E+07
sp|Q9TSI0|CASB_BUBBU	LQDKIHPFAQTQSL	1624,86	45	58	1,22E+07
sp|Q9TSI0|CASB_BUBBU	QDKIHPF	883,46	46	52	2,41E+08
sp|Q9TSI0|CASB_BUBBU	QDKIHPFAQ	1082,55	46	54	5,11E+06
sp|Q9TSI0|CASB_BUBBU	QDKIHPFAQTQ	1311,66	46	56	3,09E+08
sp|Q9TSI0|CASB_BUBBU	QDKIHPFAQTQSL	1511,77	46	58	5,29E+07
sp|Q9TSI0|CASB_BUBBU	DKIHPF	755,40	47	52	5,79E+09
sp|Q9TSI0|CASB_BUBBU	DKIHPFA	826,43	47	53	1,90E+08
sp|Q9TSI0|CASB_BUBBU	DKIHPFAQ	954,49	47	54	2,86E+08
sp|Q9TSI0|CASB_BUBBU	DKIHPFAQT	1055,54	47	55	5,36E+08
sp|Q9TSI0|CASB_BUBBU	DKIHPFAQTQ	1183,60	47	56	8,94E+09
sp|Q9TSI0|CASB_BUBBU	DKIHPFAQTQS	1270,63	47	57	4,19E+08
sp|Q9TSI0|CASB_BUBBU	DKIHPFAQTQSL	1383,71	47	58	2,50E+09
sp|Q9TSI0|CASB_BUBBU	DKIHPFAQTQSLVYP	1742,90	47	61	5,44E+06
sp|Q9TSI0|CASB_BUBBU	DKIHPFAQTQSLVYPFPGPIP	2351,23	47	67	3,12E+07
sp|Q9TSI0|CASB_BUBBU	DKIHPFAQTQSLVYPFPGPIPK	2479,33	47	68	1,55E+08
sp|Q9TSI0|CASB_BUBBU	DKIHPFAQTQSLVYPFPGPIPKSL	2679,44	47	70	1,72E+08
sp|Q9TSI0|CASB_BUBBU	DKIHPFAQTQSLVYPFPGPIPKSLP	2776,50	47	71	2,46E+07
sp|Q9TSI0|CASB_BUBBU	DKIHPFAQTQSLVYPFPGPIPKSLPQ	2904,55	47	72	1,87E+08
sp|Q9TSI0|CASB_BUBBU	DKIHPFAQTQSLVYPFPGPIPKSLPQNIPPL	3438,87	47	77	7,00E+07
sp|Q9TSI0|CASB_BUBBU	DKIHPFAQTQSLVYPFPGPIPKSLPQNIPPLTQTPV	3965,15	47	82	6,53E+07
sp|Q9TSI0|CASB_BUBBU	KIHPFAQTQ	1068,57	48	56	1,12E+08

sp|Q9TSI0|CASB_BUBBU	KIHPFAQTQSL	1268,69	48	58	1,28E+07
sp|Q9TSI0|CASB_BUBBU	IHPFAQTQ	940,48	49	56	9,86E+07
sp|Q9TSI0|CASB_BUBBU	IHPFAQTQSL	1140,59	49	58	2,74E+07
sp|Q9TSI0|CASB_BUBBU	HPFAQTQ	827,39	50	56	9,27E+07
sp|Q9TSI0|CASB_BUBBU	HPFAQTQSL	1027,51	50	58	1,27E+07
sp|Q9TSI0|CASB_BUBBU	PFAQT	562,28	51	55	5,99E+06
sp|Q9TSI0|CASB_BUBBU	PFAQTQ	690,33	51	56	1,07E+08
sp|Q9TSI0|CASB_BUBBU	FAQTQS	680,31	52	57	2,46E+06
sp|Q9TSI0|CASB_BUBBU	FAQTQSL	793,40	52	58	4,83E+06
sp|Q9TSI0|CASB_BUBBU	FAQTQSLVYPFPGPIPK	1889,01	52	68	1,25E+06
sp|Q9TSI0|CASB_BUBBU	AQTQSLVYPFPGPIPK	1741,94	53	68	2,86E+07
sp|Q9TSI0|CASB_BUBBU	AQTQSLVYPFPGPIPKSL	1942,06	53	70	1,39E+07
sp|Q9TSI0|CASB_BUBBU	AQTQSLVYPFPGPIPKSLPQ	2167,17	53	72	1,09E+08
sp|Q9TSI0|CASB_BUBBU	QTQSL	575,29	54	58	3,61E+07
sp|Q9TSI0|CASB_BUBBU	QTQSLVYPFPGPIPK	1670,90	54	68	2,38E+07
sp|Q9TSI0|CASB_BUBBU	QTQSLVYPFPGPIPKSL	1871,02	54	70	1,33E+07
sp|Q9TSI0|CASB_BUBBU	QTQSLVYPFPGPIPKSLPQ	2096,13	54	72	2,28E+07
sp|Q9TSI0|CASB_BUBBU	QTQSLVYPFPGPIPKSLPQNIPPLTQTPVVVPPFLQP	4034,23	54	90	5,92E+07
sp|Q9TSI0|CASB_BUBBU	QTQSLVYPFPGPIPKSLPQNIPPLTQTPVVVPPFLQPEIM	4407,40	54	93	5,00E+07
sp|Q9TSI0|CASB_BUBBU	TQSLVYPFPGPIPKSLPQ	1968,07	55	72	2,61E+07
sp|Q9TSI0|CASB_BUBBU	TQSLVYPFPGPIPKSLPQNIPPLTQTPVVVPPFLQP	3906,17	55	90	2,93E+07
sp|Q9TSI0|CASB_BUBBU	QSLVYPFPGPI	1216,65	56	66	0,00E+00
sp|Q9TSI0|CASB_BUBBU	QSLVYPFPGPIPK	1441,80	56	68	0,00E+00
sp|Q9TSI0|CASB_BUBBU	QSLVYPFPGPIPKSLPQ	1867,02	56	72	0,00E+00
sp|Q9TSI0|CASB_BUBBU	SLVYPFP	821,43	57	63	1,22E+06
sp|Q9TSI0|CASB_BUBBU	SLVYPFPGPIPK	1313,74	57	68	4,00E+08
sp|Q9TSI0|CASB_BUBBU	SLVYPFPGPIPKS	1400,77	57	69	6,82E+07
sp|Q9TSI0|CASB_BUBBU	SLVYPFPGPIPKSL	1513,85	57	70	4,05E+08
sp|Q9TSI0|CASB_BUBBU	SLVYPFPGPIPKSLP	1610,91	57	71	1,15E+08
sp|Q9TSI0|CASB_BUBBU	SLVYPFPGPIPKSLPQ	1738,97	57	72	1,30E+09
sp|Q9TSI0|CASB_BUBBU	SLVYPFPGPIPKSLPQN	1853,01	57	73	1,50E+06
sp|Q9TSI0|CASB_BUBBU	SLVYPFPGPIPKSLPQNIPPL	2273,28	57	77	2,31E+08
sp|Q9TSI0|CASB_BUBBU	SLVYPFPGPIPKSLPQNIPPLTQT	2603,44	57	80	1,31E+07
sp|Q9TSI0|CASB_BUBBU	SLVYPFPGPIPKSLPQNIPPLTQTP	2700,49	57	81	6,50E+07
sp|Q9TSI0|CASB_BUBBU	SLVYPFPGPIPKSLPQNIPPLTQTPV	2799,56	57	82	2,79E+08
sp|Q9TSI0|CASB_BUBBU	SLVYPFPGPIPKSLPQNIPPLTQTPVVVPPF	3338,87	57	87	1,21E+08
sp|Q9TSI0|CASB_BUBBU	SLVYPFPGPIPKSLPQNIPPLTQTPVVVPPFL	3451,95	57	88	2,47E+07
sp|Q9TSI0|CASB_BUBBU	SLVYPFPGPIPKSLPQNIPPLTQTPVVVPPFLQ	3580,01	57	89	4,25E+07
sp|Q9TSI0|CASB_BUBBU	SLVYPFPGPIPKSLPQNIPPLTQTPVVVPPFLQP	3677,06	57	90	2,00E+09
sp|Q9TSI0|CASB_BUBBU	SLVYPFPGPIPKSLPQNIPPLTQTPVVVPPFLQPE	3806,11	57	91	2,76E+08
sp|Q9TSI0|CASB_BUBBU	SLVYPFPGPIPKSLPQNIPPLTQTPVVVPPFLQPEI	3919,19	57	92	2,03E+08
sp|Q9TSI0|CASB_BUBBU	SLVYPFPGPIPKSLPQNIPPLTQTPVVVPPFLQPEIM	4050,23	57	93	2,01E+08
sp|Q9TSI0|CASB_BUBBU	SLVYPFPGPIPKSLPQNIPPLTQTPVVVPPFLQPEIMGV	4206,32	57	95	3,72E+07
sp|Q9TSI0|CASB_BUBBU	LVYPFP	734,40	58	63	4,76E+07
sp|Q9TSI0|CASB_BUBBU	LVYPFPGPIPK	1226,71	58	68	1,51E+08
sp|Q9TSI0|CASB_BUBBU	LVYPFPGPIPKS	1313,74	58	69	4,31E+07
sp|Q9TSI0|CASB_BUBBU	LVYPFPGPIPKSL	1426,82	58	70	1,72E+08
sp|Q9TSI0|CASB_BUBBU	LVYPFPGPIPKSLP	1523,88	58	71	5,60E+07
sp|Q9TSI0|CASB_BUBBU	LVYPFPGPIPKSLPQ	1651,93	58	72	8,82E+08
sp|Q9TSI0|CASB_BUBBU	LVYPFPGPIPKSLPQN	1765,98	58	73	1,40E+06
sp|Q9TSI0|CASB_BUBBU	LVYPFPGPIPKSLPQNIPPL	2186,25	58	77	1,57E+08
sp|Q9TSI0|CASB_BUBBU	LVYPFPGPIPKSLPQNIPPLTQTPV	2712,53	58	82	6,72E+07
sp|Q9TSI0|CASB_BUBBU	LVYPFPGPIPKSLPQNIPPLTQTPVVVPPFLQPE	3719,07	58	91	9,39E+07
sp|Q9TSI0|CASB_BUBBU	LVYPFPGPIPKSLPQNIPPLTQTPVVVPPFLQPEI	3832,16	58	92	4,94E+07
sp|Q9TSI0|CASB_BUBBU	LVYPFPGPIPKSLPQNIPPLTQTPVVVPPFLQPEIM	3963,20	58	93	4,43E+07
sp|Q9TSI0|CASB_BUBBU	LVYPFPGPIPKSLPQNIPPLTQTPVVVPPFLQPEIMGVS	4206,32	58	96	1,63E+07
sp|Q9TSI0|CASB_BUBBU	LVYPFPGPIPKSLPQNIPPLTQTPVVVPPFLQPEIMGVSK	4334,42	58	97	6,02E+08
sp|Q9TSI0|CASB_BUBBU	VYPFPGPIPK	1113,62	59	68	2,74E+08
sp|Q9TSI0|CASB_BUBBU	VYPFPGPIPKS	1200,65	59	69	8,22E+07
sp|Q9TSI0|CASB_BUBBU	VYPFPGPIPKSL	1313,74	59	70	1,03E+08
sp|Q9TSI0|CASB_BUBBU	VYPFPGPIPKSLP	1410,79	59	71	2,44E+07
sp|Q9TSI0|CASB_BUBBU	VYPFPGPIPKSLPQ	1538,85	59	72	6,68E+08
sp|Q9TSI0|CASB_BUBBU	VYPFPGPIPKSLPQNIPPL	2073,17	59	77	2,90E+08
sp|Q9TSI0|CASB_BUBBU	VYPFPGPIPKSLPQNIPPLTQTPVVVPPFLQPEI	3719,07	59	92	7,73E+07
sp|Q9TSI0|CASB_BUBBU	VYPFPGPIPKSLPQNIPPLTQTPVVVPPFLQPEIMGVSK	4221,33	59	97	1,55E+09
sp|Q9TSI0|CASB_BUBBU	YPFPGP	676,32	60	65	1,74E+06
sp|Q9TSI0|CASB_BUBBU	YPFPGPIPK	1014,55	60	68	2,28E+08
sp|Q9TSI0|CASB_BUBBU	YPFPGPIPKS	1101,59	60	69	1,81E+08
sp|Q9TSI0|CASB_BUBBU	YPFPGPIPKSL	1214,67	60	70	5,56E+07
sp|Q9TSI0|CASB_BUBBU	YPFPGPIPKSLP	1311,72	60	71	4,78E+07
sp|Q9TSI0|CASB_BUBBU	YPFPGPIPKSLPQ	1439,78	60	72	9,87E+08
sp|Q9TSI0|CASB_BUBBU	YPFPGPIPKSLPQN	1553,82	60	73	4,98E+06
sp|Q9TSI0|CASB_BUBBU	YPFPGPIPKSLPQNIPPL	1974,10	60	77	2,40E+08
sp|Q9TSI0|CASB_BUBBU	YPFPGPIPKSLPQNIPPLTQTPV	2500,37	60	82	1,34E+07
sp|Q9TSI0|CASB_BUBBU	YPFPGPIPKSLPQNIPPLTQTPVVVPPF	3039,68	60	87	1,92E+07
sp|Q9TSI0|CASB_BUBBU	YPFPGPIPKSLPQNIPPLTQTPVVVPPFLQ	3280,83	60	89	8,25E+06
sp|Q9TSI0|CASB_BUBBU	YPFPGPIPKSLPQNIPPLTQTPVVVPPFLQP	3377,88	60	90	4,17E+08
sp|Q9TSI0|CASB_BUBBU	YPFPGPIPKSLPQNIPPLTQTPVVVPPFLQPE	3506,92	60	91	5,21E+07
sp|Q9TSI0|CASB_BUBBU	YPFPGPIPKSLPQNIPPLTQTPVVVPPFLQPEI	3620,01	60	92	2,24E+07
sp|Q9TSI0|CASB_BUBBU	YPFPGPIPKSLPQNIPPLTQTPVVVPPFLQPEIM	3751,05	60	93	2,83E+07
sp|Q9TSI0|CASB_BUBBU	YPFPGPIPKSLPQNIPPLTQTPVVVPPFLQPEIMGVS	3994,17	60	96	1,26E+07
sp|Q9TSI0|CASB_BUBBU	YPFPGPIPKSLPQNIPPLTQTPVVVPPFLQPEIMGVSK	4122,26	60	97	1,79E+08
sp|Q9TSI0|CASB_BUBBU	PFPGP	513,26	61	65	2,81E+06
sp|Q9TSI0|CASB_BUBBU	PFPGPIPK	851,49	61	68	1,64E+06
sp|Q9TSI0|CASB_BUBBU	PFPGPIPKSLPQ	1276,72	61	72	1,41E+07
sp|Q9TSI0|CASB_BUBBU	FPGPIPK	754,44	62	68	5,89E+07
sp|Q9TSI0|CASB_BUBBU	FPGPIPKS	841,47	62	69	4,71E+07
sp|Q9TSI0|CASB_BUBBU	FPGPIPKSL	954,55	62	70	1,15E+07
sp|Q9TSI0|CASB_BUBBU	FPGPIPKSLP	1051,61	62	71	1,36E+07
sp|Q9TSI0|CASB_BUBBU	FPGPIPKSLPQ	1179,67	62	72	1,79E+08
sp|Q9TSI0|CASB_BUBBU	FPGPIPKSLPQN	1293,71	62	73	2,95E+06
sp|Q9TSI0|CASB_BUBBU	FPGPIPKSLPQNIPPL	1713,98	62	77	2,91E+08
sp|Q9TSI0|CASB_BUBBU	FPGPIPKSLPQNIPPLTQT	2044,14	62	80	1,22E+07
sp|Q9TSI0|CASB_BUBBU	FPGPIPKSLPQNIPPLTQTP	2141,19	62	81	2,24E+07
sp|Q9TSI0|CASB_BUBBU	FPGPIPKSLPQNIPPLTQTPV	2240,26	62	82	9,42E+07
sp|Q9TSI0|CASB_BUBBU	FPGPIPKSLPQNIPPLTQTPVVVPPF	2779,57	62	87	3,92E+07
sp|Q9TSI0|CASB_BUBBU	FPGPIPKSLPQNIPPLTQTPVVVPPFLQ	3020,71	62	89	1,15E+07
sp|Q9TSI0|CASB_BUBBU	FPGPIPKSLPQNIPPLTQTPVVVPPFLQP	3117,76	62	90	7,47E+08

sp|Q9TSI0|CASB_BUBBU	FPGPIPKSLPQNIPPLTQTPVVVPPFLQPE	3246,81	62	91	6,68E+07
sp|Q9TSI0|CASB_BUBBU	FPGPIPKSLPQNIPPLTQTPVVVPPFLQPEI	3359,89	62	92	6,37E+07
sp|Q9TSI0|CASB_BUBBU	FPGPIPKSLPQNIPPLTQTPVVVPPFLQPEIM	3490,93	62	93	6,97E+07
sp|Q9TSI0|CASB_BUBBU	FPGPIPKSLPQNIPPLTQTPVVVPPFLQPEIMG	3547,95	62	94	1,71E+07
sp|Q9TSI0|CASB_BUBBU	FPGPIPKSLPQNIPPLTQTPVVVPPFLQPEIMGV	3647,02	62	95	2,33E+07
sp|Q9TSI0|CASB_BUBBU	FPGPIPKSLPQNIPPLTQTPVVVPPFLQPEIMGVSK	3862,15	62	97	5,33E+08
sp|Q9TSI0|CASB_BUBBU	FPGPIPKSLPQNIPPLTQTPVVVPPFLQPEIMGVSKVK	4089,31	62	99	1,15E+08
sp|Q9TSI0|CASB_BUBBU	PGPIP	479,27	63	67	4,23E+07
sp|Q9TSI0|CASB_BUBBU	GPIPKSLP	807,49	64	71	1,01E+07
sp|Q9TSI0|CASB_BUBBU	GPIPKSLPQ	935,54	64	72	5,74E+08
sp|Q9TSI0|CASB_BUBBU	GPIPKSLPQN	1049,59	64	73	7,81E+06
sp|Q9TSI0|CASB_BUBBU	GPIPKSLPQNI	1162,67	64	74	3,20E+06
sp|Q9TSI0|CASB_BUBBU	GPIPKSLPQNIPP	1356,78	64	76	5,10E+06
sp|Q9TSI0|CASB_BUBBU	GPIPKSLPQNIPPL	1469,86	64	77	3,19E+08
sp|Q9TSI0|CASB_BUBBU	GPIPKSLPQNIPPLT	1570,91	64	78	1,99E+06
sp|Q9TSI0|CASB_BUBBU	GPIPKSLPQNIPPLTQT	1800,01	64	80	1,46E+07
sp|Q9TSI0|CASB_BUBBU	GPIPKSLPQNIPPLTQTP	1897,07	64	81	2,40E+07
sp|Q9TSI0|CASB_BUBBU	GPIPKSLPQNIPPLTQTPV	1996,14	64	82	6,15E+07
sp|Q9TSI0|CASB_BUBBU	GPIPKSLPQNIPPLTQTPVVVPPF	2535,45	64	87	9,29E+06
sp|Q9TSI0|CASB_BUBBU	GPIPKSLPQNIPPLTQTPVVVPPFLQ	2776,59	64	89	2,38E+07
sp|Q9TSI0|CASB_BUBBU	GPIPKSLPQNIPPLTQTPVVVPPFLQP	2873,64	64	90	8,13E+08
sp|Q9TSI0|CASB_BUBBU	GPIPKSLPQNIPPLTQTPVVVPPFLQPE	3002,68	64	91	1,01E+08
sp|Q9TSI0|CASB_BUBBU	GPIPKSLPQNIPPLTQTPVVVPPFLQPEIMG	3303,83	64	94	2,25E+07
sp|Q9TSI0|CASB_BUBBU	GPIPKSLPQNIPPLTQTPVVVPPFLQPEIMGVSK	3618,03	64	97	3,44E+08
sp|Q9TSI0|CASB_BUBBU	GPIPKSLPQNIPPLTQTPVVVPPFLQPEIMGVSKVK	3845,19	64	99	3,01E+07
sp|Q9TSI0|CASB_BUBBU	GPIPKSLPQNIPPLTQTPVVVPPFLQPEIMGVSKVKEA	4045,27	64	101	2,73E+07
sp|Q9TSI0|CASB_BUBBU	IPKSLPQ	781,47	66	72	1,15E+08
sp|Q9TSI0|CASB_BUBBU	IPKSLPQNIPPL	1315,79	66	77	2,02E+07
sp|Q9TSI0|CASB_BUBBU	IPKSLPQNIPPLTQTP	1742,99	66	81	3,06E+06
sp|Q9TSI0|CASB_BUBBU	IPKSLPQNIPPLTQTPV	1842,06	66	82	9,67E+05
sp|Q9TSI0|CASB_BUBBU	IPKSLPQNIPPLTQTPVVVPPFLQP	2719,57	66	90	2,56E+07
sp|Q9TSI0|CASB_BUBBU	PKSLPQ	668,39	67	72	3,26E+06
sp|Q9TSI0|CASB_BUBBU	KSLPQNIPPL	1105,65	68	77	1,08E+07
sp|Q9TSI0|CASB_BUBBU	KSLPQNIPPLTQTPVVVPPFLQPEI	2751,56	68	92	2,10E+07
sp|Q9TSI0|CASB_BUBBU	KSLPQNIPPLTQTPVVVPPFLQPEIM	2882,60	68	93	1,73E+07
sp|Q9TSI0|CASB_BUBBU	KSLPQNIPPLTQTPVVVPPFLQPEIMGVSK	3253,81	68	97	2,27E+07
sp|Q9TSI0|CASB_BUBBU	SLPQNIPPL	977,55	69	77	1,37E+07
sp|Q9TSI0|CASB_BUBBU	SLPQNIPPLTQT	1307,71	69	80	3,27E+06
sp|Q9TSI0|CASB_BUBBU	SLPQNIPPLTQTP	1404,76	69	81	4,54E+06
sp|Q9TSI0|CASB_BUBBU	SLPQNIPPLTQTPV	1503,83	69	82	3,67E+07
sp|Q9TSI0|CASB_BUBBU	SLPQNIPPLTQTPVVVPPFLQP	2381,34	69	90	6,32E+07
sp|Q9TSI0|CASB_BUBBU	SLPQNIPPLTQTPVVVPPFLQPEIM	2754,50	69	93	2,78E+06
sp|Q9TSI0|CASB_BUBBU	SLPQNIPPLTQTPVVVPPFLQPEIMGVSK	3125,72	69	97	3,97E+08
sp|Q9TSI0|CASB_BUBBU	SLPQNIPPLTQTPVVVPPFLQPEIMGVSKVK	3352,88	69	99	9,90E+07
sp|Q9TSI0|CASB_BUBBU	SLPQNIPPLTQTPVVVPPFLQPEIMGVSKVKEA	3552,96	69	101	1,13E+08
sp|Q9TSI0|CASB_BUBBU	LPQNIPPL	890,52	70	77	4,53E+07
sp|Q9TSI0|CASB_BUBBU	LPQNIPPLT	991,57	70	78	0,00E+00
sp|Q9TSI0|CASB_BUBBU	LPQNIPPLTQ	1119,63	70	79	8,15E+05
sp|Q9TSI0|CASB_BUBBU	LPQNIPPLTQT	1220,68	70	80	8,84E+06
sp|Q9TSI0|CASB_BUBBU	LPQNIPPLTQTP	1317,73	70	81	3,15E+06
sp|Q9TSI0|CASB_BUBBU	LPQNIPPLTQTPV	1416,80	70	82	1,48E+07
sp|Q9TSI0|CASB_BUBBU	LPQNIPPLTQTPVVVPPFLQP	2294,30	70	90	4,31E+07
sp|Q9TSI0|CASB_BUBBU	LPQNIPPLTQTPVVVPPFLQPEI	2536,43	70	92	2,93E+06
sp|Q9TSI0|CASB_BUBBU	LPQNIPPLTQTPVVVPPFLQPEIMGVS	2910,59	70	96	1,19E+07
sp|Q9TSI0|CASB_BUBBU	LPQNIPPLTQTPVVVPPFLQPEIMGVSK	3038,69	70	97	3,63E+07
sp|Q9TSI0|CASB_BUBBU	PQNIPPL	777,44	71	77	0,00E+00
sp|Q9TSI0|CASB_BUBBU	PQNIPPLTQTP	1204,65	71	81	0,00E+00
sp|Q9TSI0|CASB_BUBBU	PQNIPPLTQTPV	1303,71	71	82	0,00E+00
sp|Q9TSI0|CASB_BUBBU	PQNIPPLTQTPVVV	1501,85	71	84	5,32E+05
sp|Q9TSI0|CASB_BUBBU	PQNIPPLTQTPVVVPPFLQP	2181,22	71	90	1,50E+08
sp|Q9TSI0|CASB_BUBBU	PQNIPPLTQTPVVVPPFLQPE	2310,26	71	91	5,29E+06
sp|Q9TSI0|CASB_BUBBU	PQNIPPLTQTPVVVPPFLQPEI	2423,35	71	92	1,60E+07
sp|Q9TSI0|CASB_BUBBU	PQNIPPLTQTPVVVPPFLQPEIM	2554,39	71	93	2,21E+07
sp|Q9TSI0|CASB_BUBBU	PQNIPPLTQTPVVVPPFLQPEIMGV	2710,48	71	95	3,54E+06
sp|Q9TSI0|CASB_BUBBU	PQNIPPLTQTPVVVPPFLQPEIMGVS	2797,51	71	96	1,68E+07
sp|Q9TSI0|CASB_BUBBU	PQNIPPLTQTPVVVPPFLQPEIMGVSK	2925,60	71	97	7,48E+07
sp|Q9TSI0|CASB_BUBBU	QNIPP	567,30	72	76	3,72E+06
sp|Q9TSI0|CASB_BUBBU	QNIPPLTQTPV	1206,66	72	82	3,64E+06
sp|Q9TSI0|CASB_BUBBU	QNIPPLTQTPVVVPPFLQP	2084,17	72	90	3,97E+07
sp|Q9TSI0|CASB_BUBBU	QNIPPLTQTPVVVPPFLQPEIM	2457,33	72	93	2,14E+07
sp|Q9TSI0|CASB_BUBBU	QNIPPLTQTPVVVPPFLQPEIMGV	2613,42	72	95	2,75E+06
sp|Q9TSI0|CASB_BUBBU	QNIPPLTQTPVVVPPFLQPEIMGVS	2700,46	72	96	2,50E+07
sp|Q9TSI0|CASB_BUBBU	QNIPPLTQTPVVVPPFLQPEIMGVSK	2828,55	72	97	1,33E+08
sp|Q9TSI0|CASB_BUBBU	QNIPPLTQTPVVVPPFLQPEIMGVSKVK	3055,71	72	99	1,99E+07
sp|Q9TSI0|CASB_BUBBU	NIPPLTQTPV	1078,60	73	82	2,46E+07
sp|Q9TSI0|CASB_BUBBU	NIPPLTQTPVVVPPFLQPEIM	2329,28	73	93	4,77E+06
sp|Q9TSI0|CASB_BUBBU	NIPPLTQTPVVVPPFLQPEIMGVSK	2700,49	73	97	8,19E+07
sp|Q9TSI0|CASB_BUBBU	IPPLTQTP	865,49	74	81	1,52E+06
sp|Q9TSI0|CASB_BUBBU	IPPLTQTPV	964,56	74	82	7,16E+07
sp|Q9TSI0|CASB_BUBBU	IPPLTQTPVVVPPFLQP	1842,07	74	90	2,18E+07
sp|Q9TSI0|CASB_BUBBU	IPPLTQTPVVVPPFLQPEIMGVSK	2586,45	74	97	7,13E+06
sp|Q9TSI0|CASB_BUBBU	PPLTQTPVVVPPFL	1503,87	75	88	8,65E+06
sp|Q9TSI0|CASB_BUBBU	PPLTQTPVVVPPFLQPEVMGVSKV	2558,42	75	98	0,00E+00
sp|Q9TSI0|CASB_BUBBU	LTQTPVVVPPF	1196,68	77	87	2,56E+06
sp|Q9TSI0|CASB_BUBBU	LTQTPVVVPPFLQP	1534,88	77	90	7,68E+07
sp|Q9TSI0|CASB_BUBBU	LTQTPVVVPPFLQPEIM	1908,04	77	93	1,06E+07
sp|Q9TSI0|CASB_BUBBU	LTQTPVVVPPFLQPEIMGVS	2151,17	77	96	5,92E+06
sp|Q9TSI0|CASB_BUBBU	LTQTPVVVPPFLQPEIMGVSK	2279,26	77	97	4,13E+07
sp|Q9TSI0|CASB_BUBBU	LTQTPVVVPPFLQPEIMGVSKVK	2506,42	77	99	3,05E+07
sp|Q9TSI0|CASB_BUBBU	TQTPVVVPPFLQP	1421,79	78	90	5,20E+07
sp|Q9TSI0|CASB_BUBBU	TQTPVVVPPFLQPE	1550,83	78	91	3,84E+07
sp|Q9TSI0|CASB_BUBBU	TQTPVVVPPFLQPEIM	1794,96	78	93	4,06E+07
sp|Q9TSI0|CASB_BUBBU	TQTPVVVPPFLQPEIMGVSK	2166,18	78	97	9,36E+07
sp|Q9TSI0|CASB_BUBBU	QTPVVVPPF	982,55	79	87	1,29E+06
sp|Q9TSI0|CASB_BUBBU	QTPVVVPPFLQ	1223,69	79	89	0,00E+00
sp|Q9TSI0|CASB_BUBBU	QTPVVVPPFLQP	1320,74	79	90	2,35E+07
sp|Q9TSI0|CASB_BUBBU	QTPVVVPPFLQPE	1449,79	79	91	3,14E+07
sp|Q9TSI0|CASB_BUBBU	QTPVVVPPFLQPEI	1562,87	79	92	3,47E+07

sp|Q9TSI0|CASB_BUBBU	QTPVVVPPFLQPEIM	1693,91	79	93	2,63E+07
sp|Q9TSI0|CASB_BUBBU	TPVVVPP	707,42	80	86	2,19E+06
sp|Q9TSI0|CASB_BUBBU	TPVVVPPF	854,49	80	87	2,19E+06
sp|Q9TSI0|CASB_BUBBU	TPVVVPPFL	967,57	80	88	7,30E+06
sp|Q9TSI0|CASB_BUBBU	TPVVVPPFLQ	1095,63	80	89	1,45E+07
sp|Q9TSI0|CASB_BUBBU	TPVVVPPFLQP	1192,69	80	90	9,37E+07
sp|Q9TSI0|CASB_BUBBU	TPVVVPPFLQPE	1321,73	80	91	6,37E+07
sp|Q9TSI0|CASB_BUBBU	TPVVVPPFLQPEI	1434,81	80	92	6,29E+07
sp|Q9TSI0|CASB_BUBBU	TPVVVPPFLQPEIM	1565,85	80	93	1,28E+08
sp|Q9TSI0|CASB_BUBBU	TPVVVPPFLQPEIMGVS	1808,97	80	96	1,05E+07
sp|Q9TSI0|CASB_BUBBU	TPVVVPPFLQPEIMGVSK	1937,07	80	97	2,67E+07
sp|Q9TSI0|CASB_BUBBU	PVVVPP	606,37	81	86	3,31E+07
sp|Q9TSI0|CASB_BUBBU	PVVVPPF	753,44	81	87	1,40E+06
sp|Q9TSI0|CASB_BUBBU	PVVVPPFLQP	1091,64	81	90	6,49E+07
sp|Q9TSI0|CASB_BUBBU	PVVVPPFLQPE	1220,68	81	91	1,89E+07
sp|Q9TSI0|CASB_BUBBU	PVVVPPFLQPEI	1333,76	81	92	1,36E+07
sp|Q9TSI0|CASB_BUBBU	PVVVPPFLQPEIM	1464,81	81	93	4,58E+07
sp|Q9TSI0|CASB_BUBBU	PVVVPPFLQPEIMG	1521,83	81	94	0,00E+00
sp|Q9TSI0|CASB_BUBBU	PVVVPPFLQPEIMGVS	1707,93	81	96	3,53E+07
sp|Q9TSI0|CASB_BUBBU	PVVVPPFLQPEIMGVSK	1836,02	81	97	8,05E+07
sp|Q9TSI0|CASB_BUBBU	PVVVPPFLQPEIMGVSKVK	2063,19	81	99	2,75E+07
sp|Q9TSI0|CASB_BUBBU	VVVPP	509,32	82	86	6,36E+06
sp|Q9TSI0|CASB_BUBBU	VVVPPF	656,39	82	87	2,31E+06
sp|Q9TSI0|CASB_BUBBU	VVVPPFL	769,47	82	88	3,05E+06
sp|Q9TSI0|CASB_BUBBU	VVVPPFLQ	897,53	82	89	1,03E+07
sp|Q9TSI0|CASB_BUBBU	VVVPPFLQP	994,59	82	90	1,04E+08
sp|Q9TSI0|CASB_BUBBU	VVVPPFLQPE	1123,63	82	91	4,81E+07
sp|Q9TSI0|CASB_BUBBU	VVVPPFLQPEI	1236,71	82	92	6,00E+07
sp|Q9TSI0|CASB_BUBBU	VVVPPFLQPEIM	1367,75	82	93	8,92E+07
sp|Q9TSI0|CASB_BUBBU	VVVPPFLQPEIMG	1424,77	82	94	6,62E+06
sp|Q9TSI0|CASB_BUBBU	VVVPPFLQPEIMGV	1523,84	82	95	3,64E+06
sp|Q9TSI0|CASB_BUBBU	VVVPPFLQPEIMGVS	1610,87	82	96	3,47E+07
sp|Q9TSI0|CASB_BUBBU	VVVPPFLQPEIMGVSK	1738,97	82	97	2,05E+08
sp|Q9TSI0|CASB_BUBBU	VVVPPFLQPEIMGVSKVK	1966,13	82	99	2,44E+07
sp|Q9TSI0|CASB_BUBBU	VVVPPFLQPEIMGVSKVKEA	2166,21	82	101	9,37E+06
sp|Q9TSI0|CASB_BUBBU	VVPPFLQ	798,46	83	89	3,28E+06
sp|Q9TSI0|CASB_BUBBU	VVPPFLQP	895,52	83	90	4,98E+06
sp|Q9TSI0|CASB_BUBBU	VVPPFLQPEI	1137,64	83	92	1,92E+07
sp|Q9TSI0|CASB_BUBBU	VVPPFLQPEIMGVSK	1639,90	83	97	2,90E+07
sp|Q9TSI0|CASB_BUBBU	VPPFLQP	796,45	84	90	8,74E+06
sp|Q9TSI0|CASB_BUBBU	VPPFLQPE	925,49	84	91	1,35E+07
sp|Q9TSI0|CASB_BUBBU	VPPFLQPEI	1038,58	84	92	3,96E+07
sp|Q9TSI0|CASB_BUBBU	VPPFLQPEIM	1169,62	84	93	8,17E+07
sp|Q9TSI0|CASB_BUBBU	VPPFLQPEIMGV	1325,71	84	95	1,28E+07
sp|Q9TSI0|CASB_BUBBU	VPPFLQPEIMGVS	1412,74	84	96	7,69E+06
sp|Q9TSI0|CASB_BUBBU	VPPFLQPEIMGVSK	1540,83	84	97	1,85E+07
sp|Q9TSI0|CASB_BUBBU	PFLQPEIMGVSK	1344,71	86	97	2,94E+06
sp|Q9TSI0|CASB_BUBBU	FLQPE	632,32	87	91	3,60E+07
sp|Q9TSI0|CASB_BUBBU	FLQPEIM	876,44	87	93	1,35E+06
sp|Q9TSI0|CASB_BUBBU	FLQPEIMGVSK	1247,66	87	97	3,37E+07
sp|Q9TSI0|CASB_BUBBU	FLQPEIMGVSKVK	1474,82	87	99	1,41E+07
sp|Q9TSI0|CASB_BUBBU	FLQPEIMGVSKVKEA	1674,90	87	101	6,27E+06
sp|Q9TSI0|CASB_BUBBU	LQPEIM	729,37	88	93	2,20E+06
sp|Q9TSI0|CASB_BUBBU	LQPEIMGVSK	1100,59	88	97	5,57E+07
sp|Q9TSI0|CASB_BUBBU	QPEIM	616,29	89	93	2,03E+07
sp|Q9TSI0|CASB_BUBBU	QPEIMG	673,31	89	94	1,45E+06
sp|Q9TSI0|CASB_BUBBU	QPEIMGVSK	987,51	89	97	6,42E+07
sp|Q9TSI0|CASB_BUBBU	PEIMGVSK	859,45	90	97	2,64E+07
sp|Q9TSI0|CASB_BUBBU	EIMGVSK	762,39	91	97	2,90E+09
sp|Q9TSI0|CASB_BUBBU	EIMGVSKVK	989,56	91	99	4,69E+08
sp|Q9TSI0|CASB_BUBBU	EIMGVSKVKE	1118,60	91	100	2,09E+07
sp|Q9TSI0|CASB_BUBBU	EIMGVSKVKEA	1189,64	91	101	6,62E+06
sp|Q9TSI0|CASB_BUBBU	EIMGVSKVKEAMAPKH	1753,92	91	106	6,25E+06
sp|Q9TSI0|CASB_BUBBU	IMGVSK	633,35	92	97	2,71E+08
sp|Q9TSI0|CASB_BUBBU	IMGVSKVK	860,52	92	99	8,16E+07
sp|Q9TSI0|CASB_BUBBU	MGVSK	520,27	93	97	6,41E+07
sp|Q9TSI0|CASB_BUBBU	GVSKVKEA	816,47	94	101	1,38E+08
sp|Q9TSI0|CASB_BUBBU	GVSKVKEAMAPK	1243,70	94	105	5,57E+07
sp|Q9TSI0|CASB_BUBBU	GVSKVKEAMAPKH	1380,75	94	106	2,25E+06
sp|Q9TSI0|CASB_BUBBU	SKVKEAMAPK	1087,61	96	105	4,11E+06
sp|Q9TSI0|CASB_BUBBU	SKVKEAMAPKH	1224,66	96	106	1,37E+06
sp|Q9TSI0|CASB_BUBBU	KVKEAMAPK	1000,57	97	105	1,05E+07
sp|Q9TSI0|CASB_BUBBU	KVKEAMAPKH	1137,63	97	106	1,57E+07
sp|Q9TSI0|CASB_BUBBU	KVKEAMAPKHKEMPFPKYPVEPFTESQSL	3372,73	97	125	3,32E+06
sp|Q9TSI0|CASB_BUBBU	KVKEAMAPKHKEMPFPKYPVEPFTESQSLT	3473,77	97	126	4,68E+06
sp|Q9TSI0|CASB_BUBBU	KVKEAMAPKHKEMPFPKYPVEPFTESQSLTLT	3687,90	97	128	4,13E+07
sp|Q9TSI0|CASB_BUBBU	KVKEAMAPKHKEMPFPKYPVEPFTESQSLTLTDVE	4031,04	97	131	1,67E+07
sp|Q9TSI0|CASB_BUBBU	VKEAMAPKH	1009,54	98	106	1,74E+08
sp|Q9TSI0|CASB_BUBBU	VKEAMAPKHKE	1266,68	98	108	1,82E+06
sp|Q9TSI0|CASB_BUBBU	VKEAMAPKHKEM	1397,72	98	109	9,39E+06
sp|Q9TSI0|CASB_BUBBU	VKEAMAPKHKEMPFPKYPVEPF	2599,33	98	119	6,68E+07
sp|Q9TSI0|CASB_BUBBU	VKEAMAPKHKEMPFPKYPVEPFTE	2829,42	98	121	2,26E+07
sp|Q9TSI0|CASB_BUBBU	VKEAMAPKHKEMPFPKYPVEPFTES	2916,46	98	122	8,14E+07
sp|Q9TSI0|CASB_BUBBU	VKEAMAPKHKEMPFPKYPVEPFTESQ	3044,51	98	123	2,90E+08
sp|Q9TSI0|CASB_BUBBU	VKEAMAPKHKEMPFPKYPVEPFTESQS	3131,55	98	124	6,48E+07
sp|Q9TSI0|CASB_BUBBU	VKEAMAPKHKEMPFPKYPVEPFTESQSL	3244,63	98	125	1,34E+08
sp|Q9TSI0|CASB_BUBBU	VKEAMAPKHKEMPFPKYPVEPFTESQSLT	3345,68	98	126	9,05E+07
sp|Q9TSI0|CASB_BUBBU	VKEAMAPKHKEMPFPKYPVEPFTESQSLTL	3458,76	98	127	1,66E+08
sp|Q9TSI0|CASB_BUBBU	VKEAMAPKHKEMPFPKYPVEPFTESQSLTLT	3559,81	98	128	4,88E+08
sp|Q9TSI0|CASB_BUBBU	VKEAMAPKHKEMPFPKYPVEPFTESQSLTLTD	3674,84	98	129	8,38E+06
sp|Q9TSI0|CASB_BUBBU	VKEAMAPKHKEMPFPKYPVEPFTESQSLTLTDVE	3902,95	98	131	4,30E+08
sp|Q9TSI0|CASB_BUBBU	VKEAMAPKHKEMPFPKYPVEPFTESQSLTLTDVEN	4016,99	98	132	3,39E+07
sp|Q9TSI0|CASB_BUBBU	VKEAMAPKHKEMPFPKYPVEPFTESQSLTLTDVENL	4130,07	98	133	6,20E+07
sp|Q9TSI0|CASB_BUBBU	KEAMAPK	773,41	99	105	2,16E+07
sp|Q9TSI0|CASB_BUBBU	KEAMAPKH	910,47	99	106	4,38E+06
sp|Q9TSI0|CASB_BUBBU	KEAMAPKHKEMPFPKYPVEPFTESQ	2945,45	99	123	1,63E+07
sp|Q9TSI0|CASB_BUBBU	KEAMAPKHKEMPFPKYPVEPFTESQSLTLT	3460,74	99	128	1,19E+07
sp|Q9TSI0|CASB_BUBBU	EAMAPK	645,32	100	105	2,46E+06

sp|Q9TSI0|CASB_BUBBU	EAMAPKH	782,37	100	106	5,02E+07
sp|Q9TSI0|CASB_BUBBU	EAMAPKHKEMPFPKYPVE	2128,05	100	117	2,93E+06
sp|Q9TSI0|CASB_BUBBU	EAMAPKHKEMPFPKYPVEPF	2372,17	100	119	5,14E+07
sp|Q9TSI0|CASB_BUBBU	EAMAPKHKEMPFPKYPVEPFTE	2602,26	100	121	2,08E+07
sp|Q9TSI0|CASB_BUBBU	EAMAPKHKEMPFPKYPVEPFTES	2689,29	100	122	5,87E+07
sp|Q9TSI0|CASB_BUBBU	EAMAPKHKEMPFPKYPVEPFTESQ	2817,35	100	123	1,68E+08
sp|Q9TSI0|CASB_BUBBU	EAMAPKHKEMPFPKYPVEPFTESQS	2904,38	100	124	1,95E+07
sp|Q9TSI0|CASB_BUBBU	EAMAPKHKEMPFPKYPVEPFTESQSL	3017,47	100	125	3,04E+07
sp|Q9TSI0|CASB_BUBBU	EAMAPKHKEMPFPKYPVEPFTESQSLT	3118,51	100	126	4,17E+07
sp|Q9TSI0|CASB_BUBBU	EAMAPKHKEMPFPKYPVEPFTESQSLTL	3231,60	100	127	1,04E+08
sp|Q9TSI0|CASB_BUBBU	EAMAPKHKEMPFPKYPVEPFTESQSLTLT	3332,65	100	128	4,28E+08
sp|Q9TSI0|CASB_BUBBU	EAMAPKHKEMPFPKYPVEPFTESQSLTLTDVE	3675,78	100	131	1,24E+08
sp|Q9TSI0|CASB_BUBBU	EAMAPKHKEMPFPKYPVEPFTESQSLTLTDVEN	3789,83	100	132	1,74E+07
sp|Q9TSI0|CASB_BUBBU	EAMAPKHKEMPFPKYPVEPFTESQSLTLTDVENLHLPLPL	4573,33	100	139	6,71E+07
sp|Q9TSI0|CASB_BUBBU	AMAPKHKEMPFPKYPVEPF	2243,13	101	119	8,54E+07
sp|Q9TSI0|CASB_BUBBU	AMAPKHKEMPFPKYPVEPFTE	2473,22	101	121	1,94E+07
sp|Q9TSI0|CASB_BUBBU	AMAPKHKEMPFPKYPVEPFTES	2560,25	101	122	8,17E+07
sp|Q9TSI0|CASB_BUBBU	AMAPKHKEMPFPKYPVEPFTESQ	2688,31	101	123	2,86E+08
sp|Q9TSI0|CASB_BUBBU	AMAPKHKEMPFPKYPVEPFTESQS	2775,34	101	124	1,72E+07
sp|Q9TSI0|CASB_BUBBU	AMAPKHKEMPFPKYPVEPFTESQSL	2888,42	101	125	6,07E+07
sp|Q9TSI0|CASB_BUBBU	AMAPKHKEMPFPKYPVEPFTESQSLT	2989,47	101	126	5,90E+07
sp|Q9TSI0|CASB_BUBBU	AMAPKHKEMPFPKYPVEPFTESQSLTL	3102,56	101	127	4,93E+07
sp|Q9TSI0|CASB_BUBBU	AMAPKHKEMPFPKYPVEPFTESQSLTLT	3203,60	101	128	2,94E+08
sp|Q9TSI0|CASB_BUBBU	AMAPKHKEMPFPKYPVEPFTESQSLTLTD	3318,63	101	129	1,23E+07
sp|Q9TSI0|CASB_BUBBU	AMAPKHKEMPFPKYPVEPFTESQSLTLTDVE	3546,74	101	131	1,33E+08
sp|Q9TSI0|CASB_BUBBU	AMAPKHKEMPFPKYPVEPFTESQSLTLTDVEN	3660,78	101	132	3,64E+07
sp|Q9TSI0|CASB_BUBBU	AMAPKHKEMPFPKYPVEPFTESQSLTLTDVENLHLPLPL	4444,29	101	139	6,05E+07
sp|Q9TSI0|CASB_BUBBU	AMAPKHKEMPFPKYPVEPFTESQSLTLTDVENLHLPLPLL	4557,37	101	140	4,89E+07
sp|Q9TSI0|CASB_BUBBU	MAPKHKEMPFPKYPVE	1927,97	102	117	4,88E+06
sp|Q9TSI0|CASB_BUBBU	MAPKHKEMPFPKYPVEPF	2172,09	102	119	8,11E+07
sp|Q9TSI0|CASB_BUBBU	MAPKHKEMPFPKYPVEPFTE	2402,18	102	121	5,14E+06
sp|Q9TSI0|CASB_BUBBU	MAPKHKEMPFPKYPVEPFTES	2489,21	102	122	9,78E+07
sp|Q9TSI0|CASB_BUBBU	MAPKHKEMPFPKYPVEPFTESQ	2617,27	102	123	2,26E+08
sp|Q9TSI0|CASB_BUBBU	MAPKHKEMPFPKYPVEPFTESQS	2704,30	102	124	3,01E+07
sp|Q9TSI0|CASB_BUBBU	MAPKHKEMPFPKYPVEPFTESQSL	2817,39	102	125	9,22E+07
sp|Q9TSI0|CASB_BUBBU	MAPKHKEMPFPKYPVEPFTESQSLT	2918,43	102	126	5,38E+07
sp|Q9TSI0|CASB_BUBBU	MAPKHKEMPFPKYPVEPFTESQSLTL	3031,52	102	127	1,07E+08
sp|Q9TSI0|CASB_BUBBU	MAPKHKEMPFPKYPVEPFTESQSLTLT	3132,57	102	128	3,13E+08
sp|Q9TSI0|CASB_BUBBU	MAPKHKEMPFPKYPVEPFTESQSLTLTD	3247,59	102	129	7,27E+06
sp|Q9TSI0|CASB_BUBBU	MAPKHKEMPFPKYPVEPFTESQSLTLTDVE	3475,70	102	131	1,95E+08
sp|Q9TSI0|CASB_BUBBU	MAPKHKEMPFPKYPVEPFTESQSLTLTDVEN	3589,75	102	132	9,66E+07
sp|Q9TSI0|CASB_BUBBU	MAPKHKEMPFPKYPVEPFTESQSLTLTDVENLHLP	4050,03	102	136	2,07E+07
sp|Q9TSI0|CASB_BUBBU	MAPKHKEMPFPKYPVEPFTESQSLTLTDVENLHLPLP	4260,16	102	138	3,70E+07
sp|Q9TSI0|CASB_BUBBU	MAPKHKEMPFPKYPVEPFTESQSLTLTDVENLHLPLPL	4373,25	102	139	2,10E+08
sp|Q9TSI0|CASB_BUBBU	MAPKHKEMPFPKYPVEPFTESQSLTLTDVENLHLPLPLL	4486,33	102	140	9,98E+07
sp|Q9TSI0|CASB_BUBBU	APKHKEMP	936,49	103	110	1,72E+06
sp|Q9TSI0|CASB_BUBBU	APKHKEMPFPKYPVEPF	2041,05	103	119	4,81E+07
sp|Q9TSI0|CASB_BUBBU	APKHKEMPFPKYPVEPFTE	2271,14	103	121	8,58E+06
sp|Q9TSI0|CASB_BUBBU	APKHKEMPFPKYPVEPFTES	2358,17	103	122	3,09E+06
sp|Q9TSI0|CASB_BUBBU	APKHKEMPFPKYPVEPFTESQ	2486,23	103	123	1,10E+08
sp|Q9TSI0|CASB_BUBBU	APKHKEMPFPKYPVEPFTESQS	2573,26	103	124	1,63E+07
sp|Q9TSI0|CASB_BUBBU	APKHKEMPFPKYPVEPFTESQSL	2686,35	103	125	2,61E+07
sp|Q9TSI0|CASB_BUBBU	APKHKEMPFPKYPVEPFTESQSLT	2787,39	103	126	1,61E+07
sp|Q9TSI0|CASB_BUBBU	APKHKEMPFPKYPVEPFTESQSLTL	2900,48	103	127	1,73E+07
sp|Q9TSI0|CASB_BUBBU	APKHKEMPFPKYPVEPFTESQSLTLT	3001,53	103	128	3,59E+07
sp|Q9TSI0|CASB_BUBBU	APKHKEMPFPKYPVEPFTESQSLTLTDVE	3344,66	103	131	2,93E+07
sp|Q9TSI0|CASB_BUBBU	APKHKEMPFPKYPVEPFTESQSLTLTDVEN	3458,71	103	132	6,81E+06
sp|Q9TSI0|CASB_BUBBU	APKHKEMPFPKYPVEPFTESQSLTLTDVENLHLPLPLLQS	4570,38	103	142	2,27E+08
sp|Q9TSI0|CASB_BUBBU	KHKEMPFPKYPVEPF	1872,96	105	119	1,16E+07
sp|Q9TSI0|CASB_BUBBU	KHKEMPFPKYPVEPFTE	2103,05	105	121	1,38E+07
sp|Q9TSI0|CASB_BUBBU	KHKEMPFPKYPVEPFTES	2190,08	105	122	1,60E+07
sp|Q9TSI0|CASB_BUBBU	KHKEMPFPKYPVEPFTESQ	2318,14	105	123	1,98E+08
sp|Q9TSI0|CASB_BUBBU	KHKEMPFPKYPVEPFTESQS	2405,17	105	124	7,83E+07
sp|Q9TSI0|CASB_BUBBU	KHKEMPFPKYPVEPFTESQSL	2518,26	105	125	3,69E+07
sp|Q9TSI0|CASB_BUBBU	KHKEMPFPKYPVEPFTESQSLT	2619,30	105	126	2,82E+07
sp|Q9TSI0|CASB_BUBBU	KHKEMPFPKYPVEPFTESQSLTL	2732,39	105	127	8,19E+07
sp|Q9TSI0|CASB_BUBBU	KHKEMPFPKYPVEPFTESQSLTLT	2833,44	105	128	1,70E+08
sp|Q9TSI0|CASB_BUBBU	KHKEMPFPKYPVEPFTESQSLTLTD	2948,46	105	129	5,59E+06
sp|Q9TSI0|CASB_BUBBU	KHKEMPFPKYPVEPFTESQSLTLTDVE	3176,57	105	131	2,05E+08
sp|Q9TSI0|CASB_BUBBU	KHKEMPFPKYPVEPFTESQSLTLTDVEN	3290,62	105	132	7,61E+06
sp|Q9TSI0|CASB_BUBBU	KHKEMPFPKYPVEPFTESQSLTLTDVENLHLPLPLLQS	4402,29	105	142	3,11E+08
sp|Q9TSI0|CASB_BUBBU	KHKEMPFPKYPVEPFTESQSLTLTDVENLHLPLPLLQSW	4588,37	105	143	3,23E+07
sp|Q9TSI0|CASB_BUBBU	HKEMP	640,30	106	110	2,38E+06
sp|Q9TSI0|CASB_BUBBU	HKEMPF	787,37	106	111	1,48E+07
sp|Q9TSI0|CASB_BUBBU	HKEMPFPKYP	1272,63	106	115	3,80E+06
sp|Q9TSI0|CASB_BUBBU	HKEMPFPKYPVE	1500,74	106	117	1,33E+07
sp|Q9TSI0|CASB_BUBBU	HKEMPFPKYPVEP	1597,80	106	118	5,69E+07
sp|Q9TSI0|CASB_BUBBU	HKEMPFPKYPVEPF	1744,86	106	119	4,94E+08
sp|Q9TSI0|CASB_BUBBU	HKEMPFPKYPVEPFT	1845,91	106	120	6,97E+06
sp|Q9TSI0|CASB_BUBBU	HKEMPFPKYPVEPFTE	1974,96	106	121	5,37E+07
sp|Q9TSI0|CASB_BUBBU	HKEMPFPKYPVEPFTES	2061,99	106	122	1,49E+08
sp|Q9TSI0|CASB_BUBBU	HKEMPFPKYPVEPFTESQ	2190,05	106	123	4,04E+08
sp|Q9TSI0|CASB_BUBBU	HKEMPFPKYPVEPFTESQS	2277,08	106	124	6,38E+07
sp|Q9TSI0|CASB_BUBBU	HKEMPFPKYPVEPFTESQSL	2390,16	106	125	5,79E+07
sp|Q9TSI0|CASB_BUBBU	HKEMPFPKYPVEPFTESQSLT	2491,21	106	126	1,55E+07
sp|Q9TSI0|CASB_BUBBU	HKEMPFPKYPVEPFTESQSLTL	2604,29	106	127	4,49E+07
sp|Q9TSI0|CASB_BUBBU	HKEMPFPKYPVEPFTESQSLTLT	2705,34	106	128	2,03E+08
sp|Q9TSI0|CASB_BUBBU	HKEMPFPKYPVEPFTESQSLTLTD	2820,37	106	129	3,83E+06
sp|Q9TSI0|CASB_BUBBU	HKEMPFPKYPVEPFTESQSLTLTDVE	3048,48	106	131	1,21E+08
sp|Q9TSI0|CASB_BUBBU	HKEMPFPKYPVEPFTESQSLTLTDVEN	3162,52	106	132	1,47E+07
sp|Q9TSI0|CASB_BUBBU	HKEMPFPKYPVEPFTESQSLTLTDVENLHLPLPLLQS	4274,20	106	142	2,00E+08
sp|Q9TSI0|CASB_BUBBU	KEMPFP	747,36	107	112	1,81E+06
sp|Q9TSI0|CASB_BUBBU	KEMPFPK	875,46	107	113	1,11E+07
sp|Q9TSI0|CASB_BUBBU	KEMPFPKYPVE	1363,68	107	117	3,19E+06
sp|Q9TSI0|CASB_BUBBU	KEMPFPKYPVEP	1460,74	107	118	5,09E+06
sp|Q9TSI0|CASB_BUBBU	KEMPFPKYPVEPF	1607,81	107	119	8,29E+07
sp|Q9TSI0|CASB_BUBBU	KEMPFPKYPVEPFTE	1837,90	107	121	1,44E+06
sp|Q9TSI0|CASB_BUBBU	KEMPFPKYPVEPFTES	1924,93	107	122	9,54E+07

sp|Q9TSI0|CASB_BUBBU	KEMPFPKYPVEPFTESQ	2052,99	107	123	2,94E+08
sp|Q9TSI0|CASB_BUBBU	KEMPFPKYPVEPFTESQS	2140,02	107	124	4,90E+07
sp|Q9TSI0|CASB_BUBBU	KEMPFPKYPVEPFTESQSL	2253,10	107	125	1,13E+08
sp|Q9TSI0|CASB_BUBBU	KEMPFPKYPVEPFTESQSLT	2354,15	107	126	8,93E+07
sp|Q9TSI0|CASB_BUBBU	KEMPFPKYPVEPFTESQSLTL	2467,23	107	127	3,47E+07
sp|Q9TSI0|CASB_BUBBU	KEMPFPKYPVEPFTESQSLTLT	2568,28	107	128	1,63E+09
sp|Q9TSI0|CASB_BUBBU	KEMPFPKYPVEPFTESQSLTLTD	2683,31	107	129	3,78E+07
sp|Q9TSI0|CASB_BUBBU	KEMPFPKYPVEPFTESQSLTLTDVE	2911,42	107	131	4,47E+08
sp|Q9TSI0|CASB_BUBBU	KEMPFPKYPVEPFTESQSLTLTDVEN	3025,46	107	132	2,79E+07
sp|Q9TSI0|CASB_BUBBU	KEMPFPKYPVEPFTESQSLTLTDVENL	3138,55	107	133	1,98E+07
sp|Q9TSI0|CASB_BUBBU	KEMPFPKYPVEPFTESQSLTLTDVENLHLPLP	3695,88	107	138	5,01E+07
sp|Q9TSI0|CASB_BUBBU	KEMPFPKYPVEPFTESQSLTLTDVENLHLPLPLLQ	4050,11	107	141	1,47E+08
sp|Q9TSI0|CASB_BUBBU	KEMPFPKYPVEPFTESQSLTLTDVENLHLPLPLLQS	4137,14	107	142	5,55E+08
sp|Q9TSI0|CASB_BUBBU	KEMPFPKYPVEPFTESQSLTLTDVENLHLPLPLLQSW	4323,22	107	143	2,47E+07
sp|Q9TSI0|CASB_BUBBU	EMPFP	619,27	108	112	1,01E+08
sp|Q9TSI0|CASB_BUBBU	EMPFPKYP	1007,48	108	115	1,99E+06
sp|Q9TSI0|CASB_BUBBU	EMPFPKYPVE	1235,59	108	117	9,38E+06
sp|Q9TSI0|CASB_BUBBU	EMPFPKYPVEP	1332,64	108	118	3,90E+07
sp|Q9TSI0|CASB_BUBBU	EMPFPKYPVEPF	1479,71	108	119	3,81E+08
sp|Q9TSI0|CASB_BUBBU	EMPFPKYPVEPFT	1580,76	108	120	7,77E+06
sp|Q9TSI0|CASB_BUBBU	EMPFPKYPVEPFTE	1709,80	108	121	7,71E+07
sp|Q9TSI0|CASB_BUBBU	EMPFPKYPVEPFTES	1796,83	108	122	2,50E+08
sp|Q9TSI0|CASB_BUBBU	EMPFPKYPVEPFTESQ	1924,89	108	123	6,13E+08
sp|Q9TSI0|CASB_BUBBU	EMPFPKYPVEPFTESQS	2011,92	108	124	7,89E+07
sp|Q9TSI0|CASB_BUBBU	EMPFPKYPVEPFTESQSL	2125,01	108	125	1,36E+08
sp|Q9TSI0|CASB_BUBBU	EMPFPKYPVEPFTESQSLT	2226,06	108	126	1,13E+08
sp|Q9TSI0|CASB_BUBBU	EMPFPKYPVEPFTESQSLTL	2339,14	108	127	7,52E+07
sp|Q9TSI0|CASB_BUBBU	EMPFPKYPVEPFTESQSLTLT	2440,19	108	128	1,70E+08
sp|Q9TSI0|CASB_BUBBU	EMPFPKYPVEPFTESQSLTLTDVE	2783,33	108	131	1,79E+07
sp|Q9TSI0|CASB_BUBBU	EMPFPKYPVEPFTESQSLTLTDVENLHLPLPLL	3793,95	108	140	7,67E+06
sp|Q9TSI0|CASB_BUBBU	EMPFPKYPVEPFTESQSLTLTDVENLHLPLPLLQSW	4195,12	108	143	8,56E+06
sp|Q9TSI0|CASB_BUBBU	MPFPKY	781,38	109	114	3,15E+06
sp|Q9TSI0|CASB_BUBBU	MPFPKYP	878,44	109	115	7,24E+05
sp|Q9TSI0|CASB_BUBBU	MPFPKYPV	977,50	109	116	3,08E+06
sp|Q9TSI0|CASB_BUBBU	MPFPKYPVE	1106,55	109	117	7,17E+06
sp|Q9TSI0|CASB_BUBBU	MPFPKYPVEP	1203,60	109	118	2,06E+07
sp|Q9TSI0|CASB_BUBBU	MPFPKYPVEPF	1350,67	109	119	3,31E+08
sp|Q9TSI0|CASB_BUBBU	MPFPKYPVEPFT	1451,72	109	120	1,21E+07
sp|Q9TSI0|CASB_BUBBU	MPFPKYPVEPFTE	1580,76	109	121	7,43E+07
sp|Q9TSI0|CASB_BUBBU	MPFPKYPVEPFTES	1667,79	109	122	1,86E+08
sp|Q9TSI0|CASB_BUBBU	MPFPKYPVEPFTESQ	1795,85	109	123	3,54E+08
sp|Q9TSI0|CASB_BUBBU	MPFPKYPVEPFTESQS	1882,88	109	124	3,99E+07
sp|Q9TSI0|CASB_BUBBU	MPFPKYPVEPFTESQSL	1995,97	109	125	9,76E+07
sp|Q9TSI0|CASB_BUBBU	MPFPKYPVEPFTESQSLT	2097,01	109	126	2,05E+07
sp|Q9TSI0|CASB_BUBBU	MPFPKYPVEPFTESQSLTL	2210,10	109	127	8,92E+06
sp|Q9TSI0|CASB_BUBBU	MPFPKYPVEPFTESQSLTLT	2311,14	109	128	9,17E+07
sp|Q9TSI0|CASB_BUBBU	MPFPKYPVEPFTESQSLTLTDVENLHLPLPLLQS	3880,00	109	142	9,32E+06
sp|Q9TSI0|CASB_BUBBU	PFPKYPVEPF	1219,63	110	119	2,29E+07
sp|Q9TSI0|CASB_BUBBU	PFPKYPVEPFTE	1449,72	110	121	5,46E+06
sp|Q9TSI0|CASB_BUBBU	PFPKYPVEPFTESQ	1664,81	110	123	1,43E+07
sp|Q9TSI0|CASB_BUBBU	PFPKYPVEPFTESQS	1751,84	110	124	2,61E+06
sp|Q9TSI0|CASB_BUBBU	PFPKYPVEPFTESQSL	1864,92	110	125	1,58E+06
sp|Q9TSI0|CASB_BUBBU	PFPKYPVEPFTESQSLT	1965,97	110	126	5,85E+05
sp|Q9TSI0|CASB_BUBBU	PFPKYPVEPFTESQSLTLT	2180,10	110	128	9,91E+06
sp|Q9TSI0|CASB_BUBBU	FPKYPV	749,41	111	116	1,20E+07
sp|Q9TSI0|CASB_BUBBU	FPKYPVE	878,45	111	117	1,42E+07
sp|Q9TSI0|CASB_BUBBU	FPKYPVEP	975,51	111	118	1,01E+08
sp|Q9TSI0|CASB_BUBBU	FPKYPVEPF	1122,58	111	119	1,81E+09
sp|Q9TSI0|CASB_BUBBU	FPKYPVEPFTE	1352,67	111	121	7,78E+07
sp|Q9TSI0|CASB_BUBBU	FPKYPVEPFTES	1439,70	111	122	1,29E+08
sp|Q9TSI0|CASB_BUBBU	FPKYPVEPFTESQ	1567,76	111	123	3,58E+08
sp|Q9TSI0|CASB_BUBBU	FPKYPVEPFTESQS	1654,79	111	124	8,29E+07
sp|Q9TSI0|CASB_BUBBU	FPKYPVEPFTESQSL	1767,87	111	125	2,85E+07
sp|Q9TSI0|CASB_BUBBU	FPKYPVEPFTESQSLT	1868,92	111	126	1,69E+07
sp|Q9TSI0|CASB_BUBBU	FPKYPVEPFTESQSLTL	1982,00	111	127	3,65E+07
sp|Q9TSI0|CASB_BUBBU	FPKYPVEPFTESQSLTLT	2083,05	111	128	1,17E+08
sp|Q9TSI0|CASB_BUBBU	FPKYPVEPFTESQSLTLTDVE	2426,19	111	131	1,34E+07
sp|Q9TSI0|CASB_BUBBU	FPKYPVEPFTESQSLTLTDVENLHLPLPLLQS	3651,91	111	142	2,93E+07
sp|Q9TSI0|CASB_BUBBU	PKYPVEPF	975,51	112	119	6,11E+06
sp|Q9TSI0|CASB_BUBBU	PKYPVEPFTE	1205,60	112	121	2,05E+06
sp|Q9TSI0|CASB_BUBBU	PKYPVEPFTESQ	1420,69	112	123	1,07E+07
sp|Q9TSI0|CASB_BUBBU	PKYPVEPFTESQS	1507,72	112	124	6,30E+05
sp|Q9TSI0|CASB_BUBBU	KYPVEP	731,39	113	118	8,45E+05
sp|Q9TSI0|CASB_BUBBU	KYPVEPF	878,45	113	119	7,51E+07
sp|Q9TSI0|CASB_BUBBU	KYPVEPFTE	1108,54	113	121	5,47E+06
sp|Q9TSI0|CASB_BUBBU	KYPVEPFTES	1195,58	113	122	1,20E+07
sp|Q9TSI0|CASB_BUBBU	KYPVEPFTESQ	1323,63	113	123	3,34E+07
sp|Q9TSI0|CASB_BUBBU	KYPVEPFTESQS	1410,67	113	124	6,33E+06
sp|Q9TSI0|CASB_BUBBU	KYPVEPFTESQSL	1523,75	113	125	4,00E+06
sp|Q9TSI0|CASB_BUBBU	KYPVEPFTESQSLT	1624,80	113	126	2,52E+06
sp|Q9TSI0|CASB_BUBBU	KYPVEPFTESQSLTL	1737,88	113	127	4,28E+06
sp|Q9TSI0|CASB_BUBBU	KYPVEPFTESQSLTLT	1838,93	113	128	2,22E+07
sp|Q9TSI0|CASB_BUBBU	KYPVEPFTESQSLTLTDVE	2182,07	113	131	4,99E+06
sp|Q9TSI0|CASB_BUBBU	KYPVEPFTESQSLTLTDVENLHLPLPLLQS	3407,79	113	142	8,77E+07
sp|Q9TSI0|CASB_BUBBU	YPVEPF	750,36	114	119	3,68E+08
sp|Q9TSI0|CASB_BUBBU	YPVEPFTE	980,45	114	121	5,41E+06
sp|Q9TSI0|CASB_BUBBU	YPVEPFTES	1067,48	114	122	1,45E+07
sp|Q9TSI0|CASB_BUBBU	YPVEPFTESQ	1195,54	114	123	2,66E+08
sp|Q9TSI0|CASB_BUBBU	YPVEPFTESQS	1282,57	114	124	1,02E+08
sp|Q9TSI0|CASB_BUBBU	YPVEPFTESQSL	1395,66	114	125	2,20E+07
sp|Q9TSI0|CASB_BUBBU	YPVEPFTESQSLT	1496,70	114	126	1,16E+07
sp|Q9TSI0|CASB_BUBBU	YPVEPFTESQSLTL	1609,79	114	127	1,36E+07
sp|Q9TSI0|CASB_BUBBU	YPVEPFTESQSLTLT	1710,84	114	128	7,93E+07
sp|Q9TSI0|CASB_BUBBU	PVEPF	587,30	115	119	1,53E+06
sp|Q9TSI0|CASB_BUBBU	PVEPFTE	817,39	115	121	0,00E+00
sp|Q9TSI0|CASB_BUBBU	PVEPFTES	904,42	115	122	4,93E+06
sp|Q9TSI0|CASB_BUBBU	PVEPFTESQ	1032,48	115	123	7,84E+07
sp|Q9TSI0|CASB_BUBBU	PVEPFTESQS	1119,51	115	124	2,87E+07

sp|Q9TSI0|CASB_BUBBU	PVEPFTESQSL	1232,59	115	125	1,27E+07
sp|Q9TSI0|CASB_BUBBU	PVEPFTESQSLT	1333,64	115	126	2,28E+06
sp|Q9TSI0|CASB_BUBBU	PVEPFTESQSLTLT	1547,77	115	128	2,78E+07
sp|Q9TSI0|CASB_BUBBU	PVEPFTESQSLTLTD	1662,80	115	129	7,76E+06
sp|Q9TSI0|CASB_BUBBU	PVEPFTESQSLTLTDVE	1890,91	115	131	1,76E+07
sp|Q9TSI0|CASB_BUBBU	PVEPFTESQSLTLTDVEN	2004,95	115	132	1,51E+07
sp|Q9TSI0|CASB_BUBBU	PVEPFTESQSLTLTDVENLHLPLPLLQS	3116,63	115	142	2,08E+08
sp|Q9TSI0|CASB_BUBBU	VEPFTE	720,33	116	121	1,09E+07
sp|Q9TSI0|CASB_BUBBU	VEPFTES	807,37	116	122	8,05E+06
sp|Q9TSI0|CASB_BUBBU	VEPFTESQ	935,42	116	123	4,55E+07
sp|Q9TSI0|CASB_BUBBU	VEPFTESQS	1022,46	116	124	4,15E+07
sp|Q9TSI0|CASB_BUBBU	EPFTESQ	836,36	117	123	5,00E+07
sp|Q9TSI0|CASB_BUBBU	EPFTESQS	923,39	117	124	1,12E+07
sp|Q9TSI0|CASB_BUBBU	PFTESQS	794,34	118	124	2,71E+06
sp|Q9TSI0|CASB_BUBBU	PFTESQSLTLT	1222,61	118	128	2,75E+07
sp|Q9TSI0|CASB_BUBBU	PFTESQSLTLTD	1337,64	118	129	1,18E+07
sp|Q9TSI0|CASB_BUBBU	PFTESQSLTLTDVE	1565,75	118	131	3,02E+07
sp|Q9TSI0|CASB_BUBBU	PFTESQSLTLTDVEN	1679,79	118	132	3,67E+07
sp|Q9TSI0|CASB_BUBBU	PFTESQSLTLTDVENLH	1929,93	118	134	1,19E+06
sp|Q9TSI0|CASB_BUBBU	PFTESQSLTLTDVENLHLPLPLL	2576,37	118	140	1,22E+07
sp|Q9TSI0|CASB_BUBBU	PFTESQSLTLTDVENLHLPLPLLQ	2704,43	118	141	2,46E+07
sp|Q9TSI0|CASB_BUBBU	PFTESQSLTLTDVENLHLPLPLLQS	2791,46	118	142	1,59E+08
sp|Q9TSI0|CASB_BUBBU	FTESQSLTLT	1125,56	119	128	4,10E+06
sp|Q9TSI0|CASB_BUBBU	TESQSLTLT	978,49	120	128	2,11E+08
sp|Q9TSI0|CASB_BUBBU	TESQSLTLTD	1093,51	120	129	9,57E+06
sp|Q9TSI0|CASB_BUBBU	TESQSLTLTDVE	1321,62	120	131	7,18E+07
sp|Q9TSI0|CASB_BUBBU	TESQSLTLTDVENLHLPLPLL	2332,25	120	140	6,84E+06
sp|Q9TSI0|CASB_BUBBU	TESQSLTLTDVENLHLPLPLLQ	2460,31	120	141	1,26E+07
sp|Q9TSI0|CASB_BUBBU	TESQSLTLTDVENLHLPLPLLQS	2547,34	120	142	3,72E+07
sp|Q9TSI0|CASB_BUBBU	TESQSLTLTDVENLHLPLPLLQSW	2733,42	120	143	6,04E+07
sp|Q9TSI0|CASB_BUBBU	ESQSLTLT	877,44	121	128	3,67E+06
sp|Q9TSI0|CASB_BUBBU	ESQSLTLTDVE	1220,58	121	131	5,15E+06
sp|Q9TSI0|CASB_BUBBU	SQSLTL	647,35	122	127	7,68E+06
sp|Q9TSI0|CASB_BUBBU	SQSLTLTDVE	1091,53	122	131	1,85E+07
sp|Q9TSI0|CASB_BUBBU	SQSLTLTDVENLHLPLPLLQS	2317,25	122	142	5,01E+07
sp|Q9TSI0|CASB_BUBBU	SQSLTLTDVENLHLPLPLLQSW	2503,33	122	143	2,59E+07
sp|Q9TSI0|CASB_BUBBU	QSLTLT	661,36	123	128	0,00E+00
sp|Q9TSI0|CASB_BUBBU	QSLTLTD	776,39	123	129	4,34E+06
sp|Q9TSI0|CASB_BUBBU	QSLTLTDVE	1004,50	123	131	5,09E+07
sp|Q9TSI0|CASB_BUBBU	QSLTLTDVEN	1118,55	123	132	6,03E+06
sp|Q9TSI0|CASB_BUBBU	QSLTLTDVENL	1231,63	123	133	2,08E+07
sp|Q9TSI0|CASB_BUBBU	QSLTLTDVENLH	1368,69	123	134	3,30E+06
sp|Q9TSI0|CASB_BUBBU	QSLTLTDVENLHLP	1578,83	123	136	0,00E+00
sp|Q9TSI0|CASB_BUBBU	QSLTLTDVENLHLPLP	1788,96	123	138	4,05E+07
sp|Q9TSI0|CASB_BUBBU	QSLTLTDVENLHLPLPL	1902,05	123	139	2,13E+07
sp|Q9TSI0|CASB_BUBBU	QSLTLTDVENLHLPLPLLQ	2143,19	123	141	1,27E+08
sp|Q9TSI0|CASB_BUBBU	QSLTLTDVENLHLPLPLLQS	2230,22	123	142	0,00E+00
sp|Q9TSI0|CASB_BUBBU	QSLTLTDVENLHLPLPLLQSW	2416,30	123	143	9,62E+07
sp|Q9TSI0|CASB_BUBBU	SLTLTDVE	876,44	124	131	1,11E+08
sp|Q9TSI0|CASB_BUBBU	SLTLTDVEN	990,49	124	132	2,40E+07
sp|Q9TSI0|CASB_BUBBU	SLTLTDVENL	1103,57	124	133	9,41E+07
sp|Q9TSI0|CASB_BUBBU	SLTLTDVENLH	1240,63	124	134	2,53E+07
sp|Q9TSI0|CASB_BUBBU	SLTLTDVENLHLPLP	1660,90	124	138	6,78E+07
sp|Q9TSI0|CASB_BUBBU	SLTLTDVENLHLPLPL	1773,99	124	139	7,56E+07
sp|Q9TSI0|CASB_BUBBU	SLTLTDVENLHLPLPLL	1887,07	124	140	2,09E+08
sp|Q9TSI0|CASB_BUBBU	SLTLTDVENLHLPLPLLQ	2015,13	124	141	4,58E+08
sp|Q9TSI0|CASB_BUBBU	SLTLTDVENLHLPLPLLQS	2102,16	124	142	2,02E+09
sp|Q9TSI0|CASB_BUBBU	SLTLTDVENLHLPLPLLQSW	2288,24	124	143	3,70E+08
sp|Q9TSI0|CASB_BUBBU	SLTLTDVENLHLPLPLLQSWMHQPPQPLPPTVMFPPQSVL	4510,38	124	163	1,31E+07
sp|Q9TSI0|CASB_BUBBU	LTLTDVE	789,41	125	131	1,90E+07
sp|Q9TSI0|CASB_BUBBU	LTLTDVENL	1016,54	125	133	3,82E+07
sp|Q9TSI0|CASB_BUBBU	LTLTDVENLHLPLP	1573,87	125	138	2,47E+08
sp|Q9TSI0|CASB_BUBBU	LTLTDVENLHLPLPL	1686,96	125	139	1,77E+07
sp|Q9TSI0|CASB_BUBBU	LTLTDVENLHLPLPLL	1800,04	125	140	4,75E+07
sp|Q9TSI0|CASB_BUBBU	LTLTDVENLHLPLPLLQ	1928,10	125	141	8,22E+07
sp|Q9TSI0|CASB_BUBBU	LTLTDVENLHLPLPLLQSW	2201,21	125	143	1,51E+08
sp|Q9TSI0|CASB_BUBBU	LTLTDVENLHLPLPLLQSWMH	2469,31	125	145	8,15E+06
sp|Q9TSI0|CASB_BUBBU	TLTDVE	676,33	126	131	1,39E+09
sp|Q9TSI0|CASB_BUBBU	TLTDVENL	903,45	126	133	1,13E+08
sp|Q9TSI0|CASB_BUBBU	TLTDVENLH	1040,51	126	134	6,51E+07
sp|Q9TSI0|CASB_BUBBU	TLTDVENLHL	1153,60	126	135	1,05E+07
sp|Q9TSI0|CASB_BUBBU	TLTDVENLHLP	1250,65	126	136	1,15E+08
sp|Q9TSI0|CASB_BUBBU	TLTDVENLHLPL	1363,73	126	137	5,14E+07
sp|Q9TSI0|CASB_BUBBU	TLTDVENLHLPLP	1460,79	126	138	1,37E+08
sp|Q9TSI0|CASB_BUBBU	TLTDVENLHLPLPL	1573,87	126	139	2,47E+08
sp|Q9TSI0|CASB_BUBBU	TLTDVENLHLPLPLL	1686,96	126	140	4,35E+08
sp|Q9TSI0|CASB_BUBBU	TLTDVENLHLPLPLLQ	1815,01	126	141	7,98E+08
sp|Q9TSI0|CASB_BUBBU	TLTDVENLHLPLPLLQS	1902,05	126	142	1,75E+09
sp|Q9TSI0|CASB_BUBBU	TLTDVENLHLPLPLLQSW	2088,13	126	143	2,25E+09
sp|Q9TSI0|CASB_BUBBU	TLTDVENLHLPLPLLQSWMH	2356,22	126	145	3,94E+07
sp|Q9TSI0|CASB_BUBBU	TLTDVENLHLPLPLLQSWMHQPPQPLPPTVMFPPQSVLS	4397,30	126	164	2,31E+07
sp|Q9TSI0|CASB_BUBBU	LTDVENLH	939,47	127	134	4,14E+06
sp|Q9TSI0|CASB_BUBBU	LTDVENLHL	1052,55	127	135	8,32E+05
sp|Q9TSI0|CASB_BUBBU	LTDVENLHLP	1149,60	127	136	0,00E+00
sp|Q9TSI0|CASB_BUBBU	LTDVENLHLPL	1262,69	127	137	3,68E+06
sp|Q9TSI0|CASB_BUBBU	LTDVENLHLPLP	1359,74	127	138	2,48E+07
sp|Q9TSI0|CASB_BUBBU	LTDVENLHLPLPL	1472,82	127	139	3,24E+07
sp|Q9TSI0|CASB_BUBBU	LTDVENLHLPLPLL	1585,91	127	140	3,73E+07
sp|Q9TSI0|CASB_BUBBU	LTDVENLHLPLPLLQ	1713,97	127	141	9,13E+07
sp|Q9TSI0|CASB_BUBBU	LTDVENLHLPLPLLQS	1801,00	127	142	2,31E+08
sp|Q9TSI0|CASB_BUBBU	LTDVENLHLPLPLLQSW	1987,08	127	143	2,52E+08
sp|Q9TSI0|CASB_BUBBU	LTDVENLHLPLPLLQSWMH	2255,18	127	145	7,78E+06
sp|Q9TSI0|CASB_BUBBU	TDVENL	689,32	128	133	1,02E+08
sp|Q9TSI0|CASB_BUBBU	TDVENLH	826,38	128	134	8,88E+07
sp|Q9TSI0|CASB_BUBBU	TDVENLHL	939,47	128	135	2,05E+07
sp|Q9TSI0|CASB_BUBBU	TDVENLHLP	1036,52	128	136	8,69E+07
sp|Q9TSI0|CASB_BUBBU	TDVENLHLPL	1149,60	128	137	2,08E+07
sp|Q9TSI0|CASB_BUBBU	TDVENLHLPLP	1246,66	128	138	5,70E+07

sp|Q9TSI0|CASB_BUBBU	TDVENLHLPLPL	1359,74	128	139	1,65E+08
sp|Q9TSI0|CASB_BUBBU	TDVENLHLPLPLL	1472,82	128	140	1,64E+08
sp|Q9TSI0|CASB_BUBBU	TDVENLHLPLPLLQ	1600,88	128	141	3,92E+08
sp|Q9TSI0|CASB_BUBBU	TDVENLHLPLPLLQS	1687,91	128	142	4,55E+08
sp|Q9TSI0|CASB_BUBBU	TDVENLHLPLPLLQSW	1873,99	128	143	8,69E+08
sp|Q9TSI0|CASB_BUBBU	TDVENLHLPLPLLQSWMHQPPQPLPPTVMFPPQSVLS	4183,16	128	164	1,44E+07
sp|Q9TSI0|CASB_BUBBU	DVENLHL	838,42	129	135	9,73E+06
sp|Q9TSI0|CASB_BUBBU	DVENLHLP	935,47	129	136	1,35E+08
sp|Q9TSI0|CASB_BUBBU	DVENLHLPL	1048,56	129	137	4,13E+07
sp|Q9TSI0|CASB_BUBBU	DVENLHLPLP	1145,61	129	138	1,56E+08
sp|Q9TSI0|CASB_BUBBU	DVENLHLPLPL	1258,69	129	139	2,72E+08
sp|Q9TSI0|CASB_BUBBU	DVENLHLPLPLL	1371,78	129	140	8,62E+08
sp|Q9TSI0|CASB_BUBBU	DVENLHLPLPLLQ	1499,83	129	141	2,19E+09
sp|Q9TSI0|CASB_BUBBU	DVENLHLPLPLLQS	1586,87	129	142	5,74E+09
sp|Q9TSI0|CASB_BUBBU	DVENLHLPLPLLQSW	1772,95	129	143	6,90E+09
sp|Q9TSI0|CASB_BUBBU	DVENLHLPLPLLQSWMH	2041,05	129	145	1,41E+08
sp|Q9TSI0|CASB_BUBBU	DVENLHLPLPLLQSWMHQPPQPLPPT	2996,56	129	154	2,19E+07
sp|Q9TSI0|CASB_BUBBU	DVENLHLPLPLLQSWMHQPPQPLPPTVMFPPQS	3782,93	129	161	3,38E+07
sp|Q9TSI0|CASB_BUBBU	DVENLHLPLPLLQSWMHQPPQPLPPTVMFPPQSV	3882,00	129	162	1,60E+08
sp|Q9TSI0|CASB_BUBBU	DVENLHLPLPLLQSWMHQPPQPLPPTVMFPPQSVL	3995,08	129	163	1,00E+08
sp|Q9TSI0|CASB_BUBBU	DVENLHLPLPLLQSWMHQPPQPLPPTVMFPPQSVLS	4082,12	129	164	1,34E+08
sp|Q9TSI0|CASB_BUBBU	DVENLHLPLPLLQSWMHQPPQPLPPTVMFPPQSVLSL	4195,20	129	165	7,23E+07
sp|Q9TSI0|CASB_BUBBU	DVENLHLPLPLLQSWMHQPPQPLPPTVMFPPQSVLSLS	4282,23	129	166	1,45E+07
sp|Q9TSI0|CASB_BUBBU	DVENLHLPLPLLQSWMHQPPQPLPPTVMFPPQSVLSLSQ	4410,29	129	167	1,22E+07
sp|Q9TSI0|CASB_BUBBU	VENLHLP	820,44	130	136	4,73E+07
sp|Q9TSI0|CASB_BUBBU	VENLHLPL	933,53	130	137	2,24E+07
sp|Q9TSI0|CASB_BUBBU	VENLHLPLP	1030,58	130	138	4,72E+07
sp|Q9TSI0|CASB_BUBBU	VENLHLPLPL	1143,67	130	139	3,27E+08
sp|Q9TSI0|CASB_BUBBU	VENLHLPLPLL	1256,75	130	140	6,58E+08
sp|Q9TSI0|CASB_BUBBU	VENLHLPLPLLQ	1384,81	130	141	3,61E+08
sp|Q9TSI0|CASB_BUBBU	VENLHLPLPLLQS	1471,84	130	142	4,21E+08
sp|Q9TSI0|CASB_BUBBU	VENLHLPLPLLQSW	1657,92	130	143	4,09E+08
sp|Q9TSI0|CASB_BUBBU	VENLHLPLPLLQSWMH	1926,02	130	145	9,34E+06
sp|Q9TSI0|CASB_BUBBU	ENLHL	624,32	131	135	2,27E+06
sp|Q9TSI0|CASB_BUBBU	ENLHLPL	834,46	131	137	2,67E+06
sp|Q9TSI0|CASB_BUBBU	ENLHLPLP	931,51	131	138	3,47E+06
sp|Q9TSI0|CASB_BUBBU	ENLHLPLPL	1044,60	131	139	2,47E+07
sp|Q9TSI0|CASB_BUBBU	ENLHLPLPLL	1157,68	131	140	4,65E+07
sp|Q9TSI0|CASB_BUBBU	ENLHLPLPLLQ	1285,74	131	141	6,63E+07
sp|Q9TSI0|CASB_BUBBU	ENLHLPLPLLQS	1372,77	131	142	2,92E+07
sp|Q9TSI0|CASB_BUBBU	ENLHLPLPLLQSW	1558,85	131	143	5,75E+07
sp|Q9TSI0|CASB_BUBBU	NLHLPLP	802,47	132	138	4,13E+07
sp|Q9TSI0|CASB_BUBBU	NLHLPLPL	915,55	132	139	7,76E+07
sp|Q9TSI0|CASB_BUBBU	NLHLPLPLL	1028,64	132	140	2,30E+08
sp|Q9TSI0|CASB_BUBBU	NLHLPLPLLQ	1156,70	132	141	5,51E+08
sp|Q9TSI0|CASB_BUBBU	NLHLPLPLLQS	1243,73	132	142	2,31E+09
sp|Q9TSI0|CASB_BUBBU	NLHLPLPLLQSW	1429,81	132	143	8,06E+08
sp|Q9TSI0|CASB_BUBBU	NLHLPLPLLQSWMH	1697,91	132	145	5,18E+06
sp|Q9TSI0|CASB_BUBBU	NLHLPLPLLQSWMHQPPQPLPPTVMFPPQSVL	3651,95	132	163	3,44E+07
sp|Q9TSI0|CASB_BUBBU	LHLPLPL	801,51	133	139	1,50E+08
sp|Q9TSI0|CASB_BUBBU	LHLPLPLL	914,60	133	140	5,64E+07
sp|Q9TSI0|CASB_BUBBU	LHLPLPLLQ	1042,65	133	141	4,68E+08
sp|Q9TSI0|CASB_BUBBU	LHLPLPLLQS	1129,69	133	142	8,28E+08
sp|Q9TSI0|CASB_BUBBU	LHLPLPLLQSW	1315,77	133	143	1,28E+08
sp|Q9TSI0|CASB_BUBBU	HLPLPLL	801,51	134	140	1,74E+08
sp|Q9TSI0|CASB_BUBBU	HLPLPLLQ	929,57	134	141	3,50E+08
sp|Q9TSI0|CASB_BUBBU	HLPLPLLQS	1016,60	134	142	7,15E+08
sp|Q9TSI0|CASB_BUBBU	HLPLPLLQSW	1202,68	134	143	4,10E+08
sp|Q9TSI0|CASB_BUBBU	LPLPL	551,37	135	139	4,03E+07
sp|Q9TSI0|CASB_BUBBU	LPLPLLQ	792,51	135	141	5,82E+07
sp|Q9TSI0|CASB_BUBBU	LPLPLLQS	879,54	135	142	2,25E+08
sp|Q9TSI0|CASB_BUBBU	LPLPLLQSW	1065,62	135	143	2,51E+08
sp|Q9TSI0|CASB_BUBBU	LPLPLLQSWM	1196,66	135	144	7,26E+05
sp|Q9TSI0|CASB_BUBBU	LPLPLLQSWMHQPPQPLPPTVMFPPQSVL	3287,76	135	163	1,41E+07
sp|Q9TSI0|CASB_BUBBU	LPLLQ	582,37	137	141	2,74E+06
sp|Q9TSI0|CASB_BUBBU	LPLLQSW	855,49	137	143	2,42E+08
sp|Q9TSI0|CASB_BUBBU	LPLLQSWMH	1123,58	137	145	5,34E+06
sp|Q9TSI0|CASB_BUBBU	LPLLQSWMHQPP	1445,75	137	148	2,62E+06
sp|Q9TSI0|CASB_BUBBU	LPLLQSWMHQPPQPLPPT	2079,10	137	154	1,61E+07
sp|Q9TSI0|CASB_BUBBU	LPLLQSWMHQPPQPLPPTVMFPPQSVLSL	3277,74	137	165	2,48E+07
sp|Q9TSI0|CASB_BUBBU	PLLQS	556,32	138	142	4,46E+05
sp|Q9TSI0|CASB_BUBBU	PLLQSW	742,40	138	143	1,58E+06
sp|Q9TSI0|CASB_BUBBU	LLQSW	645,35	139	143	7,36E+07
sp|Q9TSI0|CASB_BUBBU	LLQSWMHQPPQPLPPTVMFPPQSVLSL	3067,60	139	165	1,15E+07
sp|Q9TSI0|CASB_BUBBU	LQSWMHQPPQPLPPT	1755,86	140	154	5,04E+06
sp|Q9TSI0|CASB_BUBBU	LQSWMHQPPQPLPPTVMFPPQSVL	2754,40	140	163	5,29E+07
sp|Q9TSI0|CASB_BUBBU	LQSWMHQPPQPLPPTVMFPPQSVLSL	2954,52	140	165	1,88E+07
sp|Q9TSI0|CASB_BUBBU	QSWMHQPP	1009,44	141	148	3,19E+07
sp|Q9TSI0|CASB_BUBBU	QSWMHQPPQPLPPT	1642,79	141	154	2,73E+07
sp|Q9TSI0|CASB_BUBBU	QSWMHQPPQPLPPTVMFPP	2214,08	141	159	0,00E+00
sp|Q9TSI0|CASB_BUBBU	QSWMHQPPQPLPPTVMFPPQ	2342,13	141	160	1,03E+07
sp|Q9TSI0|CASB_BUBBU	QSWMHQPPQPLPPTVMFPPQS	2429,17	141	161	4,12E+07
sp|Q9TSI0|CASB_BUBBU	QSWMHQPPQPLPPTVMFPPQSV	2528,23	141	162	1,45E+08
sp|Q9TSI0|CASB_BUBBU	QSWMHQPPQPLPPTVMFPPQSVL	2641,32	141	163	8,07E+08
sp|Q9TSI0|CASB_BUBBU	QSWMHQPHQPLPPTVMFPPQSVLS	2768,36	141	164	4,73E+06
sp|Q9TSI0|CASB_BUBBU	QSWMHQPPQPLPPTVMFPPQSVLS	2728,35	141	164	2,47E+08
sp|Q9TSI0|CASB_BUBBU	QSWMHQPPQPLPPTVMFPPQSVLSL	2841,43	141	165	1,67E+08
sp|Q9TSI0|CASB_BUBBU	QSWMHQPPQPLPPTVMFPPQSVLSLSQ	3056,53	141	167	3,54E+07
sp|Q9TSI0|CASB_BUBBU	QSWMHQPPQPLPPTVMFPPQSVLSLSQSK	3271,65	141	169	9,17E+07
sp|Q9TSI0|CASB_BUBBU	QSWMHQPPQPLPPTVMFPPQSVLSLSQSKVLPVPQ	3905,04	141	175	1,13E+08
sp|Q9TSI0|CASB_BUBBU	QSWMHQPPQPLPPTVMFPPQSVLSLSQSKVLPVPQK	4033,13	141	176	9,78E+07
sp|Q9TSI0|CASB_BUBBU	SWMHQP	784,33	142	147	3,34E+07
sp|Q9TSI0|CASB_BUBBU	SWMHQPP	881,39	142	148	2,84E+08
sp|Q9TSI0|CASB_BUBBU	SWMHQPHQPLPPT	1554,74	142	154	0,00E+00
sp|Q9TSI0|CASB_BUBBU	SWMHQPPQPLPPT	1514,73	142	154	8,28E+07
sp|Q9TSI0|CASB_BUBBU	SWMHQPPQPLPPTV	1613,80	142	155	1,23E+06
sp|Q9TSI0|CASB_BUBBU	SWMHQPPQPLPPTVMFPP	2086,02	142	159	8,77E+06
sp|Q9TSI0|CASB_BUBBU	SWMHQPPQPLPPTVMFPPQ	2214,08	142	160	2,06E+08

sp|Q9TSI0|CASB_BUBBU	SWMHQPPQPLPPTVMFPPQS	2301,11	142	161	7,06E+07
sp|Q9TSI0|CASB_BUBBU	SWMHQPHQPLPPTVMFPPQSV	2440,18	142	162	9,95E+06
sp|Q9TSI0|CASB_BUBBU	SWMHQPPQPLPPTVMFPPQSV	2400,18	142	162	3,34E+08
sp|Q9TSI0|CASB_BUBBU	SWMHQPPQPLPPTVMFPPQSVL	2513,26	142	163	1,93E+09
sp|Q9TSI0|CASB_BUBBU	SWMHQPHQPLPPTVMFPPQSVLS	2640,30	142	164	1,92E+07
sp|Q9TSI0|CASB_BUBBU	SWMHQPPQPLPPTVMFPPQSVLS	2600,29	142	164	6,43E+08
sp|Q9TSI0|CASB_BUBBU	SWMHQPHQPLPPTVMFPPQSVLSL	2753,38	142	165	3,60E+07
sp|Q9TSI0|CASB_BUBBU	SWMHQPPQPLPPTVMFPPQSVLSL	2713,38	142	165	7,95E+08
sp|Q9TSI0|CASB_BUBBU	SWMHQPPQPLPPTVMFPPQSVLSLS	2800,41	142	166	1,09E+08
sp|Q9TSI0|CASB_BUBBU	SWMHQPPQPLPPTVMFPPQSVLSLSQS	3015,50	142	168	7,29E+07
sp|Q9TSI0|CASB_BUBBU	SWMHQPPQPLPPTVMFPPQSVLSLSQSK	3143,59	142	169	2,29E+08
sp|Q9TSI0|CASB_BUBBU	SWMHQPPQPLPPTVMFPPQSVLSLSQSKVLPVPQ	3776,98	142	175	2,45E+08
sp|Q9TSI0|CASB_BUBBU	SWMHQPPQPLPPTVMFPPQSVLSLSQSKVLPVPQK	3905,07	142	176	3,32E+08
sp|Q9TSI0|CASB_BUBBU	SWMHQPPQPLPPTVMFPPQSVLSLSQSKVLPVPQKAVPYP	4432,35	142	181	1,71E+08
sp|Q9TSI0|CASB_BUBBU	WMHQPP	794,35	143	148	5,20E+09
sp|Q9TSI0|CASB_BUBBU	WMHQPPQP	1019,46	143	150	2,00E+06
sp|Q9TSI0|CASB_BUBBU	WMHQPPQPLPP	1326,65	143	153	3,84E+06
sp|Q9TSI0|CASB_BUBBU	WMHQPPQPLPPT	1427,70	143	154	8,60E+07
sp|Q9TSI0|CASB_BUBBU	WMHQPPQPLPPTV	1526,77	143	155	1,24E+06
sp|Q9TSI0|CASB_BUBBU	WMHQPPQPLPPTVMFPPQ	2127,04	143	160	9,31E+07
sp|Q9TSI0|CASB_BUBBU	WMHQPPQPLPPTVMFPPQS	2214,08	143	161	1,29E+08
sp|Q9TSI0|CASB_BUBBU	WMHQPPQPLPPTVMFPPQSV	2313,14	143	162	6,57E+07
sp|Q9TSI0|CASB_BUBBU	WMHQPHQPLPPTVMFPPQSVL	2466,23	143	163	1,66E+08
sp|Q9TSI0|CASB_BUBBU	WMHQPPQPLPPTVMFPPQSVL	2426,23	143	163	9,88E+08
sp|Q9TSI0|CASB_BUBBU	WMHQPPQPLPPTVMFPPQSVLS	2513,26	143	164	5,53E+08
sp|Q9TSI0|CASB_BUBBU	WMHQPPQPLPPTVMFPPQSVLSL	2626,34	143	165	5,23E+08
sp|Q9TSI0|CASB_BUBBU	WMHQPPQPLPPTVMFPPQSVLSLS	2713,38	143	166	5,41E+07
sp|Q9TSI0|CASB_BUBBU	WMHQPPQPLPPTVMFPPQSVLSLSQ	2841,43	143	167	1,07E+08
sp|Q9TSI0|CASB_BUBBU	WMHQPPQPLPPTVMFPPQSVLSLSQS	2928,47	143	168	6,05E+07
sp|Q9TSI0|CASB_BUBBU	WMHQPPQPLPPTVMFPPQSVLSLSQSK	3056,56	143	169	1,93E+08
sp|Q9TSI0|CASB_BUBBU	WMHQPPQPLPPTVMFPPQSVLSLSQSKVLPVPQ	3689,95	143	175	3,73E+08
sp|Q9TSI0|CASB_BUBBU	WMHQPPQPLPPTVMFPPQSVLSLSQSKVLPVPQK	3818,04	143	176	2,63E+08
sp|Q9TSI0|CASB_BUBBU	WMHQPPQPLPPTVMFPPQSVLSLSQSKVLPVPQKAVPYPQ	4473,37	143	182	1,09E+09
sp|Q9TSI0|CASB_BUBBU	MHQPP	608,27	144	148	9,09E+06
sp|Q9TSI0|CASB_BUBBU	MHQPPQP	833,39	144	150	1,63E+07
sp|Q9TSI0|CASB_BUBBU	MHQPHQPLPPT	1281,63	144	154	1,70E+07
sp|Q9TSI0|CASB_BUBBU	MHQPPQPLPPT	1241,62	144	154	4,82E+07
sp|Q9TSI0|CASB_BUBBU	MHQPPQPLPPTV	1340,69	144	155	0,00E+00
sp|Q9TSI0|CASB_BUBBU	MHQPPQPLPPTVM	1471,73	144	156	5,82E+06
sp|Q9TSI0|CASB_BUBBU	MHQPPQPLPPTVMF	1618,80	144	157	1,54E+06
sp|Q9TSI0|CASB_BUBBU	MHQPHQPLPPTVMFPPQ	1980,97	144	160	6,32E+07
sp|Q9TSI0|CASB_BUBBU	MHQPPQPLPPTVMFPPQ	1940,96	144	160	1,63E+08
sp|Q9TSI0|CASB_BUBBU	MHQPHQPLPPTVMFPPQS	2068,00	144	161	2,79E+06
sp|Q9TSI0|CASB_BUBBU	MHQPPQPLPPTVMFPPQS	2028,00	144	161	1,19E+08
sp|Q9TSI0|CASB_BUBBU	MHQPHQPLPPTVMFPPQSV	2167,07	144	162	8,60E+07
sp|Q9TSI0|CASB_BUBBU	MHQPPQPLPPTVMFPPQSV	2127,06	144	162	2,91E+08
sp|Q9TSI0|CASB_BUBBU	MHQPHQPLPPTVMFPPQSVL	2280,15	144	163	4,82E+08
sp|Q9TSI0|CASB_BUBBU	MHQPPQPLPPTVMFPPQSVL	2240,15	144	163	3,71E+09
sp|Q9TSI0|CASB_BUBBU	MHQPHQPLPPTVMFPPQSVLS	2367,19	144	164	2,46E+08
sp|Q9TSI0|CASB_BUBBU	MHQPPQPLPPTVMFPPQSVLS	2327,18	144	164	9,13E+08
sp|Q9TSI0|CASB_BUBBU	MHQPHQPLPPTVMFPPQSVLSL	2480,27	144	165	2,13E+08
sp|Q9TSI0|CASB_BUBBU	MHQPPQPLPPTVMFPPQSVLSL	2440,26	144	165	2,32E+09
sp|Q9TSI0|CASB_BUBBU	MHQPPQPLPPTVMFPPQSVLSLS	2527,30	144	166	3,09E+08
sp|Q9TSI0|CASB_BUBBU	MHQPHQPLPPTVMFPPQSVLSLSQ	2695,36	144	167	3,20E+07
sp|Q9TSI0|CASB_BUBBU	MHQPPQPLPPTVMFPPQSVLSLSQ	2655,36	144	167	2,10E+08
sp|Q9TSI0|CASB_BUBBU	MHQPHQPLPPTVMFPPQSVLSLSQS	2782,39	144	168	1,42E+07
sp|Q9TSI0|CASB_BUBBU	MHQPPQPLPPTVMFPPQSVLSLSQS	2742,39	144	168	1,82E+08
sp|Q9TSI0|CASB_BUBBU	MHQPPQPLPPTVMFPPQSVLSLSQSK	2870,48	144	169	5,13E+08
sp|Q9TSI0|CASB_BUBBU	MHQPPQPLPPTVMFPPQSVLSLSQSKVLPVP	3375,81	144	174	2,28E+07
sp|Q9TSI0|CASB_BUBBU	MHQPPQPLPPTVMFPPQSVLSLSQSKVLPVPQ	3503,87	144	175	7,60E+08
sp|Q9TSI0|CASB_BUBBU	MHQPPQPLPPTVMFPPQSVLSLSQSKVLPVPQK	3631,96	144	176	9,62E+08
sp|Q9TSI0|CASB_BUBBU	MHQPPQPLPPTVMFPPQSVLSLSQSKVLPVPQKAVPYP	4159,24	144	181	3,38E+08
sp|Q9TSI0|CASB_BUBBU	MHQPPQPLPPTVMFPPQSVLSLSQSKVLPVPQKAVPYPQ	4287,30	144	182	3,43E+09
sp|Q9TSI0|CASB_BUBBU	MHQPPQPLPPTVMFPPQSVLSLSQSKVLPVPQKAVPYPQR	4443,40	144	183	2,68E+09
sp|Q9TSI0|CASB_BUBBU	HQPPQP	702,34	145	150	1,13E+06
sp|Q9TSI0|CASB_BUBBU	HQPPQPLPPT	1110,58	145	154	5,47E+07
sp|Q9TSI0|CASB_BUBBU	HQPPQPLPPTVM	1340,69	145	156	4,37E+06
sp|Q9TSI0|CASB_BUBBU	HQPPQPLPPTVMFPP	1681,87	145	159	0,00E+00
sp|Q9TSI0|CASB_BUBBU	HQPHQPLPPTVMFPPQ	1849,93	145	160	1,42E+07
sp|Q9TSI0|CASB_BUBBU	HQPPQPLPPTVMFPPQ	1809,92	145	160	2,09E+07
sp|Q9TSI0|CASB_BUBBU	HQPHQPLPPTVMFPPQS	1936,96	145	161	5,41E+05
sp|Q9TSI0|CASB_BUBBU	HQPPQPLPPTVMFPPQS	1896,96	145	161	2,24E+07
sp|Q9TSI0|CASB_BUBBU	HQPHQPLPPTVMFPPQSV	2036,03	145	162	3,03E+07
sp|Q9TSI0|CASB_BUBBU	HQPPQPLPPTVMFPPQSV	1996,02	145	162	7,47E+07
sp|Q9TSI0|CASB_BUBBU	HQPHQPLPPTVMFPPQSVL	2149,11	145	163	2,43E+07
sp|Q9TSI0|CASB_BUBBU	HQPPQPLPPTVMFPPQSVL	2109,11	145	163	3,77E+08
sp|Q9TSI0|CASB_BUBBU	HQPHQPLPPTVMFPPQSVLS	2236,15	145	164	2,26E+07
sp|Q9TSI0|CASB_BUBBU	HQPPQPLPPTVMFPPQSVLS	2196,14	145	164	8,05E+07
sp|Q9TSI0|CASB_BUBBU	HQPPQPLPPTVMFPPQSVLSL	2309,22	145	165	2,08E+08
sp|Q9TSI0|CASB_BUBBU	HQPPQPLPPTVMFPPQSVLSLS	2396,26	145	166	1,60E+07
sp|Q9TSI0|CASB_BUBBU	HQPPQPLPPTVMFPPQSVLSLSQS	2611,35	145	168	2,89E+07
sp|Q9TSI0|CASB_BUBBU	HQPPQPLPPTVMFPPQSVLSLSQSK	2739,44	145	169	3,28E+07
sp|Q9TSI0|CASB_BUBBU	HQPPQPLPPTVMFPPQSVLSLSQSKVLPVPQ	3372,83	145	175	4,92E+07
sp|Q9TSI0|CASB_BUBBU	HQPPQPLPPTVMFPPQSVLSLSQSKVLPVPQK	3500,92	145	176	1,36E+08
sp|Q9TSI0|CASB_BUBBU	HQPPQPLPPTVMFPPQSVLSLSQSKVLPVPQKAVPYPQ	4156,25	145	182	2,25E+08
sp|Q9TSI0|CASB_BUBBU	HQPPQPLPPTVMFPPQSVLSLSQSKVLPVPQKAVPYPQR	4312,36	145	183	2,17E+08
sp|Q9TSI0|CASB_BUBBU	QPPQPLPPT	973,52	146	154	1,99E+06
sp|Q9TSI0|CASB_BUBBU	QPPQPLPPTVM	1203,63	146	156	0,00E+00
sp|Q9TSI0|CASB_BUBBU	QPHQPLPPTVMFPPQ	1712,87	146	160	3,65E+06
sp|Q9TSI0|CASB_BUBBU	QPPQPLPPTVMFPPQ	1672,86	146	160	2,55E+07
sp|Q9TSI0|CASB_BUBBU	QPPQPLPPTVMFPPQS	1759,90	146	161	9,09E+06
sp|Q9TSI0|CASB_BUBBU	QPPQPLPPTVMFPPQSV	1858,97	146	162	1,14E+07
sp|Q9TSI0|CASB_BUBBU	QPHQPLPPTVMFPPQSVL	2012,06	146	163	1,77E+07
sp|Q9TSI0|CASB_BUBBU	QPPQPLPPTVMFPPQSVL	1972,05	146	163	4,98E+07
sp|Q9TSI0|CASB_BUBBU	QPPQPLPPTVMFPPQSVLS	2059,08	146	164	2,22E+07
sp|Q9TSI0|CASB_BUBBU	QPPQPLPPTVMFPPQSVLSL	2172,17	146	165	4,09E+07
sp|Q9TSI0|CASB_BUBBU	QPPQPLPPTVMFPPQSVLSLSQ	2387,26	146	167	1,01E+07
sp|Q9TSI0|CASB_BUBBU	QPPQPLPPTVMFPPQSVLSLSQSKVLPVPQKAVPYPQR	4175,30	146	183	6,04E+07

sp|Q9TSI0|CASB_BUBBU	HQPLPP	687,37	148	153	1,66E+06
sp|Q9TSI0|CASB_BUBBU	HQPLPPT	788,42	148	154	6,78E+06
sp|Q9TSI0|CASB_BUBBU	HQPLPPTVMFPPQ	1487,76	148	160	2,95E+07
sp|Q9TSI0|CASB_BUBBU	HQPLPPTVMFPPQS	1574,79	148	161	1,51E+07
sp|Q9TSI0|CASB_BUBBU	HQPLPPTVMFPPQSVL	1786,94	148	163	3,52E+07
sp|Q9TSI0|CASB_BUBBU	HQPLPPTVMFPPQSVLS	1873,98	148	164	3,52E+07
sp|Q9TSI0|CASB_BUBBU	HQPLPPTVMFPPQSVLSL	1987,06	148	165	4,21E+07
sp|Q9TSI0|CASB_BUBBU	HQPLPPTVMFPPQSVLSLSQSK	2417,28	148	169	1,55E+07
sp|Q9TSI0|CASB_BUBBU	QPLPPT	651,36	149	154	4,60E+07
sp|Q9TSI0|CASB_BUBBU	QPLPPTVM	881,47	149	156	1,23E+06
sp|Q9TSI0|CASB_BUBBU	QPLPPTVMFPP	1222,64	149	159	1,72E+07
sp|Q9TSI0|CASB_BUBBU	QPLPPTVMFPPQ	1350,70	149	160	1,23E+08
sp|Q9TSI0|CASB_BUBBU	QPLPPTVMFPPQS	1437,73	149	161	5,31E+07
sp|Q9TSI0|CASB_BUBBU	QPLPPTVMFPPQSV	1536,80	149	162	1,22E+07
sp|Q9TSI0|CASB_BUBBU	QPLPPTVMFPPQSVL	1649,89	149	163	1,06E+09
sp|Q9TSI0|CASB_BUBBU	QPLPPTVMFPPQSVLS	1736,92	149	164	2,60E+08
sp|Q9TSI0|CASB_BUBBU	QPLPPTVMFPPQSVLSL	1850,00	149	165	6,62E+08
sp|Q9TSI0|CASB_BUBBU	QPLPPTVMFPPQSVLSLS	1937,03	149	166	8,91E+07
sp|Q9TSI0|CASB_BUBBU	QPLPPTVMFPPQSVLSLSQ	2065,09	149	167	1,84E+08
sp|Q9TSI0|CASB_BUBBU	QPLPPTVMFPPQSVLSLSQS	2152,12	149	168	9,39E+07
sp|Q9TSI0|CASB_BUBBU	QPLPPTVMFPPQSVLSLSQSK	2280,22	149	169	1,93E+08
sp|Q9TSI0|CASB_BUBBU	QPLPPTVMFPPQSVLSLSQSKVLPVP	2785,55	149	174	2,92E+07
sp|Q9TSI0|CASB_BUBBU	QPLPPTVMFPPQSVLSLSQSKVLPVPQ	2913,60	149	175	3,10E+08
sp|Q9TSI0|CASB_BUBBU	QPLPPTVMFPPQSVLSLSQSKVLPVPQK	3041,70	149	176	6,87E+08
sp|Q9TSI0|CASB_BUBBU	QPLPPTVMFPPQSVLSLSQSKVLPVPQKAVPYP	3568,97	149	181	1,53E+08
sp|Q9TSI0|CASB_BUBBU	QPLPPTVMFPPQSVLSLSQSKVLPVPQKAVPYPQ	3697,03	149	182	2,12E+09
sp|Q9TSI0|CASB_BUBBU	QPLPPTVMFPPQSVLSLSQSKVLPVPQKAVPYPQR	3853,13	149	183	2,33E+09
sp|Q9TSI0|CASB_BUBBU	QPLPPTVMFPPQSVLSLSQSKVLPVPQKAVPYPQRDMP	4196,25	149	186	2,83E+07
sp|Q9TSI0|CASB_BUBBU	QPLPPTVMFPPQSVLSLSQSKVLPVPQKAVPYPQRDMPIQ	4437,40	149	188	4,34E+08
sp|Q9TSI0|CASB_BUBBU	LPPTVMFPP	997,53	151	159	6,71E+06
sp|Q9TSI0|CASB_BUBBU	LPPTVMFPPQ	1125,59	151	160	6,28E+07
sp|Q9TSI0|CASB_BUBBU	LPPTVMFPPQS	1212,62	151	161	2,88E+07
sp|Q9TSI0|CASB_BUBBU	LPPTVMFPPQSV	1311,69	151	162	3,25E+07
sp|Q9TSI0|CASB_BUBBU	LPPTVMFPPQSVL	1424,77	151	163	1,28E+08
sp|Q9TSI0|CASB_BUBBU	LPPTVMFPPQSVLS	1511,81	151	164	4,25E+07
sp|Q9TSI0|CASB_BUBBU	LPPTVMFPPQSVLSL	1624,89	151	165	5,55E+07
sp|Q9TSI0|CASB_BUBBU	LPPTVMFPPQSVLSLS	1711,92	151	166	9,96E+06
sp|Q9TSI0|CASB_BUBBU	LPPTVMFPPQSVLSLSQ	1839,98	151	167	8,74E+06
sp|Q9TSI0|CASB_BUBBU	LPPTVMFPPQSVLSLSQSKVLPVPQ	2688,49	151	175	1,60E+07
sp|Q9TSI0|CASB_BUBBU	LPPTVMFPPQSVLSLSQSKVLPVPQKAVPYPQ	3471,92	151	182	4,66E+07
sp|Q9TSI0|CASB_BUBBU	TVMFPP	690,34	154	159	3,65E+07
sp|Q9TSI0|CASB_BUBBU	TVMFPPQ	818,40	154	160	7,10E+07
sp|Q9TSI0|CASB_BUBBU	TVMFPPQS	905,43	154	161	8,95E+06
sp|Q9TSI0|CASB_BUBBU	TVMFPPQSV	1004,50	154	162	3,64E+07
sp|Q9TSI0|CASB_BUBBU	TVMFPPQSVL	1117,58	154	163	3,24E+08
sp|Q9TSI0|CASB_BUBBU	TVMFPPQSVLS	1204,62	154	164	4,87E+07
sp|Q9TSI0|CASB_BUBBU	TVMFPPQSVLSL	1317,70	154	165	2,20E+08
sp|Q9TSI0|CASB_BUBBU	TVMFPPQSVLSLS	1404,73	154	166	2,93E+07
sp|Q9TSI0|CASB_BUBBU	TVMFPPQSVLSLSQ	1532,79	154	167	9,17E+07
sp|Q9TSI0|CASB_BUBBU	TVMFPPQSVLSLSQS	1619,82	154	168	1,15E+07
sp|Q9TSI0|CASB_BUBBU	TVMFPPQSVLSLSQSK	1747,92	154	169	3,58E+07
sp|Q9TSI0|CASB_BUBBU	TVMFPPQSVLSLSQSKVLPVPQK	2509,40	154	176	5,11E+08
sp|Q9TSI0|CASB_BUBBU	TVMFPPQSVLSLSQSKVLPVPQKAVPYPQ	3164,73	154	182	1,30E+09
sp|Q9TSI0|CASB_BUBBU	TVMFPPQSVLSLSQSKVLPVPQKAVPYPQR	3320,83	154	183	3,74E+08
sp|Q9TSI0|CASB_BUBBU	TVMFPPQSVLSLSQSKVLPVPQKAVPYPQRDMPIQ	3905,09	154	188	1,57E+08
sp|Q9TSI0|CASB_BUBBU	TVMFPPQSVLSLSQSKVLPVPQKAVPYPQRDMPIQA	3976,13	154	189	2,74E+09
sp|Q9TSI0|CASB_BUBBU	TVMFPPQSVLSLSQSKVLPVPQKAVPYPQRDMPIQAF	4123,20	154	190	2,55E+09
sp|Q9TSI0|CASB_BUBBU	TVMFPPQSVLSLSQSKVLPVPQKAVPYPQRDMPIQAFL	4236,28	154	191	3,15E+08
sp|Q9TSI0|CASB_BUBBU	TVMFPPQSVLSLSQSKVLPVPQKAVPYPQRDMPIQAFLL	4349,37	154	192	4,69E+07
sp|Q9TSI0|CASB_BUBBU	VMFPP	589,29	155	159	1,79E+07
sp|Q9TSI0|CASB_BUBBU	VMFPPQ	717,35	155	160	7,61E+05
sp|Q9TSI0|CASB_BUBBU	VMFPPQS	804,38	155	161	5,25E+06
sp|Q9TSI0|CASB_BUBBU	VMFPPQSV	903,45	155	162	0,00E+00
sp|Q9TSI0|CASB_BUBBU	VMFPPQSVL	1016,54	155	163	8,82E+07
sp|Q9TSI0|CASB_BUBBU	VMFPPQSVLS	1103,57	155	164	2,16E+07
sp|Q9TSI0|CASB_BUBBU	VMFPPQSVLSL	1216,65	155	165	4,73E+07
sp|Q9TSI0|CASB_BUBBU	VMFPPQSVLSLS	1303,68	155	166	5,99E+06
sp|Q9TSI0|CASB_BUBBU	VMFPPQSVLSLSQSK	1646,87	155	169	3,38E+06
sp|Q9TSI0|CASB_BUBBU	VMFPPQSVLSLSQSKVLPVPQKAVPYPQRDMPIQA	3875,08	155	189	5,94E+07
sp|Q9TSI0|CASB_BUBBU	MFPPQSVL	917,47	156	163	1,17E+07
sp|Q9TSI0|CASB_BUBBU	MFPPQSVLSL	1117,58	156	165	3,59E+06
sp|Q9TSI0|CASB_BUBBU	FPPQS	574,28	157	161	2,53E+06
sp|Q9TSI0|CASB_BUBBU	FPPQSVL	786,43	157	163	1,69E+07
sp|Q9TSI0|CASB_BUBBU	FPPQSVLS	873,46	157	164	2,29E+06
sp|Q9TSI0|CASB_BUBBU	FPPQSVLSL	986,54	157	165	8,64E+06
sp|Q9TSI0|CASB_BUBBU	FPPQSVLSLSQ	1201,63	157	167	0,00E+00
sp|Q9TSI0|CASB_BUBBU	PPQSVL	639,36	158	163	3,76E+06
sp|Q9TSI0|CASB_BUBBU	PPQSVLSL	839,48	158	165	3,36E+06
sp|Q9TSI0|CASB_BUBBU	PPQSVLSLSQ	1054,57	158	167	2,20E+06
sp|Q9TSI0|CASB_BUBBU	PPQSVLSLSQS	1141,60	158	168	2,30E+06
sp|Q9TSI0|CASB_BUBBU	PPQSVLSLSQSK	1269,69	158	169	4,80E+06
sp|Q9TSI0|CASB_BUBBU	PPQSVLSLSQSKVLPVPQKAVPYPQ	2686,51	158	182	7,53E+06
sp|Q9TSI0|CASB_BUBBU	PPQSVLSLSQSKVLPVPQKAVPYPQR	2842,61	158	183	4,72E+06
sp|Q9TSI0|CASB_BUBBU	PPQSVLSLSQSKVLPVPQKAVPYPQRDMPIQA	3497,91	158	189	1,12E+07
sp|Q9TSI0|CASB_BUBBU	QSVLSL	645,37	160	165	2,96E+07
sp|Q9TSI0|CASB_BUBBU	QSVLSLSQ	860,46	160	167	1,09E+07
sp|Q9TSI0|CASB_BUBBU	QSVLSLSQS	947,49	160	168	1,43E+07
sp|Q9TSI0|CASB_BUBBU	QSVLSLSQSK	1075,59	160	169	5,05E+07
sp|Q9TSI0|CASB_BUBBU	QSVLSLSQSKVLP	1384,79	160	172	4,79E+06
sp|Q9TSI0|CASB_BUBBU	QSVLSLSQSKVLPVPQ	1708,97	160	175	2,07E+07
sp|Q9TSI0|CASB_BUBBU	QSVLSLSQSKVLPVPQK	1837,07	160	176	3,19E+07
sp|Q9TSI0|CASB_BUBBU	QSVLSLSQSKVLPVPQKAVPYP	2364,34	160	181	2,31E+07
sp|Q9TSI0|CASB_BUBBU	QSVLSLSQSKVLPVPQKAVPYPQ	2492,40	160	182	1,33E+08
sp|Q9TSI0|CASB_BUBBU	QSVLSLSQSKVLPVPQKAVPYPQR	2648,50	160	183	4,49E+07
sp|Q9TSI0|CASB_BUBBU	QSVLSLSQSKVLPVPQKAVPYPQRDMPIQ	3232,76	160	188	1,47E+07
sp|Q9TSI0|CASB_BUBBU	QSVLSLSQSKVLPVPQKAVPYPQRDMPIQA	3303,80	160	189	2,62E+08
sp|Q9TSI0|CASB_BUBBU	QSVLSLSQSKVLPVPQKAVPYPQRDMPIQAF	3450,87	160	190	2,32E+07
sp|Q9TSI0|CASB_BUBBU	SVLSL	517,31	161	165	7,12E+07

sp|Q9TSI0|CASB_BUBBU	SVLSLSQS	819,43	161	168	6,44E+07
sp|Q9TSI0|CASB_BUBBU	SVLSLSQSK	947,53	161	169	1,06E+08
sp|Q9TSI0|CASB_BUBBU	SVLSLSQSKVLPVPQ	1580,91	161	175	1,96E+07
sp|Q9TSI0|CASB_BUBBU	SVLSLSQSKVLPVPQK	1709,01	161	176	1,41E+07
sp|Q9TSI0|CASB_BUBBU	SVLSLSQSKVLPVPQKAVPYPQ	2364,34	161	182	1,22E+08
sp|Q9TSI0|CASB_BUBBU	SVLSLSQSKVLPVPQKAVPYPQR	2520,44	161	183	5,98E+06
sp|Q9TSI0|CASB_BUBBU	SVLSLSQSKVLPVPQKAVPYPQRDMPIQ	3104,71	161	188	7,06E+06
sp|Q9TSI0|CASB_BUBBU	SVLSLSQSKVLPVPQKAVPYPQRDMPIQA	3175,74	161	189	2,38E+08
sp|Q9TSI0|CASB_BUBBU	SVLSLSQSKVLPVPQKAVPYPQRDMPIQAF	3322,81	161	190	1,85E+08
sp|Q9TSI0|CASB_BUBBU	VLSLSQSK	860,50	162	169	6,71E+07
sp|Q9TSI0|CASB_BUBBU	VLSLSQSKVLPVPQ	1493,88	162	175	8,52E+06
sp|Q9TSI0|CASB_BUBBU	VLSLSQSKVLPVPQKAVPYPQ	2277,31	162	182	2,52E+07
sp|Q9TSI0|CASB_BUBBU	VLSLSQSKVLPVPQKAVPYPQR	2433,41	162	183	1,16E+07
sp|Q9TSI0|CASB_BUBBU	VLSLSQSKVLPVPQKAVPYPQRDMPIQA	3088,71	162	189	4,90E+07
sp|Q9TSI0|CASB_BUBBU	VLSLSQSKVLPVPQKAVPYPQRDMPIQAF	3235,78	162	190	2,22E+06
sp|Q9TSI0|CASB_BUBBU	LSLSQSK	761,43	163	169	1,20E+07
sp|Q9TSI0|CASB_BUBBU	LSLSQSKVLPVPQK	1522,91	163	176	6,96E+06
sp|Q9TSI0|CASB_BUBBU	LSLSQSKVLPVPQKAVPYPQ	2178,24	163	182	3,46E+07
sp|Q9TSI0|CASB_BUBBU	LSLSQSKVLPVPQKAVPYPQR	2334,34	163	183	1,79E+07
sp|Q9TSI0|CASB_BUBBU	LSLSQSKVLPVPQKAVPYPQRDMPIQA	2989,64	163	189	1,07E+08
sp|Q9TSI0|CASB_BUBBU	LSLSQSKVLPVPQKAVPYPQRDMPIQAF	3136,71	163	190	1,47E+08
sp|Q9TSI0|CASB_BUBBU	LSLSQSKVLPVPQKAVPYPQRDMPIQAFL	3249,79	163	191	6,00E+08
sp|Q9TSI0|CASB_BUBBU	SLSQSKVLP	957,55	164	172	1,21E+07
sp|Q9TSI0|CASB_BUBBU	SLSQSKVLPV	1056,62	164	173	1,23E+07
sp|Q9TSI0|CASB_BUBBU	SLSQSKVLPVP	1153,67	164	174	3,15E+07
sp|Q9TSI0|CASB_BUBBU	SLSQSKVLPVPQ	1281,73	164	175	8,01E+08
sp|Q9TSI0|CASB_BUBBU	SLSQSKVLPVPQK	1409,82	164	176	3,72E+08
sp|Q9TSI0|CASB_BUBBU	SLSQSKVLPVPQKA	1480,86	164	177	3,37E+06
sp|Q9TSI0|CASB_BUBBU	SLSQSKVLPVPQKAVP	1676,98	164	179	2,37E+06
sp|Q9TSI0|CASB_BUBBU	SLSQSKVLPVPQKAVPY	1840,05	164	180	1,45E+07
sp|Q9TSI0|CASB_BUBBU	SLSQSKVLPVPQKAVPYP	1937,10	164	181	2,89E+08
sp|Q9TSI0|CASB_BUBBU	SLSQSKVLPVPQKAVPYPQ	2065,16	164	182	3,14E+09
sp|Q9TSI0|CASB_BUBBU	SLSQSKVLPVPQKAVPYPQR	2221,26	164	183	1,84E+09
sp|Q9TSI0|CASB_BUBBU	SLSQSKVLPVPQKAVPYPQRD	2336,29	164	184	2,01E+07
sp|Q9TSI0|CASB_BUBBU	SLSQSKVLPVPQKAVPYPQRDMP	2564,38	164	186	3,36E+07
sp|Q9TSI0|CASB_BUBBU	SLSQSKVLPVPQKAVPYPQRDMPI	2677,46	164	187	2,32E+07
sp|Q9TSI0|CASB_BUBBU	SLSQSKVLPVPQKAVPYPQRDMPIQ	2805,52	164	188	3,81E+08
sp|Q9TSI0|CASB_BUBBU	SLSQSKVLPVPQKAVPYPQRDMPIQA	2876,56	164	189	7,13E+09
sp|Q9TSI0|CASB_BUBBU	SLSQSKVLPVPQKAVPYPQRDMPIQAF	3023,63	164	190	5,65E+09
sp|Q9TSI0|CASB_BUBBU	SLSQSKVLPVPQKAVPYPQRDMPIQAFL	3136,71	164	191	1,38E+09
sp|Q9TSI0|CASB_BUBBU	SLSQSKVLPVPQKAVPYPQRDMPIQAFLL	3249,79	164	192	1,63E+08
sp|Q9TSI0|CASB_BUBBU	LSQSKVL	773,46	165	171	1,60E+07
sp|Q9TSI0|CASB_BUBBU	LSQSKVLPVPQ	1194,70	165	175	1,93E+08
sp|Q9TSI0|CASB_BUBBU	LSQSKVLPVPQK	1322,79	165	176	8,27E+07
sp|Q9TSI0|CASB_BUBBU	LSQSKVLPVPQKAVPYPQ	1978,13	165	182	1,45E+08
sp|Q9TSI0|CASB_BUBBU	LSQSKVLPVPQKAVPYPQR	2134,23	165	183	8,04E+07
sp|Q9TSI0|CASB_BUBBU	LSQSKVLPVPQKAVPYPQRDMPIQ	2718,49	165	188	2,50E+06
sp|Q9TSI0|CASB_BUBBU	LSQSKVLPVPQKAVPYPQRDMPIQA	2789,53	165	189	2,90E+08
sp|Q9TSI0|CASB_BUBBU	LSQSKVLPVPQKAVPYPQRDMPIQAF	2936,59	165	190	2,66E+08
sp|Q9TSI0|CASB_BUBBU	LSQSKVLPVPQKAVPYPQRDMPIQAFL	3049,68	165	191	5,82E+07
sp|Q9TSI0|CASB_BUBBU	LSQSKVLPVPQKAVPYPQRDMPIQAFLL	3162,76	165	192	3,27E+07
sp|Q9TSI0|CASB_BUBBU	SQSKVLPVP	953,55	166	174	4,39E+06
sp|Q9TSI0|CASB_BUBBU	SQSKVLPVPQ	1081,61	166	175	4,27E+08
sp|Q9TSI0|CASB_BUBBU	SQSKVLPVPQK	1209,71	166	176	3,63E+08
sp|Q9TSI0|CASB_BUBBU	SQSKVLPVPQKAVP	1476,87	166	179	3,36E+06
sp|Q9TSI0|CASB_BUBBU	SQSKVLPVPQKAVPYP	1736,98	166	181	1,29E+08
sp|Q9TSI0|CASB_BUBBU	SQSKVLPVPQKAVPYPQ	1865,04	166	182	1,89E+09
sp|Q9TSI0|CASB_BUBBU	SQSKVLPVPQKAVPYPQR	2021,14	166	183	7,42E+08
sp|Q9TSI0|CASB_BUBBU	SQSKVLPVPQKAVPYPQRD	2136,17	166	184	4,88E+06
sp|Q9TSI0|CASB_BUBBU	SQSKVLPVPQKAVPYPQRDMP	2364,26	166	186	3,85E+06
sp|Q9TSI0|CASB_BUBBU	SQSKVLPVPQKAVPYPQRDMPI	2477,35	166	187	1,15E+07
sp|Q9TSI0|CASB_BUBBU	SQSKVLPVPQKAVPYPQRDMPIQ	2605,41	166	188	2,00E+08
sp|Q9TSI0|CASB_BUBBU	SQSKVLPVPQKAVPYPQRDMPIQA	2676,44	166	189	3,50E+09
sp|Q9TSI0|CASB_BUBBU	SQSKVLPVPQKAVPYPQRDMPIQAF	2823,51	166	190	2,43E+09
sp|Q9TSI0|CASB_BUBBU	SQSKVLPVPQKAVPYPQRDMPIQAFL	2936,59	166	191	6,30E+08
sp|Q9TSI0|CASB_BUBBU	SQSKVLPVPQKAVPYPQRDMPIQAFLL	3049,68	166	192	4,66E+08
sp|Q9TSI0|CASB_BUBBU	QSKVLPV	769,47	167	173	0,00E+00
sp|Q9TSI0|CASB_BUBBU	QSKVLPVP	866,52	167	174	0,00E+00
sp|Q9TSI0|CASB_BUBBU	QSKVLPVPQ	994,58	167	175	1,25E+08
sp|Q9TSI0|CASB_BUBBU	QSKVLPVPQK	1122,68	167	176	8,44E+07
sp|Q9TSI0|CASB_BUBBU	QSKVLPVPQKA	1193,71	167	177	0,00E+00
sp|Q9TSI0|CASB_BUBBU	QSKVLPVPQKAVPY	1552,90	167	180	0,00E+00
sp|Q9TSI0|CASB_BUBBU	QSKVLPVPQKAVPYP	1649,95	167	181	1,85E+06
sp|Q9TSI0|CASB_BUBBU	QSKVLPVPQKAVPYPQ	1778,01	167	182	3,66E+08
sp|Q9TSI0|CASB_BUBBU	QSKVLPVPQKAVPYPQR	1934,11	167	183	1,46E+08
sp|Q9TSI0|CASB_BUBBU	QSKVLPVPQKAVPYPQRDMP	2277,23	167	186	3,36E+06
sp|Q9TSI0|CASB_BUBBU	QSKVLPVPQKAVPYPQRDMPIQ	2518,37	167	188	3,01E+07
sp|Q9TSI0|CASB_BUBBU	QSKVLPVPQKAVPYPQRDMPIQA	2589,41	167	189	6,24E+08
sp|Q9TSI0|CASB_BUBBU	QSKVLPVPQKAVPYPQRDMPIQAF	2736,48	167	190	5,73E+08
sp|Q9TSI0|CASB_BUBBU	QSKVLPVPQKAVPYPQRDMPIQAFL	2849,56	167	191	1,13E+08
sp|Q9TSI0|CASB_BUBBU	QSKVLPVPQKAVPYPQRDMPIQAFLL	2962,65	167	192	7,16E+07
sp|Q9TSI0|CASB_BUBBU	SKVLPV	641,41	168	173	1,23E+06
sp|Q9TSI0|CASB_BUBBU	SKVLPVPQ	866,52	168	175	4,59E+08
sp|Q9TSI0|CASB_BUBBU	SKVLPVPQK	994,62	168	176	2,01E+08
sp|Q9TSI0|CASB_BUBBU	SKVLPVPQKAVPYP	1521,89	168	181	3,01E+07
sp|Q9TSI0|CASB_BUBBU	SKVLPVPQKAVPYPQ	1649,95	168	182	7,71E+08
sp|Q9TSI0|CASB_BUBBU	SKVLPVPQKAVPYPQR	1806,05	168	183	2,42E+08
sp|Q9TSI0|CASB_BUBBU	SKVLPVPQKAVPYPQRD	1921,08	168	184	1,54E+06
sp|Q9TSI0|CASB_BUBBU	SKVLPVPQKAVPYPQRDMP	2149,17	168	186	4,59E+06
sp|Q9TSI0|CASB_BUBBU	SKVLPVPQKAVPYPQRDMPIQ	2390,31	168	188	7,75E+07
sp|Q9TSI0|CASB_BUBBU	SKVLPVPQKAVPYPQRDMPIQA	2461,35	168	189	1,11E+09
sp|Q9TSI0|CASB_BUBBU	SKVLPVPQKAVPYPQRDMPIQAF	2608,42	168	190	1,05E+09
sp|Q9TSI0|CASB_BUBBU	SKVLPVPQKAVPYPQRDMPIQAFL	2721,50	168	191	1,76E+08
sp|Q9TSI0|CASB_BUBBU	SKVLPVPQKAVPYPQRDMPIQAFLL	2834,59	168	192	1,13E+08
sp|Q9TSI0|CASB_BUBBU	KVLPVPQ	779,49	169	175	2,78E+08
sp|Q9TSI0|CASB_BUBBU	KVLPVPQK	907,59	169	176	1,06E+08
sp|Q9TSI0|CASB_BUBBU	KVLPVPQKA	978,62	169	177	2,63E+06
sp|Q9TSI0|CASB_BUBBU	KVLPVPQKAVPYP	1434,86	169	181	2,72E+07

sp|Q9TSI0|CASB_BUBBU	KVLPVPQKAVPYPQ	1562,92	169	182	5,80E+08
sp|Q9TSI0|CASB_BUBBU	KVLPVPQKAVPYPQR	1719,02	169	183	1,54E+08
sp|Q9TSI0|CASB_BUBBU	KVLPVPQKAVPYPQRD	1834,05	169	184	2,04E+06
sp|Q9TSI0|CASB_BUBBU	KVLPVPQKAVPYPQRDMP	2062,14	169	186	3,69E+06
sp|Q9TSI0|CASB_BUBBU	KVLPVPQKAVPYPQRDMPI	2175,22	169	187	4,70E+06
sp|Q9TSI0|CASB_BUBBU	KVLPVPQKAVPYPQRDMPIQ	2303,28	169	188	2,24E+07
sp|Q9TSI0|CASB_BUBBU	KVLPVPQKAVPYPQRDMPIQA	2374,32	169	189	8,17E+08
sp|Q9TSI0|CASB_BUBBU	KVLPVPQKAVPYPQRDMPIQAF	2521,39	169	190	6,47E+08
sp|Q9TSI0|CASB_BUBBU	KVLPVPQKAVPYPQRDMPIQAFL	2634,47	169	191	3,72E+07
sp|Q9TSI0|CASB_BUBBU	KVLPVPQKAVPYPQRDMPIQAFLL	2747,56	169	192	7,07E+07
sp|Q9TSI0|CASB_BUBBU	VLPVPQ	651,40	170	175	3,50E+08
sp|Q9TSI0|CASB_BUBBU	VLPVPQK	779,49	170	176	3,91E+08
sp|Q9TSI0|CASB_BUBBU	VLPVPQKA	850,53	170	177	1,74E+07
sp|Q9TSI0|CASB_BUBBU	VLPVPQKAVPYP	1306,76	170	181	7,49E+07
sp|Q9TSI0|CASB_BUBBU	VLPVPQKAVPYPQ	1434,82	170	182	9,92E+08
sp|Q9TSI0|CASB_BUBBU	VLPVPQKAVPYPQR	1590,92	170	183	9,00E+08
sp|Q9TSI0|CASB_BUBBU	VLPVPQKAVPYPQRD	1705,95	170	184	5,59E+05
sp|Q9TSI0|CASB_BUBBU	VLPVPQKAVPYPQRDMP	1934,04	170	186	3,30E+07
sp|Q9TSI0|CASB_BUBBU	VLPVPQKAVPYPQRDMPI	2047,13	170	187	1,45E+07
sp|Q9TSI0|CASB_BUBBU	VLPVPQKAVPYPQRDMPIQ	2175,19	170	188	2,10E+08
sp|Q9TSI0|CASB_BUBBU	VLPVPQKAVPYPQRDMPIQA	2246,22	170	189	3,40E+09
sp|Q9TSI0|CASB_BUBBU	VLPVPQKAVPYPQRDMPIQAF	2393,29	170	190	3,35E+09
sp|Q9TSI0|CASB_BUBBU	VLPVPQKAVPYPQRDMPIQAFL	2506,38	170	191	5,05E+07
sp|Q9TSI0|CASB_BUBBU	LPVPQ	552,33	171	175	1,19E+07
sp|Q9TSI0|CASB_BUBBU	LPVPQKA	751,46	171	177	1,04E+07
sp|Q9TSI0|CASB_BUBBU	LPVPQKAVP	947,58	171	179	2,20E+07
sp|Q9TSI0|CASB_BUBBU	LPVPQKAVPY	1110,64	171	180	2,08E+07
sp|Q9TSI0|CASB_BUBBU	LPVPQKAVPYP	1207,70	171	181	4,44E+07
sp|Q9TSI0|CASB_BUBBU	LPVPQKAVPYPQ	1335,76	171	182	3,82E+08
sp|Q9TSI0|CASB_BUBBU	LPVPQKAVPYPQR	1491,86	171	183	5,19E+07
sp|Q9TSI0|CASB_BUBBU	LPVPQKAVPYPQRDMPIQ	2076,12	171	188	1,77E+07
sp|Q9TSI0|CASB_BUBBU	LPVPQKAVPYPQRDMPIQA	2147,16	171	189	1,49E+08
sp|Q9TSI0|CASB_BUBBU	LPVPQKAVPYPQRDMPIQAF	2294,22	171	190	1,81E+08
sp|Q9TSI0|CASB_BUBBU	LPVPQKAVPYPQRDMPIQAFL	2407,31	171	191	6,58E+07
sp|Q9TSI0|CASB_BUBBU	PVPQK	567,34	172	176	1,38E+06
sp|Q9TSI0|CASB_BUBBU	PVPQKAVPYPQ	1222,67	172	182	1,63E+07
sp|Q9TSI0|CASB_BUBBU	PVPQKAVPYPQR	1378,77	172	183	1,33E+07
sp|Q9TSI0|CASB_BUBBU	PVPQKAVPYPQRDMPIQA	2034,07	172	189	2,31E+07
sp|Q9TSI0|CASB_BUBBU	PVPQKAVPYPQRDMPIQAF	2181,14	172	190	1,82E+07
sp|Q9TSI0|CASB_BUBBU	VPQKAVPY	900,51	173	180	7,42E+06
sp|Q9TSI0|CASB_BUBBU	VPQKAVPYP	997,56	173	181	5,31E+06
sp|Q9TSI0|CASB_BUBBU	VPQKAVPYPQ	1125,62	173	182	8,00E+07
sp|Q9TSI0|CASB_BUBBU	VPQKAVPYPQR	1281,72	173	183	8,26E+06
sp|Q9TSI0|CASB_BUBBU	VPQKAVPYPQRDMPIQ	1865,98	173	188	3,22E+06
sp|Q9TSI0|CASB_BUBBU	VPQKAVPYPQRDMPIQA	1937,02	173	189	3,81E+07
sp|Q9TSI0|CASB_BUBBU	VPQKAVPYPQRDMPIQAF	2084,09	173	190	3,47E+07
sp|Q9TSI0|CASB_BUBBU	VPQKAVPYPQRDMPIQAFL	2197,17	173	191	5,30E+06
sp|Q9TSI0|CASB_BUBBU	PQKAVPYPQR	1182,65	174	183	4,46E+06
sp|Q9TSI0|CASB_BUBBU	PQKAVPYPQRDMPIQAFLLYQEPVLGPVRGPFPI	3861,08	174	207	2,16E+07
sp|Q9TSI0|CASB_BUBBU	QKAVP	541,32	175	179	0,00E+00
sp|Q9TSI0|CASB_BUBBU	QKAVPYPQ	929,50	175	182	1,29E+07
sp|Q9TSI0|CASB_BUBBU	QKAVPYPQR	1085,60	175	183	0,00E+00
sp|Q9TSI0|CASB_BUBBU	QKAVPYPQRDMP	1428,72	175	186	0,00E+00
sp|Q9TSI0|CASB_BUBBU	QKAVPYPQRDMPIQ	1669,86	175	188	3,57E+05
sp|Q9TSI0|CASB_BUBBU	QKAVPYPQRDMPIQA	1740,90	175	189	9,01E+07
sp|Q9TSI0|CASB_BUBBU	QKAVPYPQRDMPIQAF	1887,97	175	190	6,16E+07
sp|Q9TSI0|CASB_BUBBU	QKAVPYPQRDMPIQAFL	2001,05	175	191	1,12E+07
sp|Q9TSI0|CASB_BUBBU	QKAVPYPQRDMPIQAFLL	2114,13	175	192	4,77E+06
sp|Q9TSI0|CASB_BUBBU	KAVPYPQ	801,44	176	182	6,38E+08
sp|Q9TSI0|CASB_BUBBU	KAVPYPQR	957,54	176	183	8,91E+08
sp|Q9TSI0|CASB_BUBBU	KAVPYPQRD	1072,57	176	184	1,28E+07
sp|Q9TSI0|CASB_BUBBU	KAVPYPQRDM	1203,61	176	185	0,00E+00
sp|Q9TSI0|CASB_BUBBU	KAVPYPQRDMPI	1413,74	176	187	5,06E+06
sp|Q9TSI0|CASB_BUBBU	KAVPYPQRDMPIQ	1541,80	176	188	1,77E+08
sp|Q9TSI0|CASB_BUBBU	KAVPYPQRDMPIQA	1612,84	176	189	9,65E+08
sp|Q9TSI0|CASB_BUBBU	KAVPYPQRDMPIQAF	1759,91	176	190	3,56E+08
sp|Q9TSI0|CASB_BUBBU	KAVPYPQRDMPIQAFL	1872,99	176	191	4,90E+06
sp|Q9TSI0|CASB_BUBBU	KAVPYPQRDMPIQAFLL	1986,08	176	192	2,90E+07
sp|Q9TSI0|CASB_BUBBU	AVPYP	545,28	177	181	1,33E+07
sp|Q9TSI0|CASB_BUBBU	AVPYPQ	673,34	177	182	2,80E+08
sp|Q9TSI0|CASB_BUBBU	AVPYPQR	829,44	177	183	1,83E+09
sp|Q9TSI0|CASB_BUBBU	AVPYPQRD	944,47	177	184	3,16E+07
sp|Q9TSI0|CASB_BUBBU	AVPYPQRDM	1075,51	177	185	3,35E+06
sp|Q9TSI0|CASB_BUBBU	AVPYPQRDMP	1172,56	177	186	3,27E+07
sp|Q9TSI0|CASB_BUBBU	AVPYPQRDMPI	1285,65	177	187	3,03E+07
sp|Q9TSI0|CASB_BUBBU	AVPYPQRDMPIQ	1413,71	177	188	5,51E+08
sp|Q9TSI0|CASB_BUBBU	AVPYPQRDMPIQA	1484,74	177	189	3,05E+09
sp|Q9TSI0|CASB_BUBBU	AVPYPQRDMPIQAF	1631,81	177	190	2,30E+09
sp|Q9TSI0|CASB_BUBBU	AVPYPQRDMPIQAFL	1744,90	177	191	6,14E+08
sp|Q9TSI0|CASB_BUBBU	AVPYPQRDMPIQAFLL	1857,98	177	192	7,28E+07
sp|Q9TSI0|CASB_BUBBU	VPYPQ	602,31	178	182	1,36E+08
sp|Q9TSI0|CASB_BUBBU	VPYPQR	758,41	178	183	2,60E+08
sp|Q9TSI0|CASB_BUBBU	VPYPQRDM	1004,48	178	185	4,65E+06
sp|Q9TSI0|CASB_BUBBU	VPYPQRDMP	1101,53	178	186	1,81E+07
sp|Q9TSI0|CASB_BUBBU	VPYPQRDMPIQ	1342,67	178	188	3,06E+08
sp|Q9TSI0|CASB_BUBBU	VPYPQRDMPIQA	1413,71	178	189	1,12E+09
sp|Q9TSI0|CASB_BUBBU	VPYPQRDMPIQAF	1560,78	178	190	6,91E+08
sp|Q9TSI0|CASB_BUBBU	VPYPQRDMPIQAFL	1673,86	178	191	1,30E+08
sp|Q9TSI0|CASB_BUBBU	PYPQRDMPIQA	1314,64	179	189	4,32E+06
sp|Q9TSI0|CASB_BUBBU	PYPQRDMPIQAF	1461,71	179	190	2,83E+06
sp|Q9TSI0|CASB_BUBBU	YPQRDMP	905,41	180	186	6,59E+07
sp|Q9TSI0|CASB_BUBBU	YPQRDMPI	1018,49	180	187	2,12E+07
sp|Q9TSI0|CASB_BUBBU	YPQRDMPIQ	1146,55	180	188	2,49E+08
sp|Q9TSI0|CASB_BUBBU	YPQRDMPIQA	1217,59	180	189	5,82E+08
sp|Q9TSI0|CASB_BUBBU	YPQRDMPIQAF	1364,65	180	190	2,24E+08
sp|Q9TSI0|CASB_BUBBU	YPQRDMPIQAFL	1477,74	180	191	3,10E+07
sp|Q9TSI0|CASB_BUBBU	YPQRDMPIQAFLL	1590,82	180	192	2,00E+07
sp|Q9TSI0|CASB_BUBBU	PQRDMPIQ	983,49	181	188	3,35E+06

sp|Q9TSI0|CASB_BUBBU	PQRDMPIQA	1054,52	181	189	1,75E+07
sp|Q9TSI0|CASB_BUBBU	PQRDMPIQAF	1201,59	181	190	3,71E+06
sp|Q9TSI0|CASB_BUBBU	QRDMPIQ	886,43	182	188	5,48E+07
sp|Q9TSI0|CASB_BUBBU	QRDMPIQA	957,47	182	189	6,57E+08
sp|Q9TSI0|CASB_BUBBU	QRDMPIQAF	1104,54	182	190	3,03E+08
sp|Q9TSI0|CASB_BUBBU	QRDMPIQAFL	1217,62	182	191	2,14E+07
sp|Q9TSI0|CASB_BUBBU	QRDMPIQAFLL	1330,71	182	192	2,01E+07
sp|Q9TSI0|CASB_BUBBU	RDMPIQ	758,37	183	188	0,00E+00
sp|Q9TSI0|CASB_BUBBU	RDMPIQA	829,41	183	189	2,90E+09
sp|Q9TSI0|CASB_BUBBU	RDMPIQAF	976,48	183	190	1,57E+09
sp|Q9TSI0|CASB_BUBBU	RDMPIQAFL	1089,56	183	191	3,69E+08
sp|Q9TSI0|CASB_BUBBU	RDMPIQAFLL	1202,65	183	192	1,93E+08
sp|Q9TSI0|CASB_BUBBU	DMPIQ	602,27	184	188	2,91E+08
sp|Q9TSI0|CASB_BUBBU	DMPIQA	673,31	184	189	5,26E+05
sp|Q9TSI0|CASB_BUBBU	DMPIQAF	820,38	184	190	2,43E+09
sp|Q9TSI0|CASB_BUBBU	DMPIQAFL	933,46	184	191	1,62E+08
sp|Q9TSI0|CASB_BUBBU	DMPIQAFLL	1046,55	184	192	1,94E+08
sp|Q9TSI0|CASB_BUBBU	MPIQA	558,28	185	189	2,44E+09
sp|Q9TSI0|CASB_BUBBU	MPIQAF	705,35	185	190	3,57E+08
sp|Q9TSI0|CASB_BUBBU	MPIQAFL	818,44	185	191	5,93E+08
sp|Q9TSI0|CASB_BUBBU	MPIQAFLL	931,52	185	192	3,05E+08
sp|Q9TSI0|CASB_BUBBU	PIQAFL	687,40	186	191	5,21E+06
sp|Q9TSI0|CASB_BUBBU	PIQAFLLYQEPVLGPVRGPFPII	2563,46	186	208	0,00E+00
sp|Q9TSI0|CASB_BUBBU	AFLLYQEPVLGPVRGP	1754,97	189	204	0,00E+00
sp|Q9TSI0|CASB_BUBBU	AFLLYQEPVLGPVRGPFPI	2112,18	189	207	3,91E+06
sp|Q9TSI0|CASB_BUBBU	AFLLYQEPVLGPVRGPFPIIV	2324,33	189	209	9,81E+06
sp|Q9TSI0|CASB_BUBBU	FLLYQEPVLGPVRG	1586,88	190	203	1,49E+07
sp|Q9TSI0|CASB_BUBBU	FLLYQEPVLGPVRGP	1683,93	190	204	2,67E+08
sp|Q9TSI0|CASB_BUBBU	FLLYQEPVLGPVRGPFP	1928,06	190	206	1,04E+07
sp|Q9TSI0|CASB_BUBBU	FLLYQEPVLGPVRGPFPI	2041,14	190	207	7,23E+07
sp|Q9TSI0|CASB_BUBBU	FLLYQEPVLGPVRGPFPII	2154,22	190	208	5,83E+07
sp|Q9TSI0|CASB_BUBBU	FLLYQEPVLGPVRGPFPIIV	2253,29	190	209	2,69E+08
sp|Q9TSI0|CASB_BUBBU	LLYQEP	761,40	191	196	3,94E+06
sp|Q9TSI0|CASB_BUBBU	LLYQEPVL	973,55	191	198	4,15E+06
sp|Q9TSI0|CASB_BUBBU	LLYQEPVLGPVR	1382,79	191	202	0,00E+00
sp|Q9TSI0|CASB_BUBBU	LLYQEPVLGPVRG	1439,81	191	203	1,19E+08
sp|Q9TSI0|CASB_BUBBU	LLYQEPVLGPVRGP	1536,87	191	204	3,86E+09
sp|Q9TSI0|CASB_BUBBU	LLYQEPVLGPVRGPF	1683,93	191	205	5,36E+07
sp|Q9TSI0|CASB_BUBBU	LLYQEPVLGPVRGPFP	1780,99	191	206	3,99E+08
sp|Q9TSI0|CASB_BUBBU	LLYQEPVLGPVRGPFPI	1894,07	191	207	2,44E+09
sp|Q9TSI0|CASB_BUBBU	LLYQEPVLGPVRGPFPII	2007,16	191	208	1,88E+09
sp|Q9TSI0|CASB_BUBBU	LLYQEPVLGPVRGPFPIIV	2106,22	191	209	4,81E+09
sp|Q9TSI0|CASB_BUBBU	LYQEPVLGPVR	1269,71	192	202	1,15E+07
sp|Q9TSI0|CASB_BUBBU	LYQEPVLGPVRG	1326,73	192	203	2,00E+07
sp|Q9TSI0|CASB_BUBBU	LYQEPVLGPVRGP	1423,78	192	204	3,15E+09
sp|Q9TSI0|CASB_BUBBU	LYQEPVLGPVRGPF	1570,85	192	205	1,48E+07
sp|Q9TSI0|CASB_BUBBU	LYQEPVLGPVRGPFP	1667,90	192	206	1,89E+08
sp|Q9TSI0|CASB_BUBBU	LYQEPVLGPVRGPFPI	1780,99	192	207	2,22E+09
sp|Q9TSI0|CASB_BUBBU	LYQEPVLGPVRGPFPIIV	1993,14	192	209	4,33E+09
sp|Q9TSI0|CASB_BUBBU	YQEPV	634,30	193	197	1,74E+07
sp|Q9TSI0|CASB_BUBBU	YQEPVLGP	901,45	193	200	4,24E+06
sp|Q9TSI0|CASB_BUBBU	YQEPVLGPV	1000,52	193	201	1,92E+07
sp|Q9TSI0|CASB_BUBBU	YQEPVLGPVR	1156,62	193	202	2,86E+08
sp|Q9TSI0|CASB_BUBBU	YQEPVLGPVRG	1213,65	193	203	6,73E+08
sp|Q9TSI0|CASB_BUBBU	YQEPVLGPVRGP	1310,70	193	204	1,44E+10
sp|Q9TSI0|CASB_BUBBU	YQEPVLGPVRGPF	1457,77	193	205	1,31E+08
sp|Q9TSI0|CASB_BUBBU	YQEPVLGPVRGPFP	1554,82	193	206	2,28E+09
sp|Q9TSI0|CASB_BUBBU	YQEPVLGPVRGPFPI	1667,90	193	207	2,28E+10
sp|Q9TSI0|CASB_BUBBU	YQEPVLGPVRGPFPII	1780,99	193	208	1,85E+10
sp|Q9TSI0|CASB_BUBBU	YQEPVLGPVRGPFPIIV	1880,06	193	209	2,36E+10
sp|Q9TSI0|CASB_BUBBU	QEPVL	584,32	194	198	1,88E+08
sp|Q9TSI0|CASB_BUBBU	QEPVLGP	738,39	194	200	2,65E+06
sp|Q9TSI0|CASB_BUBBU	QEPVLGPV	837,46	194	201	1,39E+07
sp|Q9TSI0|CASB_BUBBU	QEPVLGPVR	993,56	194	202	1,22E+08
sp|Q9TSI0|CASB_BUBBU	QEPVLGPVRG	1050,58	194	203	3,90E+08
sp|Q9TSI0|CASB_BUBBU	QEPVLGPVRGP	1147,64	194	204	6,55E+09
sp|Q9TSI0|CASB_BUBBU	QEPVLGPVRGPF	1294,70	194	205	8,93E+07
sp|Q9TSI0|CASB_BUBBU	QEPVLGPVRGPFP	1391,76	194	206	1,15E+09
sp|Q9TSI0|CASB_BUBBU	QEPVLGPVRGPFPI	1504,84	194	207	1,26E+10
sp|Q9TSI0|CASB_BUBBU	QEPVLGPVRGPFPII	1617,92	194	208	1,19E+10
sp|Q9TSI0|CASB_BUBBU	QEPVLGPVRGPFPIIV	1716,99	194	209	1,70E+10
sp|Q9TSI0|CASB_BUBBU	EPVLGP	610,33	195	200	2,47E+06
sp|Q9TSI0|CASB_BUBBU	EPVLGPV	709,40	195	201	1,60E+07
sp|Q9TSI0|CASB_BUBBU	EPVLGPVR	865,50	195	202	2,55E+08
sp|Q9TSI0|CASB_BUBBU	EPVLGPVRG	922,52	195	203	2,20E+08
sp|Q9TSI0|CASB_BUBBU	EPVLGPVRGP	1019,58	195	204	1,03E+09
sp|Q9TSI0|CASB_BUBBU	EPVLGPVRGPF	1166,64	195	205	1,26E+07
sp|Q9TSI0|CASB_BUBBU	EPVLGPVRGPFP	1263,70	195	206	1,49E+08
sp|Q9TSI0|CASB_BUBBU	EPVLGPVRGPFPI	1376,78	195	207	1,60E+09
sp|Q9TSI0|CASB_BUBBU	EPVLGPVRGPFPII	1489,87	195	208	1,60E+09
sp|Q9TSI0|CASB_BUBBU	EPVLGPVRGPFPIIV	1588,93	195	209	2,11E+09
sp|Q9TSI0|CASB_BUBBU	PVLGPV	580,36	196	201	1,71E+06
sp|Q9TSI0|CASB_BUBBU	PVLGPVRG	793,48	196	203	4,72E+06
sp|Q9TSI0|CASB_BUBBU	PVLGPVRGP	890,53	196	204	2,26E+08
sp|Q9TSI0|CASB_BUBBU	PVLGPVRGPFP	1134,66	196	206	1,31E+07
sp|Q9TSI0|CASB_BUBBU	PVLGPVRGPFPI	1247,74	196	207	1,09E+08
sp|Q9TSI0|CASB_BUBBU	PVLGPVRGPFPIIV	1459,89	196	209	3,31E+08
sp|Q9TSI0|CASB_BUBBU	VLGPVRGP	793,48	197	204	1,92E+09
sp|Q9TSI0|CASB_BUBBU	VLGPVRGPF	940,55	197	205	3,39E+07
sp|Q9TSI0|CASB_BUBBU	VLGPVRGPFP	1037,60	197	206	2,72E+08
sp|Q9TSI0|CASB_BUBBU	VLGPVRGPFPI	1150,69	197	207	1,81E+09
sp|Q9TSI0|CASB_BUBBU	VLGPVRGPFPII	1263,77	197	208	1,43E+09
sp|Q9TSI0|CASB_BUBBU	VLGPVRGPFPIIV	1362,84	197	209	4,28E+09
sp|Q9TSI0|CASB_BUBBU	LGPVR	540,34	198	202	1,34E+06
sp|Q9TSI0|CASB_BUBBU	LGPVRG	597,36	198	203	3,40E+07
sp|Q9TSI0|CASB_BUBBU	LGPVRGPF	841,48	198	205	7,17E+06
sp|Q9TSI0|CASB_BUBBU	LGPVRGPFP	938,53	198	206	6,78E+07
sp|Q9TSI0|CASB_BUBBU	LGPVRGPFPI	1051,62	198	207	8,42E+08

sp|Q9TSI0|CASB_BUBBU	LGPVRGPFPII	1164,70	198	208	1,68E+08
sp|Q9TSI0|CASB_BUBBU	LGPVRGPFPIIV	1263,77	198	209	4,90E+08
sp|Q9TSI0|CASB_BUBBU	GPVRG	484,28	199	203	1,12E+06
sp|Q9TSI0|CASB_BUBBU	GPVRGP	581,33	199	204	8,41E+07
sp|Q9TSI0|CASB_BUBBU	GPVRGPFP	825,45	199	206	7,28E+07
sp|Q9TSI0|CASB_BUBBU	GPVRGPFPI	938,53	199	207	5,74E+08
sp|Q9TSI0|CASB_BUBBU	GPVRGPFPII	1051,62	199	208	8,27E+08
sp|Q9TSI0|CASB_BUBBU	GPVRGPFPIIV	1150,69	199	209	2,63E+08
sp|Q9TSI0|CASB_BUBBU	PVRGP	524,31	200	204	3,72E+06
sp|Q9TSI0|CASB_BUBBU	PVRGPFPI	881,51	200	207	2,98E+06
sp|Q9TSI0|CASB_BUBBU	PVRGPFPIIV	1093,66	200	209	2,73E+06
sp|Q9TSI0|CASB_BUBBU	VRGPFPI	784,46	201	207	5,87E+06
sp|Q9TSI0|CASB_BUBBU	VRGPFPIIV	996,61	201	209	7,12E+06
sp|Q9TSI0|CASB_BUBBU	GPFPI	529,29	203	207	2,92E+07
sp|P11840|CASK_BUBBU	QEQNQEQPI	1112,51	1	9	2,32E+06
sp|P11840|CASK_BUBBU	QEQNQEQPIR	1268,61	1	10	0,00E+00
sp|P11840|CASK_BUBBU	QEQPI	613,31	5	9	0,00E+00
sp|P11840|CASK_BUBBU	QEQPIR	769,41	5	10	0,00E+00
sp|P11840|CASK_BUBBU	EKEERF	836,40	12	17	2,75E+06
sp|P11840|CASK_BUBBU	EKEERFF	983,47	12	18	1,51E+06
sp|P11840|CASK_BUBBU	EKEERFFNDK	1340,64	12	21	4,05E+06
sp|P11840|CASK_BUBBU	EERFFN	840,38	14	19	5,65E+06
sp|P11840|CASK_BUBBU	EERFFNDK	1083,50	14	21	1,47E+06
sp|P11840|CASK_BUBBU	ERFFND	826,36	15	20	9,64E+06
sp|P11840|CASK_BUBBU	ERFFNDK	954,46	15	21	3,79E+07
sp|P11840|CASK_BUBBU	ERFFNDKIA	1138,58	15	23	1,06E+07
sp|P11840|CASK_BUBBU	ERFFNDKIAK	1266,67	15	24	2,61E+06
sp|P11840|CASK_BUBBU	RFFNDK	825,41	16	21	1,54E+08
sp|P11840|CASK_BUBBU	RFFNDKIA	1009,53	16	23	4,75E+06
sp|P11840|CASK_BUBBU	FNDKIA	706,36	18	23	6,46E+06
sp|P11840|CASK_BUBBU	FNDKIAK	834,46	18	24	3,12E+07
sp|P11840|CASK_BUBBU	FNDKIAKYIPI	1320,74	18	28	8,39E+06
sp|P11840|CASK_BUBBU	FNDKIAKYIPIQ	1448,80	18	29	3,08E+07
sp|P02668var|CASK_VARI_BOVIN;sp|P02668|CASK_BOVIN	FSDKIAKYIPIQY	1584,86	18	30	4,73E+06
sp|P11840|CASK_BUBBU	FNDKIAKYIPIQY	1611,87	18	30	9,06E+06
sp|P11840|CASK_BUBBU	FNDKIAKYIPIQYVL	1824,02	18	32	1,62E+07
sp|P11840|CASK_BUBBU	NDKIAKYIPIQ	1301,73	19	29	2,21E+07
sp|P11840|CASK_BUBBU	NDKIAKYIPIQY	1464,80	19	30	1,68E+07
sp|P11840|CASK_BUBBU	NDKIAKYIPIQYVL	1676,95	19	32	6,23E+06
sp|P11840|CASK_BUBBU	DKIAKYIP	946,55	20	27	7,20E+06
sp|P11840|CASK_BUBBU	DKIAKYIPI	1059,63	20	28	1,44E+07
sp|P11840|CASK_BUBBU	DKIAKYIPIQ	1187,69	20	29	9,57E+07
sp|P11840|CASK_BUBBU	DKIAKYIPIQY	1350,75	20	30	2,50E+08
sp|P11840|CASK_BUBBU	DKIAKYIPIQYV	1449,82	20	31	3,72E+07
sp|P11840|CASK_BUBBU	DKIAKYIPIQYVL	1562,91	20	32	6,86E+07
sp|P11840|CASK_BUBBU	IAKYIPI	816,51	22	28	4,21E+07
sp|P11840|CASK_BUBBU	IAKYIPIQ	944,57	22	29	1,39E+08
sp|P11840|CASK_BUBBU	IAKYIPIQY	1107,63	22	30	5,66E+08
sp|P11840|CASK_BUBBU	IAKYIPIQYV	1206,70	22	31	3,85E+07
sp|P11840|CASK_BUBBU	IAKYIPIQYVL	1319,79	22	32	4,87E+07
sp|P11840|CASK_BUBBU	IAKYIPIQYVLS	1406,82	22	33	5,12E+06
sp|P11840|CASK_BUBBU	AKYIPIQ	831,49	23	29	6,66E+07
sp|P11840|CASK_BUBBU	AKYIPIQY	994,55	23	30	1,17E+08
sp|P11840|CASK_BUBBU	AKYIPIQYV	1093,62	23	31	1,10E+07
sp|P11840|CASK_BUBBU	AKYIPIQYVL	1206,70	23	32	1,84E+07
sp|P11840|CASK_BUBBU	KYIPIQ	760,45	24	29	1,09E+07
sp|P11840|CASK_BUBBU	KYIPIQY	923,51	24	30	4,12E+07
sp|P11840|CASK_BUBBU	YIPIQY	795,42	25	30	8,09E+07
sp|P11840|CASK_BUBBU	YIPIQYVL	1007,57	25	32	2,24E+07
sp|P11840|CASK_BUBBU	IPIQY	632,35	26	30	3,48E+08
sp|P11840|CASK_BUBBU	IPIQYVL	844,51	26	32	6,49E+07
sp|P11840|CASK_BUBBU	IPIQYVLS	931,54	26	33	5,67E+06
sp|P11840|CASK_BUBBU	IPIQYVLSR	1087,64	26	34	1,54E+07
sp|P11840|CASK_BUBBU	IPIQYVLSRY	1250,70	26	35	1,24E+07
sp|P11840|CASK_BUBBU	IQYVL	634,37	28	32	7,04E+06
sp|P11840|CASK_BUBBU	YVLSRYPSYG	1203,59	30	39	3,44E+06
sp|P11840|CASK_BUBBU	YVLSRYPSYGLN	1430,72	30	41	3,42E+06
sp|P11840|CASK_BUBBU	YVLSRYPSYGLNYYQQKPVA	2408,22	30	49	1,59E+06
sp|P11840|CASK_BUBBU	YVLSRYPSYGLNYYQQKPVALINN	2862,47	30	53	5,01E+06
sp|P11840|CASK_BUBBU	VLSRY	636,36	31	35	5,94E+07
sp|P11840|CASK_BUBBU	VLSRYPSY	983,51	31	38	6,35E+06
sp|P11840|CASK_BUBBU	VLSRYPSYG	1040,53	31	39	5,27E+07
sp|P11840|CASK_BUBBU	VLSRYPSYGL	1153,61	31	40	1,02E+08
sp|P11840|CASK_BUBBU	VLSRYPSYGLN	1267,66	31	41	8,33E+07
sp|P11840|CASK_BUBBU	VLSRYPSYGLNY	1430,72	31	42	3,61E+07
sp|P11840|CASK_BUBBU	VLSRYPSYGLNYY	1593,78	31	43	2,03E+06
sp|P11840|CASK_BUBBU	VLSRYPSYGLNYYQ	1721,84	31	44	2,18E+06
sp|P11840|CASK_BUBBU	VLSRYPSYGLNYYQQK	1977,99	31	46	1,46E+07
sp|P11840|CASK_BUBBU	VLSRYPSYGLNYYQQKPV	2174,12	31	48	1,06E+07
sp|P11840|CASK_BUBBU	VLSRYPSYGLNYYQQKPVA	2245,15	31	49	1,13E+07
sp|P11840|CASK_BUBBU	VLSRYPSYGLNYYQQKPVAL	2358,24	31	50	1,48E+07
sp|P11840|CASK_BUBBU	VLSRYPSYGLNYYQQKPVALIN	2585,36	31	52	7,28E+06
sp|P11840|CASK_BUBBU	VLSRYPSYGLNYYQQKPVALINN	2699,41	31	53	4,27E+07
sp|P11840|CASK_BUBBU	VLSRYPSYGLNYYQQKPVALINNQ	2827,47	31	54	5,41E+06
sp|P11840|CASK_BUBBU	VLSRYPSYGLNYYQQKPVALINNQFLPYPYYAKPA	4138,14	31	65	9,25E+07
sp|P11840|CASK_BUBBU	LSRYPSYG	941,46	32	39	8,25E+06
sp|P11840|CASK_BUBBU	LSRYPSYGL	1054,54	32	40	1,12E+07
sp|P11840|CASK_BUBBU	LSRYPSYGLN	1168,59	32	41	8,80E+06
sp|P11840|CASK_BUBBU	LSRYPSYGLNYYQQKPVALINN	2600,34	32	53	2,97E+06
sp|P11840|CASK_BUBBU	SRYPSYG	828,38	33	39	2,22E+07
sp|P11840|CASK_BUBBU	SRYPSYGL	941,46	33	40	2,69E+07
sp|P11840|CASK_BUBBU	SRYPSYGLN	1055,50	33	41	2,31E+07
sp|P11840|CASK_BUBBU	SRYPSYGLNY	1218,57	33	42	3,93E+07
sp|P11840|CASK_BUBBU	SRYPSYGLNYYQ	1509,69	33	44	4,48E+06
sp|P11840|CASK_BUBBU	SRYPSYGLNYYQQK	1765,84	33	46	6,54E+06
sp|P11840|CASK_BUBBU	SRYPSYGLNYYQQKPVA	2033,00	33	49	7,78E+06
sp|P11840|CASK_BUBBU	SRYPSYGLNYYQQKPVAL	2146,08	33	50	5,00E+06
sp|P11840|CASK_BUBBU	SRYPSYGLNYYQQKPVALINN	2487,25	33	53	1,55E+07

sp|P11840|CASK_BUBBU	SRYPSYGLNYYQQKPVALINNQFLPYPYYAKPA	3925,98	33	65	7,21E+07
sp|P11840|CASK_BUBBU	SRYPSYGLNYYQQKPVALINNQFLPYPYYAKPAAVR	4252,19	33	68	4,21E+06
sp|P11840|CASK_BUBBU	SRYPSYGLNYYQQKPVALINNQFLPYPYYAKPAAVRSPA	4507,31	33	71	4,71E+06
sp|P11840|CASK_BUBBU	RYPSYGL	854,43	34	40	5,18E+06
sp|P11840|CASK_BUBBU	RYPSYGLNY	1131,53	34	42	4,91E+06
sp|P11840|CASK_BUBBU	YPSYG	585,24	35	39	5,61E+06
sp|P11840|CASK_BUBBU	YPSYGL	698,33	35	40	6,90E+06
sp|P11840|CASK_BUBBU	YPSYGLNY	975,43	35	42	1,54E+06
sp|P11840|CASK_BUBBU	YPSYGLNYY	1138,50	35	43	3,39E+06
sp|P11840|CASK_BUBBU	YPSYGLNYYQQK	1522,71	35	46	6,10E+06
sp|P11840|CASK_BUBBU	YPSYGLNYYQQKP	1619,76	35	47	3,02E+06
sp|P11840|CASK_BUBBU	YPSYGLNYYQQKPV	1718,83	35	48	4,46E+06
sp|P11840|CASK_BUBBU	YPSYGLNYYQQKPVA	1789,87	35	49	0,00E+00
sp|P11840|CASK_BUBBU	YPSYGLNYYQQKPVAL	1902,95	35	50	4,01E+06
sp|P11840|CASK_BUBBU	YPSYGLNYYQQKPVALINN	2244,12	35	53	2,86E+06
sp|P11840|CASK_BUBBU	YPSYGLNYYQQKPVALINNQ	2372,18	35	54	0,00E+00
sp|P11840|CASK_BUBBU	YPSYGLNYYQQKPVALINNQFLPYPYYAKPA	3682,85	35	65	2,89E+07
sp|P11840|CASK_BUBBU	PSYGLN	649,31	36	41	8,87E+07
sp|P11840|CASK_BUBBU	PSYGLNY	812,37	36	42	2,00E+07
sp|P11840|CASK_BUBBU	PSYGLNYY	975,43	36	43	9,50E+06
sp|P11840|CASK_BUBBU	PSYGLNYYQ	1103,49	36	44	7,77E+06
sp|P11840|CASK_BUBBU	PSYGLNYYQQ	1231,55	36	45	5,85E+06
sp|P11840|CASK_BUBBU	PSYGLNYYQQK	1359,65	36	46	4,13E+07
sp|P11840|CASK_BUBBU	PSYGLNYYQQKP	1456,70	36	47	1,86E+07
sp|P11840|CASK_BUBBU	PSYGLNYYQQKPV	1555,77	36	48	2,05E+07
sp|P11840|CASK_BUBBU	PSYGLNYYQQKPVA	1626,80	36	49	2,73E+07
sp|P11840|CASK_BUBBU	PSYGLNYYQQKPVAL	1739,89	36	50	8,09E+07
sp|P11840|CASK_BUBBU	PSYGLNYYQQKPVALI	1852,97	36	51	0,00E+00
sp|P11840|CASK_BUBBU	PSYGLNYYQQKPVALIN	1967,02	36	52	1,16E+07
sp|P11840|CASK_BUBBU	PSYGLNYYQQKPVALINN	2081,06	36	53	1,05E+08
sp|P11840|CASK_BUBBU	PSYGLNYYQQKPVALINNQ	2209,12	36	54	3,23E+06
sp|P11840|CASK_BUBBU	PSYGLNYYQQKPVALINNQFLPYPYYAKPA	3519,79	36	65	3,40E+08
sp|P11840|CASK_BUBBU	PSYGLNYYQQKPVALINNQFLPYPYYAKPAAVRSPA	4101,12	36	71	4,66E+07
sp|P11840|CASK_BUBBU	SYGLNYYQQK	1262,59	37	46	4,12E+06
sp|P11840|CASK_BUBBU	SYGLNYYQQKP	1359,65	37	47	7,01E+06
sp|P11840|CASK_BUBBU	SYGLNYYQQKPV	1458,71	37	48	0,00E+00
sp|P11840|CASK_BUBBU	SYGLNYYQQKPVA	1529,75	37	49	0,00E+00
sp|P11840|CASK_BUBBU	SYGLNYYQQKPVALIN	1869,96	37	52	4,39E+06
sp|P11840|CASK_BUBBU	SYGLNYYQQKPVALINN	1984,01	37	53	1,11E+07
sp|P11840|CASK_BUBBU	SYGLNYYQQKPVALINNQ	2112,06	37	54	1,38E+06
sp|P11840|CASK_BUBBU	SYGLNYYQQKPVALINNQFLPYPYYAKPA	3422,73	37	65	5,41E+07
sp|P11840|CASK_BUBBU	YGLNYYQQK	1175,56	38	46	2,16E+07
sp|P11840|CASK_BUBBU	YGLNYYQQKPV	1371,68	38	48	9,80E+06
sp|P11840|CASK_BUBBU	YGLNYYQQKPVA	1442,72	38	49	1,23E+07
sp|P11840|CASK_BUBBU	YGLNYYQQKPVAL	1555,80	38	50	8,02E+06
sp|P11840|CASK_BUBBU	YGLNYYQQKPVALIN	1782,93	38	52	4,36E+06
sp|P11840|CASK_BUBBU	YGLNYYQQKPVALINNQ	2025,03	38	54	2,17E+06
sp|P11840|CASK_BUBBU	YGLNYYQQKPVALINNQFLPYPYYAKPA	3335,70	38	65	1,83E+07
sp|P11840|CASK_BUBBU	GLNYY	628,29	39	43	2,06E+07
sp|P11840|CASK_BUBBU	GLNYYQ	756,34	39	44	2,90E+06
sp|P11840|CASK_BUBBU	GLNYYQQK	1012,50	39	46	2,79E+07
sp|P11840|CASK_BUBBU	GLNYYQQKPV	1208,62	39	48	1,46E+07
sp|P11840|CASK_BUBBU	GLNYYQQKPVA	1279,66	39	49	2,19E+07
sp|P11840|CASK_BUBBU	GLNYYQQKPVAL	1392,74	39	50	1,81E+07
sp|P11840|CASK_BUBBU	GLNYYQQKPVALIN	1619,87	39	52	4,93E+06
sp|P11840|CASK_BUBBU	GLNYYQQKPVALINN	1733,91	39	53	2,65E+07
sp|P11840|CASK_BUBBU	GLNYYQQKPVALINNQ	1861,97	39	54	3,38E+06
sp|P11840|CASK_BUBBU	GLNYYQQKPVALINNQFLPYPYYAKPA	3172,64	39	65	5,76E+07
sp|P11840|CASK_BUBBU	GLNYYQQKPVALINNQFLPYPYYAKPAAVR	3498,85	39	68	5,45E+06
sp|P11840|CASK_BUBBU	LNYYQ	699,32	40	44	3,38E+06
sp|P11840|CASK_BUBBU	LNYYQQK	955,48	40	46	6,17E+06
sp|P11840|CASK_BUBBU	LNYYQQKPVALINN	1676,89	40	53	6,56E+06
sp|P11840|CASK_BUBBU	LNYYQQKPVALINNQ	1804,95	40	54	2,66E+06
sp|P11840|CASK_BUBBU	LNYYQQKPVALINNQFLPYPYYAKPA	3115,62	40	65	4,06E+07
sp|P11840|CASK_BUBBU	NYYQQ	714,30	41	45	2,74E+06
sp|P11840|CASK_BUBBU	NYYQQK	842,39	41	46	6,98E+07
sp|P11840|CASK_BUBBU	NYYQQKPV	1038,51	41	48	1,14E+07
sp|P11840|CASK_BUBBU	NYYQQKPVA	1109,55	41	49	2,10E+07
sp|P11840|CASK_BUBBU	NYYQQKPVAL	1222,63	41	50	9,64E+06
sp|P11840|CASK_BUBBU	NYYQQKPVALIN	1449,76	41	52	9,57E+06
sp|P11840|CASK_BUBBU	NYYQQKPVALINN	1563,80	41	53	4,71E+07
sp|P11840|CASK_BUBBU	NYYQQKPVALINNQ	1691,86	41	54	6,27E+06
sp|P11840|CASK_BUBBU	NYYQQKPVALINNQFLPYPYYAK	2834,44	41	63	4,25E+07
sp|P11840|CASK_BUBBU	NYYQQKPVALINNQFLPYPYYAKP	2931,50	41	64	2,40E+07
sp|P11840|CASK_BUBBU	NYYQQKPVALINNQFLPYPYYAKPA	3002,53	41	65	2,43E+08
sp|P11840|CASK_BUBBU	NYYQQKPVALINNQFLPYPYYAKPAAVRSPA	3583,86	41	71	2,09E+07
sp|P11840|CASK_BUBBU	NYYQQKPVALINNQFLPYPYYAKPAAVRSPAQI	3825,00	41	73	3,81E+06
sp|P11840|CASK_BUBBU	YYQQKP	825,40	42	47	3,24E+08
sp|P11840|CASK_BUBBU	YYQQKPVA	995,51	42	49	1,48E+07
sp|P11840|CASK_BUBBU	YYQQKPVAL	1108,59	42	50	3,37E+07
sp|P11840|CASK_BUBBU	YYQQKPVALIN	1335,72	42	52	1,36E+07
sp|P11840|CASK_BUBBU	YYQQKPVALINN	1449,76	42	53	6,60E+07
sp|P11840|CASK_BUBBU	YYQQKPVALINNQ	1577,82	42	54	1,63E+07
sp|P11840|CASK_BUBBU	YYQQKPVALINNQFLPYPYYAK	2720,40	42	63	2,30E+07
sp|P11840|CASK_BUBBU	YYQQKPVALINNQFLPYPYYAKP	2817,45	42	64	2,99E+07
sp|P11840|CASK_BUBBU	YYQQKPVALINNQFLPYPYYAKPA	2888,49	42	65	2,69E+08
sp|P11840|CASK_BUBBU	YYQQKPVALINNQFLPYPYYAKPAA	2959,53	42	66	4,58E+06
sp|P11840|CASK_BUBBU	YYQQKPVALINNQFLPYPYYAKPAAVR	3214,70	42	68	1,14E+07
sp|P11840|CASK_BUBBU	YYQQKPVALINNQFLPYPYYAKPAAVRSP	3398,78	42	70	2,58E+06
sp|P11840|CASK_BUBBU	YYQQKPVALINNQFLPYPYYAKPAAVRSPAQI	3710,96	42	73	2,41E+06
sp|P11840|CASK_BUBBU	YQQKPVA	832,44	43	49	1,88E+08
sp|P11840|CASK_BUBBU	YQQKPVAL	945,53	43	50	4,85E+07
sp|P11840|CASK_BUBBU	YQQKPVALIN	1172,66	43	52	1,71E+07
sp|P11840|CASK_BUBBU	YQQKPVALINN	1286,70	43	53	8,05E+07
sp|P11840|CASK_BUBBU	YQQKPVALINNQ	1414,76	43	54	5,70E+06
sp|P11840|CASK_BUBBU	YQQKPVALINNQFLPYPYYAK	2557,34	43	63	2,94E+07
sp|P11840|CASK_BUBBU	YQQKPVALINNQFLPYPYYAKP	2654,39	43	64	2,11E+07
sp|P11840|CASK_BUBBU	YQQKPVALINNQFLPYPYYAKPA	2725,43	43	65	2,67E+08

sp|P11840|CASK_BUBBU	YQQKPVALINNQFLPYPYYAKPAA	2796,46	43	66	8,58E+06
sp|P11840|CASK_BUBBU	YQQKPVALINNQFLPYPYYAKPAAV	2895,53	43	67	5,59E+06
sp|P11840|CASK_BUBBU	YQQKPVALINNQFLPYPYYAKPAAVR	3051,63	43	68	8,91E+06
sp|P11840|CASK_BUBBU	YQQKPVALINNQFLPYPYYAKPAAVRSPA	3306,76	43	71	1,50E+07
sp|P11840|CASK_BUBBU	YQQKPVALINNQFLPYPYYAKPAAVRSPAQILQ	3789,04	43	75	1,61E+07
sp|P11840|CASK_BUBBU	YQQKPVALINNQFLPYPYYAKPAAVRSPAQILQW	3975,12	43	76	9,45E+06
sp|P11840|CASK_BUBBU	QQKPV	598,34	44	48	2,60E+06
sp|P11840|CASK_BUBBU	QQKPVAL	782,47	44	50	2,29E+08
sp|P11840|CASK_BUBBU	QQKPVALIN	1009,59	44	52	1,44E+07
sp|P11840|CASK_BUBBU	QQKPVALINN	1123,64	44	53	3,66E+07
sp|P11840|CASK_BUBBU	QQKPVALINNQ	1251,69	44	54	1,03E+07
sp|P11840|CASK_BUBBU	QQKPVALINNQFLPYPYYAK	2394,27	44	63	8,65E+06
sp|P11840|CASK_BUBBU	QQKPVALINNQFLPYPYYAKPA	2562,36	44	65	4,25E+07
sp|P11840|CASK_BUBBU	QQKPVALINNQFLPYPYYAKPAAVR	2888,57	44	68	2,25E+06
sp|P11840|CASK_BUBBU	QQKPVALINNQFLPYPYYAKPAAVRSPAQILQ	3625,98	44	75	4,54E+06
sp|P11840|CASK_BUBBU	QKPVALINNQFLPYPYYAKPA	2434,30	45	65	2,01E+07
sp|P11840|CASK_BUBBU	KPVALINN	867,52	46	53	9,88E+06
sp|P11840|CASK_BUBBU	KPVALINNQ	995,58	46	54	4,47E+06
sp|P11840|CASK_BUBBU	KPVALINNQFLPYPYYAKPA	2306,25	46	65	4,06E+06
sp|P11840|CASK_BUBBU	PVALINNQ	867,48	47	54	6,24E+06
sp|P11840|CASK_BUBBU	PVALINNQFLPYPYYAK	2010,06	47	63	4,95E+07
sp|P11840|CASK_BUBBU	PVALINNQFLPYPYYAKPA	2178,15	47	65	3,14E+08
sp|P11840|CASK_BUBBU	PVALINNQFLPYPYYAKPAAVR	2504,36	47	68	2,72E+07
sp|P11840|CASK_BUBBU	PVALINNQFLPYPYYAKPAAVRSP	2688,44	47	70	7,15E+06
sp|P11840|CASK_BUBBU	PVALINNQFLPYPYYAKPAAVRSPA	2759,48	47	71	2,27E+07
sp|P11840|CASK_BUBBU	VALINNQ	770,43	48	54	1,37E+07
sp|P11840|CASK_BUBBU	VALINNQFLPYPYYAK	1913,01	48	63	2,01E+07
sp|P11840|CASK_BUBBU	VALINNQFLPYPYYAKP	2010,06	48	64	3,55E+07
sp|P11840|CASK_BUBBU	VALINNQFLPYPYYAKPA	2081,10	48	65	1,43E+08
sp|P11840|CASK_BUBBU	VALINNQFLPYPYYAKPAAVR	2407,31	48	68	2,89E+07
sp|P11840|CASK_BUBBU	VALINNQFLPYPYYAKPAAVRSP	2591,39	48	70	1,06E+07
sp|P11840|CASK_BUBBU	VALINNQFLPYPYYAKPAAVRSPA	2662,43	48	71	3,36E+07
sp|P11840|CASK_BUBBU	VALINNQFLPYPYYAKPAAVRSPAQ	2790,49	48	72	3,83E+06
sp|P11840|CASK_BUBBU	ALINN	543,30	49	53	3,23E+07
sp|P11840|CASK_BUBBU	ALINNQFLPYPYYAK	1813,94	49	63	3,97E+07
sp|P11840|CASK_BUBBU	ALINNQFLPYPYYAKPA	1982,03	49	65	1,88E+08
sp|P11840|CASK_BUBBU	ALINNQFLPYPYYAKPAAVR	2308,24	49	68	7,72E+06
sp|P11840|CASK_BUBBU	ALINNQFLPYPYYAKPAAVRSP	2492,32	49	70	3,54E+06
sp|P11840|CASK_BUBBU	ALINNQFLPYPYYAKPAAVRSPA	2563,36	49	71	7,36E+06
sp|P11840|CASK_BUBBU	ALINNQFLPYPYYAKPAAVRSPAQI	2804,50	49	73	4,00E+06
sp|P11840|CASK_BUBBU	LINNQFLPYPYYAK	1742,90	50	63	2,42E+07
sp|P11840|CASK_BUBBU	LINNQFLPYPYYAKP	1839,96	50	64	2,21E+07
sp|P11840|CASK_BUBBU	LINNQFLPYPYYAKPA	1910,99	50	65	1,69E+08
sp|P11840|CASK_BUBBU	LINNQFLPYPYYAKPAA	1982,03	50	66	4,35E+06
sp|P11840|CASK_BUBBU	LINNQFLPYPYYAKPAAVR	2237,20	50	68	1,42E+07
sp|P11840|CASK_BUBBU	LINNQFLPYPYYAKPAAVRSP	2421,28	50	70	6,31E+06
sp|P11840|CASK_BUBBU	LINNQFLPYPYYAKPAAVRSPA	2492,32	50	71	1,69E+07
sp|P11840|CASK_BUBBU	LINNQFLPYPYYAKPAAVRSPAQ	2620,38	50	72	4,35E+06
sp|P11840|CASK_BUBBU	LINNQFLPYPYYAKPAAVRSPAQILQ	2974,61	50	75	2,03E+07
sp|P11840|CASK_BUBBU	LINNQFLPYPYYAKPAAVRSPAQILQW	3160,69	50	76	1,28E+07
sp|P11840|CASK_BUBBU	INNQFLP	844,44	51	57	7,42E+06
sp|P11840|CASK_BUBBU	INNQFLPYPYYAKP	1726,87	51	64	1,44E+08
sp|P11840|CASK_BUBBU	INNQFLPYPYYAKPA	1797,91	51	65	7,64E+08
sp|P11840|CASK_BUBBU	INNQFLPYPYYAKPAA	1868,95	51	66	1,80E+07
sp|P11840|CASK_BUBBU	INNQFLPYPYYAKPAAV	1968,01	51	67	8,13E+06
sp|P11840|CASK_BUBBU	INNQFLPYPYYAKPAAVR	2124,12	51	68	7,76E+07
sp|P11840|CASK_BUBBU	INNQFLPYPYYAKPAAVRS	2211,15	51	69	3,82E+06
sp|P11840|CASK_BUBBU	INNQFLPYPYYAKPAAVRSP	2308,20	51	70	4,30E+07
sp|P11840|CASK_BUBBU	INNQFLPYPYYAKPAAVRSPA	2379,24	51	71	1,39E+08
sp|P11840|CASK_BUBBU	INNQFLPYPYYAKPAAVRSPAQ	2507,30	51	72	5,12E+07
sp|P11840|CASK_BUBBU	INNQFLPYPYYAKPAAVRSPAQI	2620,38	51	73	1,10E+07
sp|P11840|CASK_BUBBU	INNQFLPYPYYAKPAAVRSPAQIL	2733,46	51	74	4,84E+07
sp|P11840|CASK_BUBBU	INNQFLPYPYYAKPAAVRSPAQILQ	2861,52	51	75	1,00E+08
sp|P11840|CASK_BUBBU	NNQFLPYPY	1154,54	52	60	5,63E+06
sp|P11840|CASK_BUBBU	NNQFLPYPYY	1317,60	52	61	6,85E+06
sp|P11840|CASK_BUBBU	NNQFLPYPYYAK	1516,74	52	63	0,00E+00
sp|P11840|CASK_BUBBU	NNQFLPYPYYAKP	1613,79	52	64	2,27E+07
sp|P11840|CASK_BUBBU	NNQFLPYPYYAKPA	1684,82	52	65	4,40E+07
sp|P11840|CASK_BUBBU	NNQFLPYPYYAKPAA	1755,86	52	66	7,77E+05
sp|P11840|CASK_BUBBU	NNQFLPYPYYAKPAAVR	2011,03	52	68	1,39E+06
sp|P11840|CASK_BUBBU	NQFLP	617,32	53	57	1,24E+07
sp|P11840|CASK_BUBBU	NQFLPYPYYA	1274,60	53	62	3,66E+06
sp|P11840|CASK_BUBBU	NQFLPYPYYAK	1402,69	53	63	2,20E+07
sp|P11840|CASK_BUBBU	NQFLPYPYYAKP	1499,74	53	64	1,90E+07
sp|P11840|CASK_BUBBU	NQFLPYPYYAKPA	1570,78	53	65	7,84E+07
sp|P11840|CASK_BUBBU	NQFLPYPYYAKPAA	1641,82	53	66	3,19E+06
sp|P11840|CASK_BUBBU	NQFLPYPYYAKPAAVRSP	2081,07	53	70	1,43E+06
sp|P11840|CASK_BUBBU	NQFLPYPYYAKPAAVRSPA	2152,11	53	71	3,37E+06
sp|P11840|CASK_BUBBU	NQFLPYPYYAKPAAVRSPAQILQ	2634,40	53	75	3,77E+06
sp|P11840|CASK_BUBBU	QFLPYPYYAK	1288,65	54	63	2,15E+07
sp|P11840|CASK_BUBBU	QFLPYPYYAKP	1385,70	54	64	2,26E+07
sp|P11840|CASK_BUBBU	QFLPYPYYAKPA	1456,74	54	65	1,55E+08
sp|P11840|CASK_BUBBU	QFLPYPYYAKPAA	1527,78	54	66	3,95E+06
sp|P11840|CASK_BUBBU	QFLPYPYYAKPAAVR	1782,95	54	68	7,97E+06
sp|P11840|CASK_BUBBU	QFLPYPYYAKPAAVRSP	1967,03	54	70	0,00E+00
sp|P11840|CASK_BUBBU	QFLPYPYYAKPAAVRSPA	2038,07	54	71	1,35E+07
sp|P11840|CASK_BUBBU	QFLPYPYYAKPAAVRSPAQ	2166,13	54	72	1,90E+06
sp|P11840|CASK_BUBBU	QFLPYPYYAKPAAVRSPAQI	2279,21	54	73	5,39E+06
sp|P11840|CASK_BUBBU	QFLPYPYYAKPAAVRSPAQIL	2392,29	54	74	9,53E+06
sp|P11840|CASK_BUBBU	QFLPYPYYAKPAAVRSPAQILQ	2520,35	54	75	1,45E+07
sp|P11840|CASK_BUBBU	FLPYP	635,33	55	59	1,69E+06
sp|P11840|CASK_BUBBU	FLPYPYY	961,46	55	61	1,64E+06
sp|P11840|CASK_BUBBU	FLPYPYYAK	1160,59	55	63	2,79E+07
sp|P11840|CASK_BUBBU	FLPYPYYAKP	1257,64	55	64	1,95E+07
sp|P11840|CASK_BUBBU	FLPYPYYAKPA	1328,68	55	65	9,13E+07
sp|P11840|CASK_BUBBU	FLPYPYYAKPAA	1399,72	55	66	2,89E+06
sp|P11840|CASK_BUBBU	FLPYPYYAKPAAVR	1654,89	55	68	5,13E+06
sp|P11840|CASK_BUBBU	FLPYPYYAKPAAVRSPA	1910,01	55	71	5,70E+06

sp|P11840|CASK_BUBBU	FLPYPYYAKPAAVRSPAQ	2038,07	55	72	2,01E+06
sp|P11840|CASK_BUBBU	FLPYPYYAKPAAVRSPAQIL	2264,24	55	74	3,00E+06
sp|P11840|CASK_BUBBU	FLPYPYYAKPAAVRSPAQILQW	2578,37	55	76	1,36E+07
sp|P11840|CASK_BUBBU	LPYPYY	814,39	56	61	6,59E+06
sp|P11840|CASK_BUBBU	LPYPYYA	885,43	56	62	4,87E+06
sp|P11840|CASK_BUBBU	LPYPYYAK	1013,52	56	63	4,89E+07
sp|P11840|CASK_BUBBU	LPYPYYAKP	1110,58	56	64	7,26E+07
sp|P11840|CASK_BUBBU	LPYPYYAKPA	1181,61	56	65	8,96E+07
sp|P11840|CASK_BUBBU	LPYPYYAKPAA	1252,65	56	66	2,64E+06
sp|P11840|CASK_BUBBU	LPYPYYAKPAAVR	1507,82	56	68	3,35E+06
sp|P11840|CASK_BUBBU	PYPYYAK	900,44	57	63	0,00E+00
sp|P11840|CASK_BUBBU	YPYYA	675,29	58	62	7,95E+06
sp|P11840|CASK_BUBBU	YPYYAK	803,39	58	63	5,92E+05
sp|P11840|CASK_BUBBU	YPYYAKP	900,44	58	64	9,54E+07
sp|P11840|CASK_BUBBU	YPYYAKPA	971,48	58	65	8,37E+07
sp|P11840|CASK_BUBBU	YPYYAKPAA	1042,51	58	66	1,40E+06
sp|P11840|CASK_BUBBU	YPYYAKPAAV	1141,58	58	67	1,99E+06
sp|P11840|CASK_BUBBU	YPYYAKPAAVR	1297,68	58	68	6,95E+05
sp|P11840|CASK_BUBBU	YPYYAKPAAVRSP	1481,77	58	70	3,36E+06
sp|P11840|CASK_BUBBU	YPYYAKPAAVRSPAQIL	1907,03	58	74	5,04E+06
sp|P11840|CASK_BUBBU	YPYYAKPAAVRSPAQILQW	2221,17	58	76	5,30E+06
sp|P11840|CASK_BUBBU	PYYAKPA	808,41	59	65	6,33E+05
sp|P11840|CASK_BUBBU	YYAKP	640,32	60	64	4,78E+06
sp|P11840|CASK_BUBBU	AKPAAVRSPA	966,56	62	71	2,05E+06
sp|P11840|CASK_BUBBU	AKPAAVRSPAQILQ	1448,85	62	75	0,00E+00
sp|P11840|CASK_BUBBU	PAAVRSPA	767,43	64	71	8,87E+07
sp|P11840|CASK_BUBBU	PAAVRSPAQ	895,49	64	72	2,12E+07
sp|P11840|CASK_BUBBU	PAAVRSPAQI	1008,57	64	73	6,13E+06
sp|P11840|CASK_BUBBU	PAAVRSPAQIL	1121,66	64	74	2,27E+07
sp|P11840|CASK_BUBBU	PAAVRSPAQILQ	1249,71	64	75	5,47E+07
sp|P11840|CASK_BUBBU	PAAVRSPAQILQW	1435,79	64	76	4,84E+07
sp|P11840|CASK_BUBBU	PAAVRSPAQILQWQ	1563,85	64	77	1,56E+06
sp|P11840|CASK_BUBBU	PAAVRSPAQILQWQV	1662,92	64	78	3,13E+06
sp|P11840|CASK_BUBBU	PAAVRSPAQILQWQVLPN	1987,10	64	81	1,50E+07
sp|P11840|CASK_BUBBU	PAAVRSPAQILQWQVLPNTVPAK	2483,40	64	86	1,07E+07
sp|P11840|CASK_BUBBU	AAVRSP	599,34	65	70	1,44E+06
sp|P11840|CASK_BUBBU	AAVRSPAQIL	1024,60	65	74	5,15E+06
sp|P11840|CASK_BUBBU	AAVRSPAQILQ	1152,66	65	75	4,38E+06
sp|P11840|CASK_BUBBU	AAVRSPAQILQW	1338,74	65	76	3,26E+06
sp|P11840|CASK_BUBBU	AVRSPAQIL	953,57	66	74	5,39E+07
sp|P11840|CASK_BUBBU	AVRSPAQILQ	1081,62	66	75	2,10E+06
sp|P11840|CASK_BUBBU	AVRSPAQILQW	1267,70	66	76	5,49E+07
sp|P11840|CASK_BUBBU	AVRSPAQILQWQV	1494,83	66	78	1,54E+06
sp|P11840|CASK_BUBBU	VRSPAQIL	882,53	67	74	2,20E+07
sp|P11840|CASK_BUBBU	VRSPAQILQ	1010,59	67	75	4,94E+07
sp|P11840|CASK_BUBBU	VRSPAQILQW	1196,67	67	76	3,97E+07
sp|P11840|CASK_BUBBU	VRSPAQILQWQ	1324,73	67	77	4,37E+05
sp|P11840|CASK_BUBBU	VRSPAQILQWQVLPN	1747,97	67	81	3,03E+07
sp|P11840|CASK_BUBBU	VRSPAQILQWQVLPNTVPAK	2244,27	67	86	8,52E+06
sp|P11840|CASK_BUBBU	RSPAQIL	783,46	68	74	1,43E+07
sp|P11840|CASK_BUBBU	RSPAQILQ	911,52	68	75	1,61E+06
sp|P11840|CASK_BUBBU	SPAQILQ	755,42	69	75	7,97E+07
sp|P11840|CASK_BUBBU	SPAQILQW	941,50	69	76	3,63E+07
sp|P11840|CASK_BUBBU	SPAQILQWQ	1069,56	69	77	4,44E+06
sp|P11840|CASK_BUBBU	SPAQILQWQVL	1281,71	69	79	3,63E+06
sp|P11840|CASK_BUBBU	SPAQILQWQVLPNTVPAK	1989,10	69	86	2,41E+06
sp|P11840|CASK_BUBBU	AQILQW	757,41	71	76	6,15E+06
sp|P11840|CASK_BUBBU	AQILQWQVLPN	1308,72	71	81	2,85E+06
sp|P11840|CASK_BUBBU	AQILQWQVLPNTVPAK	1805,02	71	86	1,05E+06
sp|P11840|CASK_BUBBU	QILQWQVLPN	1237,68	72	81	4,41E+06
sp|P11840|CASK_BUBBU	ILQWQVLP	995,58	73	80	4,89E+06
sp|P11840|CASK_BUBBU	ILQWQVLPN	1109,62	73	81	1,18E+07
sp|P11840|CASK_BUBBU	QWQVL	672,36	75	79	4,41E+05
sp|P11840|CASK_BUBBU	QWQVLPN	883,46	75	81	3,45E+06
sp|P11840|CASK_BUBBU	QWQVLPNTVPAKS	1466,79	75	87	1,44E+06
sp|P11840|CASK_BUBBU	WQVLPN	755,40	76	81	0,00E+00
sp|P11840|CASK_BUBBU	QVLSNTVPAK	1055,60	77	86	1,29E+06
sp|P11840|CASK_BUBBU	QVLPNTVPAK	1065,62	77	86	6,74E+07
sp|P11840|CASK_BUBBU	QVLPNTVPAKS	1152,65	77	87	1,33E+07
sp|P11840|CASK_BUBBU	VLPNTVPA	809,46	78	85	5,50E+07
sp|P11840|CASK_BUBBU	VLPNTVPAK	937,56	78	86	1,43E+08
sp|P11840|CASK_BUBBU	VLPNTVPAKS	1024,59	78	87	3,86E+07
sp|P11840|CASK_BUBBU	LPNTVPAK	838,49	79	86	4,97E+06
sp|P11840|CASK_BUBBU	LPNTVPAKS	925,52	79	87	3,59E+07
sp|P11840|CASK_BUBBU	AQPTTMT	748,34	90	96	5,12E+06
sp|P11840|CASK_BUBBU	QDKTEIP	829,42	114	120	0,00E+00
sp|P11840|CASK_BUBBU	QDKTEIPT	930,47	114	121	0,00E+00
sp|P11840|CASK_BUBBU	QDKTEIPTIN	1157,59	114	123	0,00E+00
sp|P11840|CASK_BUBBU	QDKTEIPTINT	1258,64	114	124	4,97E+06
sp|P11840|CASK_BUBBU	DKTEIPT	802,41	115	121	0,00E+00
sp|P11840|CASK_BUBBU	DKTEIPTIN	1029,53	115	123	2,95E+06
sp|P11840|CASK_BUBBU	DKTEIPTINT	1130,58	115	124	4,90E+06
sp|P11840|CASK_BUBBU	EASSEVIESVPETN	1489,68	147	160	0,00E+00
sp|P11840|CASK_BUBBU	IESVPETN	887,42	153	160	2,11E+06
sp|P11840|CASK_BUBBU	SVPETNT	746,34	155	161	1,41E+06
sp|P02755|LACB_BUBBU	IIVTQTMKGLDIQKVAGTWYS	2351,26	1	21	1,50E+07
sp|P02755|LACB_BUBBU	IIVTQTMKGLDIQKVAGTW	2101,16	1	19	1,35E+08
sp|P02754|LACA_BOVIN;sp|P02754|LACB_BOVIN	LIVTQTMKGLDIQK	1586,91	1	14	5,14E+06
sp|P02755|LACB_BUBBU	IIVTQTMKGLDIQ	1458,81	1	13	2,61E+07
sp|P02754|LACA_BOVIN;sp|P02754|LACB_BOVIN	LIVTQTMKGL	1102,64	1	10	8,68E+07
sp|P02755|LACB_BUBBU	IIVTQTMK	932,54	1	8	4,40E+06
sp|P02754|LACA_BOVIN;sp|P02754|LACB_BOVIN	LIVTQT	673,40	1	6	5,16E+06
sp|P02754|LACA_BOVIN;sp|P02754|LACB_BOVIN	LIVTQ	572,35	1	5	1,24E+07
sp|P02754|LACA_BOVIN;sp|P02754|LACB_BOVIN	IVTQTMKGLDIQKVAGTWYS	2238,17	2	21	8,83E+06
sp|P02755|LACB_BUBBU	IVTQTMKGLDIQKVAGTW	1988,08	2	19	3,85E+06
sp|P02755|LACB_BUBBU	IVTQTMKGLDIQ	1345,73	2	13	1,33E+08
sp|P02755|LACB_BUBBU	IVTQTMKGLD	1104,58	2	11	8,37E+06
sp|P02755|LACB_BUBBU	IVTQTMKGL	989,56	2	10	0,00E+00

sp|P02755|LACB_BUBBU	IVTQTMKG	876,47	2	9	2,18E+07
sp|P02754|LACA_BOVIN;sp|P02754|LACB_BOVIN	IVTQTMK	819,45	2	8	2,31E+07
sp|P02755|LACB_BUBBU	VTQTMKGL	876,47	3	10	8,49E+06
sp|P02754|LACA_BOVIN;sp|P02754|LACB_BOVIN	TQTMKGLDIQKVAGTW	1775,92	4	19	1,19E+07
sp|P02755|LACB_BUBBU	TQTMKGLDIQ	1133,58	4	13	5,09E+06
sp|P02754|LACA_BOVIN;sp|P02754|LACB_BOVIN	QTMKGLDIQKVAGTW	1674,88	5	19	7,60E+06
sp|P02755|LACB_BUBBU	TMKGLDIQKVAGTW	1546,82	6	19	3,86E+06
sp|P02755|LACB_BUBBU	TMKGLDIQ	904,47	6	13	8,91E+06
sp|P02754|LACA_BOVIN;sp|P02754|LACB_BOVIN	KGLDIQKVAGTW	1314,73	8	19	1,93E+07
sp|P02755|LACB_BUBBU	KGLDIQKVA	970,58	8	16	2,04E+06
sp|P02754|LACA_BOVIN;sp|P02754|LACB_BOVIN	KGLDIQK	800,48	8	14	2,84E+07
sp|P02754|LACA_BOVIN;sp|P02754|LACB_BOVIN	KGLDIQ	672,38	8	13	1,55E+07
sp|P02755|LACB_BUBBU	GLDIQKVAGTW	1186,63	9	19	2,00E+07
sp|P02754|LACA_BOVIN;sp|P02754|LACB_BOVIN	LDIQKVAGTW	1129,61	10	19	8,01E+06
sp|P02755|LACB_BUBBU	LDIQKVA	785,46	10	16	1,79E+06
sp|P02754|LACA_BOVIN;sp|P02754|LACB_BOVIN	DIQKVAGTWYSLA	1450,75	11	23	3,55E+06
sp|P02754|LACA_BOVIN;sp|P02754|LACB_BOVIN	DIQKVAGTWYSL	1379,71	11	22	1,73E+07
sp|P02755|LACB_BUBBU	DIQKVAGTWY	1179,59	11	20	8,51E+06
sp|P02755|LACB_BUBBU	DIQKVAGTW	1016,53	11	19	2,81E+07
sp|P02754|LACA_BOVIN;sp|P02754|LACB_BOVIN	DIQKVAGT	830,45	11	18	8,87E+06
sp|P02755|LACB_BUBBU	IQKVAGTW	901,50	12	19	8,87E+06
sp|P02755|LACB_BUBBU	KVAGTWYSL	1023,54	14	22	8,74E+06
sp|P02755|LACB_BUBBU	KVAGTW	660,36	14	19	2,38E+06
sp|P02754|LACA_BOVIN;sp|P02754|LACB_BOVIN	VAGTWYSL	895,44	15	22	1,34E+06
sp|P02754|LACA_BOVIN;sp|P02754|LACB_BOVIN	SLAMAASDISLLDAQSAPLR	2029,05	21	40	8,29E+06
sp|P02755|LACB_BUBBU	SLAMAASDISL	1077,54	21	31	1,82E+06
sp|P02755|LACB_BUBBU	AMAASDISLLDAQSAPLRVY	2091,07	23	42	1,97E+07
sp|P02755|LACB_BUBBU	AMAASDISLLDAQSAPLR	1828,94	23	40	4,39E+06
sp|P02755|LACB_BUBBU	AMAASDISLLDAQ	1304,63	23	35	3,64E+06
sp|P02755|LACB_BUBBU	AMAASDISLL	990,51	23	32	1,73E+06
sp|P02755|LACB_BUBBU	MAASDISLLDAQSAPLR	1757,90	24	40	1,47E+06
sp|P02755|LACB_BUBBU	AASDISLLDAQSAPLRVY	1888,99	25	42	1,74E+07
sp|P02755|LACB_BUBBU	AASDISLLDAQSAPLRV	1725,93	25	41	3,06E+06
sp|P02755|LACB_BUBBU	AASDISLLDAQSAPLR	1626,86	25	40	6,88E+06
sp|P02755|LACB_BUBBU	AASDISLLDAQSAPL	1470,76	25	39	1,40E+07
sp|P02754|LACA_BOVIN;sp|P02754|LACB_BOVIN	AASDISLLDAQSAP	1357,67	25	38	0,00E+00
sp|P02754|LACA_BOVIN;sp|P02754|LACB_BOVIN	AASDISLLDAQ	1102,55	25	35	1,68E+07
sp|P02755|LACB_BUBBU	ASDISLLDAQSAPLRVY	1817,95	26	42	6,94E+06
sp|P02754|LACA_BOVIN;sp|P02754|LACB_BOVIN	ASDISLLDAQSAPLR	1555,82	26	40	1,51E+07
sp|P02755|LACB_BUBBU	ASDISLLDAQSAPL	1399,72	26	39	1,63E+07
sp|P02755|LACB_BUBBU	SDISLLDAQSAPLRV	1583,85	27	41	1,64E+06
sp|P02754|LACA_BOVIN;sp|P02754|LACB_BOVIN	SDISLLDAQSAPLR	1484,78	27	40	6,86E+06
sp|P02755|LACB_BUBBU	SDISLLDAQSAPL	1328,68	27	39	7,95E+06
sp|P02754|LACA_BOVIN;sp|P02754|LACB_BOVIN	SDISLLD	761,38	27	33	1,90E+06
sp|P02755|LACB_BUBBU	DISLLDAQSAPLR	1397,75	28	40	2,94E+06
sp|P02755|LACB_BUBBU	DISLLDAQSAPL	1241,65	28	39	9,65E+06
sp|P02755|LACB_BUBBU	ISLLDAQSAPLR	1282,72	29	40	0,00E+00
sp|P02754|LACA_BOVIN;sp|P02754|LACB_BOVIN	SLLDAQSAPLR	1169,64	30	40	9,64E+05
sp|P02755|LACB_BUBBU	LDAQSAPLRVYVE	1459,77	32	44	3,17E+06
sp|P02754|LACA_BOVIN;sp|P02754|LACB_BOVIN	LDAQSAPLRVY	1231,66	32	42	1,09E+07
sp|P02755|LACB_BUBBU	LDAQSAPLR	969,52	32	40	3,14E+06
sp|P02755|LACB_BUBBU	DAQSAPLRVYVE	1346,68	33	44	3,66E+06
sp|P02754|LACA_BOVIN;sp|P02754|LACB_BOVIN	DAQSAPLRVY	1118,57	33	42	2,92E+07
sp|P02755|LACB_BUBBU	AQSAPLRVY	1003,55	34	42	4,31E+06
sp|P02754|LACA_BOVIN;sp|P02754|LACB_BOVIN	QSAPL	514,28	35	39	1,61E+07
sp|P02755|LACB_BUBBU	SAPLRVYVE	1032,56	36	44	3,26E+06
sp|P02754|LACA_BOVIN;sp|P02754|LACB_BOVIN	SAPLRVY	804,45	36	42	1,49E+07
sp|P02755|LACB_BUBBU	APLRVYVE	945,53	37	44	0,00E+00
sp|P02754|LACA_BOVIN;sp|P02754|LACB_BOVIN	RVYVEELKPTPEGDLEIL	2099,12	40	57	8,80E+06
sp|P02755|LACB_BUBBU	RVYVEELKPTPEGDLEI	1986,03	40	56	2,17E+06
sp|P02754|LACA_BOVIN;sp|P02754|LACB_BOVIN	RVYVEELKPTPEG	1515,79	40	52	9,80E+05
sp|P02755|LACB_BUBBU	VYVEELKPTPEGDLEIL	1943,01	41	57	1,77E+07
sp|P02755|LACB_BUBBU	VYVEELKPTPEGDLEI	1829,93	41	56	7,02E+06
sp|P02754|LACA_BOVIN;sp|P02754|LACB_BOVIN	VYVEELKPTPEGDLE	1716,85	41	55	1,90E+06
sp|P02755|LACB_BUBBU	VYVEELKPTPEGDL	1587,80	41	54	9,56E+06
sp|P02755|LACB_BUBBU	VYVEELKPTPEG	1359,69	41	52	1,93E+06
sp|P02755|LACB_BUBBU	YVEELKPTPEGDLEIL	1843,95	42	57	2,07E+07
sp|P02755|LACB_BUBBU	YVEELKPTPEGDLEI	1730,86	42	56	9,60E+06
sp|P02754|LACA_BOVIN;sp|P02754|LACB_BOVIN	YVEELKPTPEGDLE	1617,78	42	55	2,76E+06
sp|P02755|LACB_BUBBU	YVEELKPTPEGDL	1488,73	42	54	2,22E+07
sp|P02755|LACB_BUBBU	YVEELKPTPEG	1260,62	42	52	6,28E+06
sp|P02754|LACA_BOVIN;sp|P02754|LACB_BOVIN	YVEELKPTPE	1203,60	42	51	3,20E+06
sp|P02754|LACA_BOVIN;sp|P02754|LACB_BOVIN	VEELKPTPEGDLEILL	1793,97	43	58	2,20E+07
sp|P02755|LACB_BUBBU	VEELKPTPEGDLEIL	1680,88	43	57	5,14E+07
sp|P02755|LACB_BUBBU	VEELKPTPEGDLE	1454,71	43	55	3,78E+06
sp|P02755|LACB_BUBBU	VEELKPTPEGDL	1325,67	43	54	3,56E+07
sp|P02755|LACB_BUBBU	EELKPTPEGDLEIL	1581,81	44	57	1,98E+07
sp|P02755|LACB_BUBBU	EELKPTPEGDLEI	1468,73	44	56	6,57E+06
sp|P02755|LACB_BUBBU	EELKPTPEG	998,49	44	52	4,92E+06
sp|P02755|LACB_BUBBU	ELKPTPEGDLEILL	1565,86	45	58	1,91E+07
sp|P02755|LACB_BUBBU	ELKPTPEGDLEIL	1452,77	45	57	4,42E+07
sp|P02755|LACB_BUBBU	ELKPTPEGDLEI	1339,69	45	56	1,76E+07
sp|P02755|LACB_BUBBU	KPTPEGDLEILL	1323,73	47	58	2,29E+07
sp|P02755|LACB_BUBBU	KPTPEGDLEIL	1210,64	47	57	2,72E+07
sp|P02755|LACB_BUBBU	KPTPEGDLEI	1097,56	47	56	1,13E+07
sp|P02755|LACB_BUBBU	KPTPEGDLE	984,48	47	55	2,15E+06
sp|P02755|LACB_BUBBU	TPEGDLEIL	985,50	49	57	4,91E+06
sp|P02754|LACA_BOVIN;sp|P02754|LACB_BOVIN	TPEGDLEI	872,41	49	56	7,19E+06
sp|P02755|LACB_BUBBU	TPEGDL	630,29	49	54	2,88E+06
sp|P02755|LACB_BUBBU	TKIPAVFKID	1130,67	76	85	1,24E+07
sp|P02754|LACA_BOVIN;sp|P02754|LACB_BOVIN	TKIPAVF	774,46	76	82	3,09E+06
sp|P02755|LACB_BUBBU	IPAVFKID	901,53	78	85	7,52E+06
sp|P02754|LACA_BOVIN;sp|P02754|LACB_BOVIN	KIDALNENKVLVL	1467,87	83	95	4,90E+06
sp|P02755|LACB_BUBBU	KIDALNENK	1043,56	83	91	1,35E+07
sp|P02755|LACB_BUBBU	IDALNENKVLVL	1339,77	84	95	1,11E+07
sp|P02755|LACB_BUBBU	IDALNENK	915,47	84	91	7,18E+06
sp|P02754|LACA_BOVIN;sp|P02754|LACB_BOVIN	DALNENKVLVL	1226,69	85	95	6,14E+06
sp|P02754|LACA_BOVIN;sp|P02754|LACB_BOVIN	DALNENKVL	1014,53	85	93	3,44E+06

sp|P02754|LACA_BOVIN;sp|P02754|LACB_BOVIN	ALNENKVLVLDTDYKK	1862,01	86	101	2,37E+06
sp|P02755|LACB_BUBBU	ALNENKVLVL	1111,66	86	95	4,42E+07
sp|P02755|LACB_BUBBU	ALNENKVLV	998,58	86	94	7,68E+06
sp|P02755|LACB_BUBBU	ALNENKVL	899,51	86	93	4,41E+07
sp|P02755|LACB_BUBBU	ALNENKV	786,42	86	92	8,24E+06
sp|P02754|LACA_BOVIN;sp|P02754|LACB_BOVIN	LNENKVLVL	1040,62	87	95	1,20E+07
sp|P02755|LACB_BUBBU	NENKVLVL	927,54	88	95	0,00E+00
sp|P02755|LACB_BUBBU	ENKVLVL	813,50	89	95	3,32E+06
sp|P02754|LACA_BOVIN;sp|P02754|LACB_BOVIN	VLVLDTDYKKY	1355,73	92	102	2,53E+06
sp|P02755|LACB_BUBBU	VLDTDYKKY	1143,58	94	102	4,58E+06
sp|P02755|LACB_BUBBU	VLDTDYKK	980,52	94	101	4,70E+06
sp|P02754|LACA_BOVIN;sp|P02754|LACB_BOVIN	TDYKKY	816,40	97	102	5,63E+06
sp|P02754|LACA_BOVIN;sp|P02754|LACB_BOVIN	DKALKALPMHI	1235,71	137	147	1,75E+06
sp|P02755|LACB_BUBBU	DKALKALP	854,52	137	144	5,73E+06
sp|P02755|LACB_BUBBU	ALPMHIR	836,47	142	148	6,56E+05
sp|P02754|LACA_BOVIN;sp|P02754|LACB_BOVIN	PMHIR	652,35	144	148	1,26E+06
sp|P02755|LACB_BUBBU	NPTQLEEQ	957,44	152	159	2,12E+06
sp|P02755|LACB_BUBBU	NPTQLEE	829,38	152	158	6,58E+05

Supplementary Material Table S6. Complete list of peptides identified by nano-LC-ESI-MS/MS in the tryptic digest of pH 4.6 water-insoluble fraction extracted from a 65-day-old buffalo blue cheese. This sample was investigated for the occurrence of putative adulteration with bovine milk. Information reported includes protein accession code, peptide sequence, experimental mass value, localization within the protein sequence and intensity value. This analysis enabled quantification of adulteration with bovine milk based on signal intensity ratio of proteotypic β-casein A1 peptides relative to the corresponding buffalo counterparts.

Protein	Sequence	Mass	Start	End	Intensity
sp|P02662|CASA1_BOVIN	HQGLPQEVLNENLLR	1758,94	8	22	4,00E+06
sp|P02662|CASA1_BOVIN	EKVNELSK	945,51	35	42	6,04E+06
sp|P02662|CASA1_BOVIN	YKVPQLEIVPNSAEER	1870,98	104	119	7,01E+05
sp|P02662|CASA1_BOVIN	VPQLEIVPNSAEER	1579,82	106	119	0,00E+00
tr|Q4F6X6|Q4F6X6_BUBBU	QPIKHQGLPQGVLNENLLR	2153,21	4	22	1,41E+07
tr|Q4F6X6|Q4F6X6_BUBBU	HQGLPQGVLNENLLR	1686,92	8	22	1,47E+07
tr|Q4F6X6|Q4F6X6_BUBBU	FFVAPFPEVFGK	1383,72	23	34	1,49E+09
tr|Q4F6X6|Q4F6X6_BUBBU	HIQKEDVPSERYLGYLEQLLRLK	2826,54	80	102	7,85E+06
tr|Q4F6X6|Q4F6X6_BUBBU	HIQKEDVPSERYLGYLEQLLR	2585,36	80	100	1,70E+08
tr|Q4F6X6|Q4F6X6_BUBBU	HIQKEDVPSER	1336,67	80	90	6,72E+09
tr|Q4F6X6|Q4F6X6_BUBBU	EDVPSERYLGYLEQLLR	2079,06	84	100	2,84E+06
tr|Q4F6X6|Q4F6X6_BUBBU	EDVPSER	830,38	84	90	2,19E+06
tr|Q4F6X6|Q4F6X6_BUBBU	YLGYLEQLLRLK	1507,88	91	102	2,19E+07
tr|Q4F6X6|Q4F6X6_BUBBU	YLGYLEQLLR	1266,70	91	100	1,95E+10
tr|Q4F6X6|Q4F6X6_BUBBU	KYNVPQLEIVPNLAEEQLHSMK	2579,34	103	124	1,28E+09
tr|Q4F6X6|Q4F6X6_BUBBU	YNVPQLEIVPNLAEEQLHSMKEGIHAQQK	3342,70	104	132	3,53E+06
tr|Q4F6X6|Q4F6X6_BUBBU	YNVPQLEIVPNLAEEQLHSMK	2451,25	104	124	2,86E+09
tr|Q4F6X6|Q4F6X6_BUBBU	EGIHAQQKEPMIGVNQELAYFYPQLFR	3205,60	125	151	1,87E+09
tr|Q4F6X6|Q4F6X6_BUBBU	EGIHAQQK	909,47	125	132	3,76E+06
tr|Q4F6X6|Q4F6X6_BUBBU	EPMIGVNQELAYFYPQLFR	2314,15	133	151	5,34E+09
tr|Q4F6X6|Q4F6X6_BUBBU	TTMPLW	747,36	194	199	8,47E+06
sp|P02663|CASA2_BOVIN	NMAINPSK	873,44	22	32	3,39E+06
sp|P02663|CASA2_BOVIN	TKLTEEEKNR	1246,65	25	32	3,73E+07
sp|P02663|CASA2_BOVIN	LTEEEKNR	1017,51	25	32	5,95E+06
sp|P02663|CASA2_BOVIN	TVYQHQK	902,46	71	80	3,26E+07
sp|P02663|CASA2_BOVIN	LNFLKKISQR	1245,76	71	76	1,51E+08
tr|A0A7T6ZLP5|A0A7T6ZLP5_BUBBU	FAWPQYLK	1051,55	81	114	2,26E+07
tr|B6VPY3|B6VPY3_BUBBU	TVDMESTEVFTKK	1513,73	81	113	2,72E+07
tr|B6VPY3|B6VPY3_BUBBU	TVDMESTEVFTK	1385,64	81	91	8,60E+07
tr|O62825|O62825_BUBBU	QEKNMAIHPSK	1281,65	92	114	2,19E+07
tr|O62825|O62825_BUBBU	NMAIHPSK	896,45	92	113	2,76E+07
tr|O62825|O62825_BUBBU	ITVDDKHYQK	1245,64	114	125	1,13E+07
tr|O62825|O62825_BUBBU	ITVDDK	689,36	115	137	1,33E+08
tr|O62825|O62825_BUBBU	ALNEINQFYQKFPQYLQYLYQGPIVLNPWDQVKR	4213,18	115	136	9,20E+05
tr|O62825|O62825_BUBBU	ALNEINQFYQKFPQYLQYLYQGPIVLNPWDQVK	4057,08	115	125	5,82E+08
tr|O62825|O62825_BUBBU	ALNEINQFYQK	1366,69	126	137	2,46E+08
tr|O62825|O62825_BUBBU	FPQYLQYLYQGPIVLNPWDQVKR	2864,50	126	136	2,26E+08
tr|O62825|O62825_BUBBU	FPQYLQYLYQGPIVLNPWDQVK	2708,40	137	150	2,81E+07
tr|O62825|O62825_BUBBU	RNAVPITPTLNR	1350,77	137	149	1,84E+07
tr|O62825|O62825_BUBBU	NAVPITPTLNREQLSTSEENSKK	2555,32	138	150	3,37E+07
tr|O62825|O62825_BUBBU	NAVPITPTLNREQLSTSEENSK	2427,22	138	150	1,54E+08
tr|O62825|O62825_BUBBU	NAVPITPTLNR	1194,67	138	149	1,58E+07
tr|O62825|O62825_BUBBU	EQLSTSEENSKK	1378,66	138	149	5,24E+07
tr|O62825|O62825_BUBBU	EQLSTSEENSK	1250,56	151	160	1,96E+06
tr|O62825|O62825_BUBBU	KTVDMESTEVITKK	1607,84	151	160	1,32E+06
tr|O62825|O62825_BUBBU	KTVDMESTEVITK	1479,75	151	158	5,25E+06
tr|O62825|O62825_BUBBU	TVDMESTEVITKK	1479,75	153	165	4,85E+08
tr|O62825|O62825_BUBBU	TVDMESTEVITK	1351,65	153	160	4,57E+05
tr|O62825|O62825_BUBBU	TKLTEEDKNR	1232,64	153	160	1,09E+07
tr|O62825|O62825_BUBBU	TKLTEEDK	962,49	161	170	0,00E+00
tr|O62825|O62825_BUBBU	LTEEDKNRLNFLK	1618,87	161	166	1,24E+07
tr|O62825|O62825_BUBBU	LTEEDKNR	1003,49	167	173	4,47E+06
tr|O62825|O62825_BUBBU	LNFLKK	761,48	174	181	3,80E+08
tr|O62825|O62825_BUBBU	ISQHYQK	902,46	174	181	4,17E+06
tr|O62825|O62825_BUBBU	FTWPQYLK	1081,56	182	188	6,68E+06
tr|O62825|O62825_BUBBU	TVYQYQK	928,47	182	188	1,40E+08
tr|O62825|O62825_BUBBU	AMKPWTQPK	1085,57	189	197	3,71E+07
tr|O62825|O62825_BUBBU	TNVIPYVR	960,54	198	205	4,11E+09
tr|B7VGH4|B7VGH4_BUBBU	FQSEEQQQMEDELQDK	2010,85	33	48	7,04E+05
tr|V9PW58|V9PW58_BUBBU	DELQDKIHPFAQTQSLVYPFPGPIPK	2964,54	43	68	6,36E+06
tr|B7VGH4|B7VGH4_BUBBU	IHPFAQTQSLVYPFPGPIPK	2236,20	49	68	3,10E+06
tr|A0N0S5|A0N0S5_BUBBU	QTQSLVYPFPGPIPK	1670,90	54	68	9,96E+07
tr|A0A0D4C3U8|A0A0D4C3U8_BUBBU	KSLPQNIPPLTQTPVVVPPFLQPEIMGVSK	3253,81	68	97	7,83E+05
tr|B7VGH4|B7VGH4_BUBBU	SLPQNIPPLTQTPVVVPPFLQPEIMGVSKVKEAMAPK	3980,19	69	105	3,01E+07
tr|B7VGH4|B7VGH4_BUBBU	SLPQNIPPLTQTPVVVPPFLQPEIMGVSKVK	3352,88	69	99	2,31E+07
tr|B7VGH4|B7VGH4_BUBBU	SLPQNIPPLTQTPVVVPPFLQPEIMGVSK	3125,72	69	97	8,63E+07
tr|B7VGH4|B7VGH4_BUBBU	VKEAMAPK	872,48	98	105	1,31E+09
tr|B7VGH4|B7VGH4_BUBBU	EAMAPK	645,32	100	105	4,47E+08
tr|B7VGH4|B7VGH4_BUBBU	HKEMPFPK	1012,52	106	113	5,20E+07
tr|B7VGH4|B7VGH4_BUBBU	EMPFPK	747,36	108	113	7,63E+07
tr|A0A0D3RIW8|A0A0D3RIW8_BUBBU	NLHLPLPLLQSWMHQPPQPLPPTVMFPPQSVLSLSQSK	4282,28	132	169	2,57E+07
tr|B7VGH4|B7VGH4_BUBBU	VLPVPQKAVPYPQRDMPIQAFLLYQEPVLGPVRGPFPIIV	4481,51	170	209	5,26E+07
tr|V9PW58|V9PW58_BUBBU	VLPVPQKAVPYPQRDMPIQAFLLYQEPVLGPVRGPFPII	4382,44	170	208	3,61E+06
tr|B7VGH4|B7VGH4_BUBBU	VLPVPQKAVPYPQRDMPIQAFLLYQEPVLGPVR	3758,07	170	202	0,00E+00
tr|B7VGH4|B7VGH4_BUBBU	VLPVPQKAVPYPQR	1590,92	170	183	1,12E+10
tr|B7VGH4|B7VGH4_BUBBU	VLPVPQK	779,49	170	176	1,72E+08
tr|B7VGH4|B7VGH4_BUBBU	AVPYPQRDMPIQAFLLYQEPVLGPVRGPFPIIV	3720,03	177	209	2,02E+07
tr|B7VGH4|B7VGH4_BUBBU	AVPYPQRDMPIQAFLLYQEPVLGPVR	2996,59	177	202	8,71E+09
tr|B7VGH4|B7VGH4_BUBBU	AVPYPQR	829,44	177	183	8,41E+08
tr|B7VGH4|B7VGH4_BUBBU	DMPIQAFLLYQEPVLGPVRGPFPIIV	2908,59	184	209	4,24E+09
tr|V9PW58|V9PW58_BUBBU	DMPIQAFLLYQEPVLGPVRGPFPII	2809,52	184	209	1,38E+07
tr|A0A0D3RIW8|A0A0D3RIW8_BUBBU	DMPIQAFLLYQEPVLGPVRGPFPI	2696,44	184	207	1,70E+08
tr|B7VGH4|B7VGH4_BUBBU	DMPIQAFLLYQEPVLGPVR	2185,16	184	202	1,95E+06
tr|B7VGH4|B7VGH4_BUBBU	GPFPIIV	741,44	203	209	1,22E+08

tr|V9PW58|V9PW58_BUBBU	GPFPII	642,37	203	208	1,61E+07
sp|P02668| CASK_BOVIN	QEQNQEQPIR	1268,61	1	10	8,96E+08
sp|P02668| CASK_BOVIN	SPAQILQWQVLSNTVPAK	1979,08	69	86	3,38E+08
sp|P02668| CASK_BOVIN	SCQAQPTTMAR	1192,53	87	97	5,46E+05
tr|Q712N6|Q712N6_BUBBU	FFNDKIAK	981,53	17	24	7,00E+07
tr|Q712N6|Q712N6_BUBBU	YIPIQYVLSR	1250,70	25	34	8,12E+08
tr|Q712N6|Q712N6_BUBBU	YPSYGLNYYQQKPVALINNQFLPYPYYAKPAAVR	4009,06	35	68	3,09E+07
tr|Q712N6|Q712N6_BUBBU	SPAQILQWQVLPNTVPAKSCQAQPTTMTR	3193,64	69	97	1,41E+06
tr|Q712N6|Q712N6_BUBBU	SPAQILQWQVLPNTVPAK	1989,10	69	86	9,57E+07
tr|Q712N6|Q712N6_BUBBU	SCQAQPTTMTR	1222,54	87	97	1,60E+07
sp|P02755|LACB_BUBBU	LIVTQTMK	932,54	1	8	1,03E+08
sp|P02755|LACB_BUBBU	VAGTWYSLAMAASDISLLDAQSAPLR	2706,37	15	40	5,61E+08
sp|P02755|LACB_BUBBU	VYVEELKPTPEGDLEILLQK	2312,25	41	60	2,05E+08
sp|P02755|LACB_BUBBU	TKIPAVFK	902,56	76	83	1,26E+06
sp|P02755|LACB_BUBBU	IDALNENK	915,47	84	91	4,51E+06
sp|P02755|LACB_BUBBU	VLVLDTDYKK	1192,67	92	101	1,35E+07
sp|P02755|LACB_BUBBU	VLVLDTDYK	1064,58	92	100	6,36E+08
tr|H9CH53|H9CH53_BUBBU	VLDTDYKK	980,52	94	101	1,60E+06
tr|H9CH53|H9CH53_BUBBU	VLDTDYK	852,42	94	100	1,25E+07
sp|P02755|LACB_BUBBU	TPEVDDEALEKFDK	1634,77	125	138	5,77E+05
sp|P02755|LACB_BUBBU	TPEVDDEALEK	1244,58	125	135	3,14E+06
sp|P02755|LACB_BUBBU	ALPMHIR	836,47	142	148	1,39E+07
